# Supplementary material for: Oligomerised RIPK1 is the main core component of the CD95 necrosome
Source: EMBO J. 2025 Apr 16;44(11):3231–65. doi: 10.1038/s44318-025-00433-0 (PMC12130296; doi:10.1038/s44318-025-00433-0)
Supplement: Supplementary file 1 — Appendix [file 44318_2025_433_MOESM1_ESM.pdf]

Appendix

Oligomerised RIPK1 is the main core component of the CD95 necrosome

Nikita V. Ivanisenko[1#], Corinna König[1#], Laura K Hillert-Richter[1#], Maria A. Feoktistova[2], Sabine Pietkiewicz[1], Max Richter[1], Diana Panayotova-Dimitrova[2], Thilo Kaehne[3], Inna N. Lavrik[1,*]

Table of contents

Appendix Figure S1 3
Appendix Figure S2 5
Appendix Figure S3 7
Appendix Figure S4 9
Appendix Figure S5 10
Appendix Figure S6 12
Appendix Figure S7 14
Appendix Figure S8 15
Appendix Figure S9 16
Appendix Figure S10 17
Appendix Figure S11 18
Appendix Figure S12 19
Appendix Table S1 20
Appendix Table S2 20
Appendix Table S3 22

Appendix Figure S1

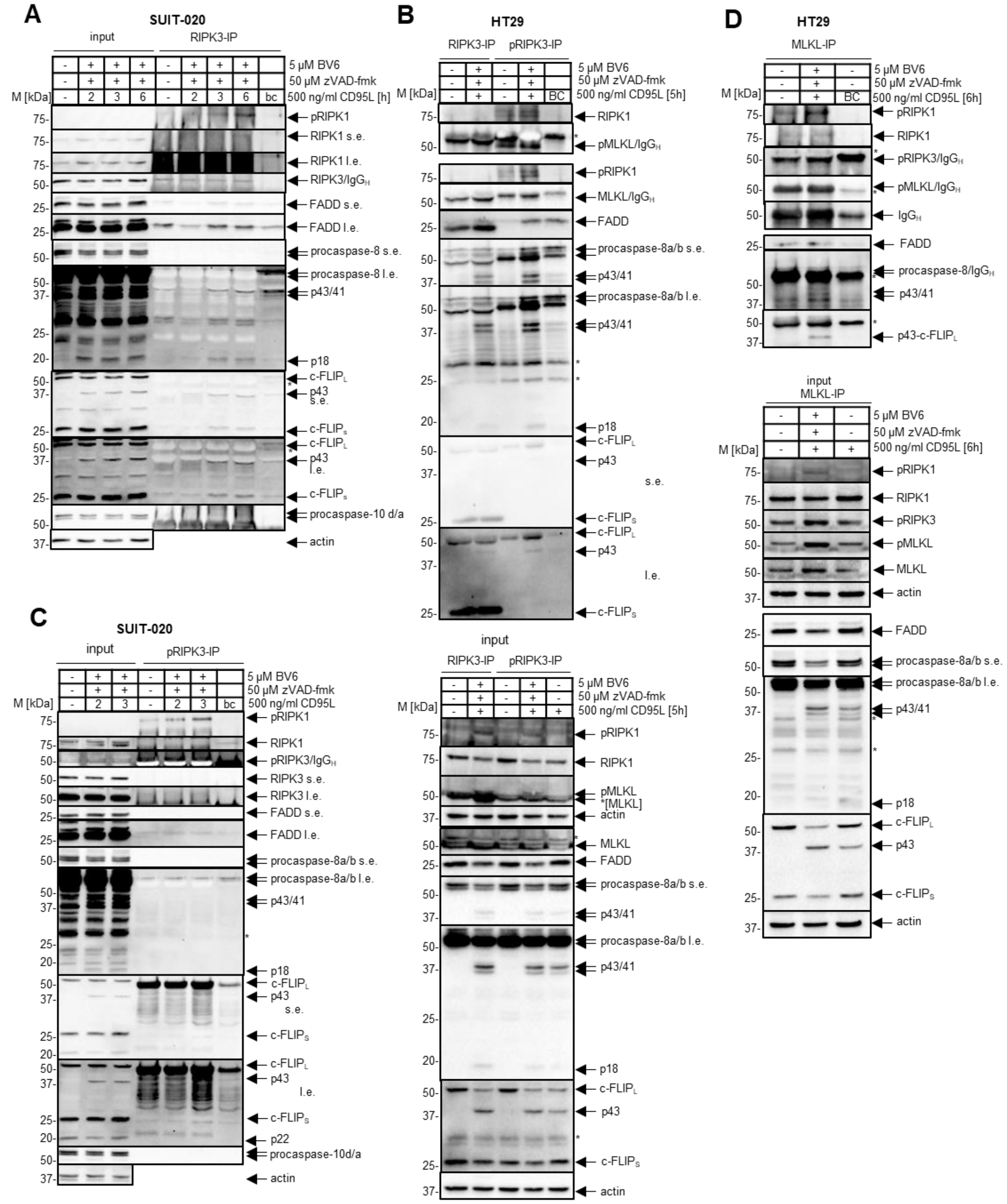

Appendix Figure S1: CD95L/BV6/zVAD-fmk-induced co-immunoprecipitation of the core necrosome components using anti-pRIPK3, anti-RIPK3 and anti-MLKL antibodies (**A-D**) SUIT-020 (**A, C**) or HT29 (**B, D**) cells were pretreated with 5 µM BV6 and 50 µM zVAD-fmk for 1 h. Afterwards cells were stimulated with 500 ng/mL CD95L for 5 h (**B**) or 6 h (**D**) or indicated timepoints (**A, C**), which was followed by RIPK3-IP (**A**), pRIPK3-IP (**B, C**) or MLKL-IP (**D**) using anti-RIPK3, anti pRIPK3 or anti-MLKL antibodies respectively. IPs were analyzed using Western Blot and actin served as loading control for total cellular lysates (inputs). One representative Western Blot out of three (**B, D**) or two (**A, C**) is shown. Abbreviations: s.e. short exposure, l.e. long exposure, IP Immunoprecipitation, BC beads only control, *-unspecific band, IgGH-the heavy chain of antibody

Appendix Figure S2

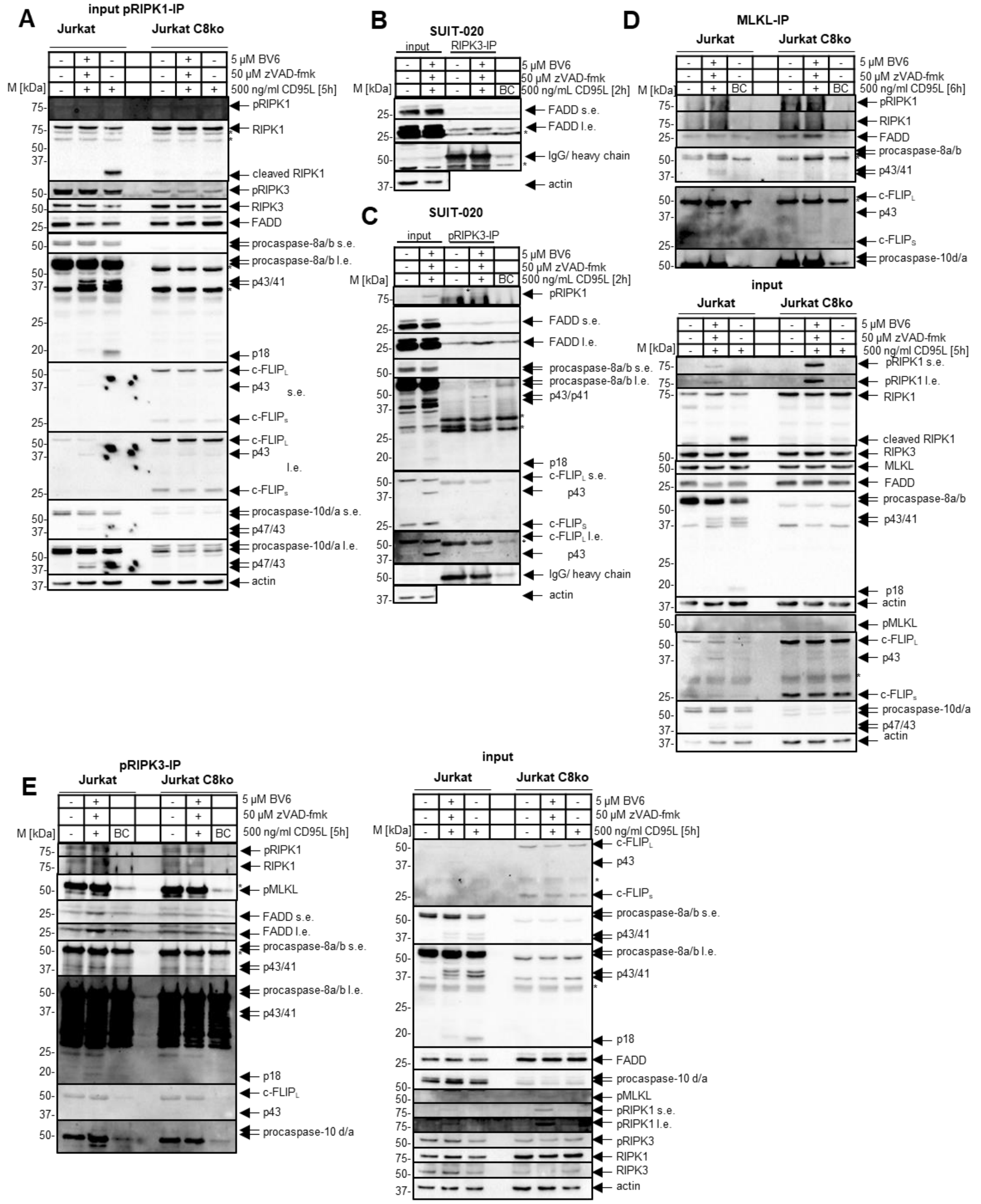

Appendix Figure S2: **CD95L/BV6/zVAD-fmk-induced co-immunoprecipitation of the core necrosome components** (**A-E**) Jurkat and Jurkat C8 ko (**A, D, E**) or SUIT-020 (**B, C**) cells were pretreated with 5 µM BV6 and 50 µM zVAD-fmk for 1 h. Afterwards cells were stimulated with 500 ng/mL CD95L for 5 h (**A, E**) or 6 h (**D**) or 2 h (**B, C**), which was followed by RIPK3-IP (**B**), pRIPK3-IP (**C, E**) or MLKL-IP (**D**) using anti-RIPK3, anti pRIPK3 or anti-MLKL antibodies respectively. (**A**) shows the input to figure 2D. IPs were analyzed using Western Blot and actin served as loading control for total cellular lysates (inputs). One representative Western Blot out of three (**A, D, E**) or two (**B, C**) is shown. Abbreviations: s.e. short exposure, l.e. long exposure, IP Immunoprecipitation, BC beads only control

Appendix Figure S3

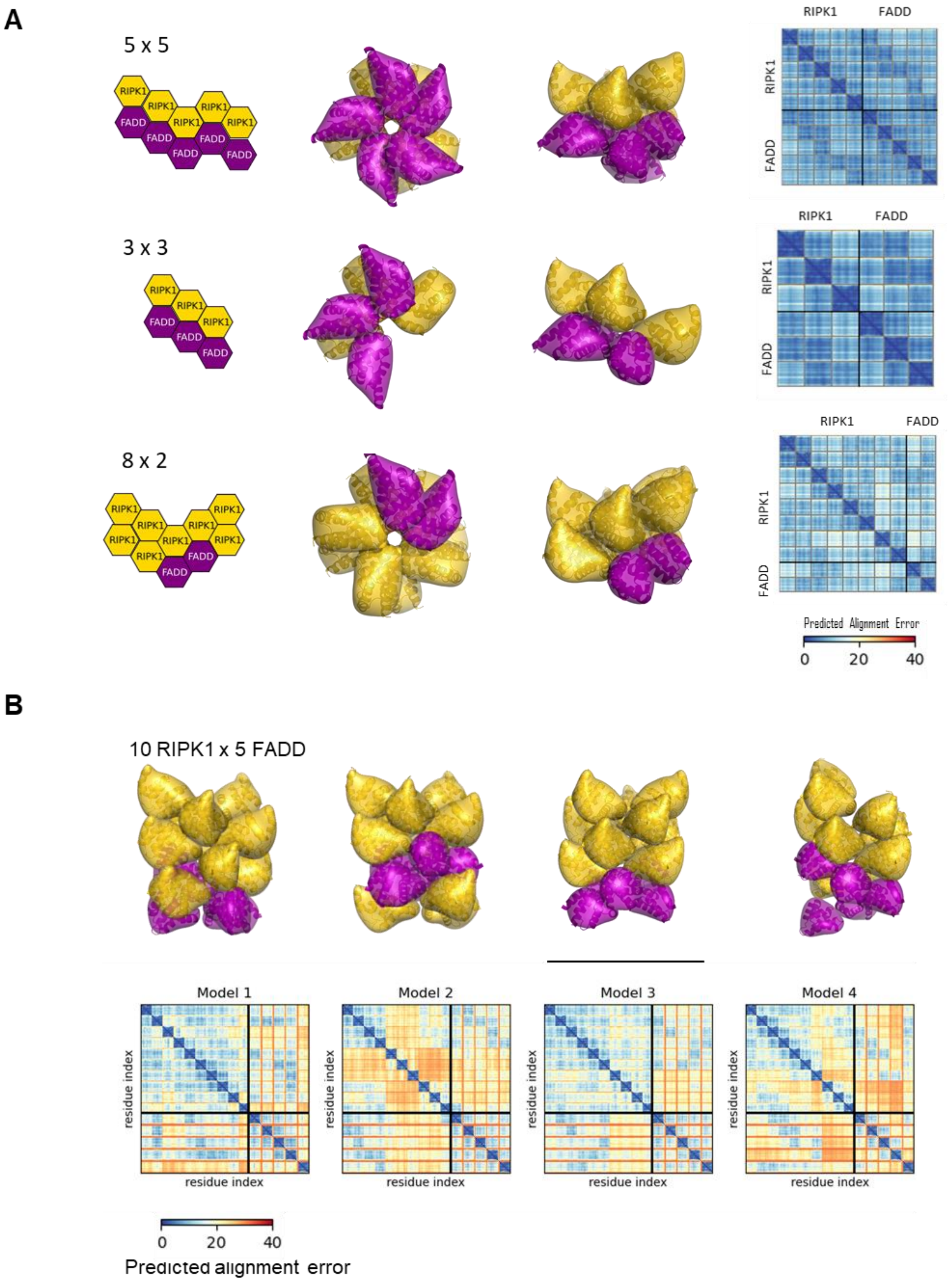

Appendix Figure S3: Molecular models of oligomers predicted by AlphaFold2-Multimer for different RIPK1 and FADD stoichiometries (**A**) Molecular models for 5 RIPK1 x 5 FADD, 3 RIPK1 x 3 FADD and 8 RIPK1 x 2 FADD. The molecular model is displayed on the left, while the heatmap indicating the predicted alignment error score is presented at the right. Predicted alignment errors are color-coded from red (indicating high error) to blue (indicating low error). Results of predictions for model weights of AlphaFold-Multimer with highest ipTM score are presented. (**B**) Molecular models for 10 RIPK1 x 5 FADD composition. The model with the highest ipTM score is underlined. The molecular model is displayed on the top, while the heatmap indicating the predicted alignment error score is presented at bottom.

Appendix Figure S4

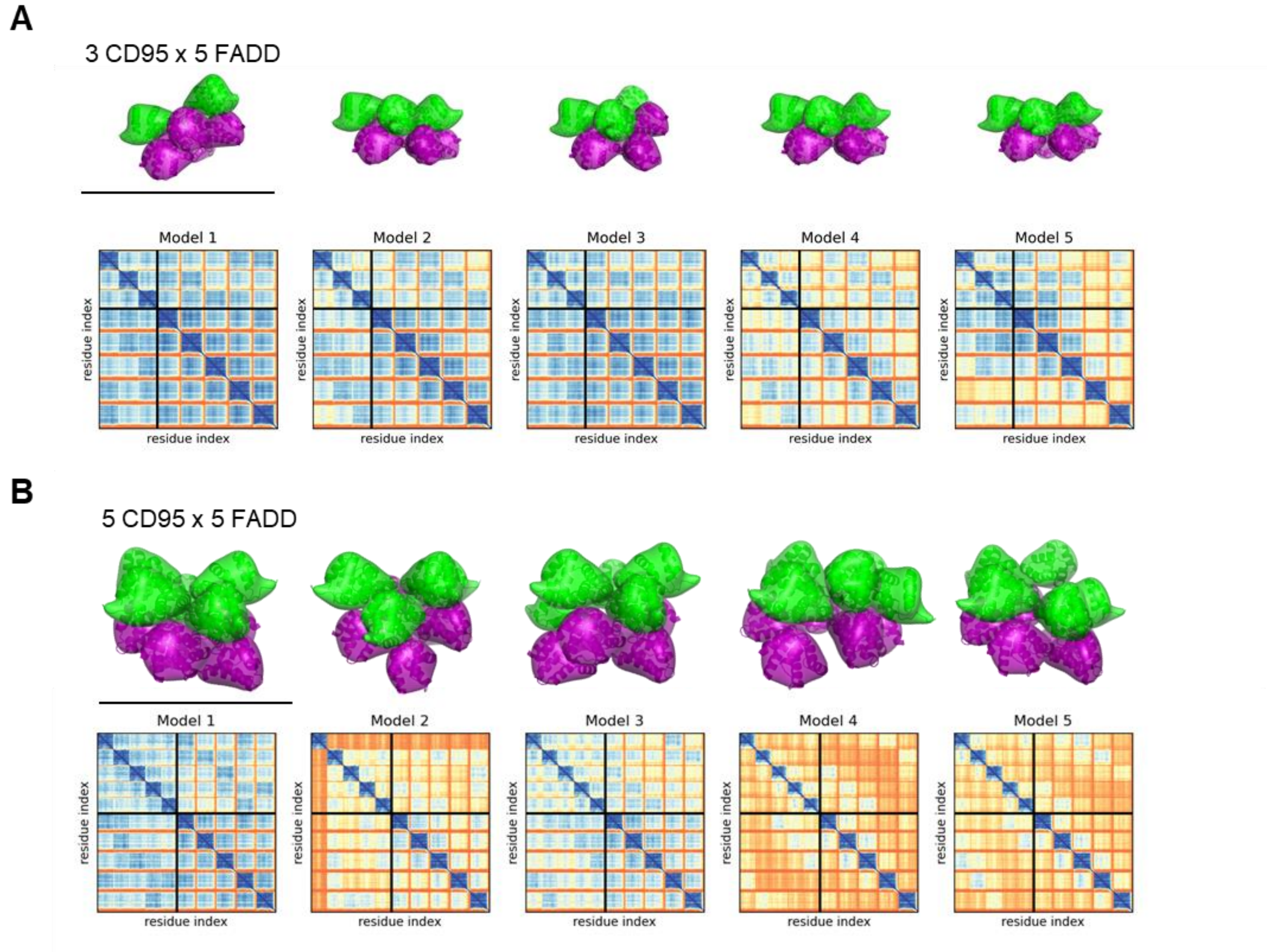

Appendix Figure S4: Molecular models of oligomers predicted by AlphaFold2-Multimer for different FADD and CD95 composition (**A, B**) Molecular models of oligomers predicted by AlphaFold-Multimer for stoichiometry 3 CD95 x 5 FADD (**A**), 5 CD95 x 5 FADD (**B**). The molecular model is displayed at the top, while the heatmap indicating the predicted alignment error score is presented at the bottom. Predicted alignment errors are color-coded from red (indicating high error) to blue (indicating low error). Results of predictions for different weights of AlphaFold-Multimer-v3 models are presented. Models with the highest ipTM score are underlined.

Appendix Figure S5

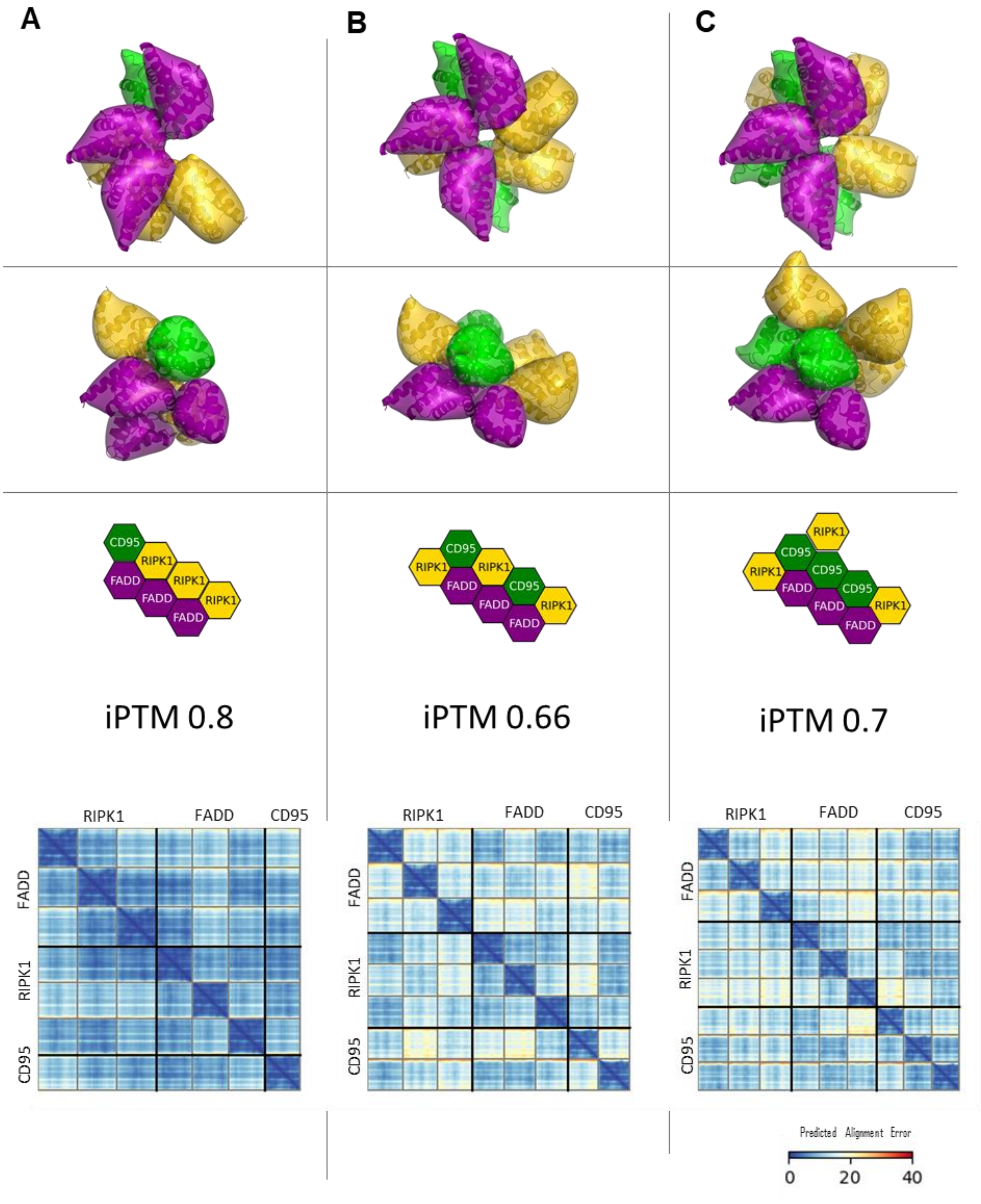

Appendix Figure S5: Molecular Models of CD95 DD, FADD DD and RIPK1 DD interaction (**A-C**) Molecular models of 1 CD95 x 3 FADD x 3 RIPK1 (**A**), 2 CD95 x 3 RIPK1 x 3 FADD (**B**) and 3 CD95 x 3 RIPK1 x 3 FADD (**C**) oligomers predicted by AlphaFold-Multimer-v3 are shown. Molecular model is shown on the top, heatmap showing predicted alignment error score is shown on the bottom. Predicted alignment error is colored from red (high error) to blue (low error). Results of predictions for models with highest ipTM score are shown. ipTM scores for each model is denoted.

Appendix Figure S6

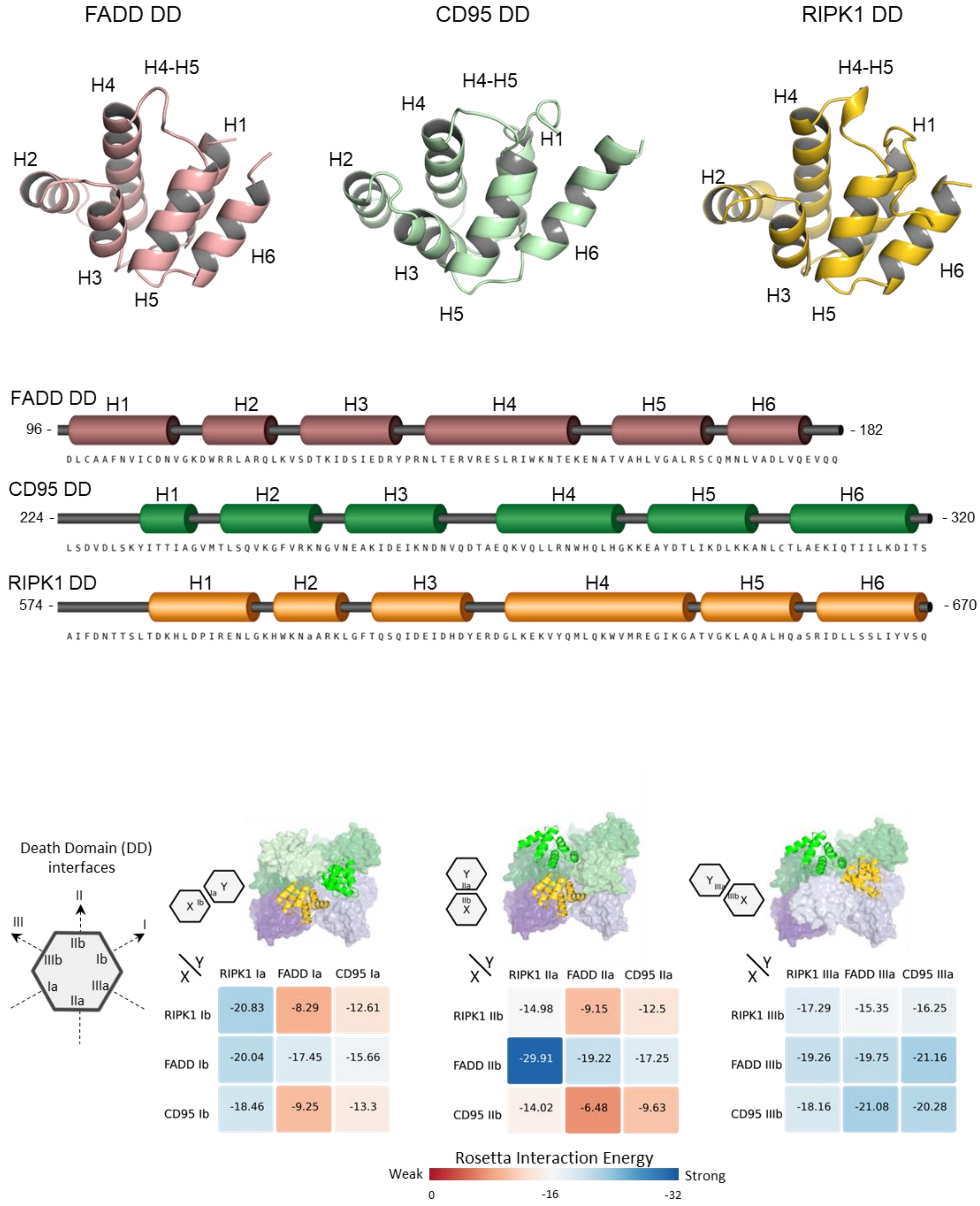

Appendix Figure S6: Structure of FADD, CD95 and RIPK1 DDs. Tertiary structure is shown on the top and primary and secondary structures are shown in the middle. DD helices are denoted. Protein regions and corresponding interacting interfaces within the DD oligomer are denoted in gray. Bottom: Predicted interaction energies for pairwise type I, II and III interactions of CD95, RIPK1 and FADD DDs are shown as heatmaps. Color gradient indicates Rosetta interaction energy from low (red color) to strong interaction (blue color). Predicted interaction energies and types of interaction with DD oligomer are denoted

Appendix Figure S7

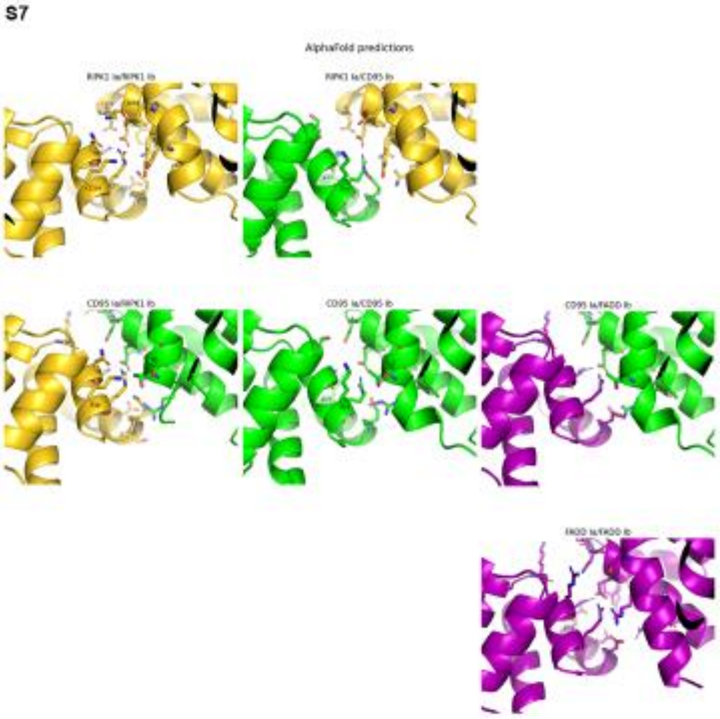

Appendix Figure S7: Structural model of type I interaction of DDs predicted by AlphaFold-Multimer-v3 CD95 DDs are shown in green color, FADD DDs in purple, RIPK1 DD in gold. Interacting subunits and corresponding interfaces are denoted.

Appendix Figure S8

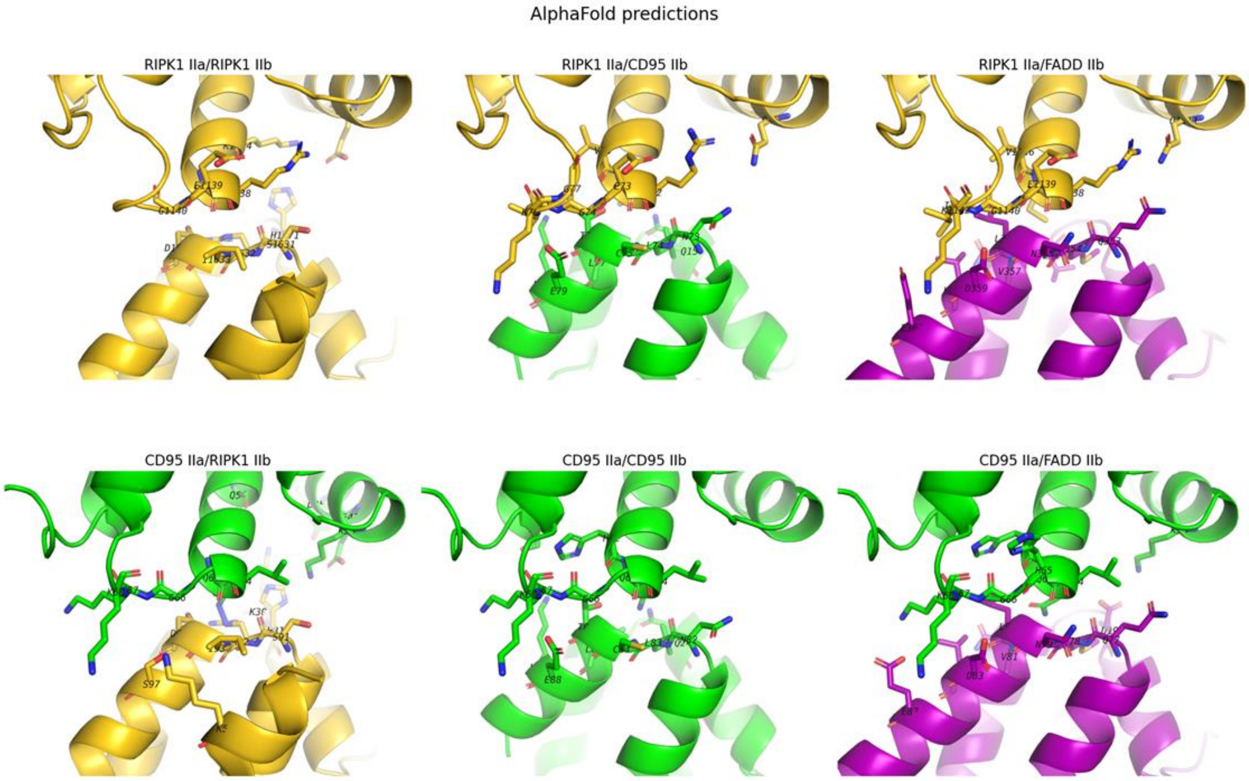

Appendix Figure S8: Structural model of type II interaction of DDs predicted by AlphaFold-Multimer-v3 CD95 DDs are shown in green color, FADD DDs in purple, RIPK1 DD in gold. Interacting subunits and corresponding interfaces are denoted.

Appendix Figure S9

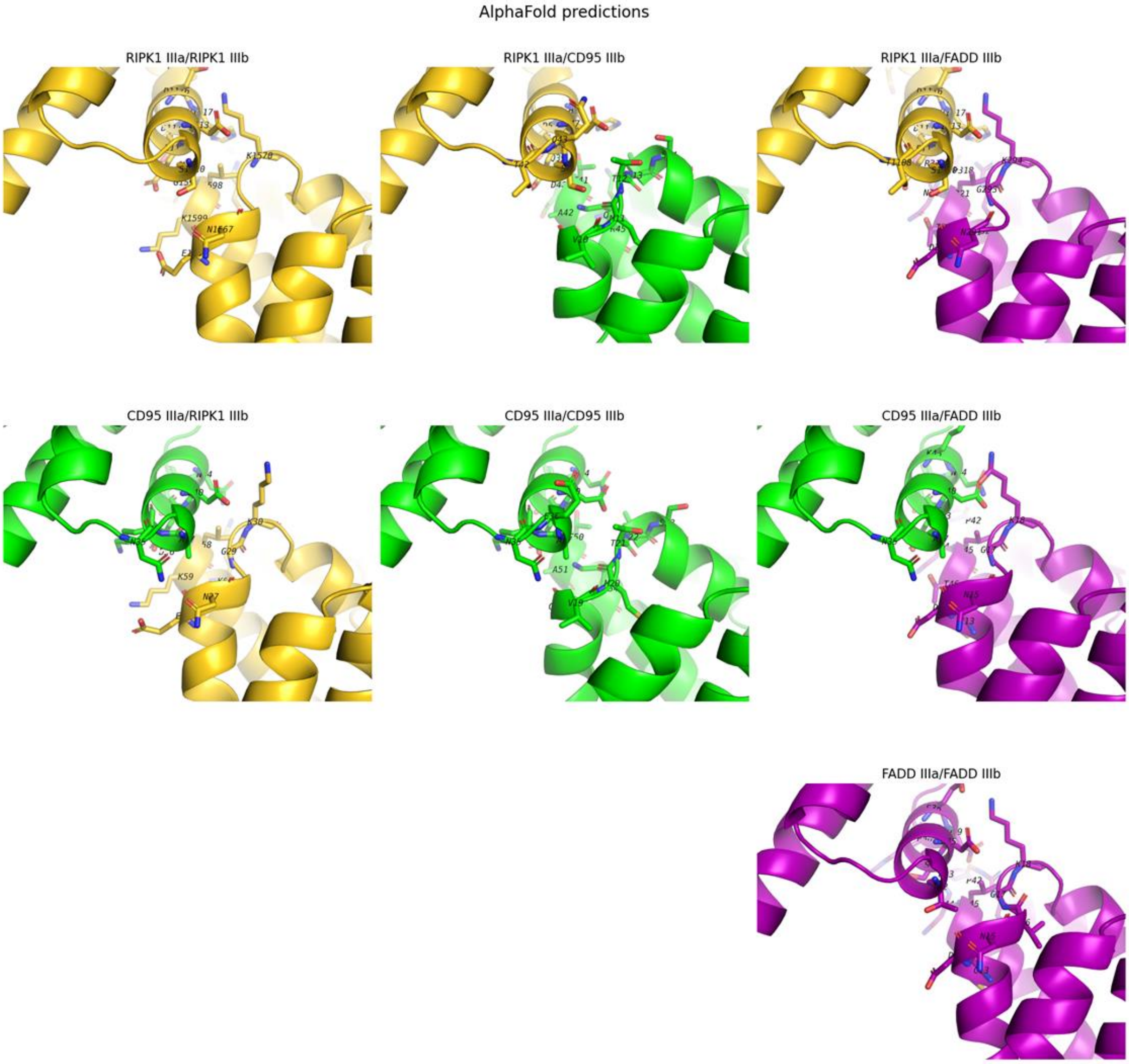

Appendix Figure S9: Structural model of type III interaction of DDs predicted by AlphaFold-Multimer-v3 CD95 DDs are shown in green color, FADD DDs in purple, RIPK1 DD in gold. Interacting subunits and corresponding interfaces are denoted.

Appendix Figure S10

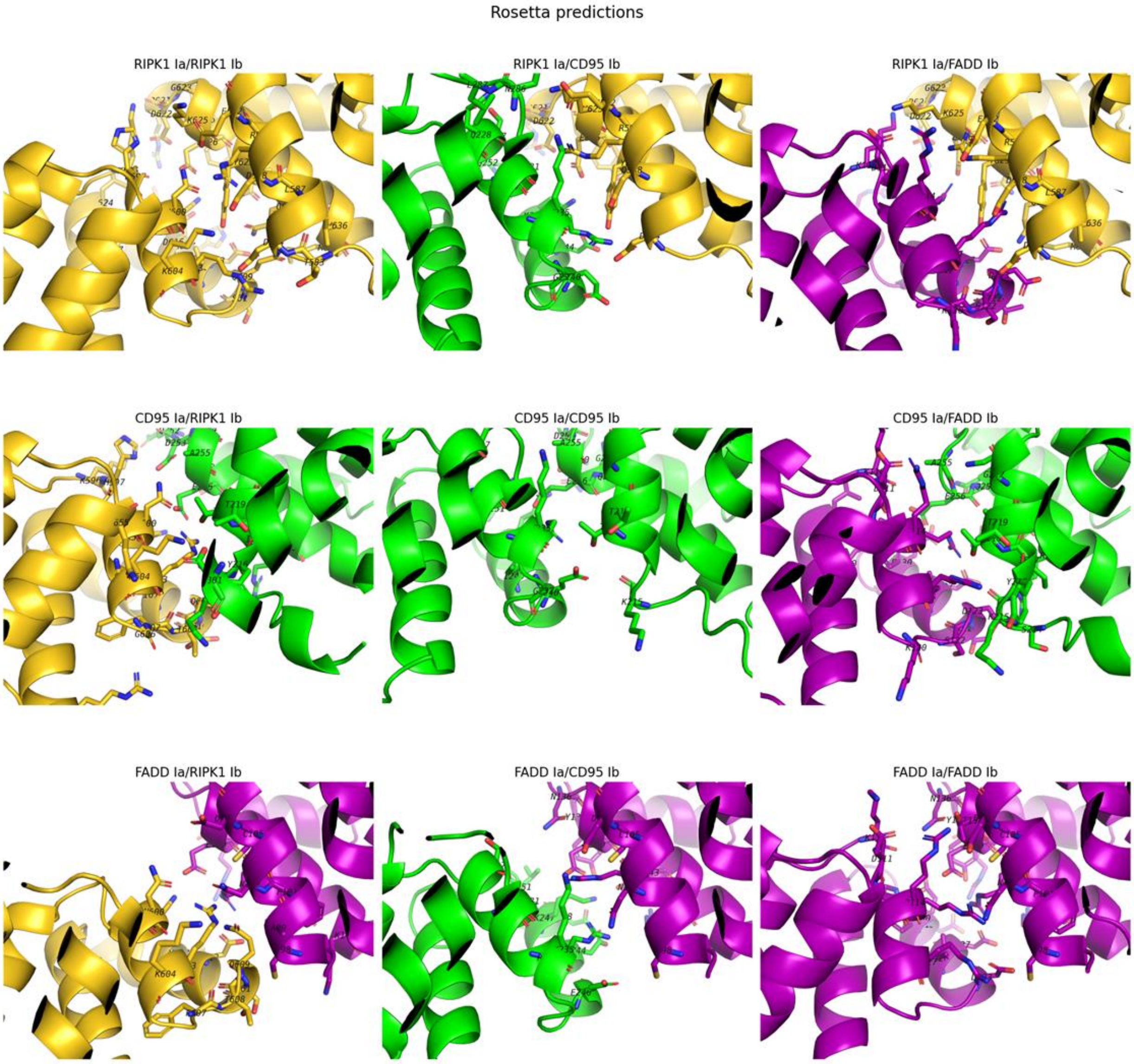

Appendix Figure S10: Structural model of type I interaction of DDs predicted by Rosetta protocol CD95 DDs are shown in green color, FADD DDs in purple, RIPK1 DD in gold. Interacting subunits and corresponding interfaces are denoted. Complexes with the best predicting binding scores are shown.

Appendix Figure S11

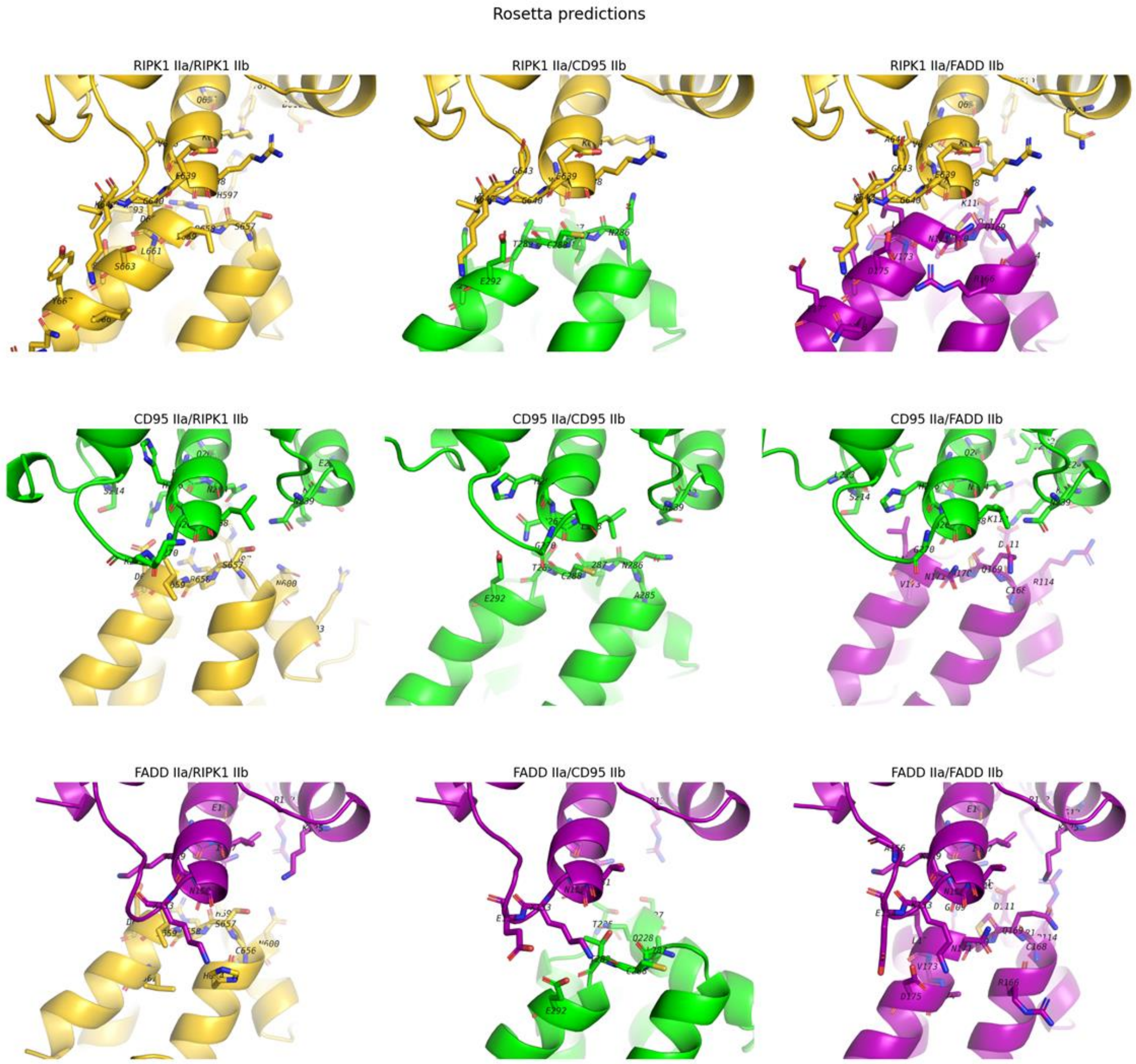

Appendix Figure S11: Structural model of type II interaction of DDs predicted by Rosetta protocol CD95 DDs are shown in green color, FADD DDs in purple, RIPK1 DD in gold. Interacting subunits and corresponding interfaces are denoted. Complexes with the best predicting binding scores are shown.

Appendix Figure S12

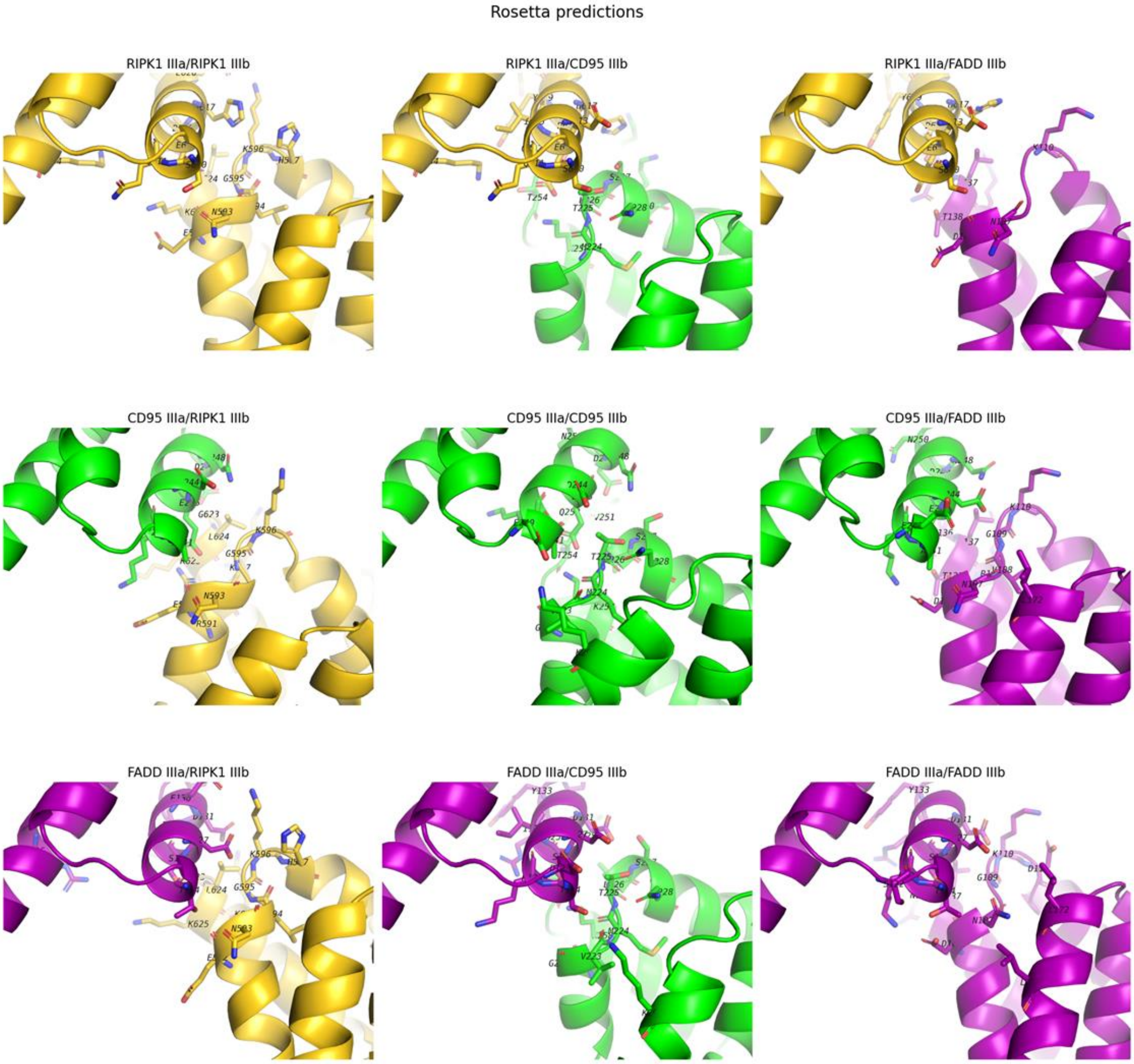

Appendix Figure S12: **Structural model of type III interaction of DDs predicted by Rosetta protocol** CD95 DDs are shown in green color, FADD DDs in purple, RIPK1 DD in gold. Interacting subunits and corresponding interfaces are denoted. Complexes with the best predicting binding scores are shown.

Protocol for structural modeling and pairwise binding energy estimation via Rosetta

1. The RIPK1, FADD, CD95 DD were obtained from PDB database according to PDB identifiers and protein regions indicated in Appendix Table S1.
2. For all other monomers (Appendix Table S1) the minimization using FastRelax protocol (Relax.xml script) was carried out generating 2500 conformations. Prior to this step, the N- and C-terminal residues were amydated and acetylated using PyMOL software. From 2500 models, the 50 models with the lowest score were selected.
3. The initial models of homo- and heterodimers were generated using PyMOL by superimposing minimized monomers on the cryo-EM structure of mouse CD95/human FADD complex (PDB ID 3OQ9) and selecting those that interact *via* type I, II or type III interactions. The interacting interfaces, types of interactions and corresponding chains in the PDB structures used for this step were taken from table S2 or table S3. For each pair >2500 models were generated and side chains on the interacting interface were optimized (relax_mono.xml script). 100 complexes with the lowest energy were selected.
4. Each selected complex was refined using 40 runs of Relax and High Resolution Protein-Protein Docking protocols of the Rosetta Software Package using refinement.xml RosettaScripts XML protocol. This protocol included small translational and rotational perturbations of interacting subunits in the course of Monte-Carlo minimization search. In this case all residues belonging to interacting subunits within 15 A were considered for optimization. This step was followed by optimization of the side chains using hbnet scoring function (Leman *et al*, 2020) that provides the bonus for the formation of hydrogen bond networks and penalties for unsatisfied hydrogen bonds. Finally, the minimization of the system using ref15 scoring function was conducted and the binding score calculation using InterfaceAnalyzer mover was carried out.
5. To ensure that generated dimers didn’t deviate from the reference structure, generated complexes that had RMSD value higher than 1.5 Å were filtered out. 5% of the complexes with the lowest subunit interaction score (dG_cross) were selected and the average binding energy was used as the estimation of interaction energy.

Appendix Table S1. Protein regions and PDB identifiers used for pairwise interacting complexes generation

Protein name	Residues	PDB ID	Reference
RIPK1 DD	574-670	6AC5	(Ding *et al*, 2019)
CD95 DD	208-304	1DDF	(Huang *et al*, 1996)
FADD DD	96-182	6ACI	(Ding *et al.*, 2019)

Appendix Table S2. PDB chains in the mouse CD95 / human FADD DD oligomer complex that were used for structural superimposition of RIPK1, CD95 and FADD DDs.

Inteface A	PDB Chain for interface A	Interface B	Chain of interface B	PDB ID
Ia	B	Ib	H	3OQ9 (Wang *et al*, 2010)
IIa	B	IIb	I	3OQ9 (Wang *et al.*, 2010)
IIIb	J	IIIa	B	3OQ9 (Wang *et al.*, 2010)

Rosetta Scripts and commands used for modeling.

Structural superimposition

Structural alignment was carried out in PyMOL using command *“align target_structure, reference_structure & chain C & resi R1-R2, cycles=50”* , where target_structure was a mobile protein, reference_structure, chain C and residues were taken according to Appendix tables S1-2.

term_min.xml

```
<ROSETTASCRIPTS>
	<SCOREFXNS>
	  <ScoreFunction name="ref15s" weights="ref2015.wts" >
```

```
    </ScoreFunction>
  </SCOREFXNS>
  <RESIDUE_SELECTORS>
  </RESIDUE_SELECTORS>
  <TASKOPERATIONS>
  </TASKOPERATIONS>
  <FILTERS>
  </FILTERS>
  <MOVERS>
    <MinMover name="min_mover" scorefxn="ref15s" chi="true" bb="true"  />
  </MOVERS>
  <APPLY_TO_POSE>
  </APPLY_TO_POSE>
  <PROTOCOLS>
    <Add mover="min_mover" />
  </PROTOCOLS>
  <OUTPUT />
</ROSETTASCRIPTS>
```

Executing command
$ROSETTA3/bin/rosetta_scripts.static.linuxgccrelease -relax:jump_move false -s protein.pdb _HNQ true -no_optH false -out:pdb_gz -parser:protocol term_min.xml -nstruct 1 -in:file:fullatom -out:file:fullatom -ex1 -ex2 -use_input_sc -out:prefix a0 -overwrite

Relax.xml

```
<ROSETTASCRIPTS>
  <SCOREFXNS>
    <ScoreFunction name="ref15s" weights="ref2015.wts" >
    </ScoreFunction>
  </SCOREFXNS>
  <RESIDUE_SELECTORS>
  </RESIDUE_SELECTORS>
  <TASKOPERATIONS>
  </TASKOPERATIONS>
  <FILTERS>
  </FILTERS>
  <MOVERS>
    <FastRelax name="fr" />
  </MOVERS>
  <APPLY_TO_POSE>
  </APPLY_TO_POSE>
  <PROTOCOLS>
    <Add mover="fr" />
  </PROTOCOLS>
  <OUTPUT />
</ROSETTASCRIPTS>
```

Executing command
$ROSETTA3/bin/rosetta_scripts.static.linuxgccrelease -relax:jump_move false -s protein.pdb _HNQ true -no_optH false -out:pdb_gz -parser:protocol script.xml -nstruct 2500 -in:file:fullatom -out:file:fullatom -ex1 -ex2 -use_input_sc -out:prefix a0 -overwrite

Hetero_relax.xml

```
<ROSETTASCRIPTS>
  <SCOREFXNS>
    <ScoreFunction name="ref15s" weights="ref2015.wts" />
  </SCOREFXNS>
  <RESIDUE_SELECTORS>
  </RESIDUE_SELECTORS>
  <TASKOPERATIONS>
    <RestrictToInterfaceVector name="rtiv" chain1_num="1" chain2_num="2" CB_dist_cutoff="15.0" />
    <RestrictToRepacking name="repackonly" />
    <IncludeCurrent name="ic" />
  </TASKOPERATIONS>
  <FILTERS>
  </FILTERS>
  <MOVERS>
    <FastRelax name="relax" scorefxn="ref15s" repeats="1" task_operations="repackonly,ic,rtiv" >
        <MoveMap chi="true" bb="false" jump = "0" >
        </MoveMap>
    </FastRelax>
  </MOVERS>
  <APPLY_TO_POSE>
  </APPLY_TO_POSE>
  <PROTOCOLS>
    <Add mover="relax" />
  </PROTOCOLS>
  <OUTPUT />
</ROSETTASCRIPTS>"""
```

Executing command

```
$ROSETTA3/bin/rosetta_scripts.static.linuxgccrelease -in:file:fullatom -out:file:fullatom -packing:ex1 -no_optH false -_HNQ -packing:ex2 -score:weights ref2015.wts -s INPUT_FILE_NAME -packing:use_input_sc -parser:protocol het_relax.xml -nstruct 1 -use_input_sc -out:pdb_gz
```

refine.xml

```
<ROSETTASCRIPTS>
    <SCOREFXNS>
      <ScoreFunction name="ref15sfxn" weights="ref2015.wts" >
        <Reweight scoretype="hbnet" weight="1.0" />
        <Reweight scoretype="buried_unsatisfied_penalty" weight="1.0" />
      </ScoreFunction>
      <ScoreFunction name="ref15s" weights="ref2015.wts" >
      </ScoreFunction>
    </SCOREFXNS>
    <RESIDUE_SELECTORS>
    </RESIDUE_SELECTORS>
    <TASKOPERATIONS>
      <RestrictToInterfaceVector name="rtiv" chain1_num="1" chain2_num="2" CB_dist_cutoff="15.0" />
      <RestrictToRepacking name="repackonly" />
    </TASKOPERATIONS>
    <FILTERS>
      <Rmsd name="rmsd" superimpose="1" threshold="1.5" chains="1,2" />
    </FILTERS>
    <MOVERS>
      <FastRelax name="relax_interface" scorefxn="ref15sfxn" repeats = "1" task_operations="repackonly">
        <MoveMap chi="true" bb="false" jump = "0" >
        </MoveMap>
      </FastRelax>
      <Docking name="dock_high" score_low="score_docking_low" score_high="ref15sfxn" fullatom="1" local_refine="1" optimize_fold_tree="1" conserve_foldtree="0" design="0" task_operations="rtiv" jumps="1" />
      <MinMover name="min" scorefxn="ref15s" chi="1" bb="1" jump="1" tolerance="0.01" />
      <InterfaceAnalyzerMover name="fullanalyze" scorefxn="ref15sfxn" packstat="0" pack_input="0" jump="1" tracer="0" use_jobname="1" resfile="0" />
    </MOVERS>
    <APPLY_TO_POSE>
    </APPLY_TO_POSE>
    <PROTOCOLS>
      <Add mover_name="relax_interface" />
      <Add mover="dock_high" />
      <Add mover="min" />
      <Add filter_name = "rmsd" />
      <Add mover="fullanalyze" />
    </PROTOCOLS>
    <OUTPUT />
</ROSETTASCRIPTS>
```

Executing command

```
$ROSETTA3/bin/rosetta_scripts.static.linuxgccrelease -in:file:fullatom -out:file:fullatom -packing:ex1 -packing:ex2 -score:weights ref2015.wts -s INPUT_FILE_NAME -packing:use_input_sc -extrachi_cutoff 0 -parser:protocol dock.xml -partners A_B -nstruct 40 -docking -partners A_B -dock_pert 0 0 -dock_mcm_trans_magnitude 0.5 -dock_mcm_rot_magnitude 0.1 -docking_local_refine -run:max_retry_job 10 -use_input_sc -out:pdb_gz
```

Appendix Table S3. Calculated binding energies for pairwise interaction of DDs. Columns include subunit names (Subunit A and Subunit B), types of interfaces (Interface A and Interface B), interaction energies (dG_cross), and standard deviations (dG_cross, std).

Subunit A name	Interface A	Subunit B name	Interafce B	dG_cross	dG_cross, std
FADD	Ia	FADD	Ib	-17.45	1.67
FADD	IIa	FADD	IIb	-19.22	0.67
FADD	IIIa	FADD	IIIb	-19.75	1.12
FADD	Ia	RIPK1	Ib	-8.29	1.44
FADD	IIa	RIPK1	IIb	-9.15	1.3
FADD	IIIa	RIPK1	IIIb	-15.35	1.19
FADD	Ia	CD95	Ib	-9.25	2.45
FADD	IIa	CD95	IIb	-6.48	0.6
FADD	IIIa	CD95	IIIb	-21.08	1.08
RIPK1	Ia	FADD	Ib	-20.04	1.52
RIPK1	IIa	FADD	IIb	-29.91	0.99
RIPK1	IIIa	FADD	IIIb	-19.26	1.53
RIPK1	Ia	RIPK1	Ib	-20.83	1.39
RIPK1	IIa	RIPK1	IIb	-14.98	0.58
RIPK1	IIIa	RIPK1	IIIb	-17.29	1.15

RIPK1	Ia	CD95	Ib	-18.46	1.16
RIPK1	IIa	CD95	IIb	-14.02	0.81
RIPK1	IIIa	CD95	IIIb	-18.16	0.63
CD95	Ia	FADD	Ib	-15.66	1.59
CD95	IIa	FADD	IIb	-17.25	1.36
CD95	IIIa	FADD	IIIb	-21.16	1.51
CD95	Ia	RIPK1	Ib	-12.61	1.3
CD95	IIa	RIPK1	IIb	-12.5	1.34
CD95	IIIa	RIPK1	IIIb	-16.25	1.3
CD95	Ia	CD95	Ib	-13.3	1.66
CD95	IIa	CD95	IIb	-9.63	0.9
CD95	IIIa	CD95	IIIb	-20.28	1.42

Reference List

Ding J, Pan X, Du L, Yao Q, Xue J, Yao H, Wang DC, Li S, Shao F (2019) Structural and Functional Insights into Host Death Domains Inactivation by the Bacterial Arginine GlcNAcyltransferase Effector. *Mol Cell* 74: 922-935 e926

Huang B, Eberstadt M, Olejniczak ET, Meadows RP, Fesik SW (1996) NMR structure and mutagenesis of the Fas (APO-1/CD95) death domain. *Nature* 384: 638-641

Leman JK, Weitzner BD, Lewis SM, Adolf-Bryfogle J, Alam N, Alford RF, Aprahamian M, Baker D, Barlow KA, Barth P *et al* (2020) Macromolecular modeling and design in Rosetta: recent methods and frameworks. *Nat Methods* 17: 665-680

Wang L, Yang JK, Kabaleeswaran V, Rice AJ, Cruz AC, Park AY, Yin Q, Damko E, Jang SB, Raunser S *et al* (2010) The Fas-FADD death domain complex structure reveals the basis of DISC assembly and disease mutations. *Nat Struct Mol Biol* 17: 1324-1329
