# Supplementary material for: Oligomerised RIPK1 is the main core component of the CD95 necrosome
Source: EMBO J. 2025 Apr 16;44(11):3231–65. doi: 10.1038/s44318-025-00433-0 (PMC12130296; doi:10.1038/s44318-025-00433-0)
Supplement: Supplementary file 5 — Source data Fig. 1 [file 44318_2025_433_MOESM5_ESM.zip › figure1A.pptx]

## Slide 1
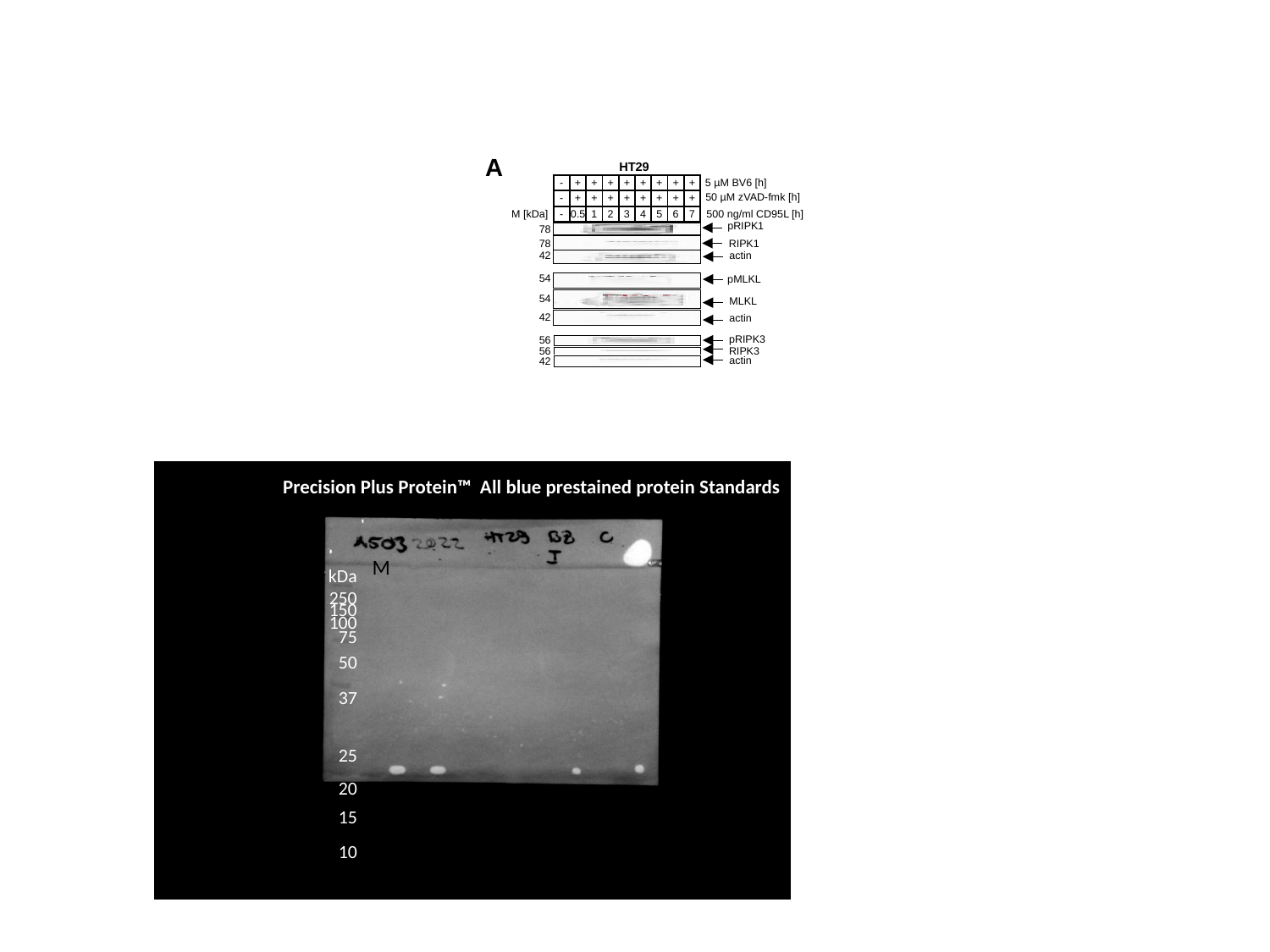

A
HT29
5 µM BV6 [h]
| - | + | + | + | + | + | + | + | + |
| --- | --- | --- | --- | --- | --- | --- | --- | --- |
| - | + | + | + | + | + | + | + | + |
| - | 0.5 | 1 | 2 | 3 | 4 | 5 | 6 | 7 |
50 µM zVAD-fmk [h]
500 ng/ml CD95L [h]
M [kDa]
pRIPK1
78
RIPK1
78
actin
42
54
pMLKL
54
MLKL
42
actin
pRIPK3
56
56
RIPK3
actin
42
Precision Plus Protein™ All blue prestained protein Standards
M
kDa
250
150
100
75
50
37
25
20
15
10

## Slide 2
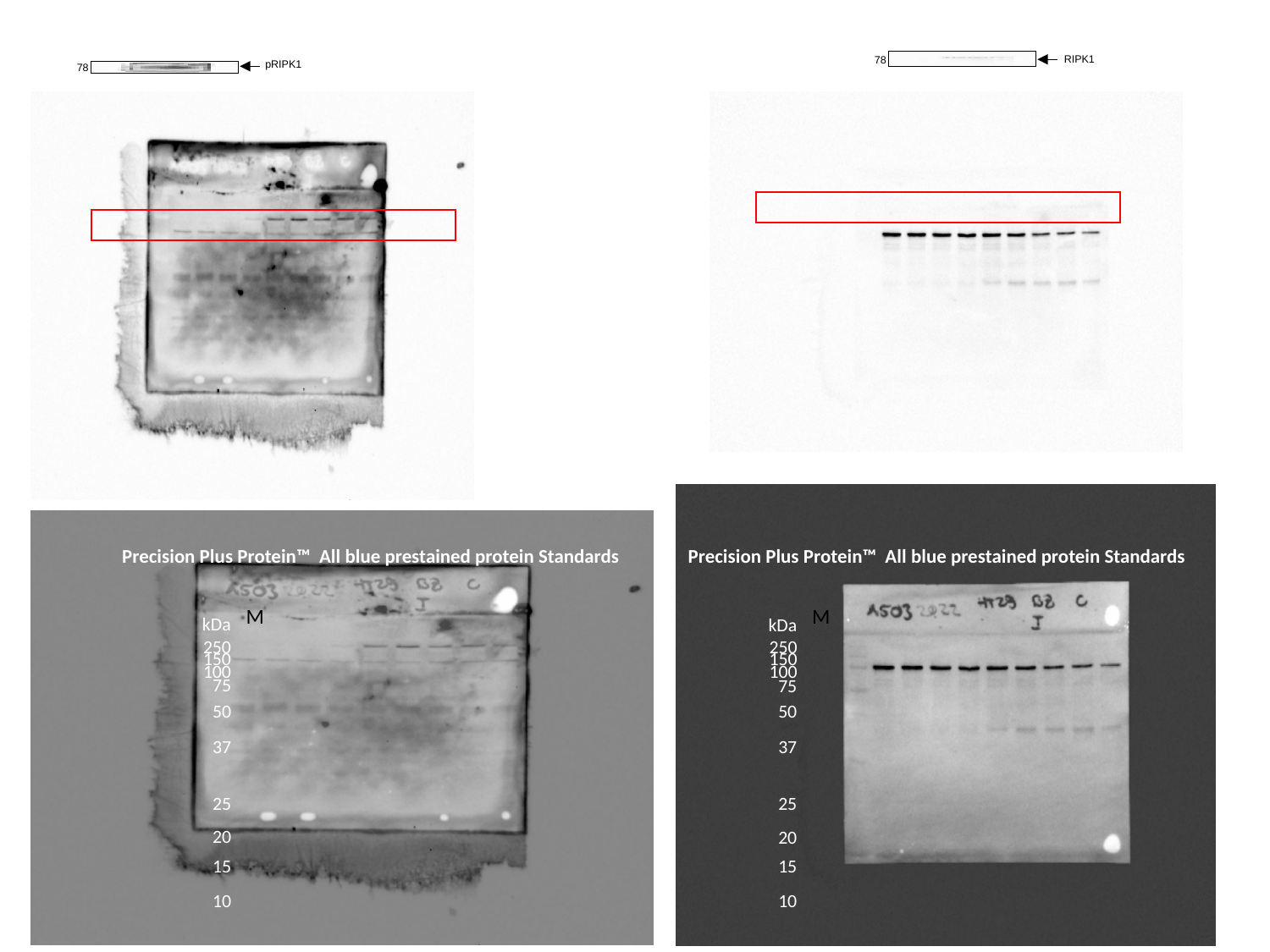

RIPK1
78
pRIPK1
78
Precision Plus Protein™ All blue prestained protein Standards
Precision Plus Protein™ All blue prestained protein Standards
M
M
kDa
kDa
250
250
150
150
100
100
75
75
50
50
37
37
25
25
20
20
15
15
10
10

## Slide 3
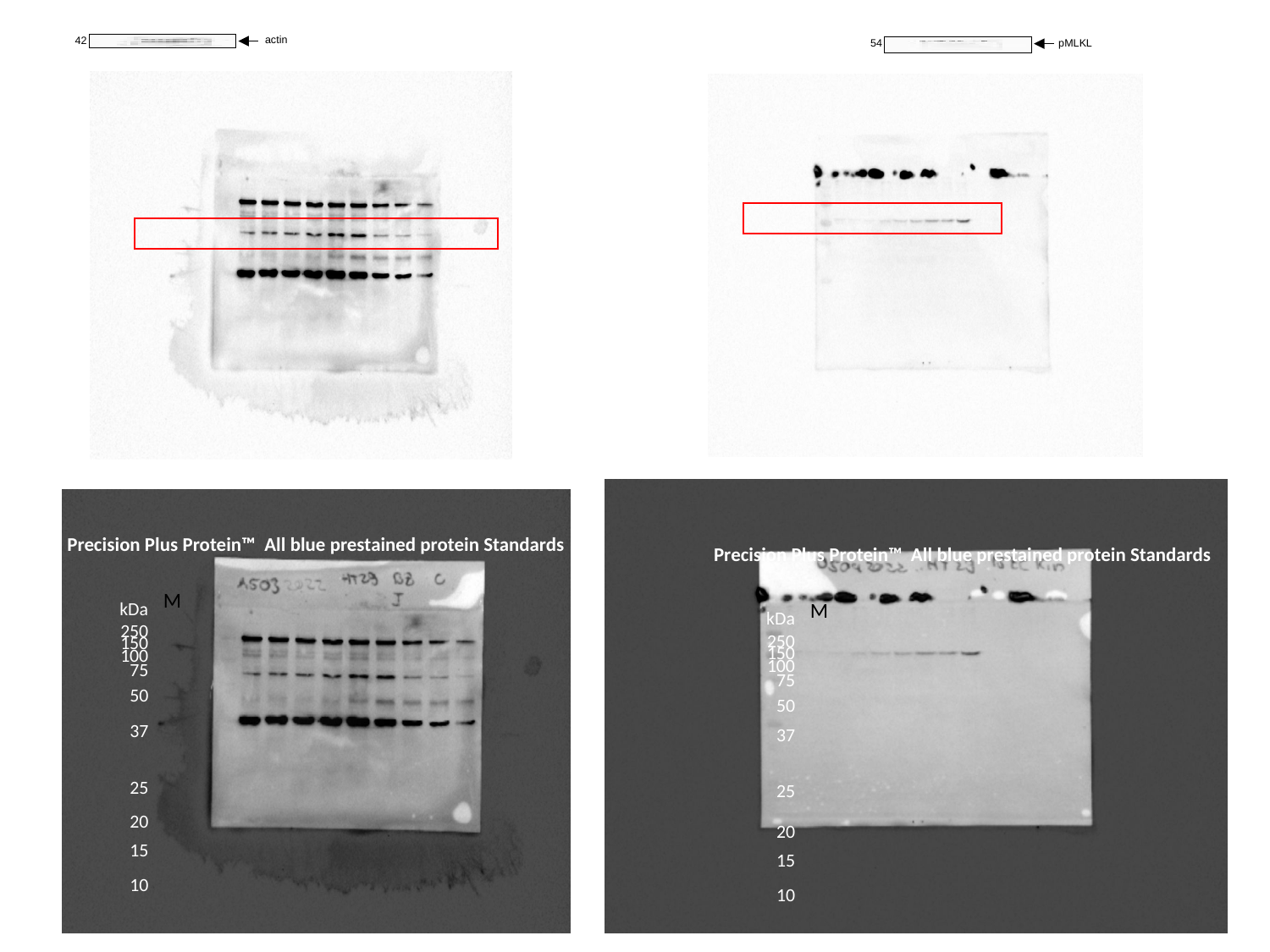

actin
42
54
pMLKL
Precision Plus Protein™ All blue prestained protein Standards
Precision Plus Protein™ All blue prestained protein Standards
M
M
kDa
kDa
250
250
150
150
100
100
75
75
50
50
37
37
25
25
20
20
15
15
10
10

## Slide 4
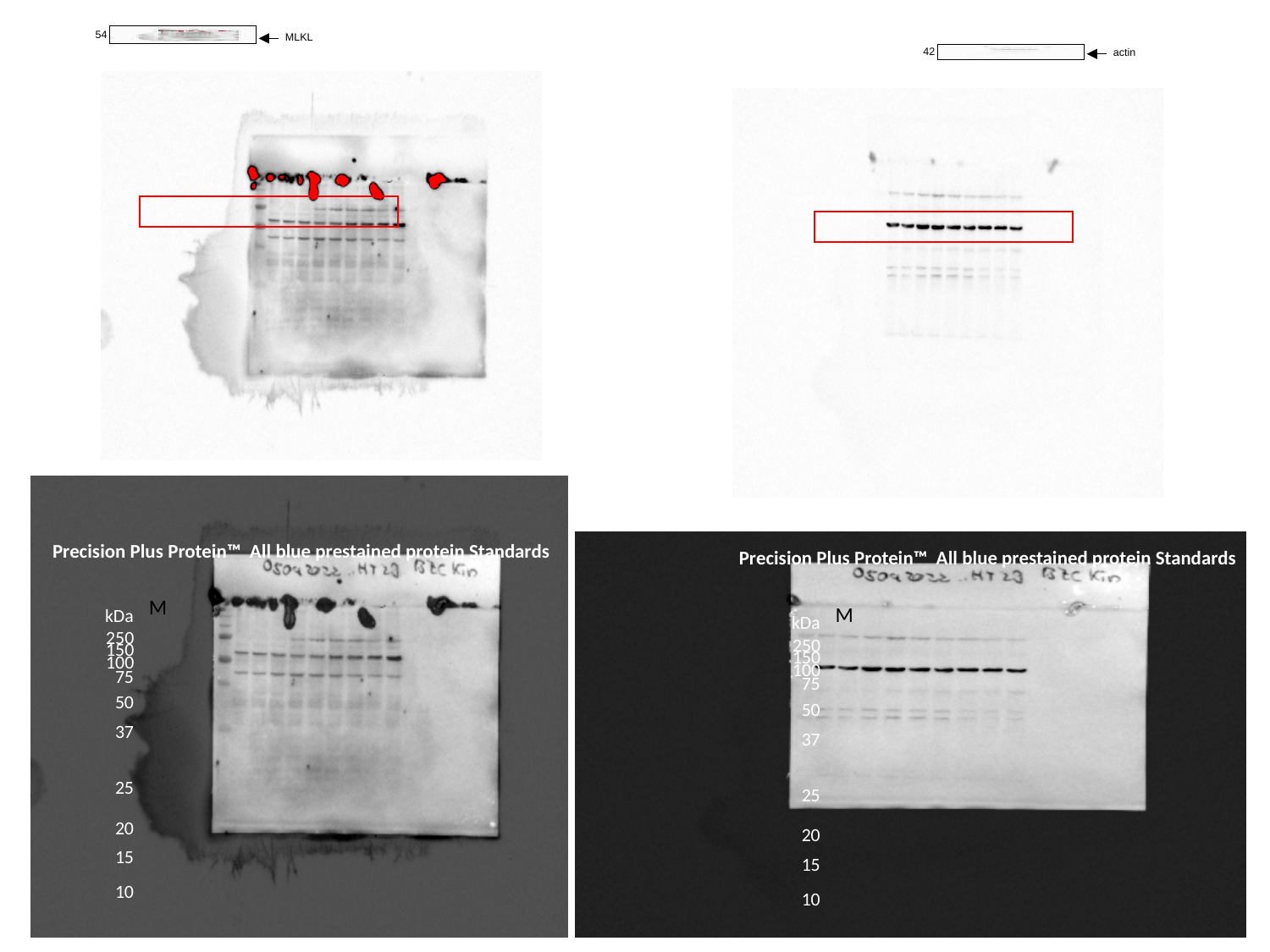

54
MLKL
42
actin
Precision Plus Protein™ All blue prestained protein Standards
Precision Plus Protein™ All blue prestained protein Standards
M
M
kDa
kDa
250
250
150
150
100
100
75
75
50
50
37
37
25
25
20
20
15
15
10
10

## Slide 5
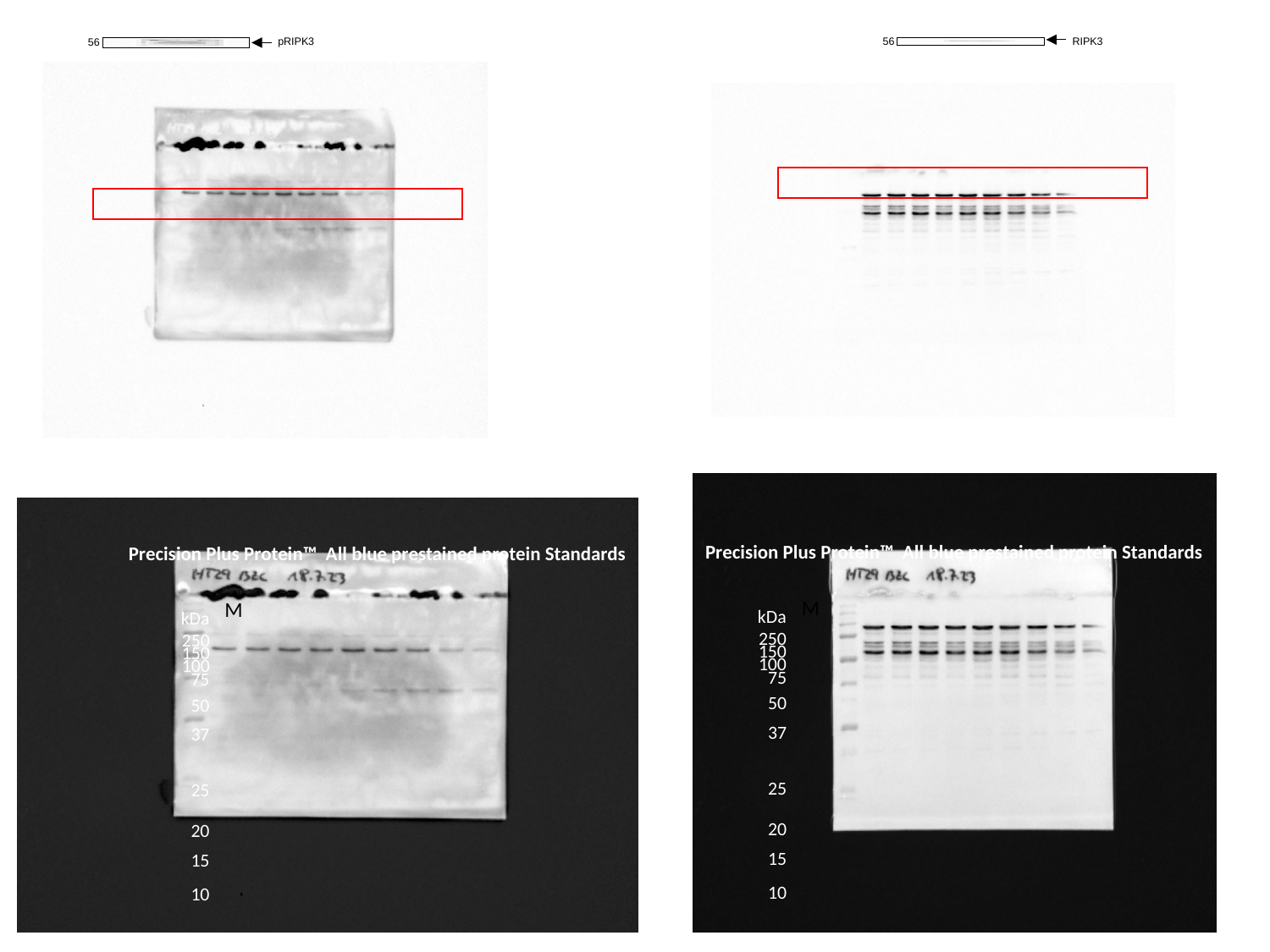

pRIPK3
56
RIPK3
56
Precision Plus Protein™ All blue prestained protein Standards
Precision Plus Protein™ All blue prestained protein Standards
M
M
kDa
kDa
250
250
150
150
100
100
75
75
50
50
37
37
25
25
20
20
15
15
10
10

## Slide 6
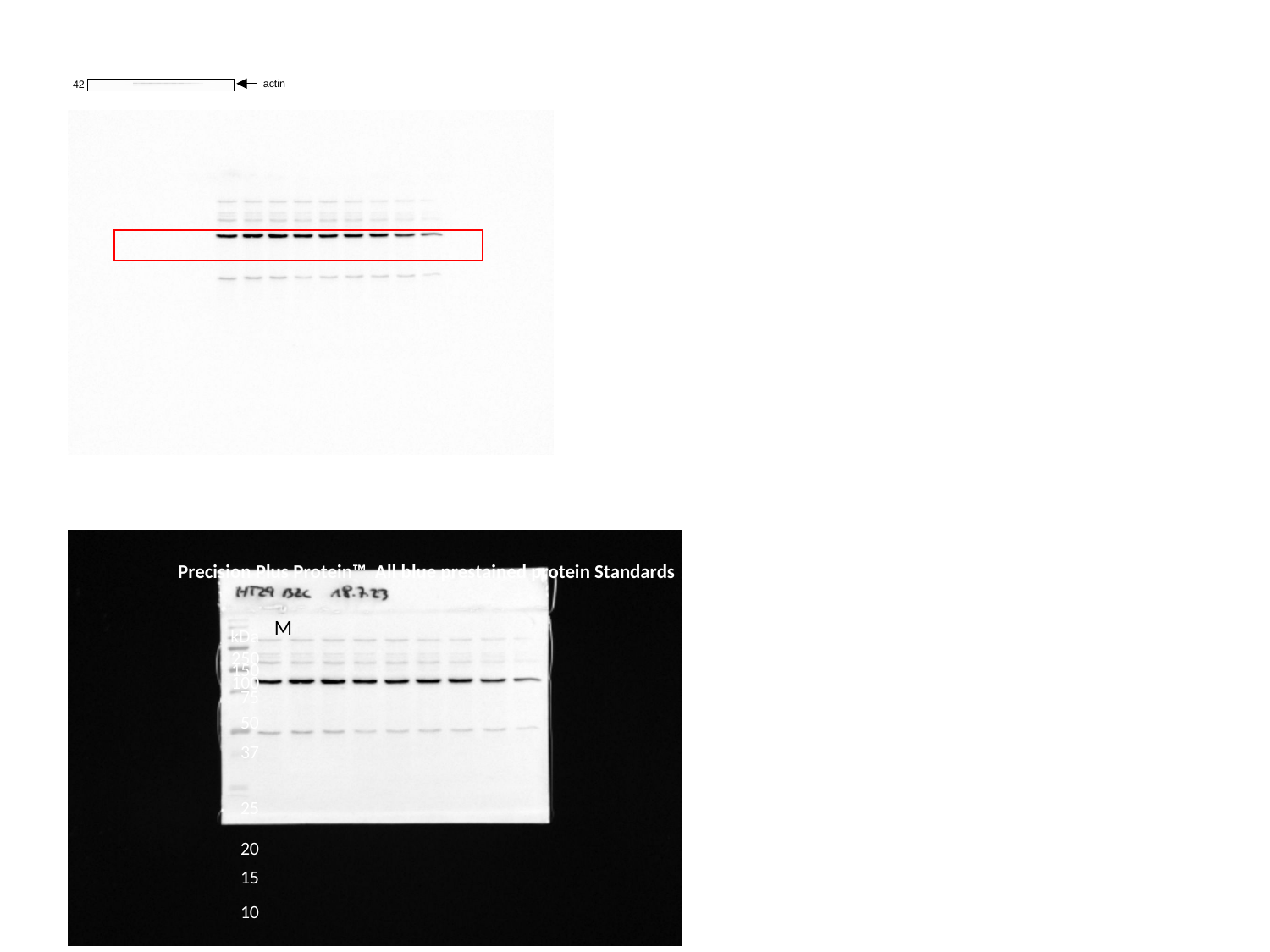

actin
42
Precision Plus Protein™ All blue prestained protein Standards
M
kDa
250
150
100
75
50
37
25
20
15
10
