# Supplementary material for: Oligomerised RIPK1 is the main core component of the CD95 necrosome
Source: EMBO J. 2025 Apr 16;44(11):3231–65. doi: 10.1038/s44318-025-00433-0 (PMC12130296; doi:10.1038/s44318-025-00433-0)
Supplement: Supplementary file 5 — Source data Fig. 1 [file 44318_2025_433_MOESM5_ESM.zip › figure1B.pptx]

## Slide 1
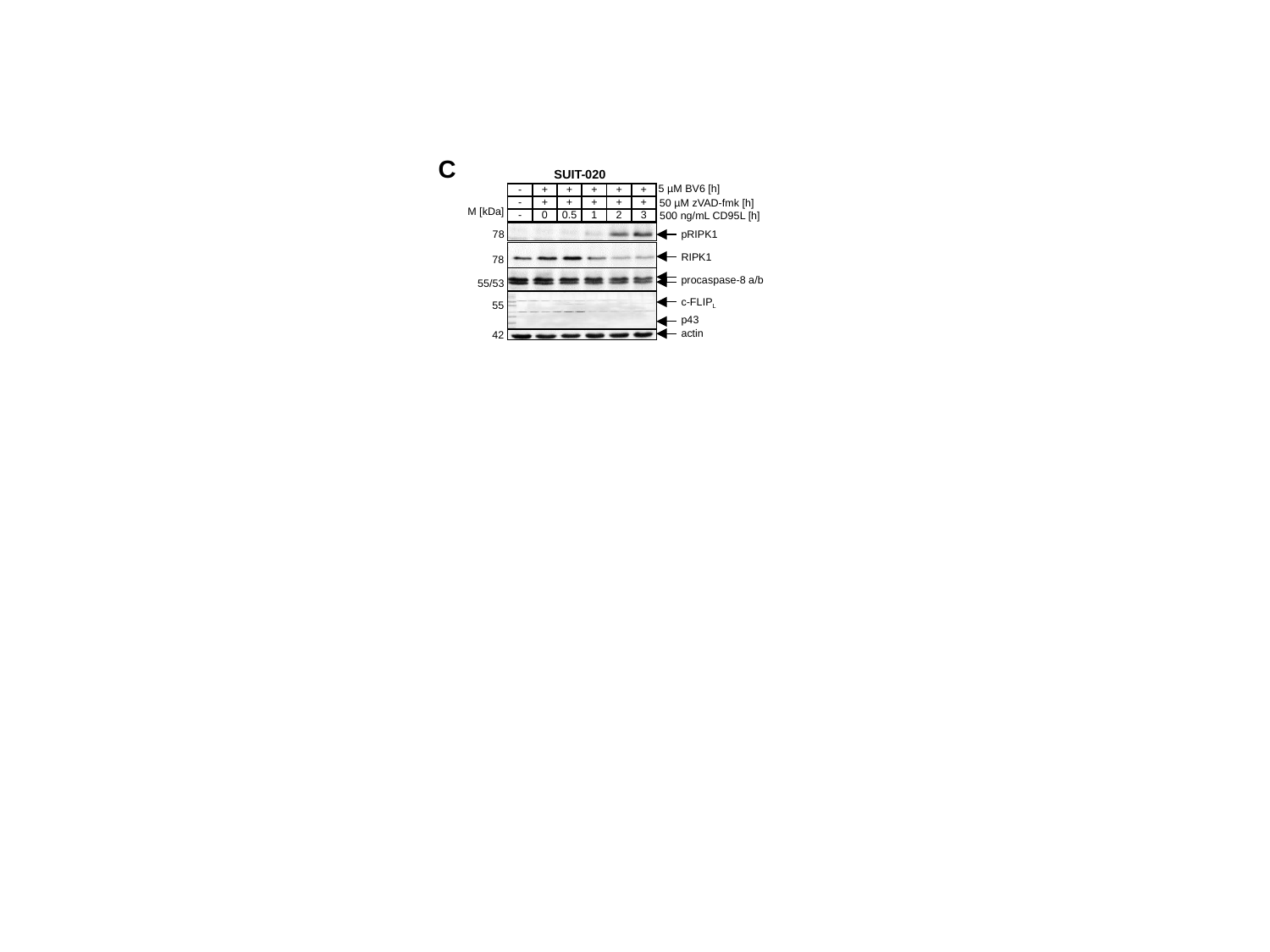

C
SUIT-020
5 µM BV6 [h]
| - | + | + | + | + | + |
| --- | --- | --- | --- | --- | --- |
| - | + | + | + | + | + |
| - | 0 | 0.5 | 1 | 2 | 3 |
50 µM zVAD-fmk [h]
M [kDa]
78
78
55/53
55
42
500 ng/mL CD95L [h]
pRIPK1
RIPK1
procaspase-8 a/b
c-FLIPL
p43
actin

## Slide 2
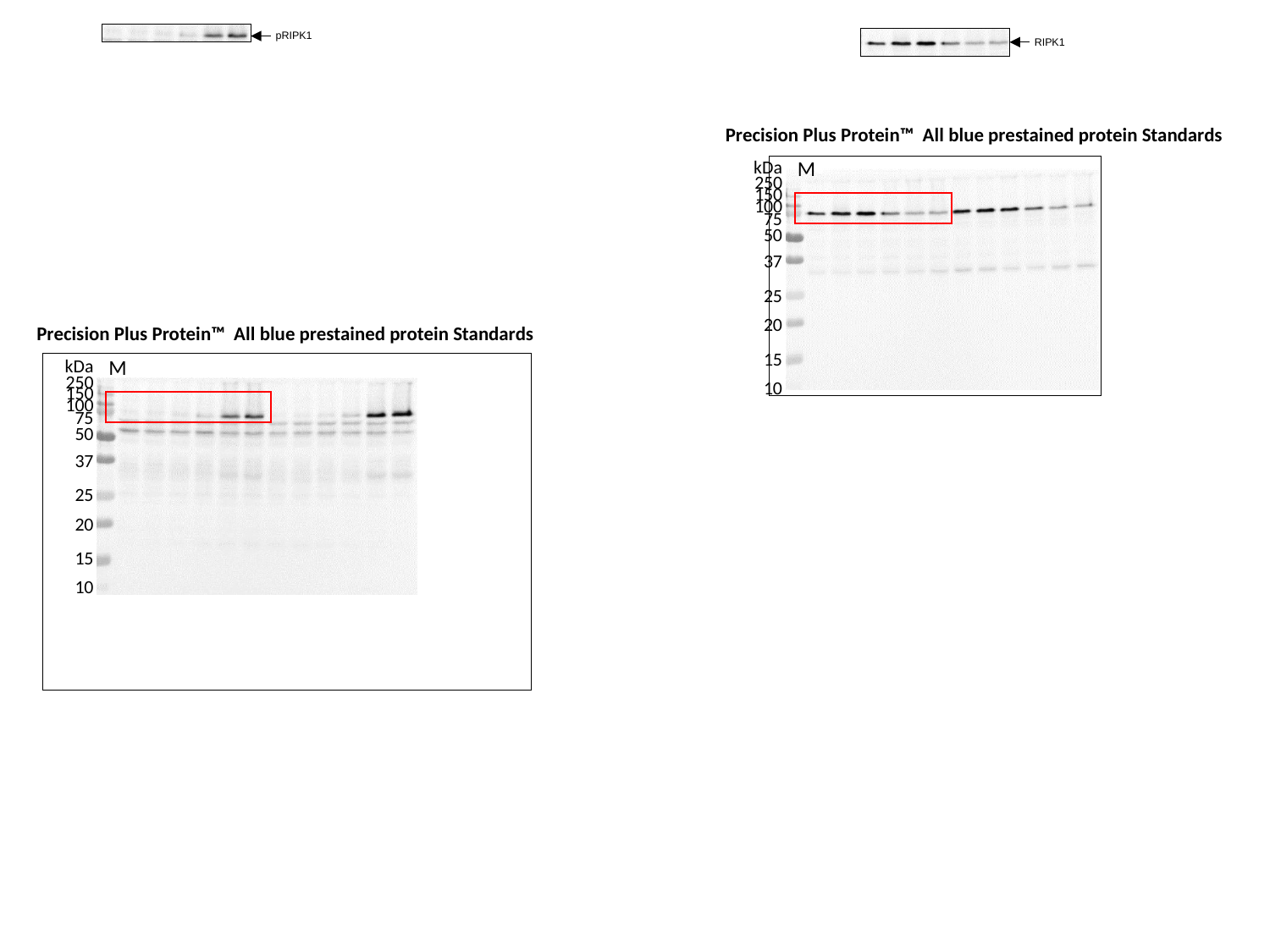

pRIPK1
RIPK1
Precision Plus Protein™ All blue prestained protein Standards
M
kDa
250
150
100
75
50
37
25
20
Precision Plus Protein™ All blue prestained protein Standards
15
M
kDa
250
10
150
100
75
50
37
25
20
15
10

## Slide 3
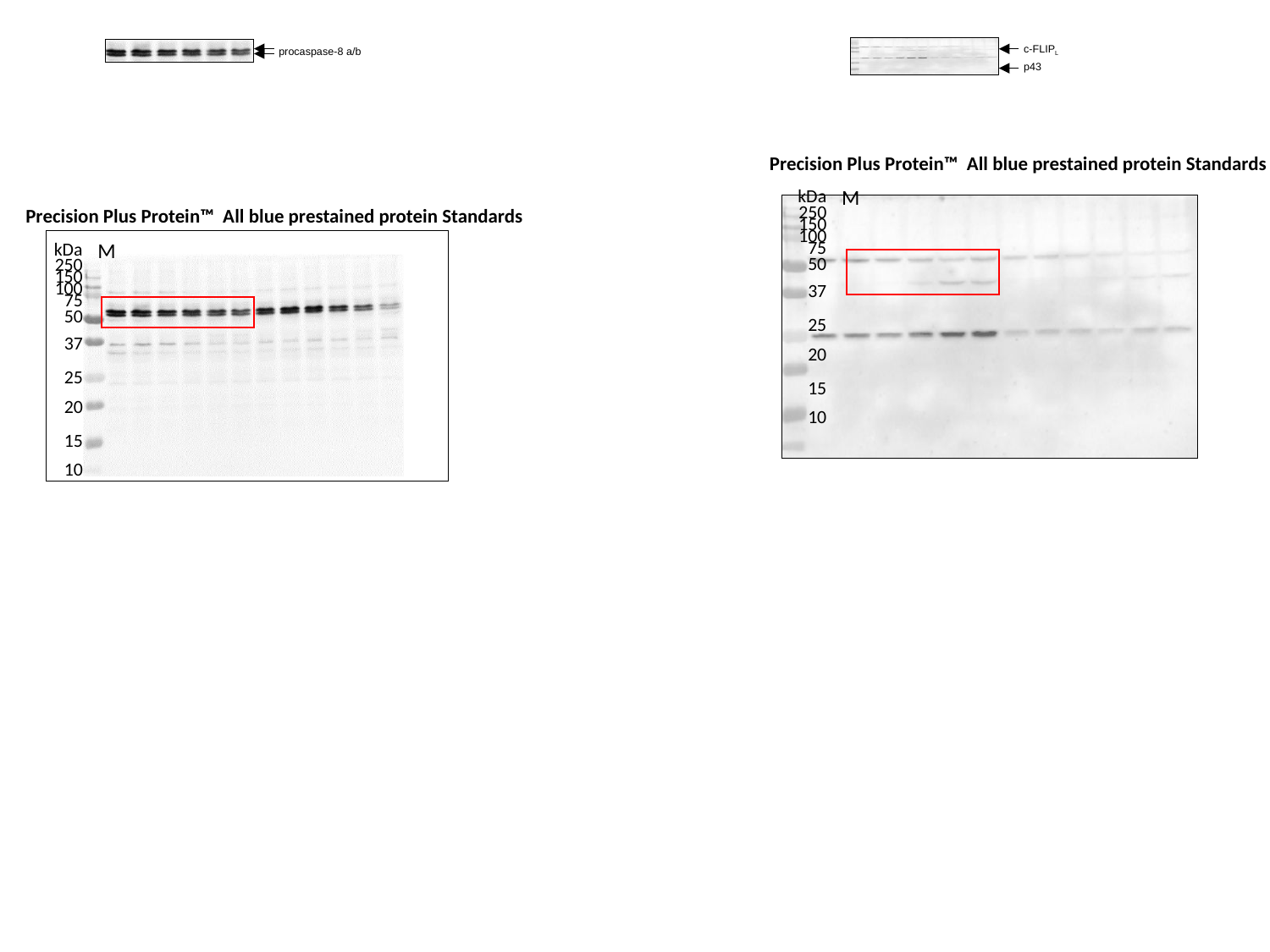

c-FLIPL
procaspase-8 a/b
p43
Precision Plus Protein™ All blue prestained protein Standards
M
kDa
250
Precision Plus Protein™ All blue prestained protein Standards
150
100
75
M
kDa
50
250
150
100
37
75
50
25
37
20
25
15
20
10
15
10

## Slide 4
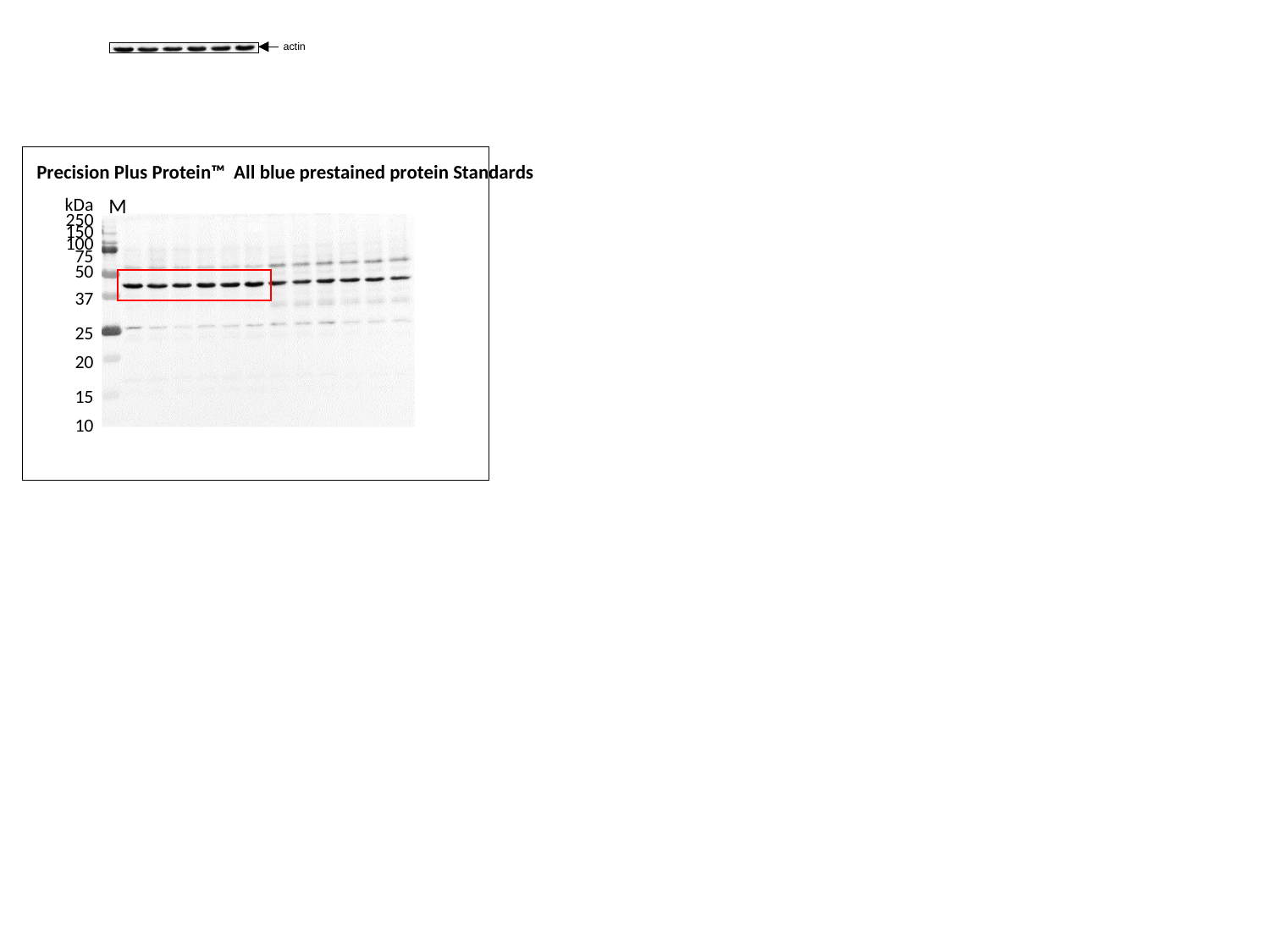

actin
Precision Plus Protein™ All blue prestained protein Standards
M
kDa
250
150
100
75
50
37
25
20
15
10
