# Supplementary material for: Oligomerised RIPK1 is the main core component of the CD95 necrosome
Source: EMBO J. 2025 Apr 16;44(11):3231–65. doi: 10.1038/s44318-025-00433-0 (PMC12130296; doi:10.1038/s44318-025-00433-0)
Supplement: Supplementary file 5 — Source data Fig. 1 [file 44318_2025_433_MOESM5_ESM.zip › figure1C.pptx]

## Slide 1
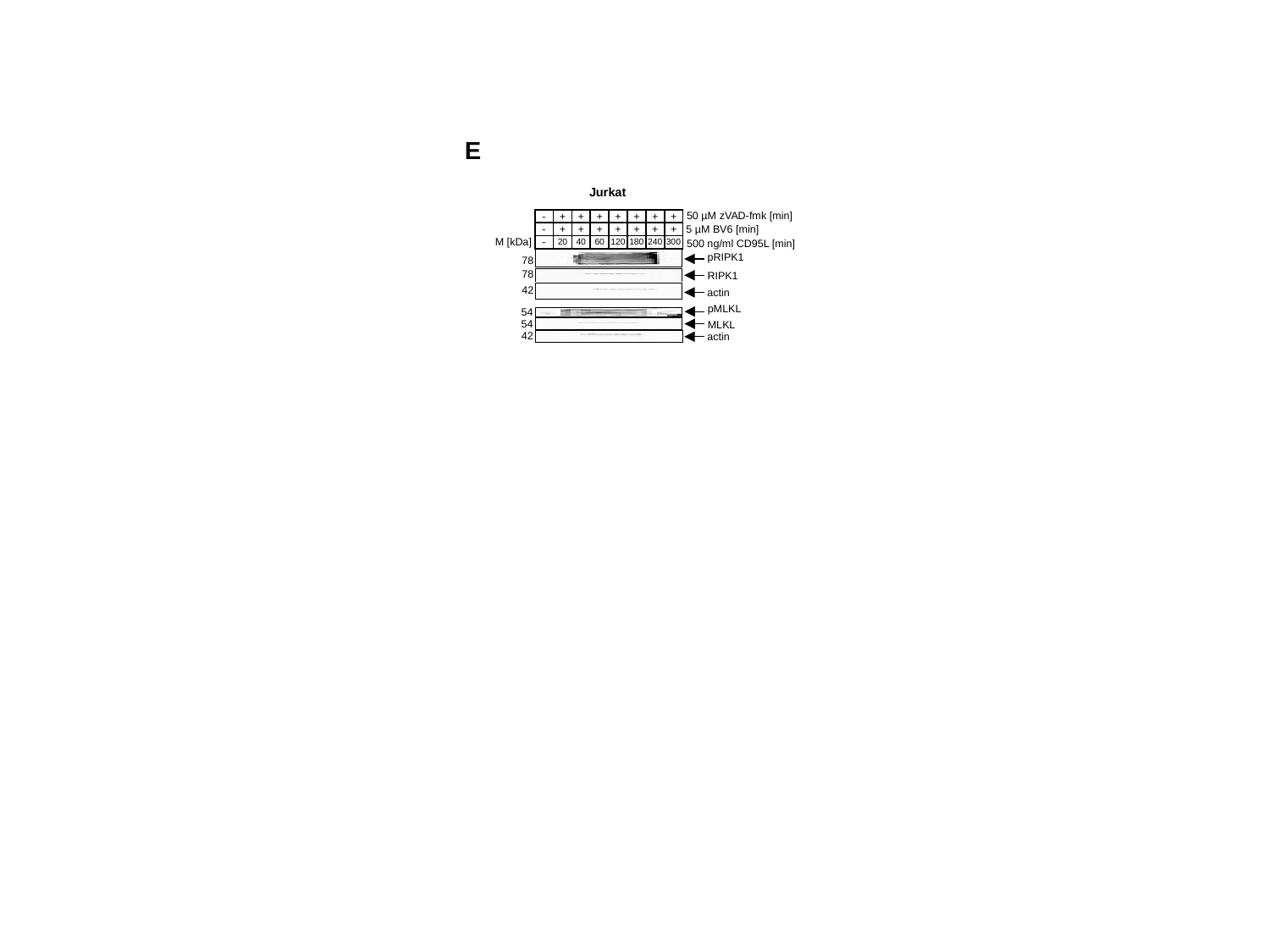

E
Jurkat
50 µM zVAD-fmk [min]
| - | + | + | + | + | + | + | + |
| --- | --- | --- | --- | --- | --- | --- | --- |
| - | + | + | + | + | + | + | + |
| - | 20 | 40 | 60 | 120 | 180 | 240 | 300 |
5 µM BV6 [min]
M [kDa]
500 ng/ml CD95L [min]
pRIPK1
78
78
RIPK1
42
actin
pMLKL
54
54
MLKL
42
actin

## Slide 2
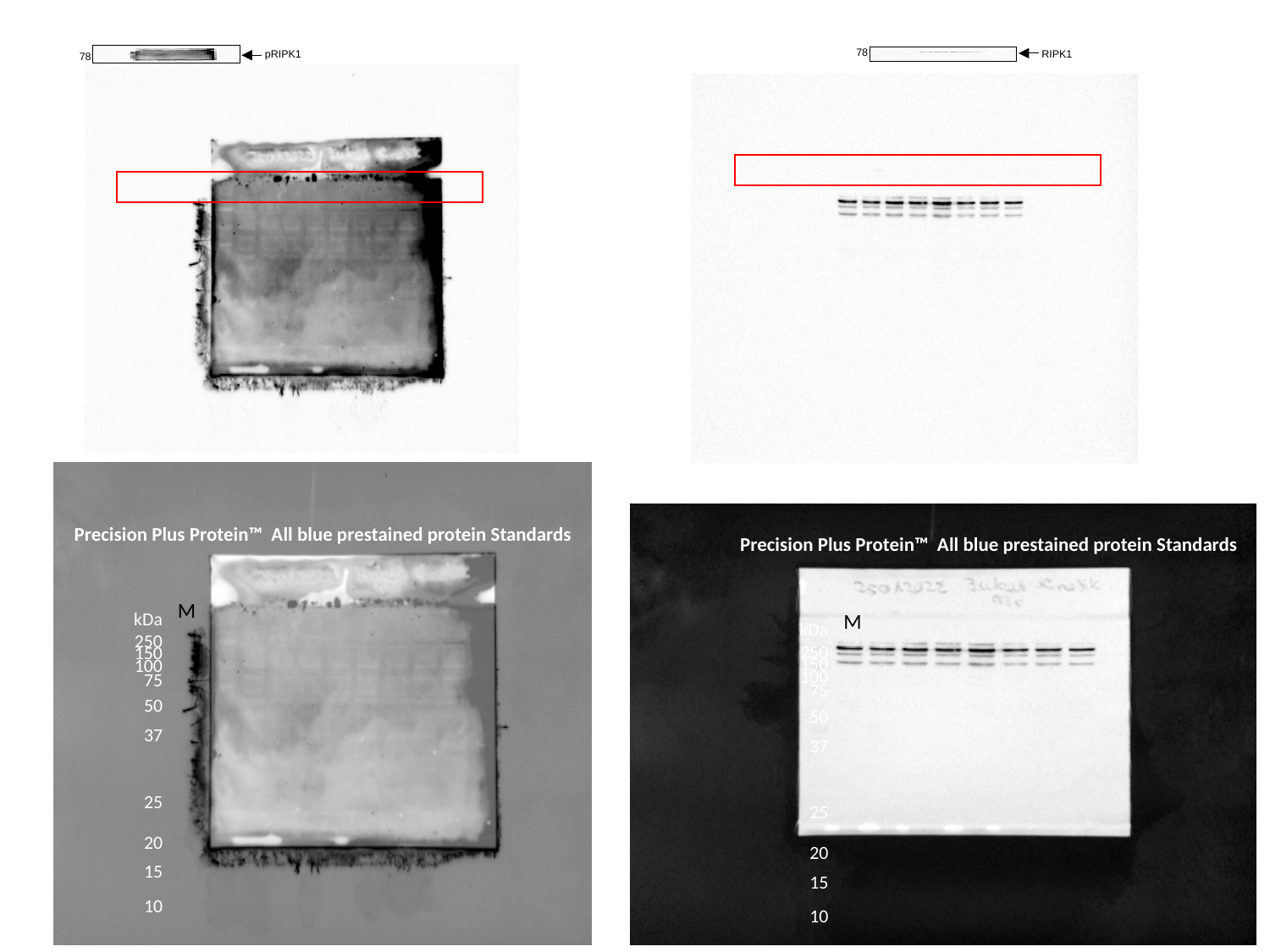

78
pRIPK1
RIPK1
78
Precision Plus Protein™ All blue prestained protein Standards
Precision Plus Protein™ All blue prestained protein Standards
M
kDa
M
kDa
250
250
150
150
100
100
75
75
50
50
37
37
25
25
20
20
15
15
10
10

## Slide 3
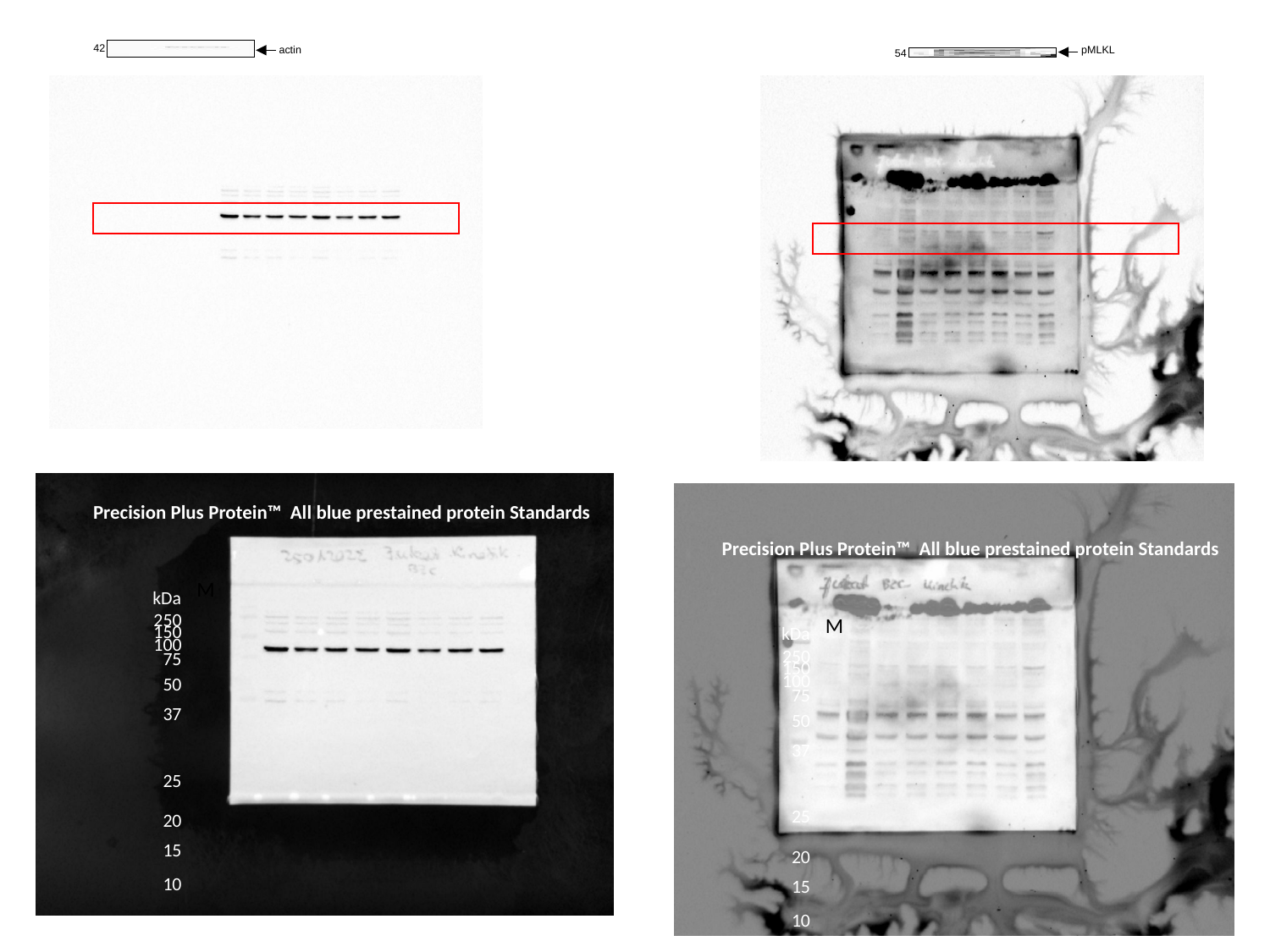

42
pMLKL
actin
54
Precision Plus Protein™ All blue prestained protein Standards
Precision Plus Protein™ All blue prestained protein Standards
M
kDa
250
M
150
kDa
100
250
75
150
100
50
75
37
50
37
25
25
20
15
20
10
15
10

## Slide 4
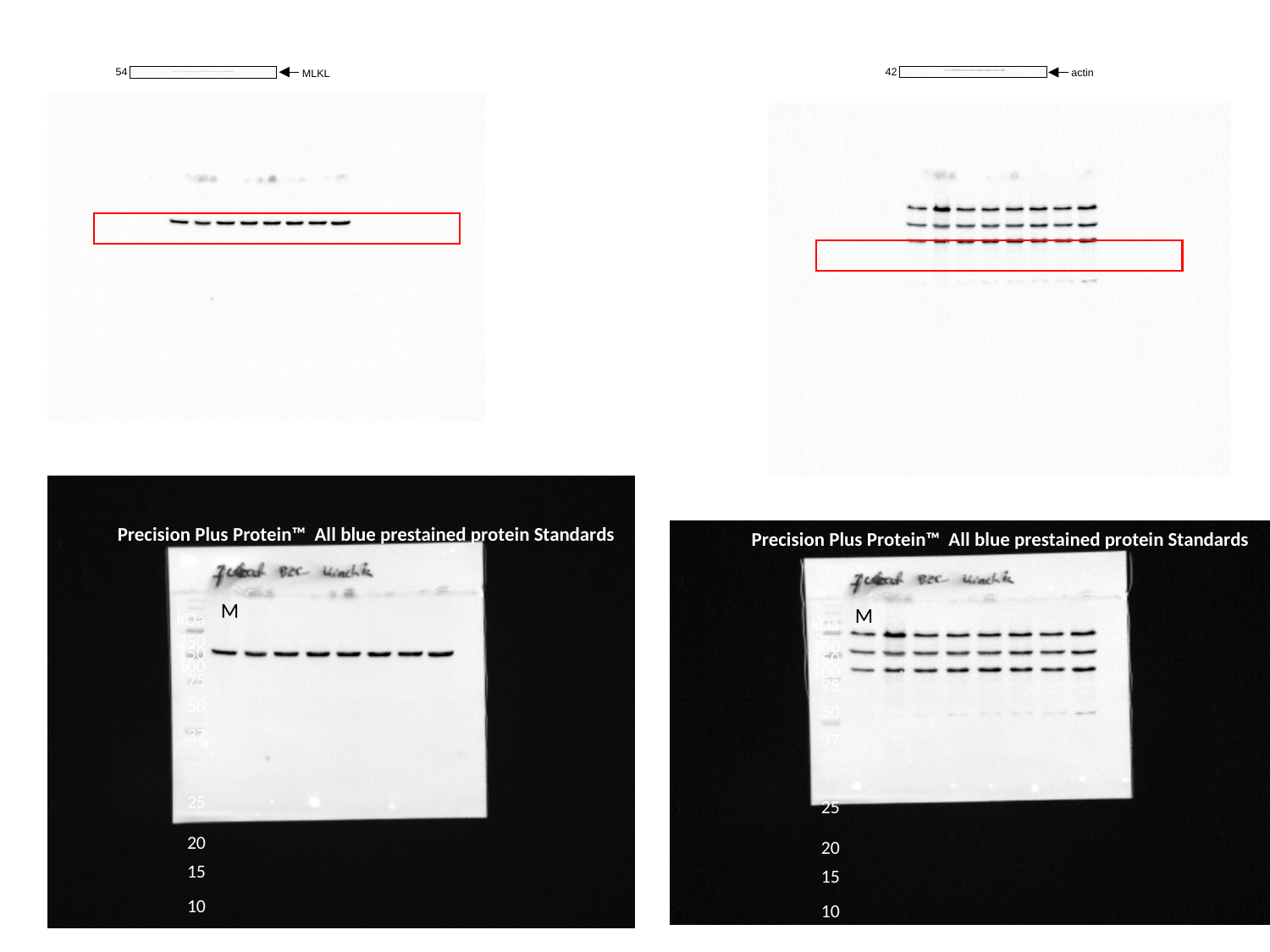

42
54
actin
MLKL
Precision Plus Protein™ All blue prestained protein Standards
Precision Plus Protein™ All blue prestained protein Standards
M
M
kDa
kDa
250
250
150
150
100
100
75
75
50
50
37
37
25
25
20
20
15
15
10
10
