# Supplementary figures and images for: Oligomerised RIPK1 is the main core component of the CD95 necrosome
Source: EMBO J. 2025 Apr 16;44(11):3231–65. doi: 10.1038/s44318-025-00433-0 (PMC12130296; doi:10.1038/s44318-025-00433-0)

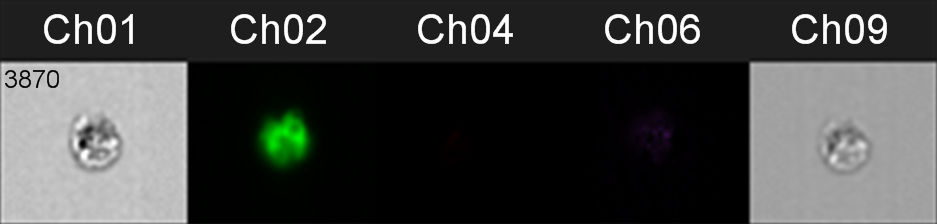

Supplement: Supplementary file 5 — Source data Fig. 1 [file 44318_2025_433_MOESM5_ESM.zip › figure-1-H-I/ht29-an1.png]

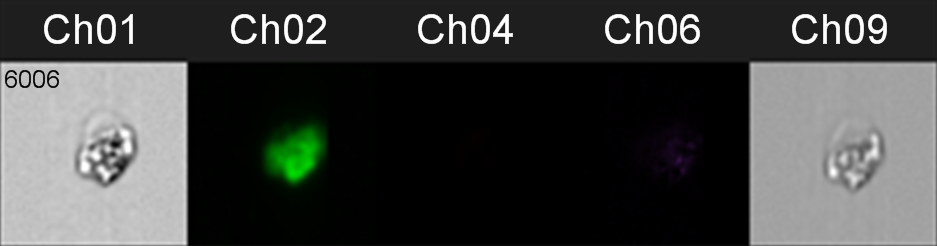

Supplement: Supplementary file 5 — Source data Fig. 1 [file 44318_2025_433_MOESM5_ESM.zip › figure-1-H-I/ht29-an2.png]

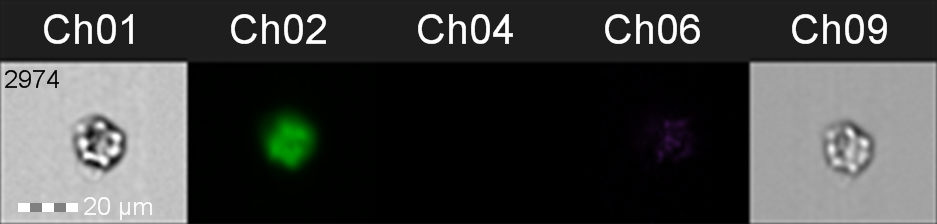

Supplement: Supplementary file 5 — Source data Fig. 1 [file 44318_2025_433_MOESM5_ESM.zip › figure-1-H-I/ht29-an3.png]

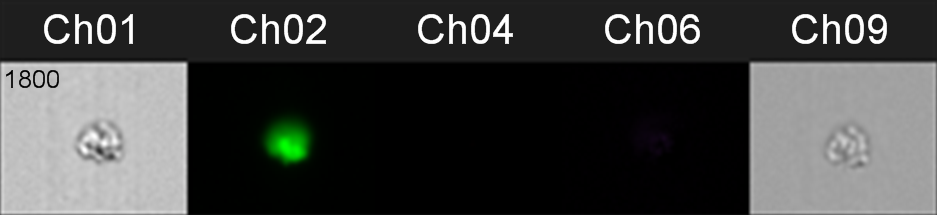

Supplement: Supplementary file 5 — Source data Fig. 1 [file 44318_2025_433_MOESM5_ESM.zip › figure-1-H-I/ht29-an4.png]

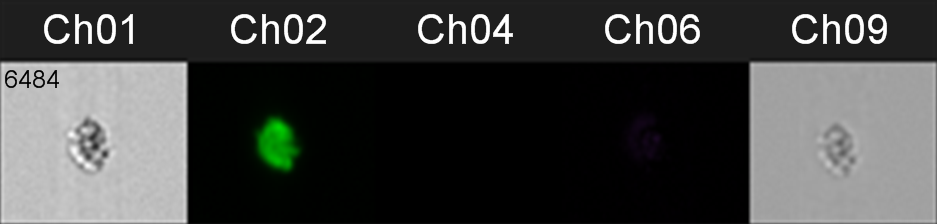

Supplement: Supplementary file 5 — Source data Fig. 1 [file 44318_2025_433_MOESM5_ESM.zip › figure-1-H-I/ht29-an5.png]

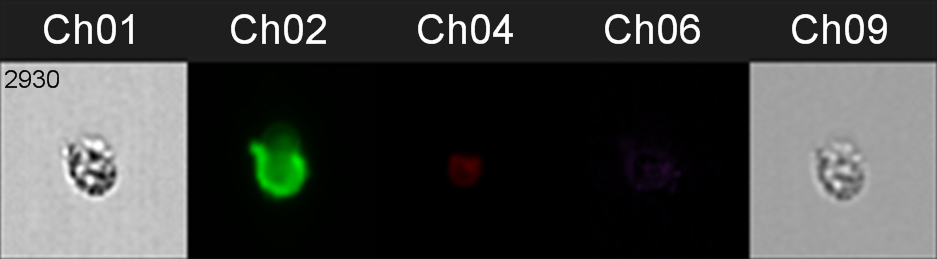

Supplement: Supplementary file 5 — Source data Fig. 1 [file 44318_2025_433_MOESM5_ESM.zip › figure-1-H-I/ht29-dp1.png]

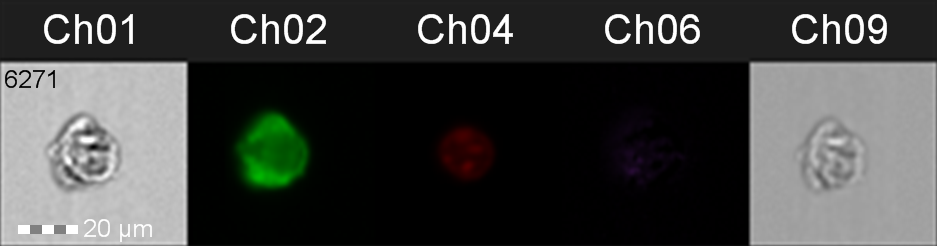

Supplement: Supplementary file 5 — Source data Fig. 1 [file 44318_2025_433_MOESM5_ESM.zip › figure-1-H-I/ht29-dp2.png]

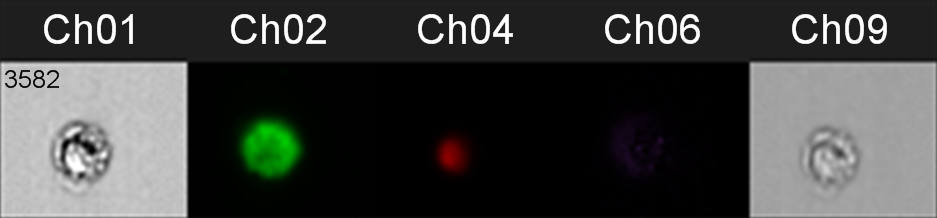

Supplement: Supplementary file 5 — Source data Fig. 1 [file 44318_2025_433_MOESM5_ESM.zip › figure-1-H-I/ht29-dp3.png]

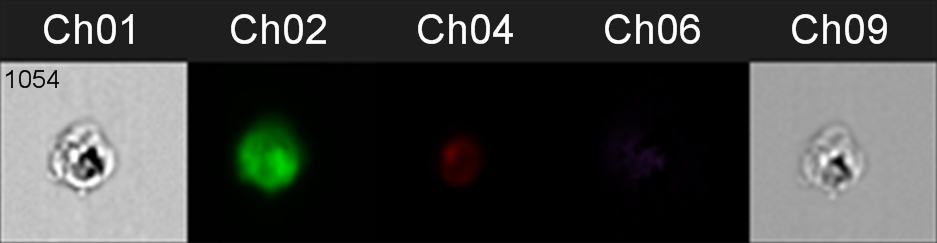

Supplement: Supplementary file 5 — Source data Fig. 1 [file 44318_2025_433_MOESM5_ESM.zip › figure-1-H-I/ht29-dp4.png]

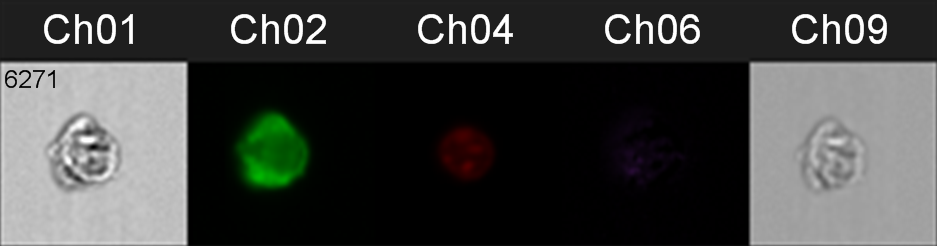

Supplement: Supplementary file 5 — Source data Fig. 1 [file 44318_2025_433_MOESM5_ESM.zip › figure-1-H-I/ht29-dp5.png]

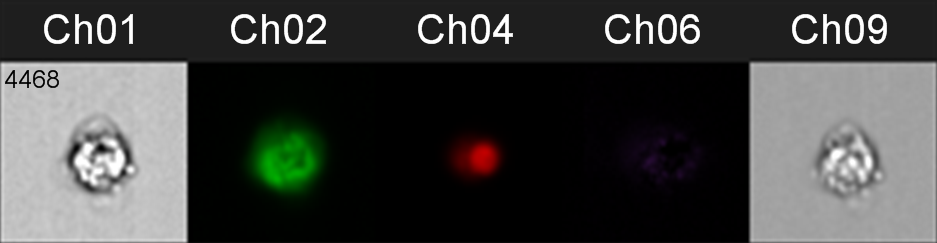

Supplement: Supplementary file 5 — Source data Fig. 1 [file 44318_2025_433_MOESM5_ESM.zip › figure-1-H-I/ht29-dp6.png]

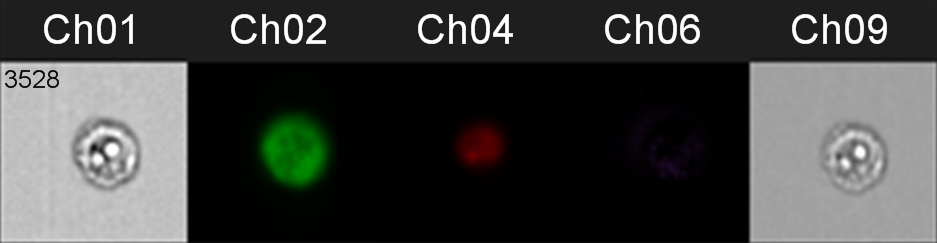

Supplement: Supplementary file 5 — Source data Fig. 1 [file 44318_2025_433_MOESM5_ESM.zip › figure-1-H-I/ht29-dp7.png]

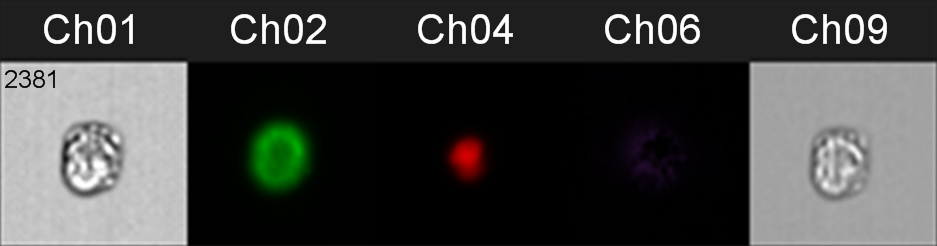

Supplement: Supplementary file 5 — Source data Fig. 1 [file 44318_2025_433_MOESM5_ESM.zip › figure-1-H-I/ht29-dp8.png]

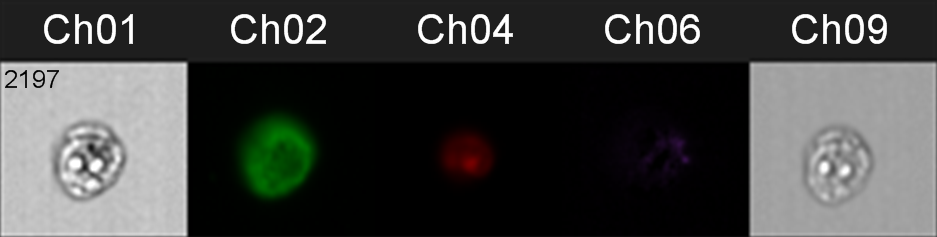

Supplement: Supplementary file 5 — Source data Fig. 1 [file 44318_2025_433_MOESM5_ESM.zip › figure-1-H-I/ht29-dp9.png]

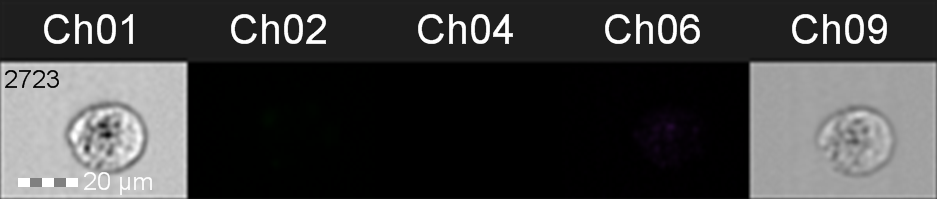

Supplement: Supplementary file 5 — Source data Fig. 1 [file 44318_2025_433_MOESM5_ESM.zip › figure-1-H-I/ht29-n1.png]

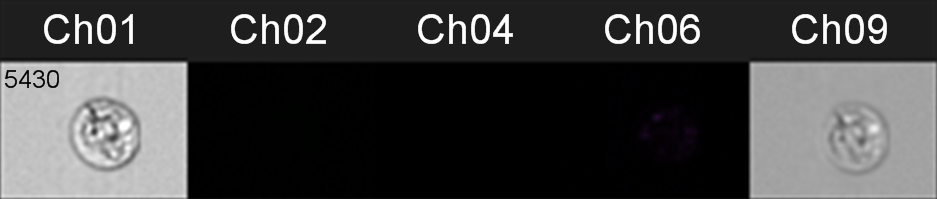

Supplement: Supplementary file 5 — Source data Fig. 1 [file 44318_2025_433_MOESM5_ESM.zip › figure-1-H-I/ht29-n2.png]

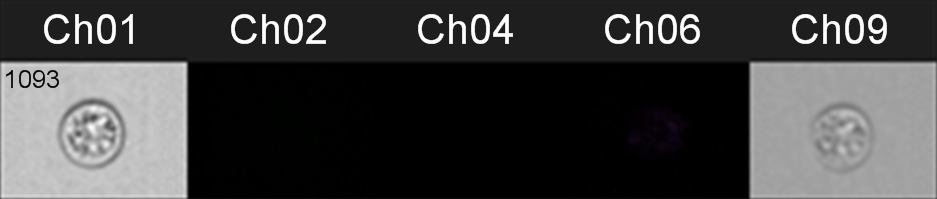

Supplement: Supplementary file 5 — Source data Fig. 1 [file 44318_2025_433_MOESM5_ESM.zip › figure-1-H-I/ht29-n3.png]

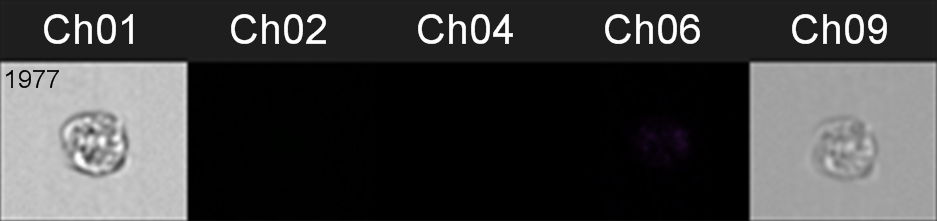

Supplement: Supplementary file 5 — Source data Fig. 1 [file 44318_2025_433_MOESM5_ESM.zip › figure-1-H-I/ht29-n4.png]

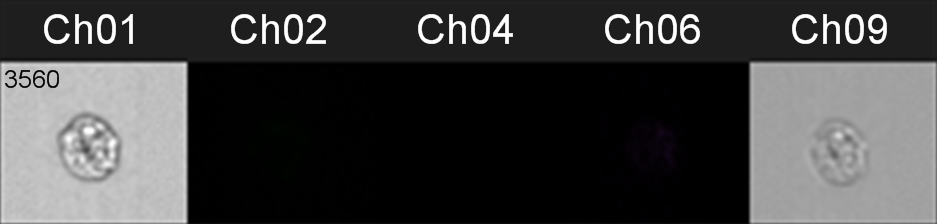

Supplement: Supplementary file 5 — Source data Fig. 1 [file 44318_2025_433_MOESM5_ESM.zip › figure-1-H-I/ht29-n5.png]

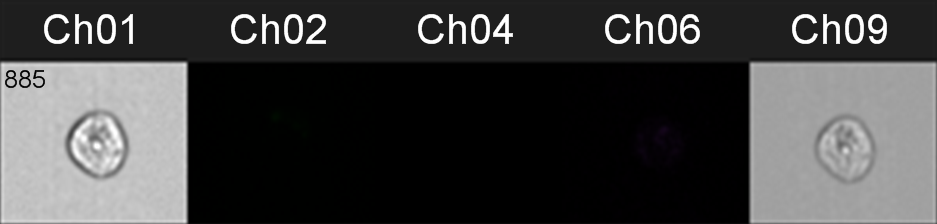

Supplement: Supplementary file 5 — Source data Fig. 1 [file 44318_2025_433_MOESM5_ESM.zip › figure-1-H-I/ht29-n6.png]

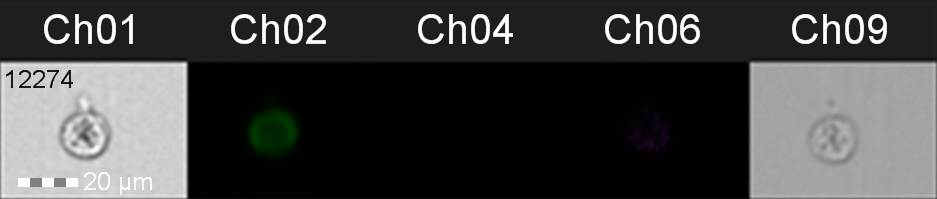

Supplement: Supplementary file 5 — Source data Fig. 1 [file 44318_2025_433_MOESM5_ESM.zip › figure-1-H-I/jurk-an1.png]

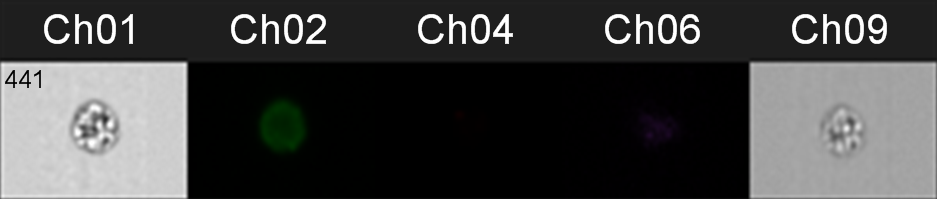

Supplement: Supplementary file 5 — Source data Fig. 1 [file 44318_2025_433_MOESM5_ESM.zip › figure-1-H-I/jurk-an2.png]

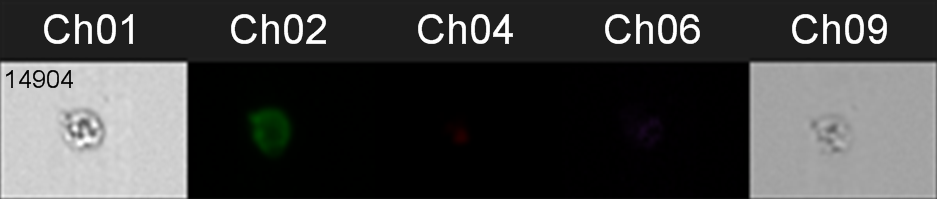

Supplement: Supplementary file 5 — Source data Fig. 1 [file 44318_2025_433_MOESM5_ESM.zip › figure-1-H-I/jurk-an3.png]

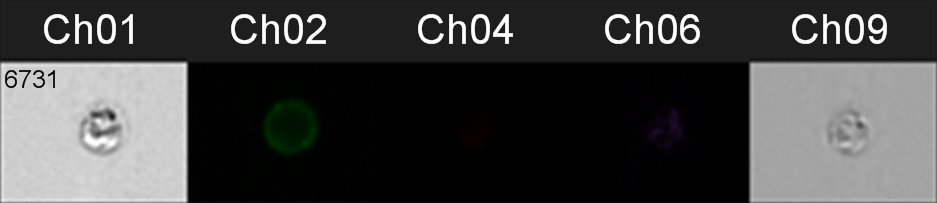

Supplement: Supplementary file 5 — Source data Fig. 1 [file 44318_2025_433_MOESM5_ESM.zip › figure-1-H-I/jurk-an4.png]

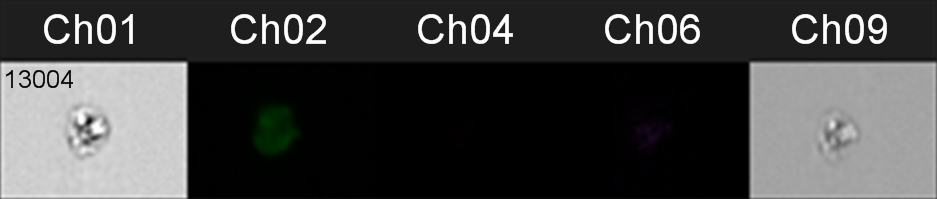

Supplement: Supplementary file 5 — Source data Fig. 1 [file 44318_2025_433_MOESM5_ESM.zip › figure-1-H-I/jurk-an5.png]

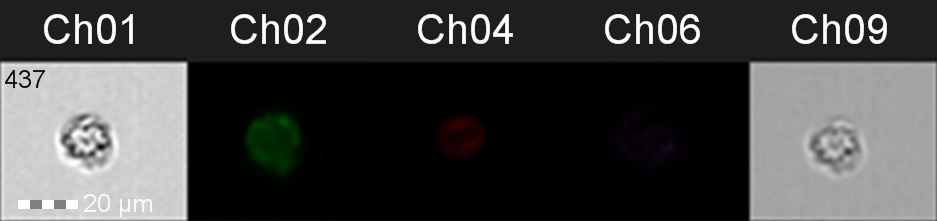

Supplement: Supplementary file 5 — Source data Fig. 1 [file 44318_2025_433_MOESM5_ESM.zip › figure-1-H-I/jurk-dp1.png]

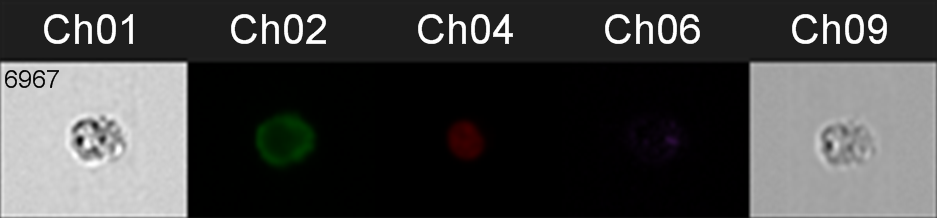

Supplement: Supplementary file 5 — Source data Fig. 1 [file 44318_2025_433_MOESM5_ESM.zip › figure-1-H-I/jurk-dp2.png]

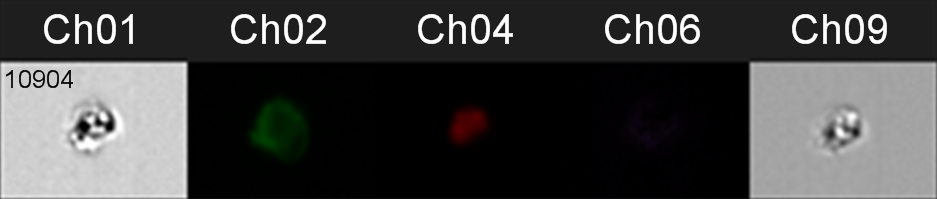

Supplement: Supplementary file 5 — Source data Fig. 1 [file 44318_2025_433_MOESM5_ESM.zip › figure-1-H-I/jurk-dp3.png]

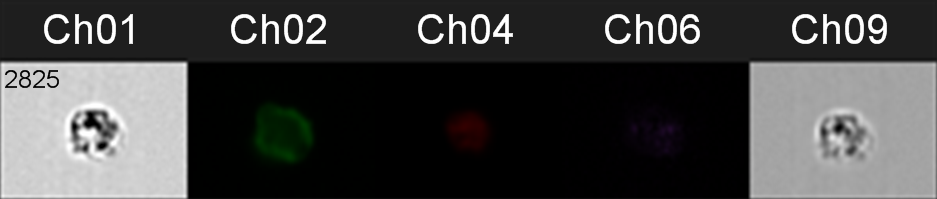

Supplement: Supplementary file 5 — Source data Fig. 1 [file 44318_2025_433_MOESM5_ESM.zip › figure-1-H-I/jurk-dp4.png]

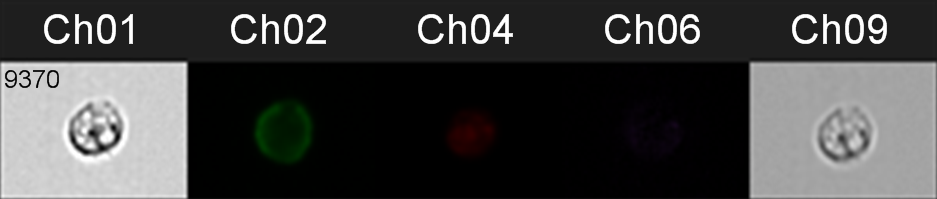

Supplement: Supplementary file 5 — Source data Fig. 1 [file 44318_2025_433_MOESM5_ESM.zip › figure-1-H-I/jurk-dp5.png]

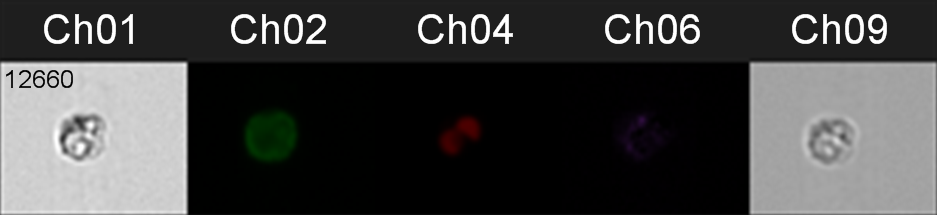

Supplement: Supplementary file 5 — Source data Fig. 1 [file 44318_2025_433_MOESM5_ESM.zip › figure-1-H-I/jurk-dp6.png]

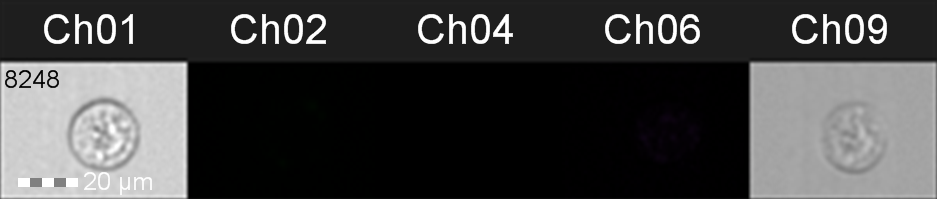

Supplement: Supplementary file 5 — Source data Fig. 1 [file 44318_2025_433_MOESM5_ESM.zip › figure-1-H-I/jurk-n1.png]

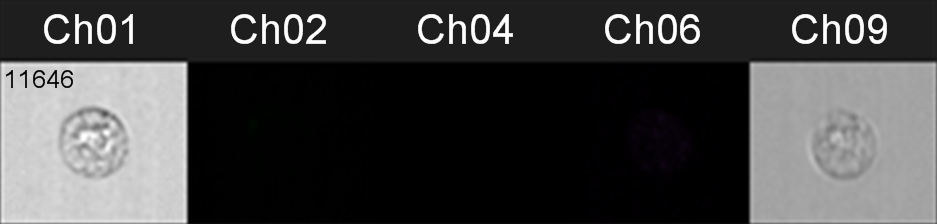

Supplement: Supplementary file 5 — Source data Fig. 1 [file 44318_2025_433_MOESM5_ESM.zip › figure-1-H-I/jurk-n2.png]

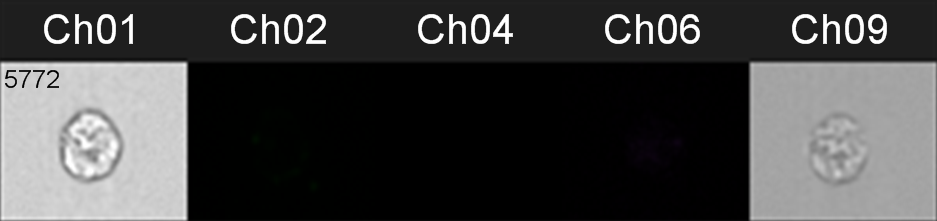

Supplement: Supplementary file 5 — Source data Fig. 1 [file 44318_2025_433_MOESM5_ESM.zip › figure-1-H-I/jurk-n3.png]

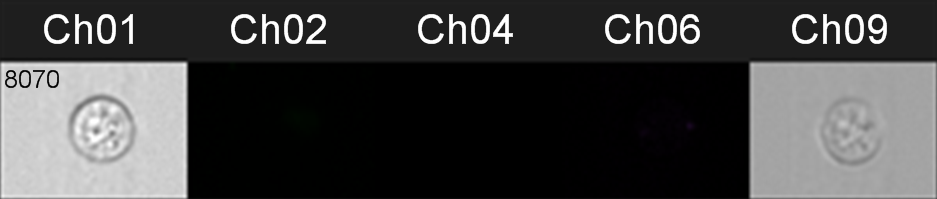

Supplement: Supplementary file 5 — Source data Fig. 1 [file 44318_2025_433_MOESM5_ESM.zip › figure-1-H-I/jurk-n4.png]

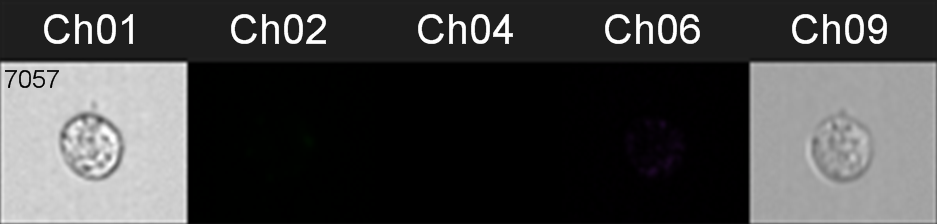

Supplement: Supplementary file 5 — Source data Fig. 1 [file 44318_2025_433_MOESM5_ESM.zip › figure-1-H-I/jurk-n5.png]

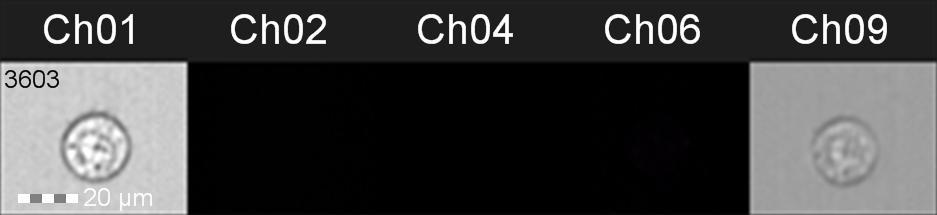

Supplement: Supplementary file 11 — Figure EV2 Source Data [file 44318_2025_433_MOESM11_ESM.zip › micr.image/jurkRIPK1-n1.png]

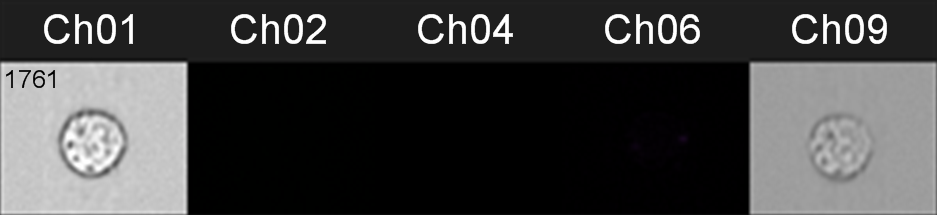

Supplement: Supplementary file 11 — Figure EV2 Source Data [file 44318_2025_433_MOESM11_ESM.zip › micr.image/jurkRIPK1-n2.png]

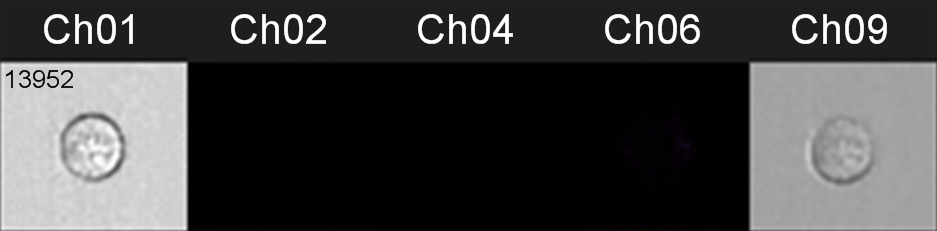

Supplement: Supplementary file 11 — Figure EV2 Source Data [file 44318_2025_433_MOESM11_ESM.zip › micr.image/jurkRIPK1-n3.png]

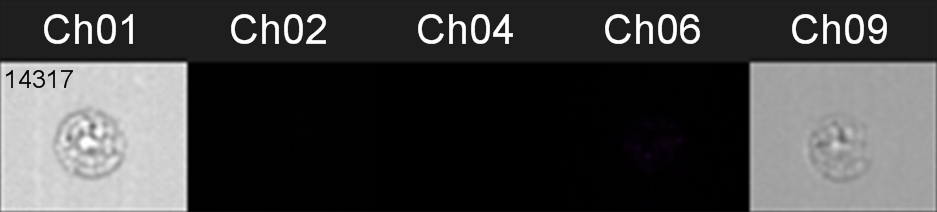

Supplement: Supplementary file 11 — Figure EV2 Source Data [file 44318_2025_433_MOESM11_ESM.zip › micr.image/jurkRIPK1-n4.png]

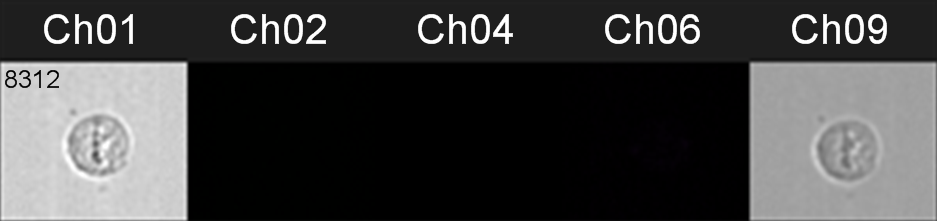

Supplement: Supplementary file 11 — Figure EV2 Source Data [file 44318_2025_433_MOESM11_ESM.zip › micr.image/jurkRIPK1-n5.png]
