# Supplementary material for: Oligomerised RIPK1 is the main core component of the CD95 necrosome
Source: EMBO J. 2025 Apr 16;44(11):3231–65. doi: 10.1038/s44318-025-00433-0 (PMC12130296; doi:10.1038/s44318-025-00433-0)
Supplement: Supplementary file 6 — Source data Fig. 2 [file 44318_2025_433_MOESM6_ESM.zip › figure2A.pptx]

## Slide 1
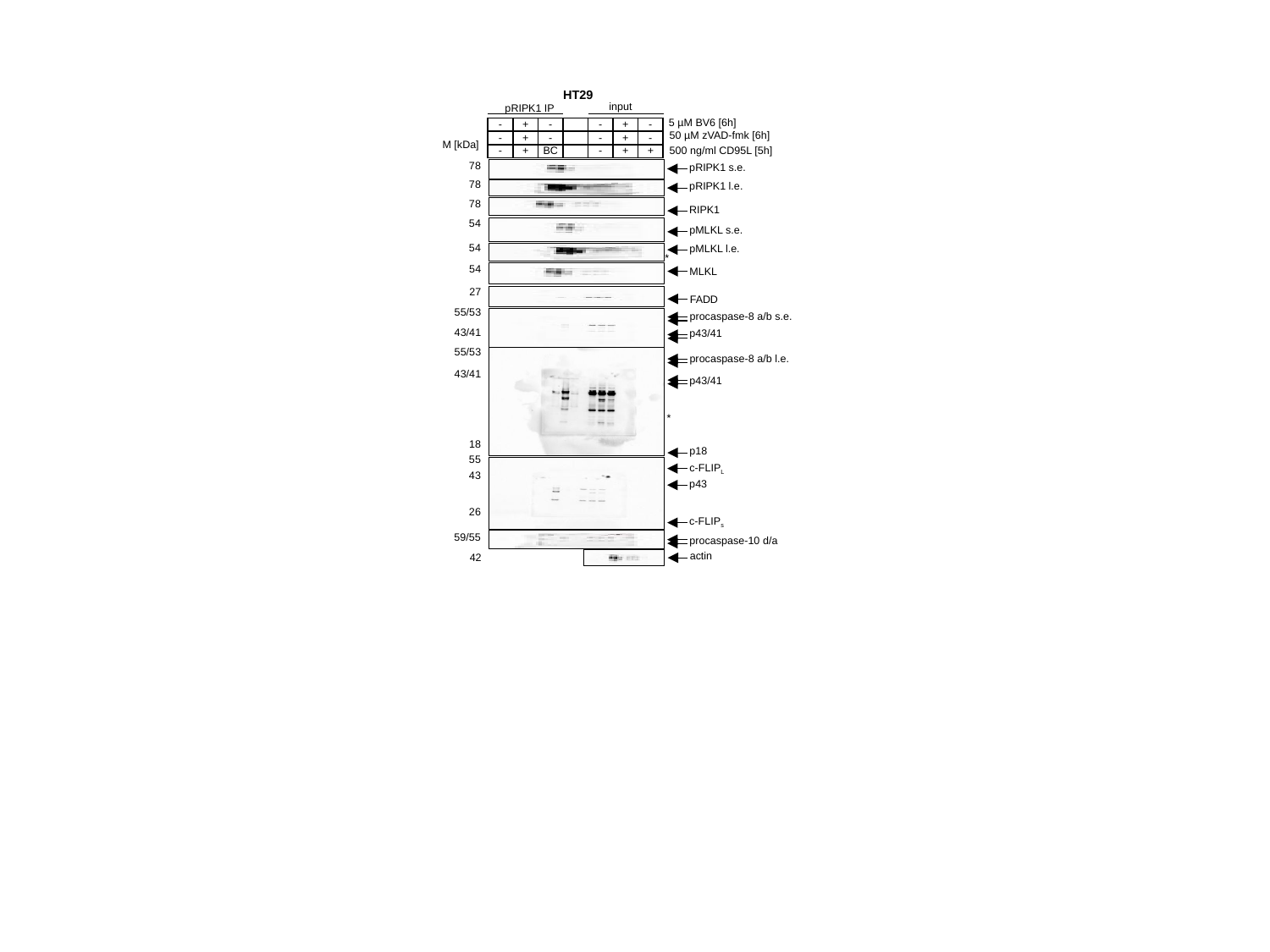

HT29
input
pRIPK1 IP
5 µM BV6 [6h]
| - | + | - | | - | + | - |
| --- | --- | --- | --- | --- | --- | --- |
| - | + | - | | - | + | - |
| - | + | BC | | - | + | + |
50 µM zVAD-fmk [6h]
M [kDa]
500 ng/ml CD95L [5h]
78
pRIPK1 s.e.
78
pRIPK1 l.e.
78
RIPK1
54
pMLKL s.e.
54
pMLKL l.e.
*
54
MLKL
27
FADD
55/53
procaspase-8 a/b s.e.
43/41
p43/41
55/53
procaspase-8 a/b l.e.
43/41
p43/41
*
18
p18
55
c-FLIPL
43
p43
26
c-FLIPs
59/55
procaspase-10 d/a
actin
42

## Slide 2
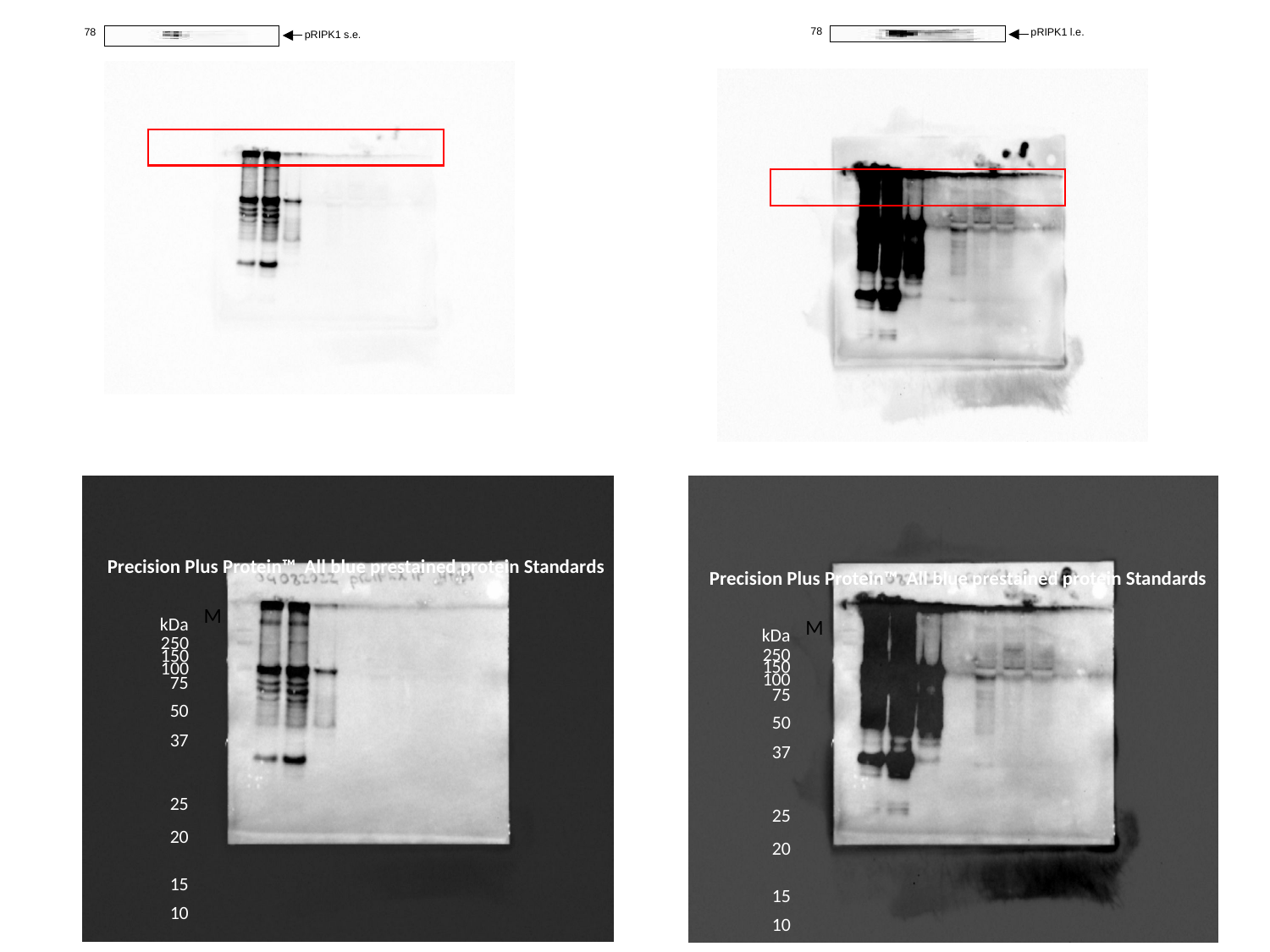

78
78
pRIPK1 l.e.
pRIPK1 s.e.
Precision Plus Protein™ All blue prestained protein Standards
Precision Plus Protein™ All blue prestained protein Standards
M
kDa
M
kDa
250
250
150
150
100
100
75
75
50
50
37
37
25
25
20
20
15
15
10
10

## Slide 3
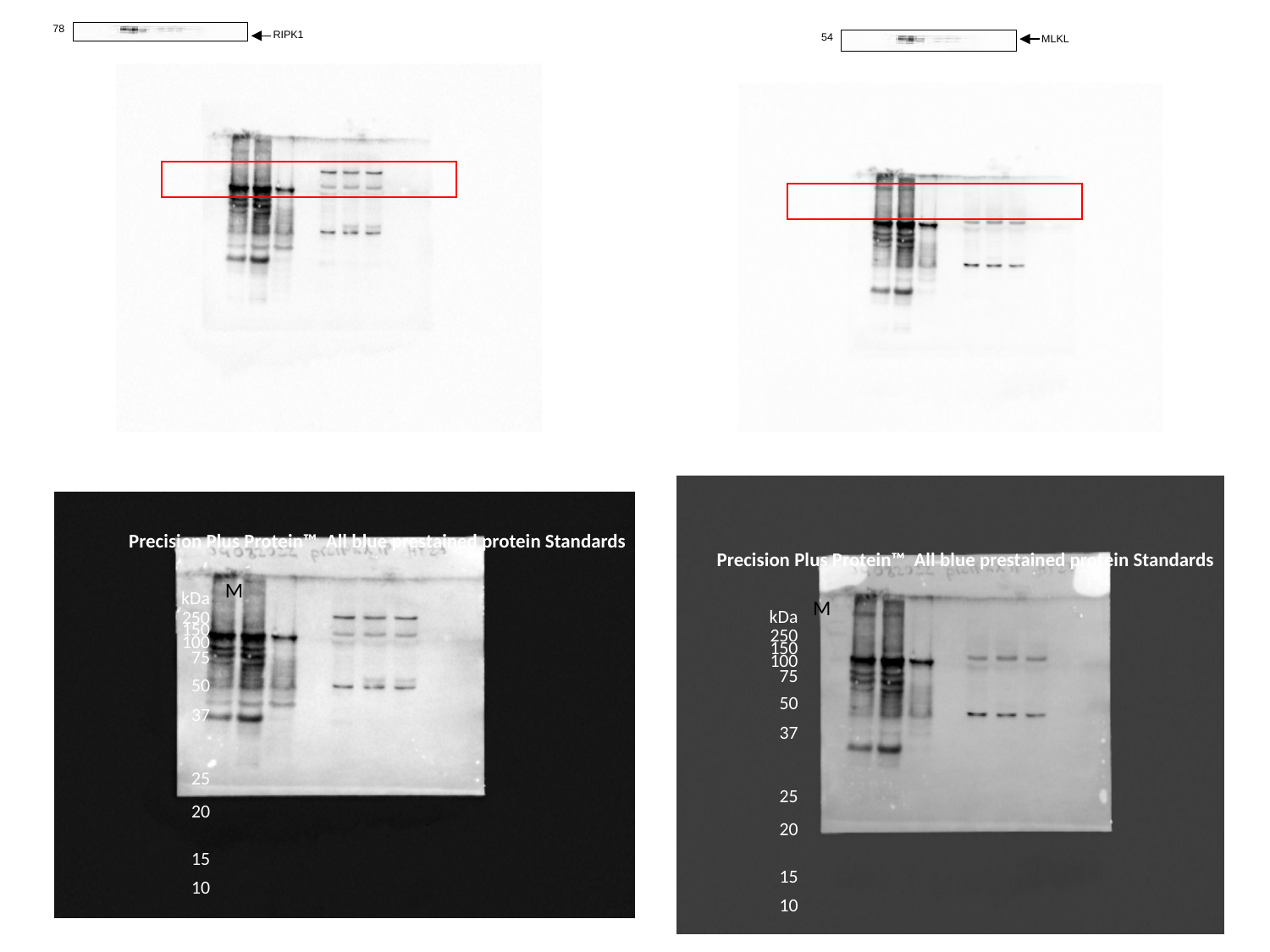

78
RIPK1
54
MLKL
Precision Plus Protein™ All blue prestained protein Standards
Precision Plus Protein™ All blue prestained protein Standards
M
kDa
M
kDa
250
150
250
100
150
75
100
75
50
50
37
37
25
25
20
20
15
15
10
10

## Slide 4
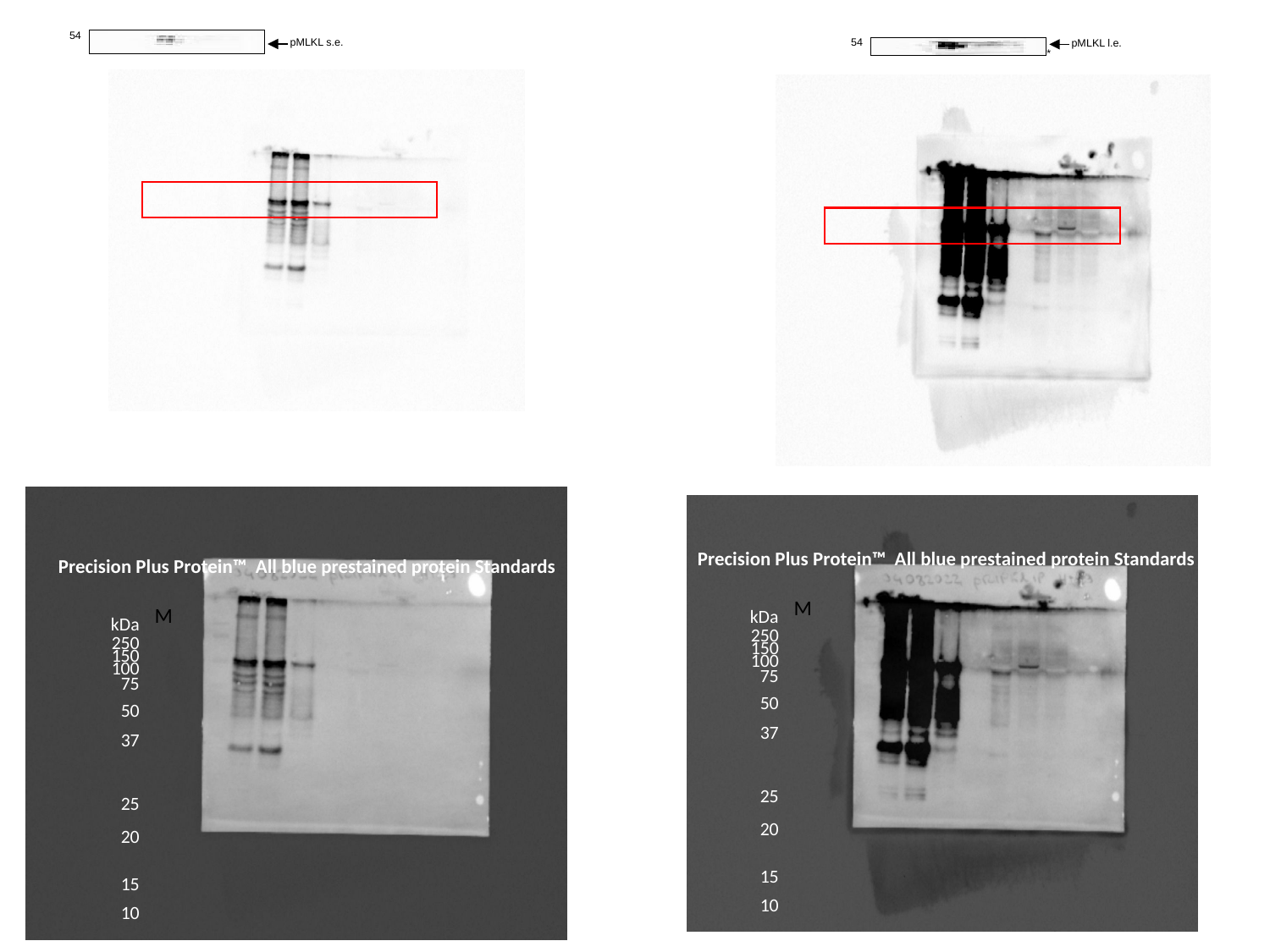

54
54
pMLKL s.e.
pMLKL l.e.
*
Precision Plus Protein™ All blue prestained protein Standards
Precision Plus Protein™ All blue prestained protein Standards
M
M
kDa
kDa
250
250
150
150
100
100
75
75
50
50
37
37
25
25
20
20
15
15
10
10

## Slide 5
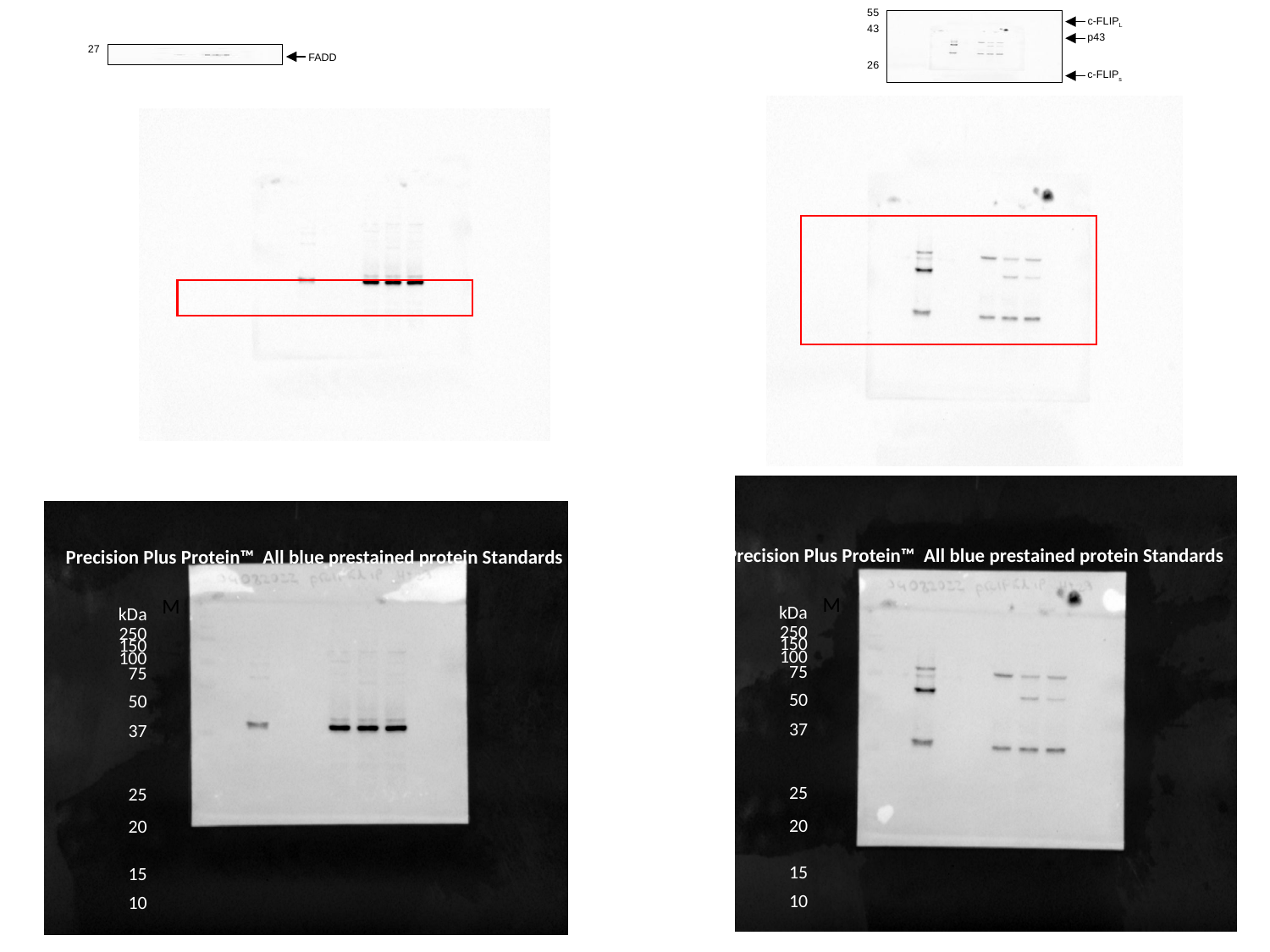

55
c-FLIPL
43
p43
27
FADD
26
c-FLIPs
Precision Plus Protein™ All blue prestained protein Standards
Precision Plus Protein™ All blue prestained protein Standards
M
M
kDa
kDa
250
250
150
150
100
100
75
75
50
50
37
37
25
25
20
20
15
15
10
10

## Slide 6
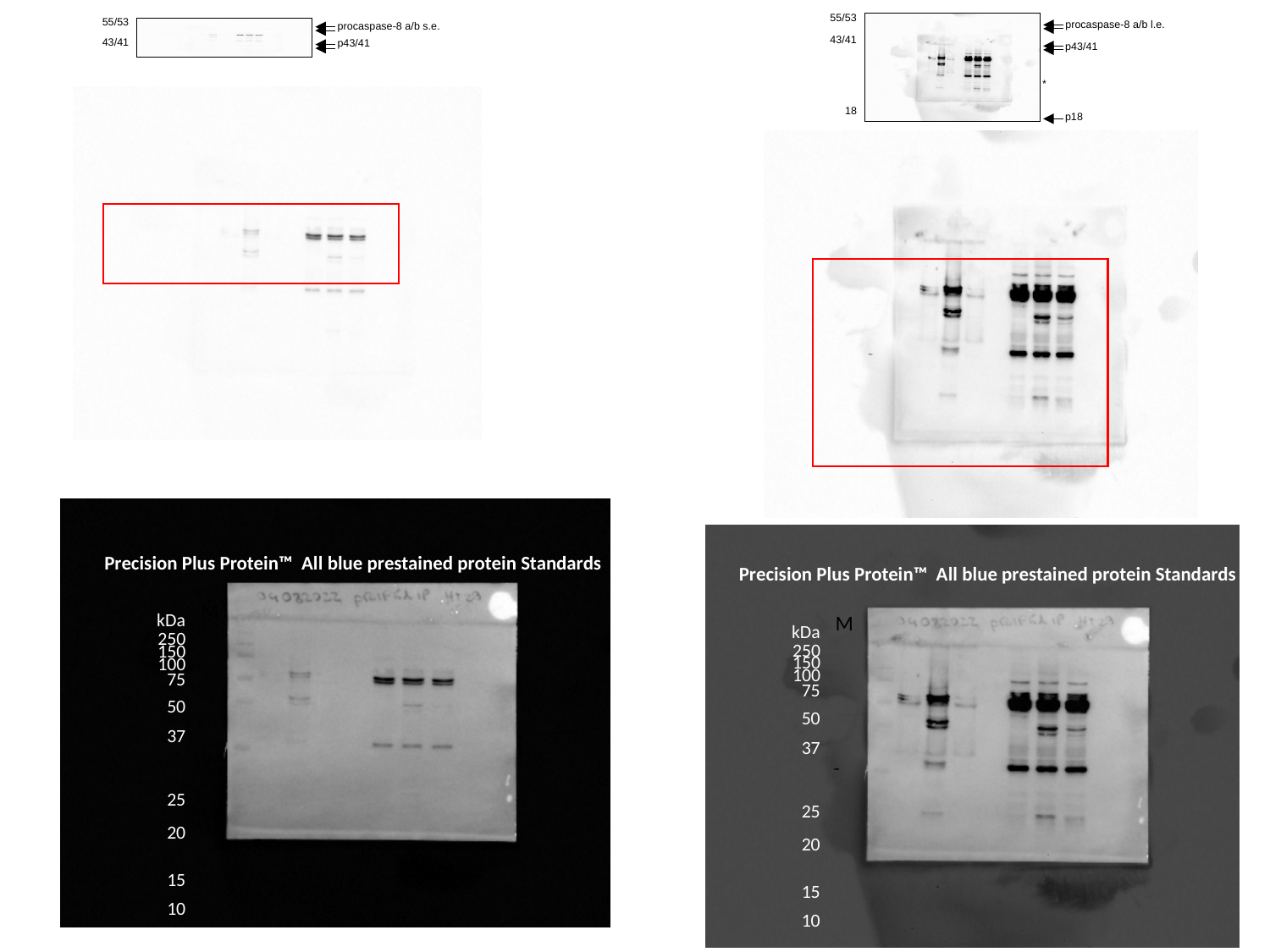

55/53
55/53
procaspase-8 a/b l.e.
procaspase-8 a/b s.e.
43/41
43/41
p43/41
p43/41
*
18
p18
Precision Plus Protein™ All blue prestained protein Standards
Precision Plus Protein™ All blue prestained protein Standards
M
kDa
M
kDa
250
250
150
150
100
100
75
75
50
50
37
37
25
25
20
20
15
15
10
10

## Slide 7
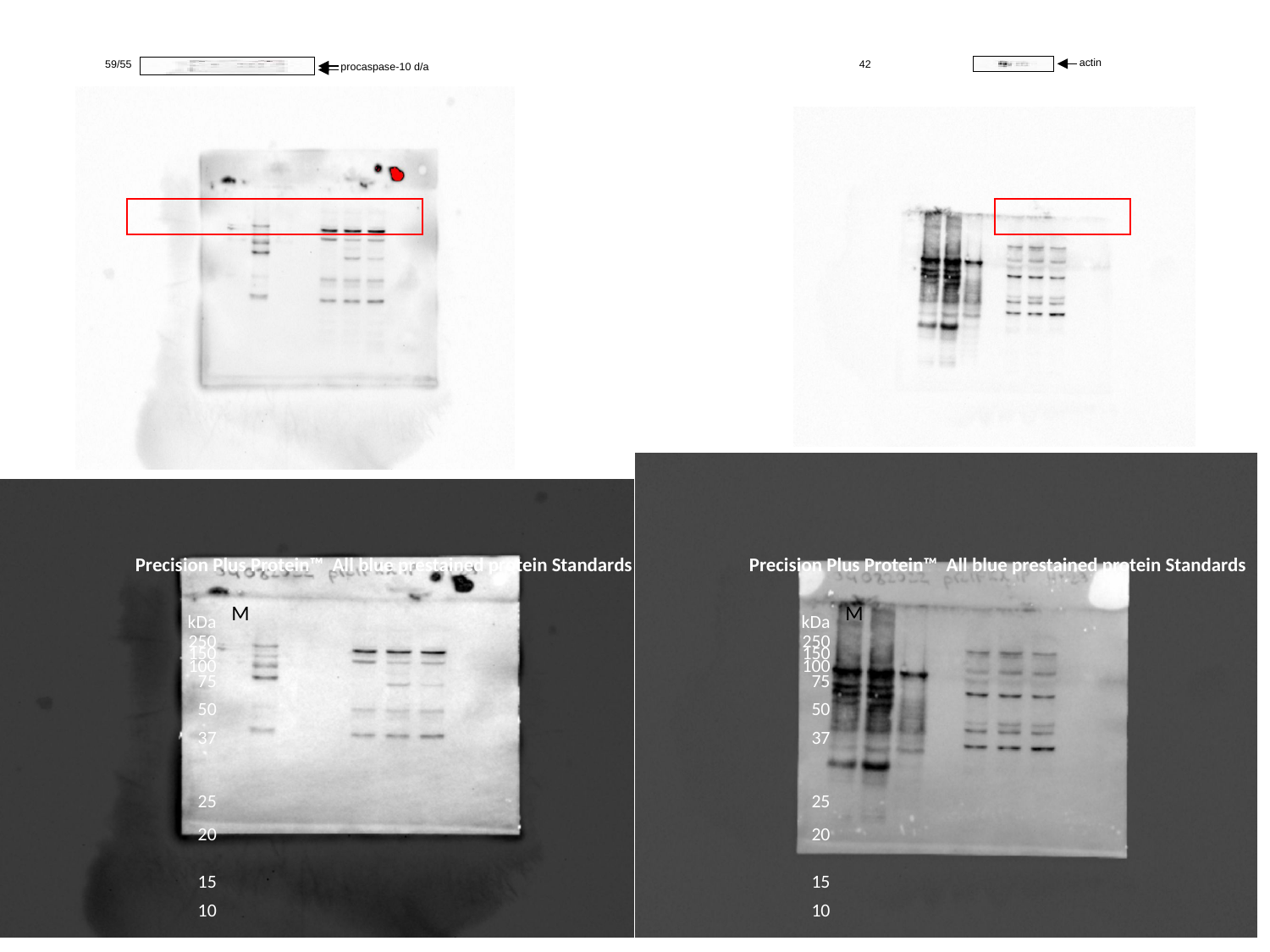

actin
42
59/55
procaspase-10 d/a
Precision Plus Protein™ All blue prestained protein Standards
Precision Plus Protein™ All blue prestained protein Standards
M
M
kDa
kDa
250
250
150
150
100
100
75
75
50
50
37
37
25
25
20
20
15
15
10
10
