# Supplementary material for: Oligomerised RIPK1 is the main core component of the CD95 necrosome
Source: EMBO J. 2025 Apr 16;44(11):3231–65. doi: 10.1038/s44318-025-00433-0 (PMC12130296; doi:10.1038/s44318-025-00433-0)
Supplement: Supplementary file 6 — Source data Fig. 2 [file 44318_2025_433_MOESM6_ESM.zip › figure2B.pptx]

## Slide 1
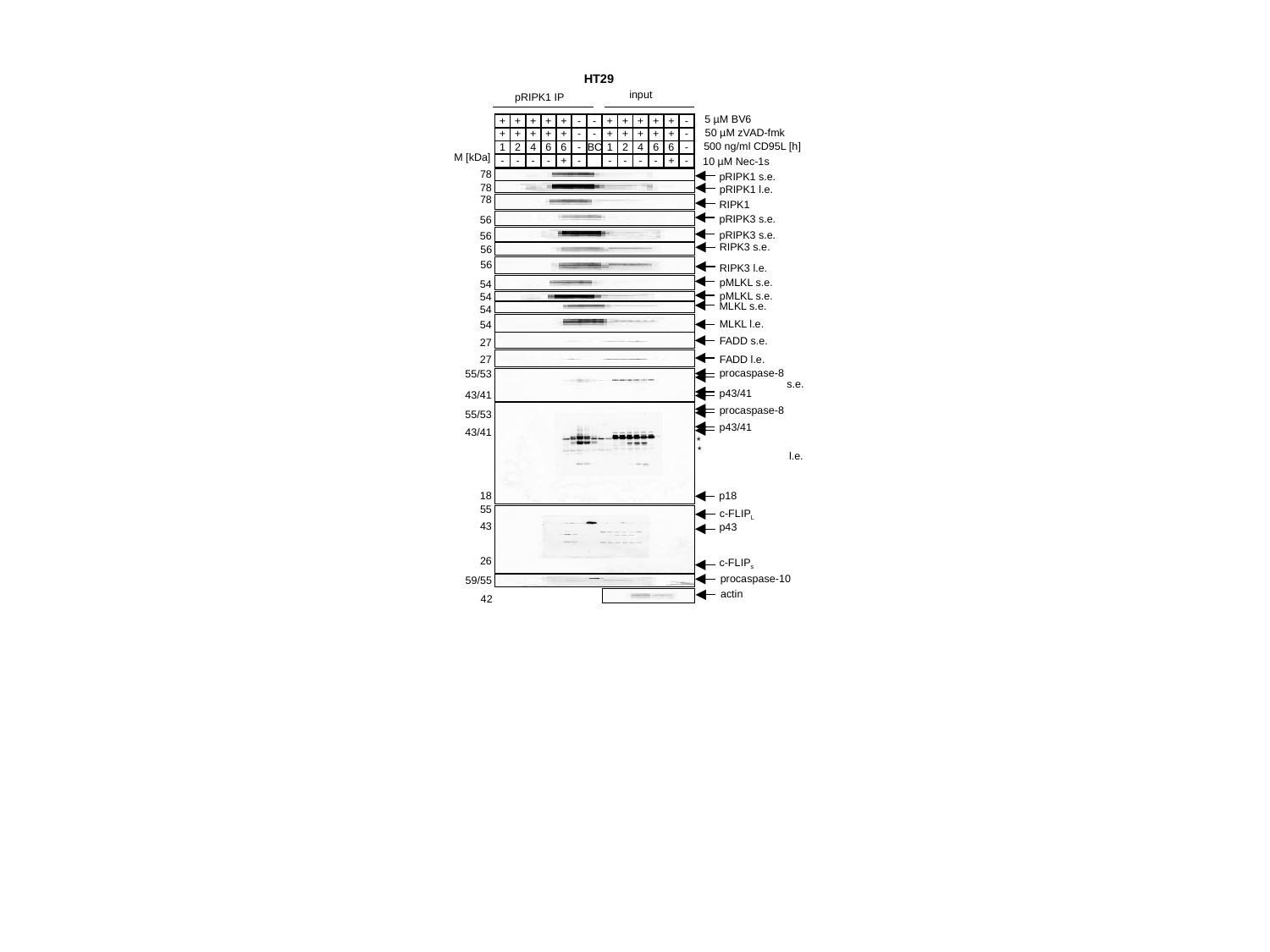

HT29
input
pRIPK1 IP
5 µM BV6
| + | + | + | + | + | - | - | + | + | + | + | + | - |
| --- | --- | --- | --- | --- | --- | --- | --- | --- | --- | --- | --- | --- |
| + | + | + | + | + | - | - | + | + | + | + | + | - |
| 1 | 2 | 4 | 6 | 6 | - | BC | 1 | 2 | 4 | 6 | 6 | - |
| - | - | - | - | + | - | | - | - | - | - | + | - |
50 µM zVAD-fmk
500 ng/ml CD95L [h]
M [kDa]
10 µM Nec-1s
78
pRIPK1 s.e.
78
pRIPK1 l.e.
78
RIPK1
pRIPK3 s.e.
56
pRIPK3 s.e.
56
RIPK3 s.e.
56
56
RIPK3 l.e.
pMLKL s.e.
54
pMLKL s.e.
54
MLKL s.e.
54
MLKL l.e.
54
FADD s.e.
27
FADD l.e.
27
procaspase-8
55/53
s.e.
p43/41
43/41
procaspase-8
55/53
p43/41
43/41
*
*
l.e.
18
p18
55
c-FLIPL
43
p43
26
c-FLIPs
procaspase-10
59/55
actin
42

## Slide 2
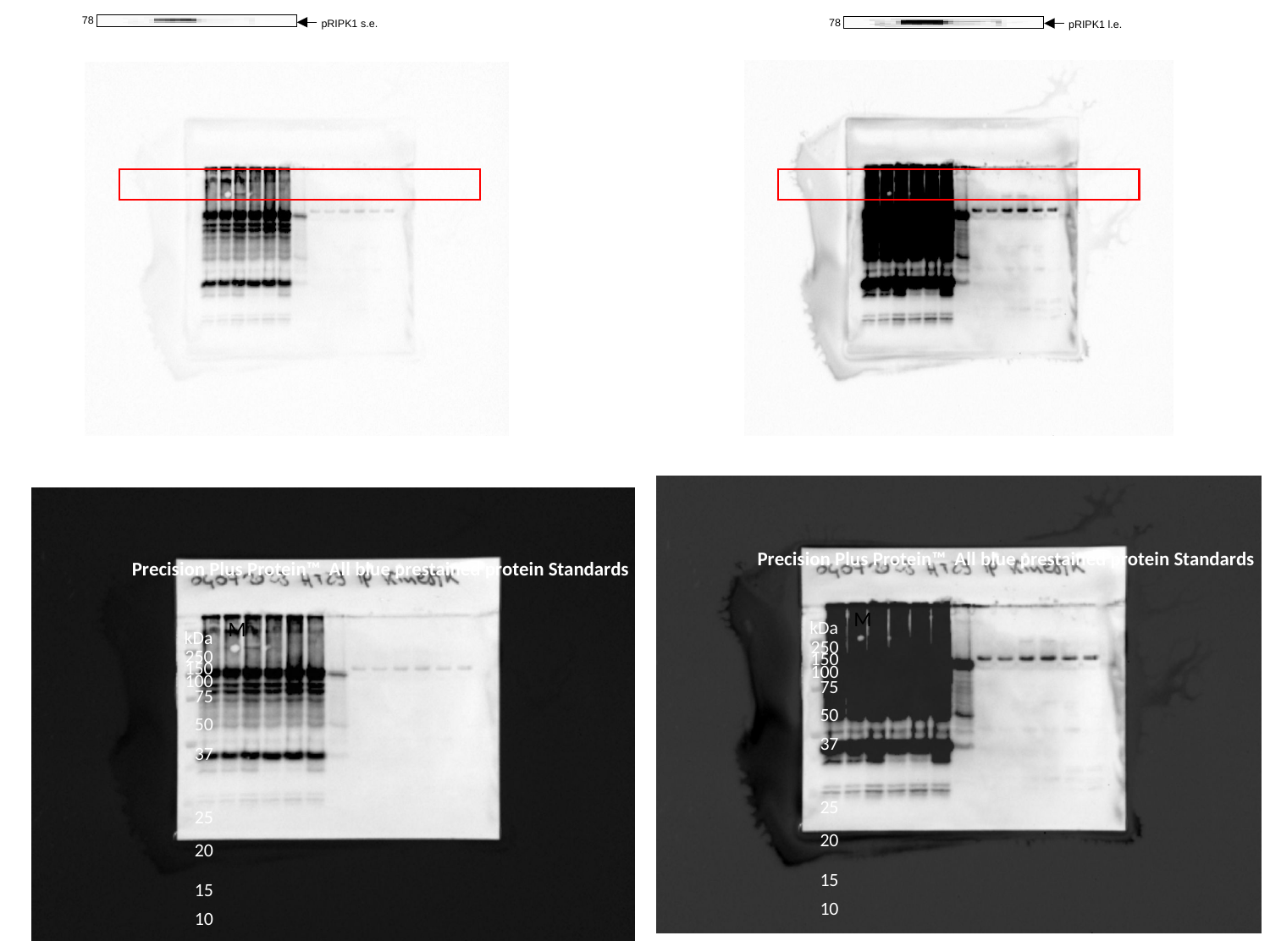

78
78
pRIPK1 s.e.
pRIPK1 l.e.
Precision Plus Protein™ All blue prestained protein Standards
Precision Plus Protein™ All blue prestained protein Standards
M
M
kDa
kDa
250
250
150
150
100
100
75
75
50
50
37
37
25
25
20
20
15
15
10
10

## Slide 3
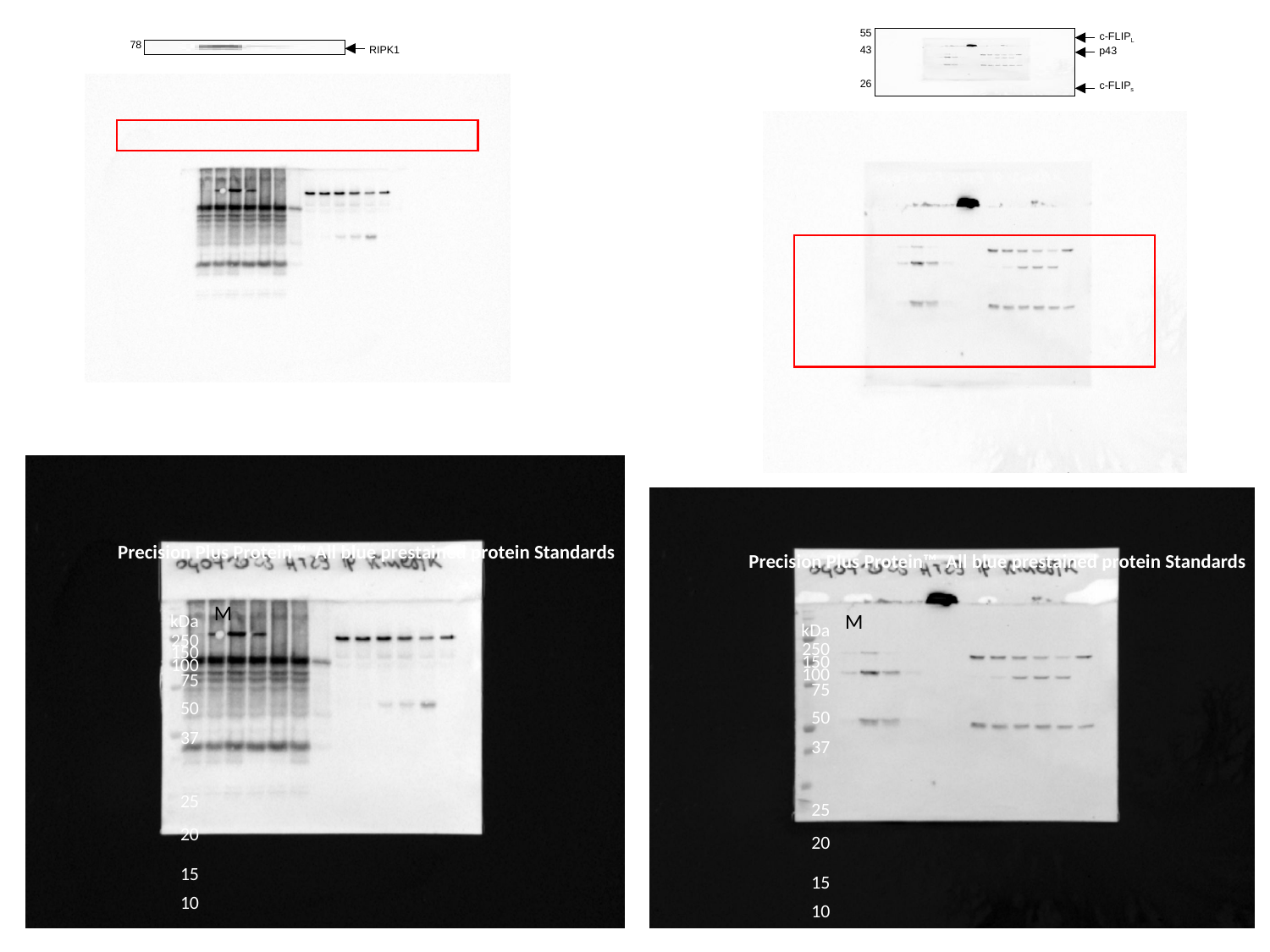

55
c-FLIPL
78
RIPK1
43
p43
26
c-FLIPs
Precision Plus Protein™ All blue prestained protein Standards
Precision Plus Protein™ All blue prestained protein Standards
M
M
kDa
kDa
250
250
150
150
100
100
75
75
50
50
37
37
25
25
20
20
15
15
10
10

## Slide 4
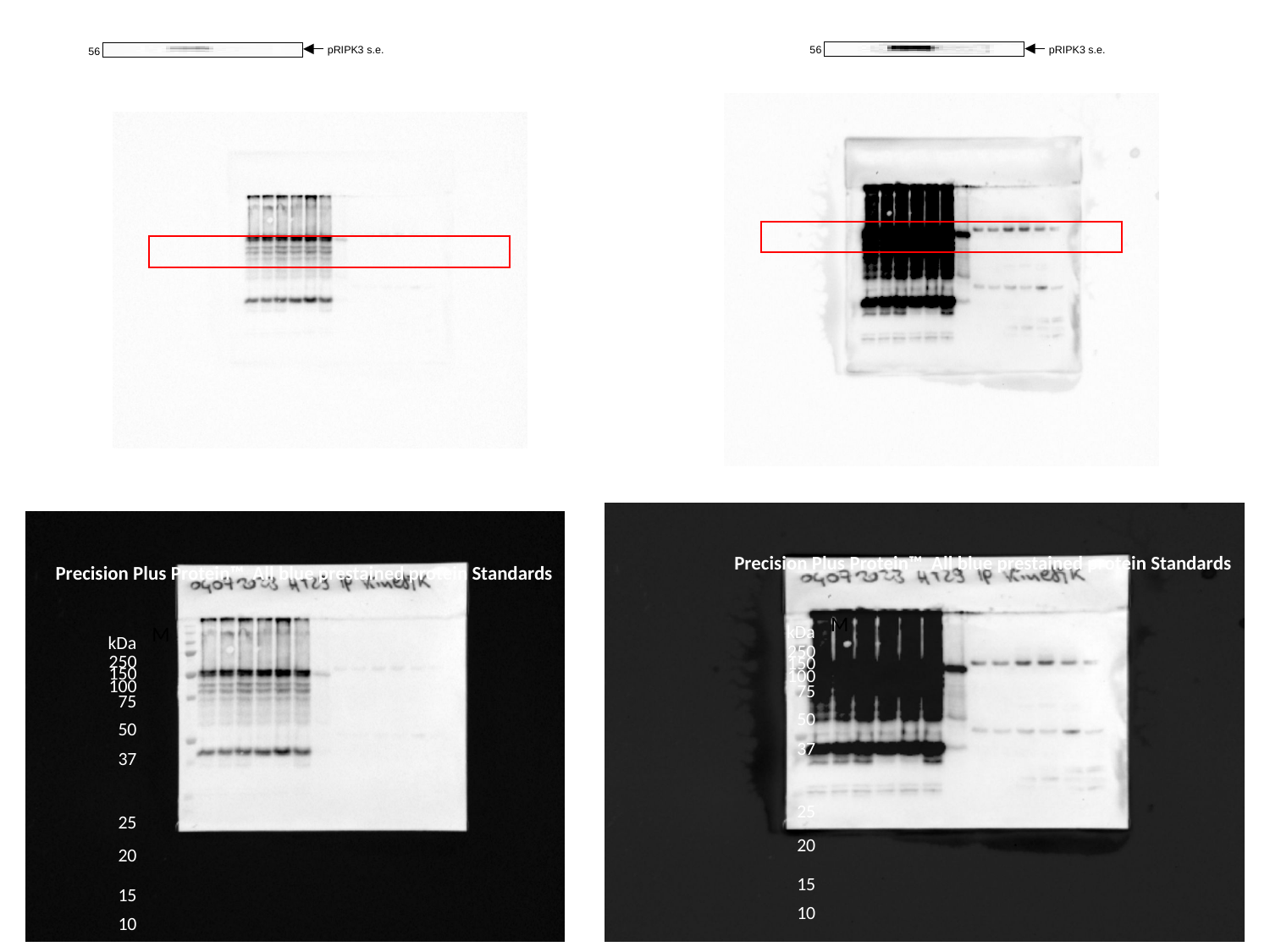

pRIPK3 s.e.
56
pRIPK3 s.e.
56
Precision Plus Protein™ All blue prestained protein Standards
Precision Plus Protein™ All blue prestained protein Standards
M
kDa
M
kDa
250
250
150
150
100
100
75
75
50
50
37
37
25
25
20
20
15
15
10
10

## Slide 5
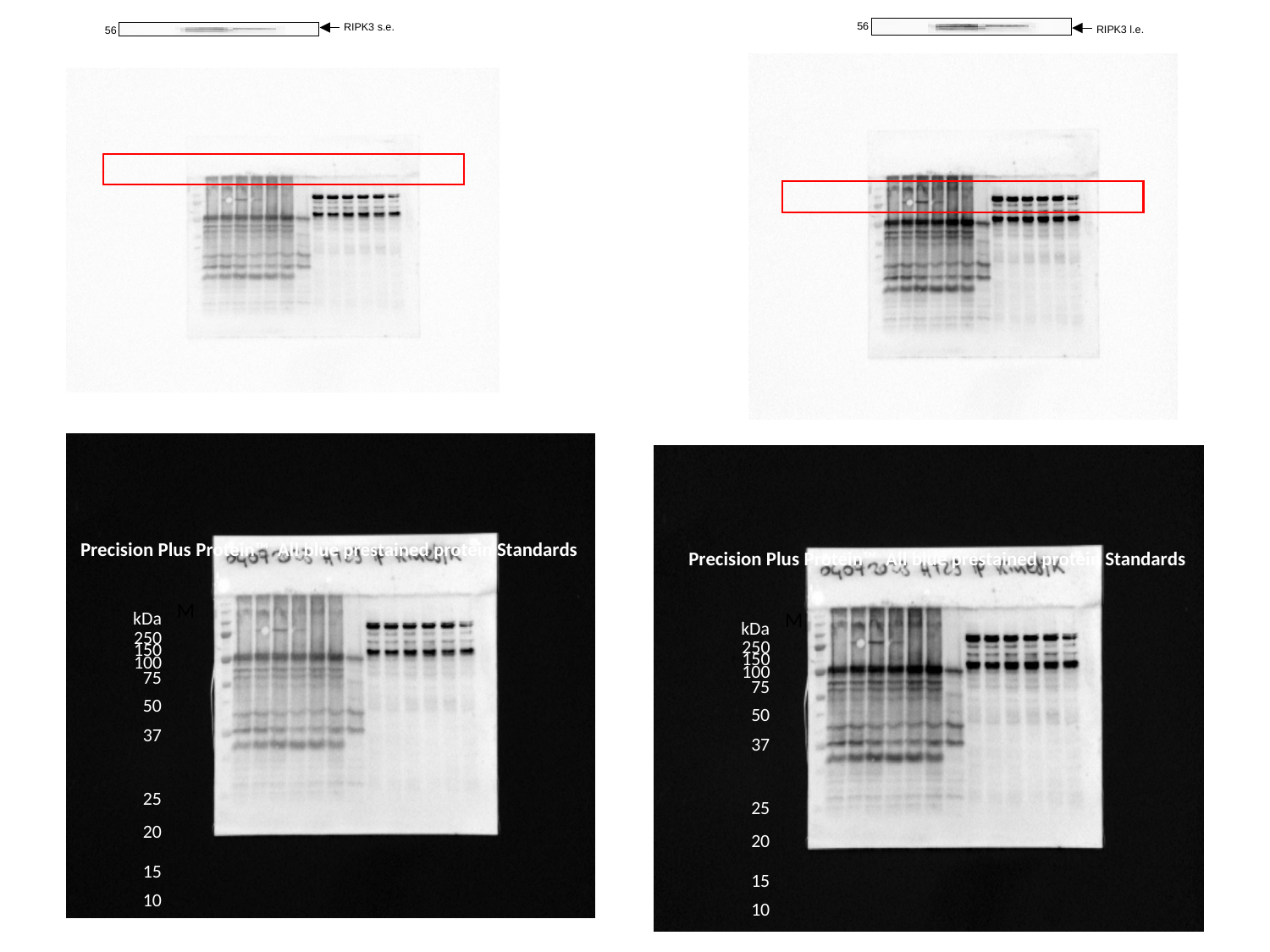

56
RIPK3 s.e.
RIPK3 l.e.
56
Precision Plus Protein™ All blue prestained protein Standards
Precision Plus Protein™ All blue prestained protein Standards
M
M
kDa
kDa
250
250
150
150
100
100
75
75
50
50
37
37
25
25
20
20
15
15
10
10

## Slide 6
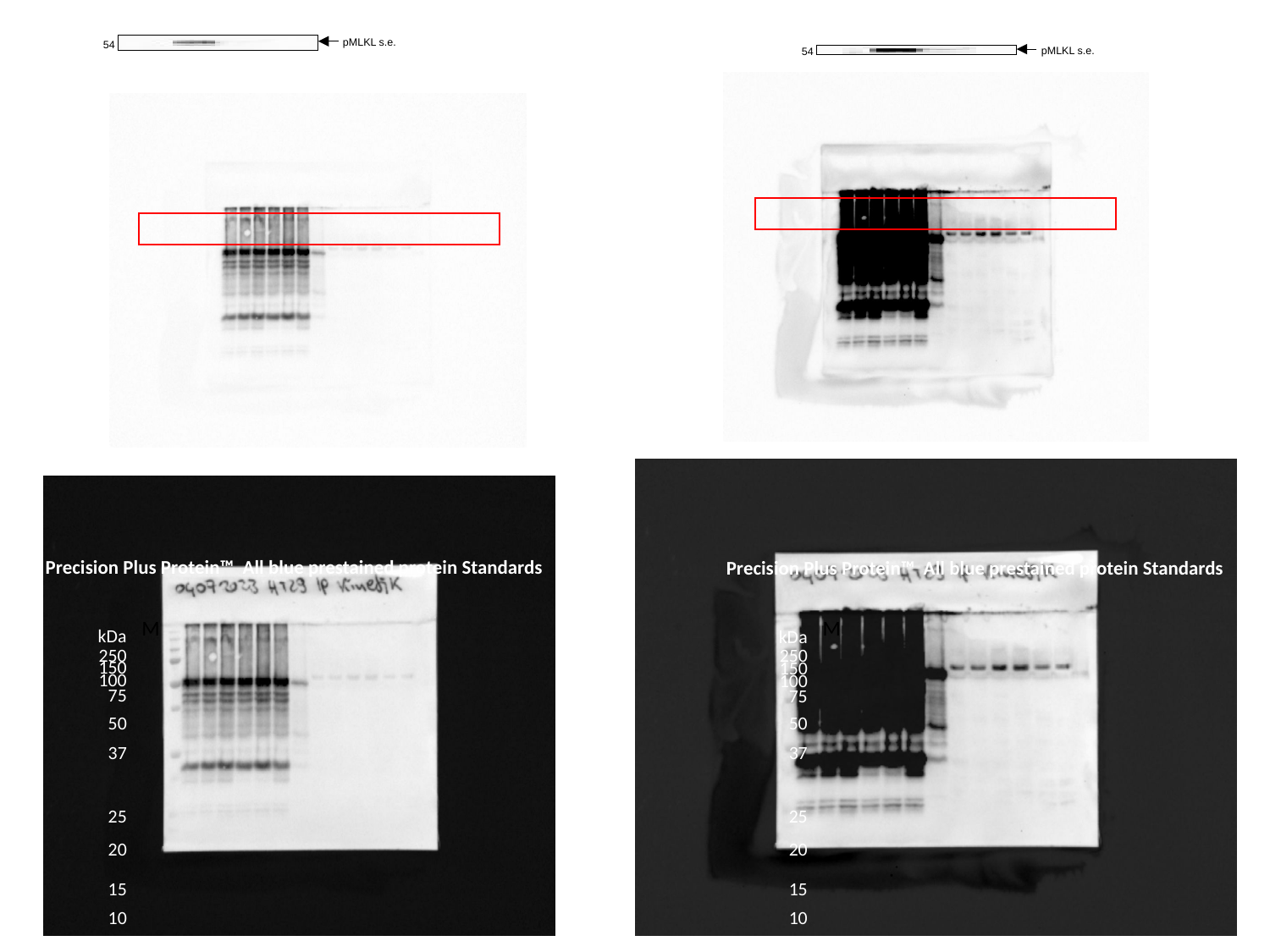

pMLKL s.e.
54
pMLKL s.e.
54
Precision Plus Protein™ All blue prestained protein Standards
Precision Plus Protein™ All blue prestained protein Standards
M
M
kDa
kDa
250
250
150
150
100
100
75
75
50
50
37
37
25
25
20
20
15
15
10
10

## Slide 7
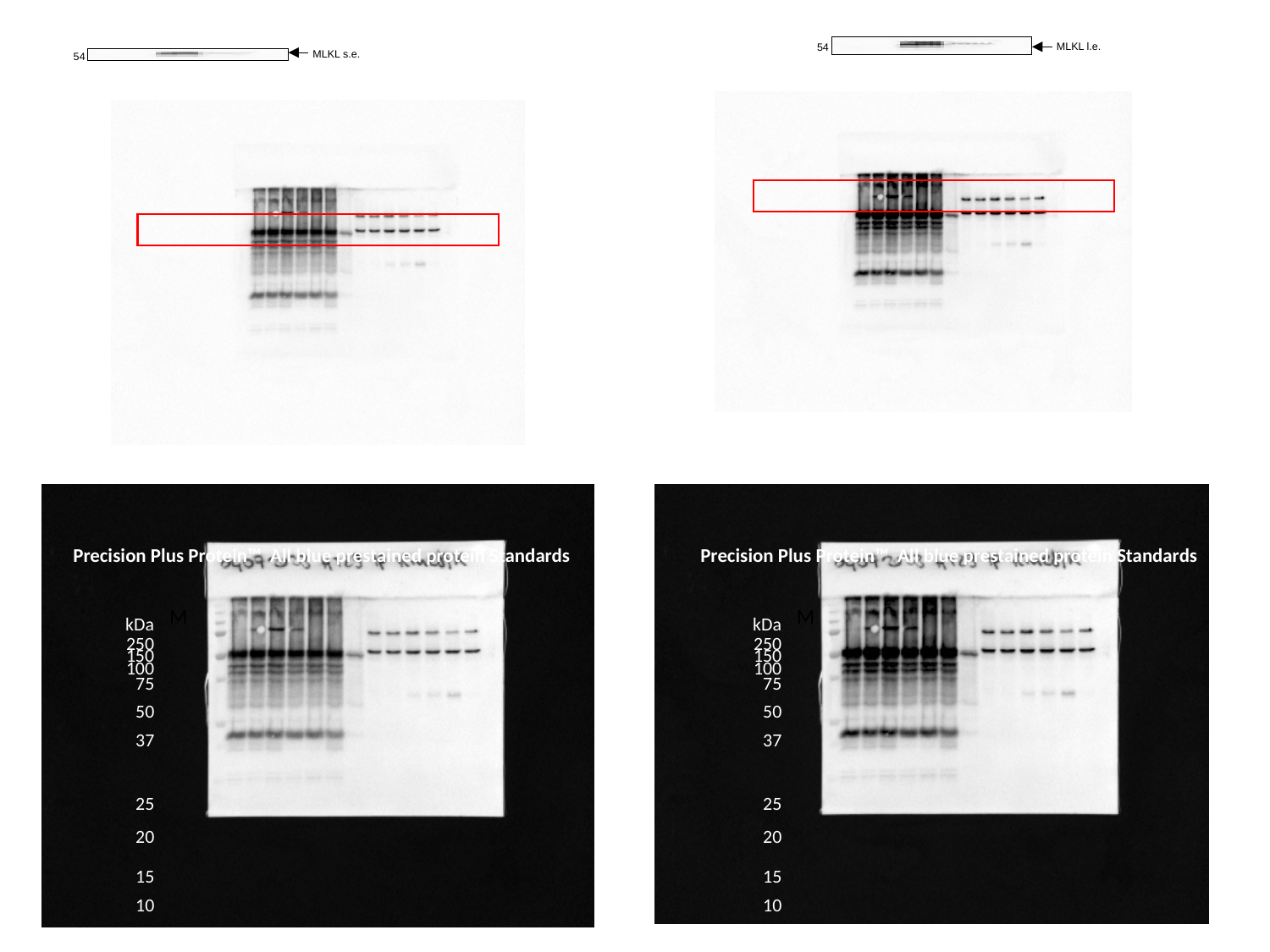

MLKL l.e.
54
MLKL s.e.
54
Precision Plus Protein™ All blue prestained protein Standards
Precision Plus Protein™ All blue prestained protein Standards
M
M
kDa
kDa
250
250
150
150
100
100
75
75
50
50
37
37
25
25
20
20
15
15
10
10

## Slide 8
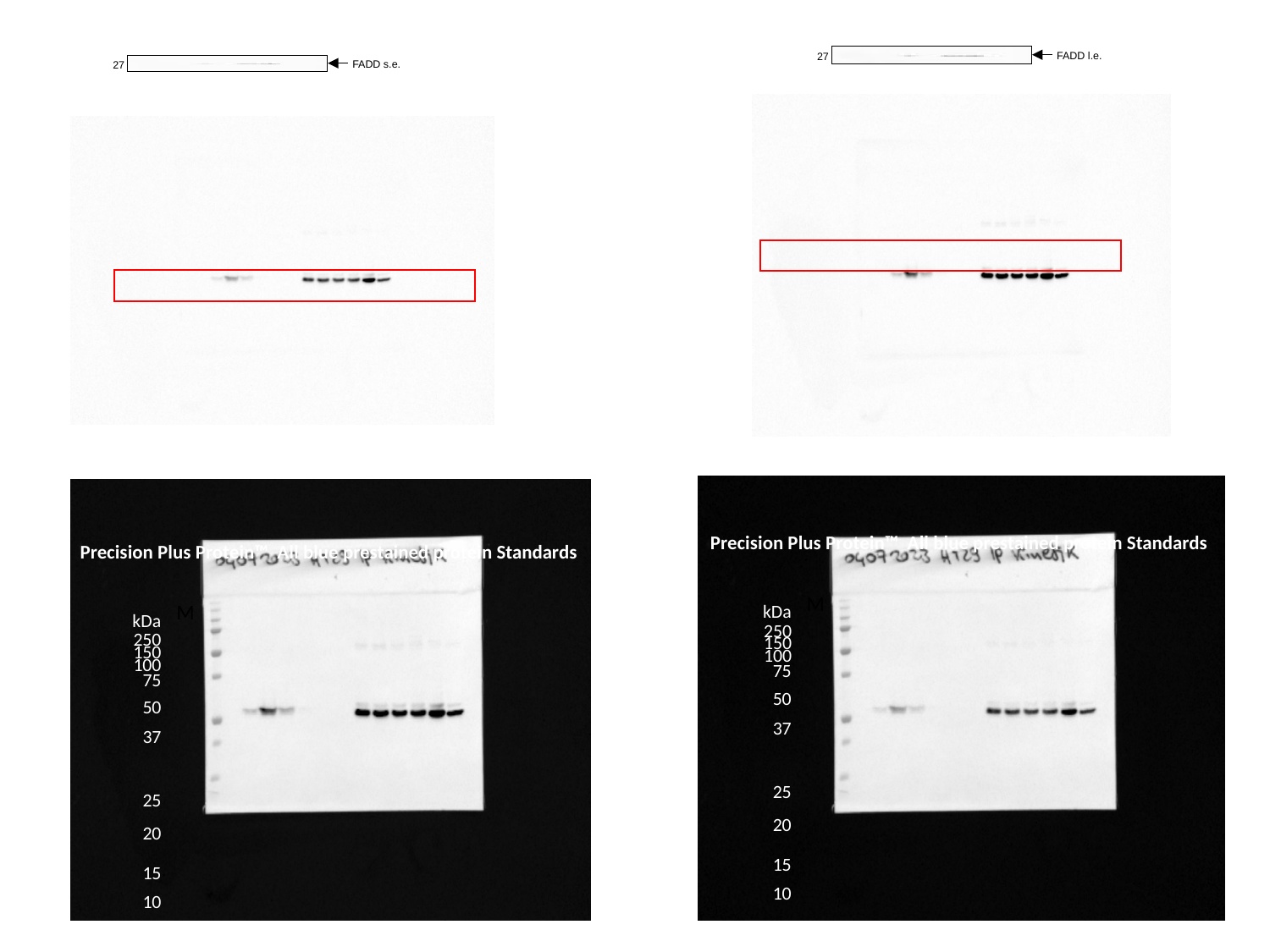

FADD l.e.
27
FADD s.e.
27
Precision Plus Protein™ All blue prestained protein Standards
Precision Plus Protein™ All blue prestained protein Standards
M
M
kDa
kDa
250
250
150
150
100
100
75
75
50
50
37
37
25
25
20
20
15
15
10
10

## Slide 9
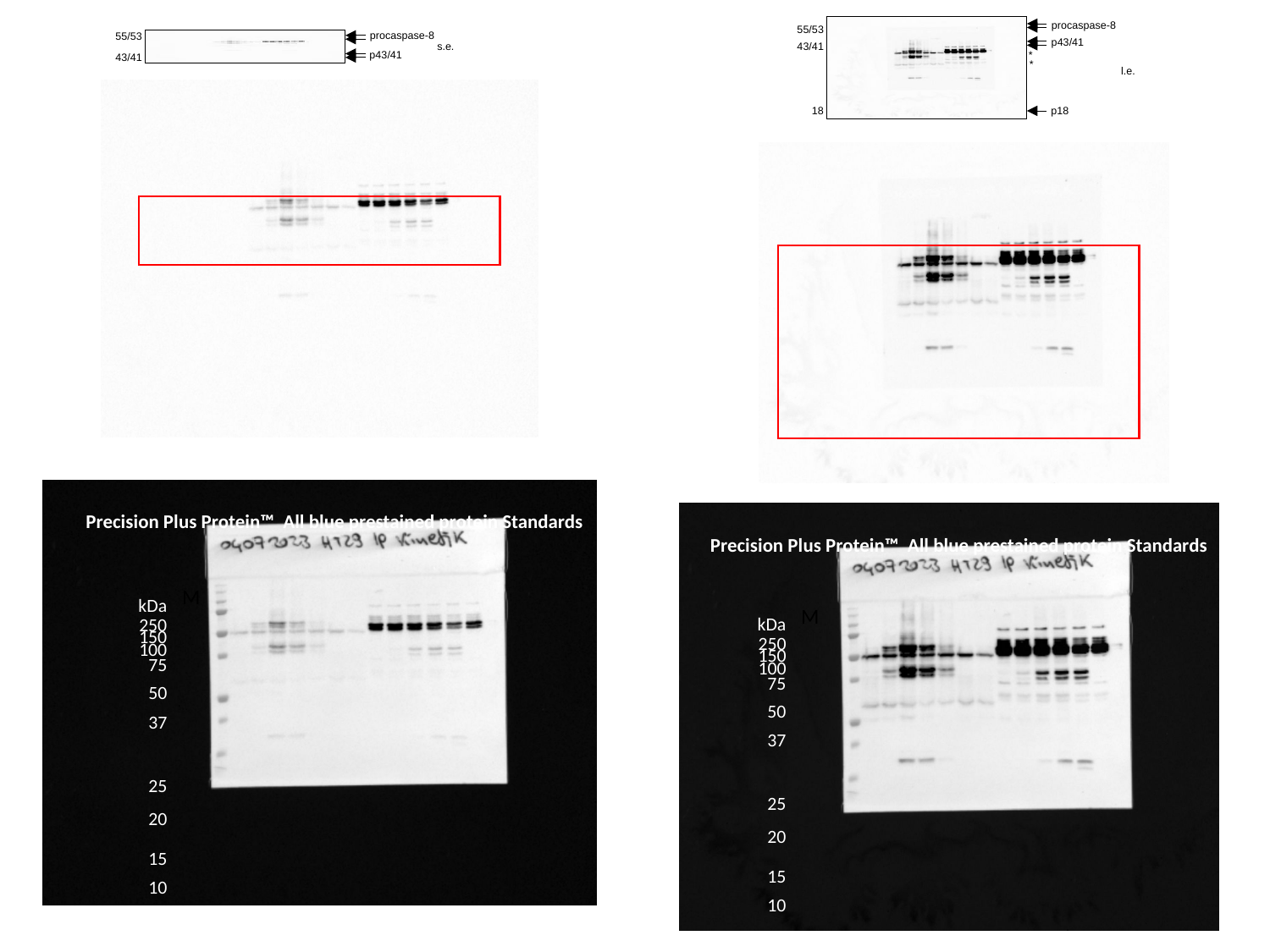

procaspase-8
55/53
procaspase-8
55/53
p43/41
s.e.
43/41
p43/41
*
43/41
*
l.e.
18
p18
Precision Plus Protein™ All blue prestained protein Standards
Precision Plus Protein™ All blue prestained protein Standards
M
kDa
M
kDa
250
150
250
100
150
75
100
75
50
50
37
37
25
25
20
20
15
15
10
10

## Slide 10
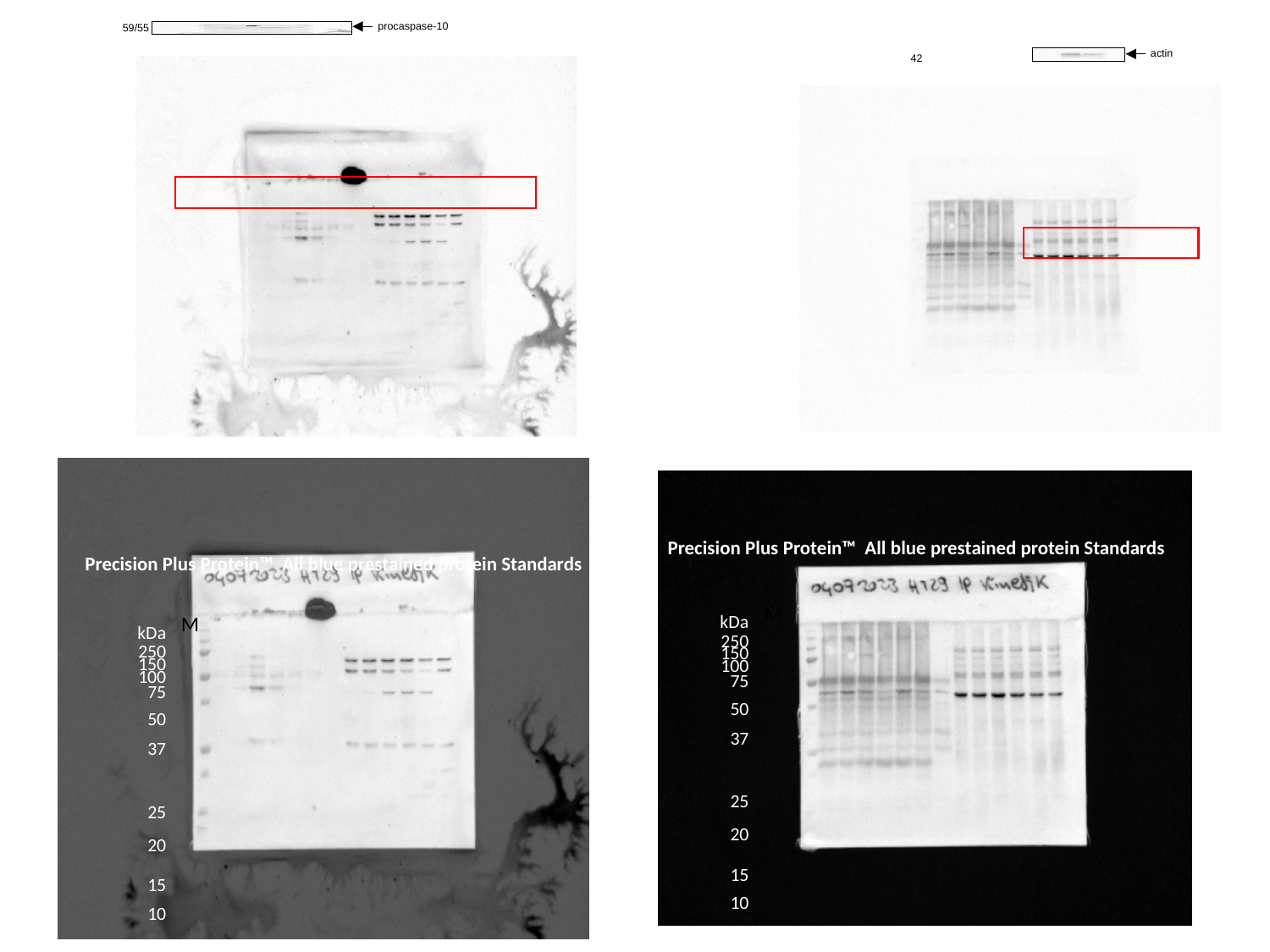

procaspase-10
59/55
actin
42
Precision Plus Protein™ All blue prestained protein Standards
Precision Plus Protein™ All blue prestained protein Standards
M
kDa
M
kDa
250
250
150
150
100
100
75
75
50
50
37
37
25
25
20
20
15
15
10
10
