# Supplementary material for: Oligomerised RIPK1 is the main core component of the CD95 necrosome
Source: EMBO J. 2025 Apr 16;44(11):3231–65. doi: 10.1038/s44318-025-00433-0 (PMC12130296; doi:10.1038/s44318-025-00433-0)
Supplement: Supplementary file 6 — Source data Fig. 2 [file 44318_2025_433_MOESM6_ESM.zip › figure2D.pptx]

## Slide 1
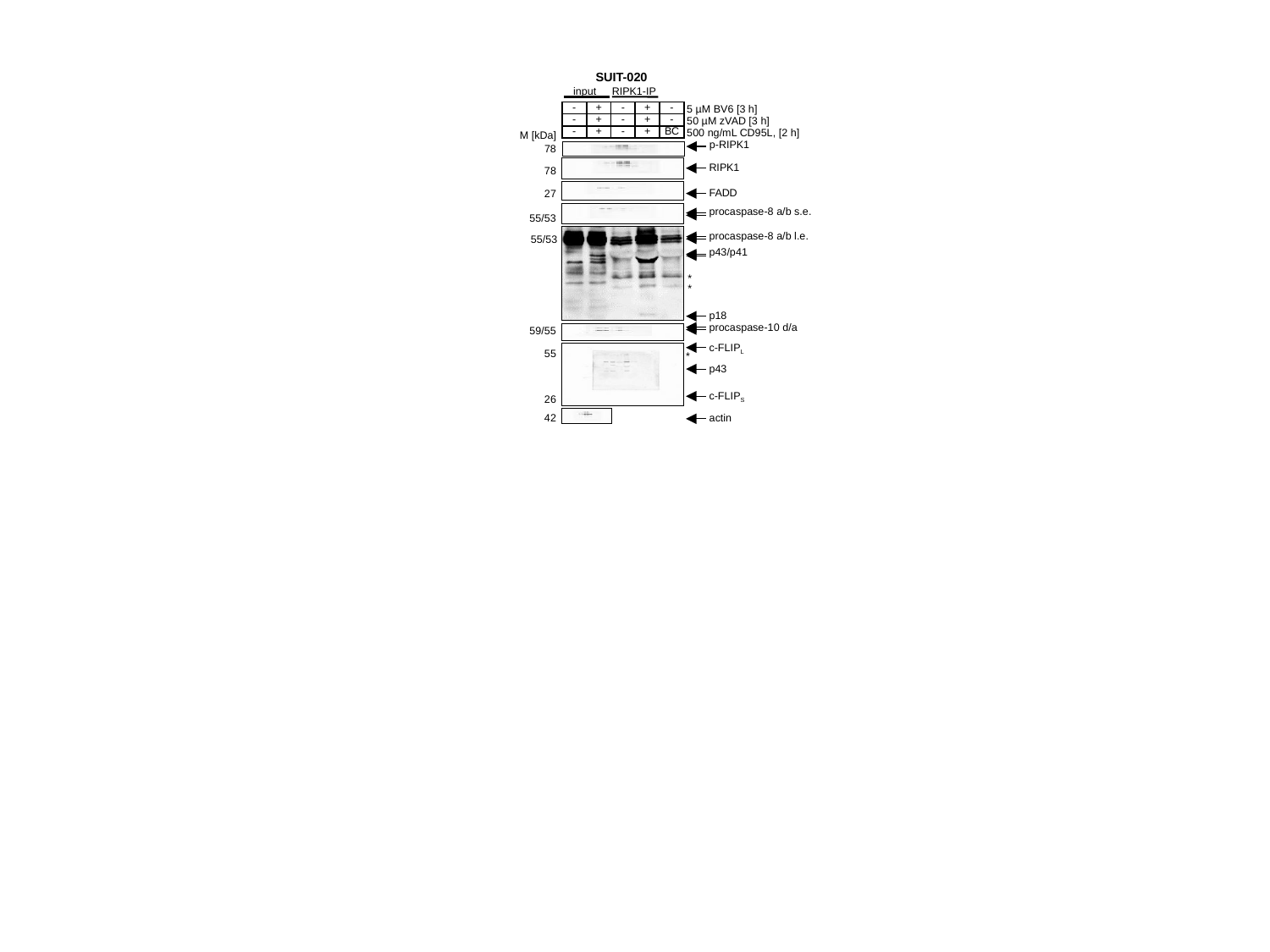

SUIT-020
input
RIPK1-IP
5 µM BV6 [3 h]
| - | + | - | + | - |
| --- | --- | --- | --- | --- |
| - | + | - | + | - |
| - | + | - | + | BC |
50 µM zVAD [3 h]
500 ng/mL CD95L, [2 h]
M [kDa]
p-RIPK1
78
RIPK1
78
FADD
27
procaspase-8 a/b s.e.
55/53
procaspase-8 a/b l.e.
55/53
p43/p41
*
*
p18
procaspase-10 d/a
59/55
c-FLIPL
55
*
p43
c-FLIPS
26
actin
42

## Slide 2
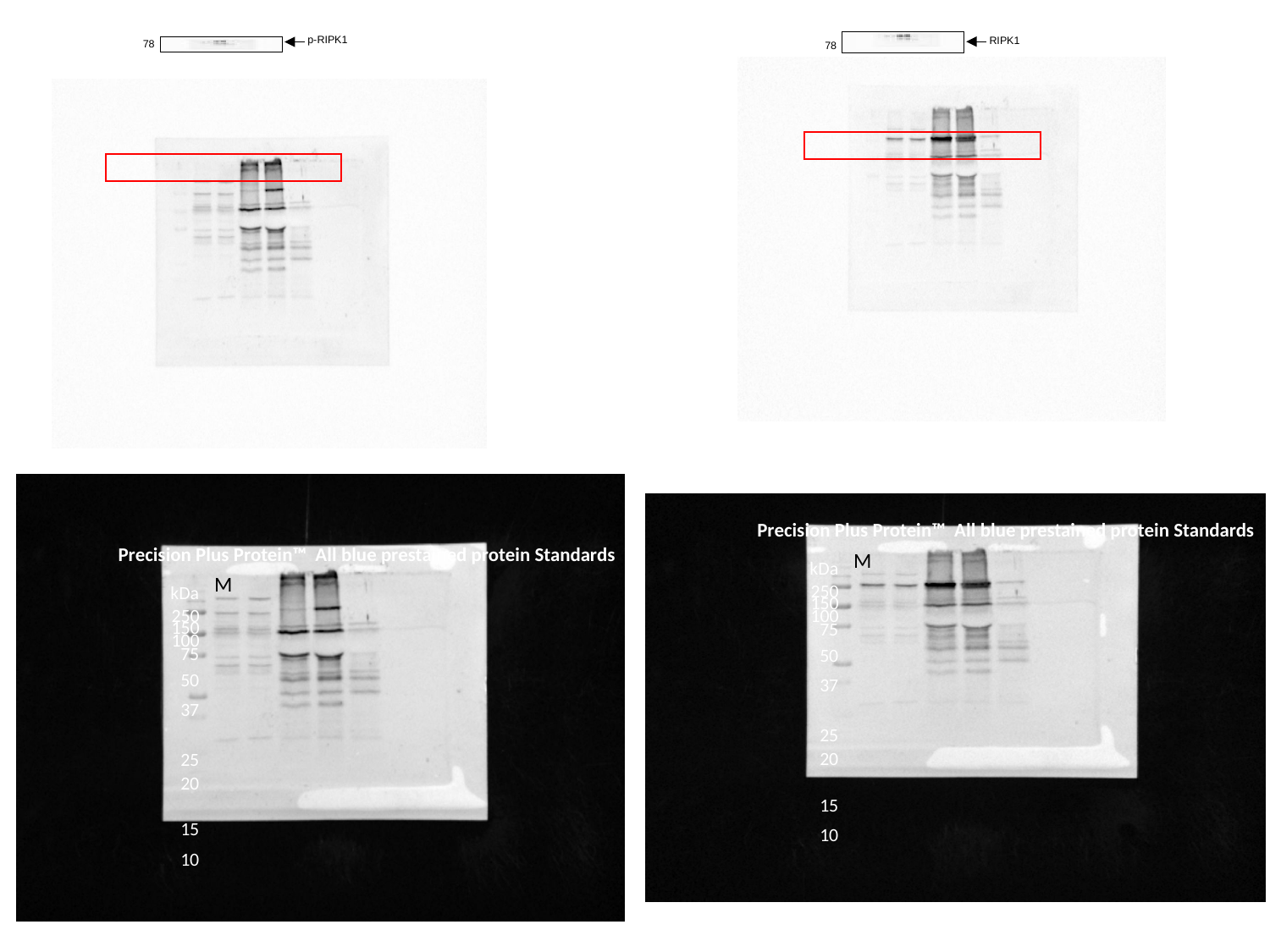

p-RIPK1
RIPK1
78
78
Precision Plus Protein™ All blue prestained protein Standards
Precision Plus Protein™ All blue prestained protein Standards
M
kDa
M
250
kDa
150
250
100
150
75
100
75
50
50
37
37
25
20
25
20
15
15
10
10

## Slide 3
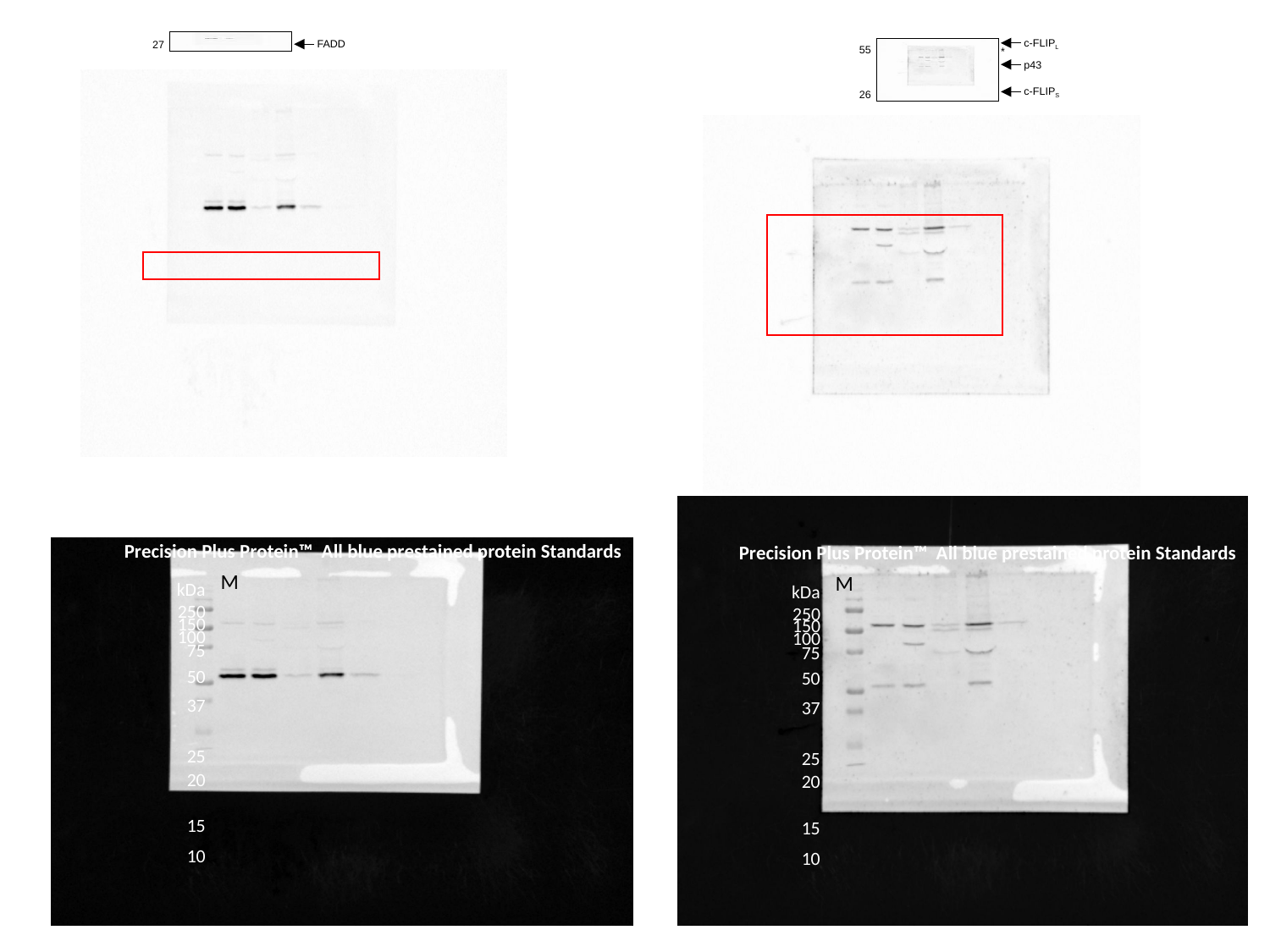

c-FLIPL
FADD
27
55
*
p43
c-FLIPS
26
Precision Plus Protein™ All blue prestained protein Standards
Precision Plus Protein™ All blue prestained protein Standards
M
M
kDa
kDa
250
250
150
150
100
100
75
75
50
50
37
37
25
25
20
20
15
15
10
10

## Slide 4
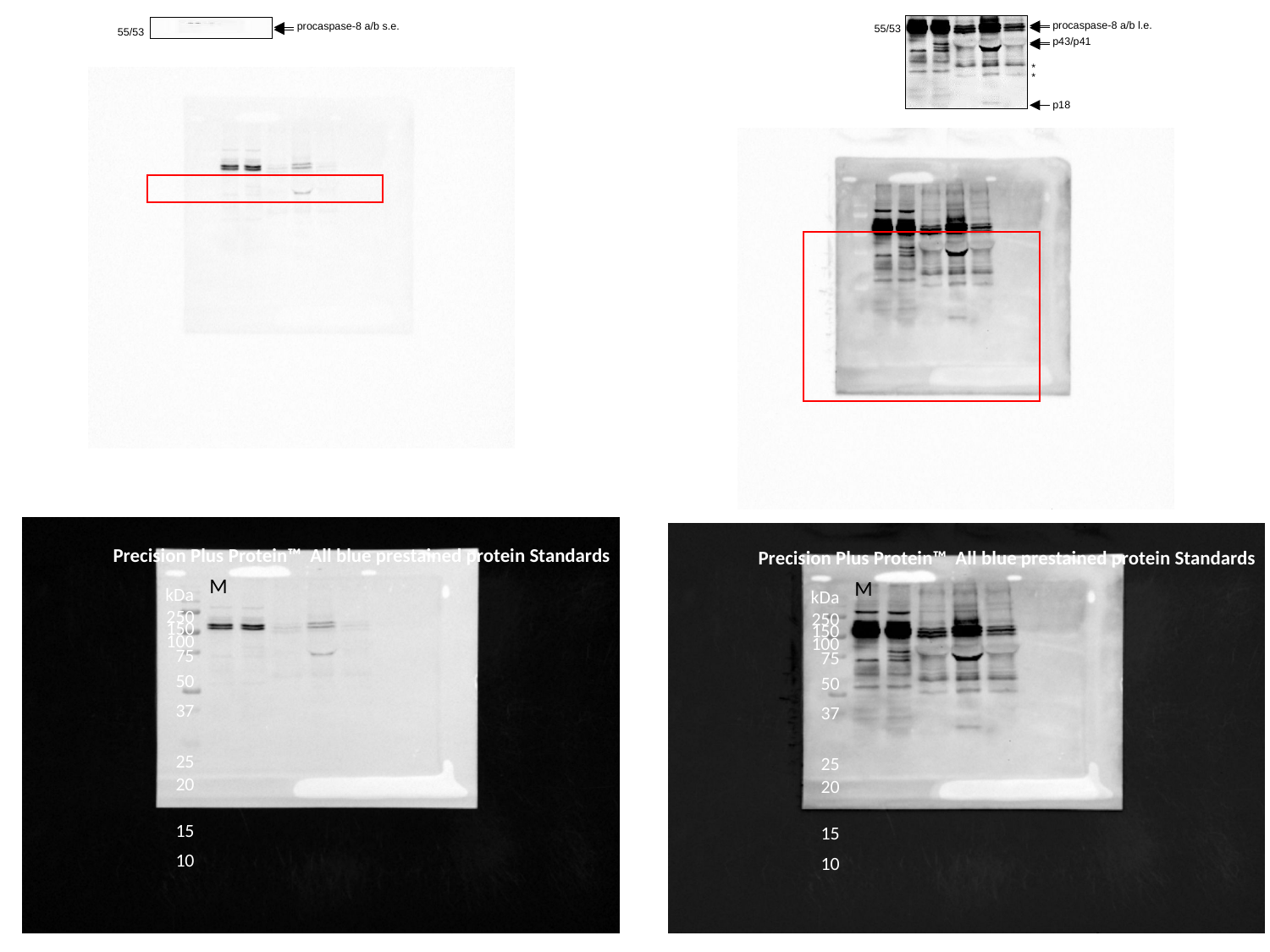

procaspase-8 a/b l.e.
procaspase-8 a/b s.e.
55/53
55/53
p43/p41
*
*
p18
Precision Plus Protein™ All blue prestained protein Standards
Precision Plus Protein™ All blue prestained protein Standards
M
M
kDa
kDa
250
250
150
150
100
100
75
75
50
50
37
37
25
25
20
20
15
15
10
10

## Slide 5
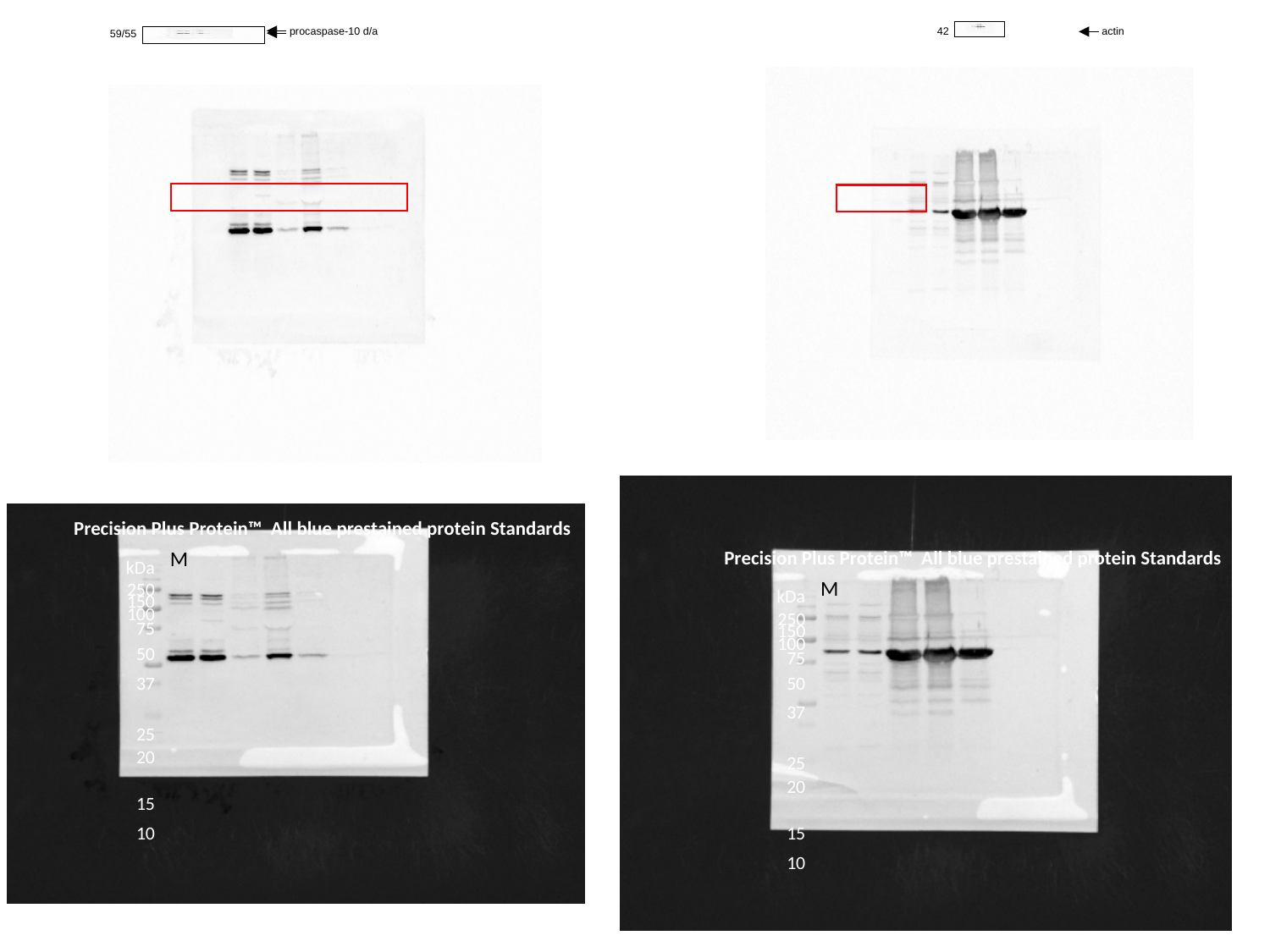

procaspase-10 d/a
actin
42
59/55
Precision Plus Protein™ All blue prestained protein Standards
M
Precision Plus Protein™ All blue prestained protein Standards
kDa
M
250
kDa
150
100
250
75
150
100
50
75
50
37
37
25
20
25
20
15
15
10
10
