# Supplementary material for: Oligomerised RIPK1 is the main core component of the CD95 necrosome
Source: EMBO J. 2025 Apr 16;44(11):3231–65. doi: 10.1038/s44318-025-00433-0 (PMC12130296; doi:10.1038/s44318-025-00433-0)
Supplement: Supplementary file 6 — Source data Fig. 2 [file 44318_2025_433_MOESM6_ESM.zip › figure2E.pptx]

## Slide 1
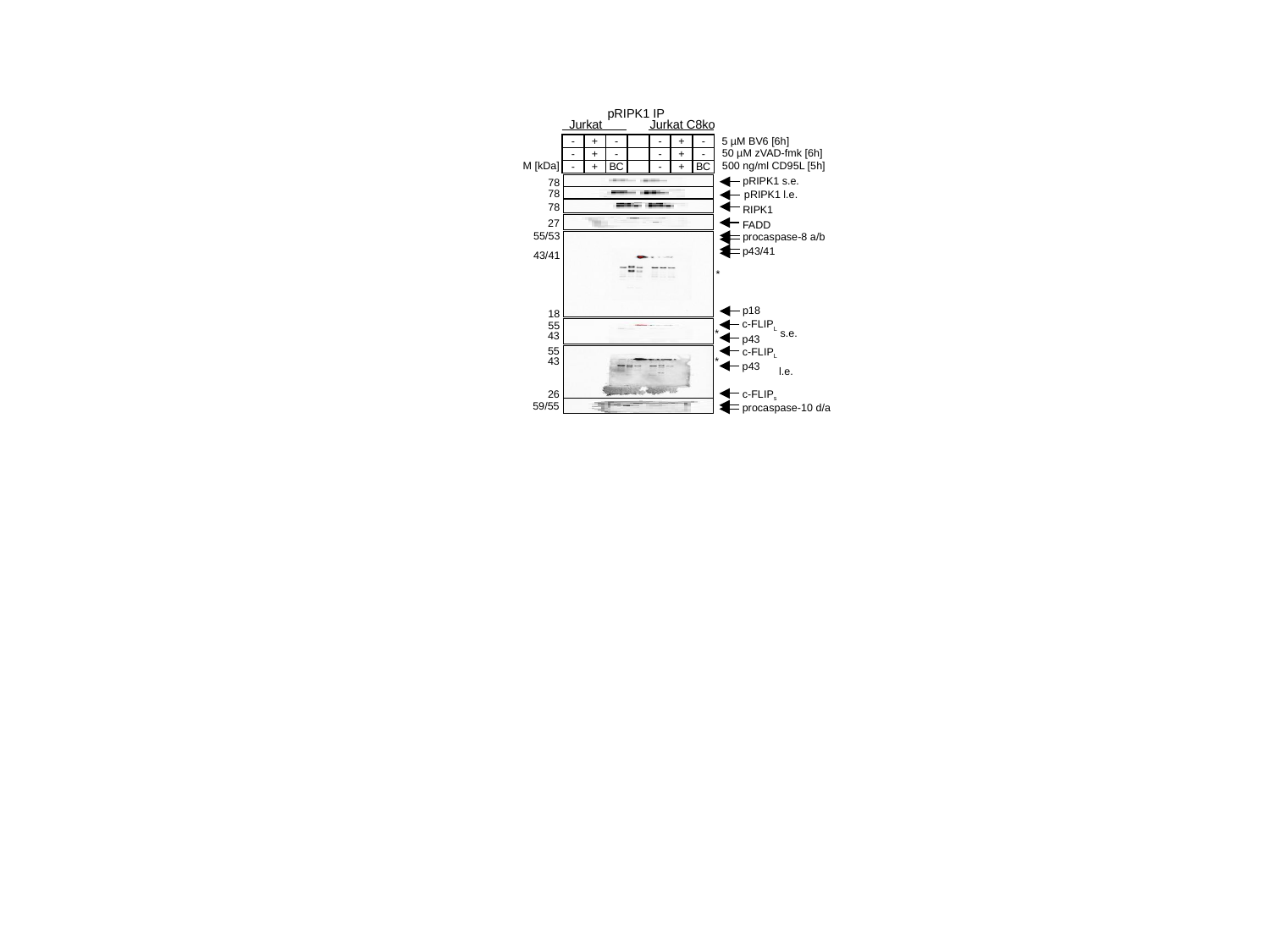

pRIPK1 IP
Jurkat
Jurkat C8ko
5 µM BV6 [6h]
| - | + | - | | - | + | - |
| --- | --- | --- | --- | --- | --- | --- |
| - | + | - | | - | + | - |
| - | + | BC | | - | + | BC |
50 µM zVAD-fmk [6h]
500 ng/ml CD95L [5h]
M [kDa]
pRIPK1 s.e.
78
78
pRIPK1 l.e.
78
RIPK1
27
FADD
55/53
procaspase-8 a/b
p43/41
43/41
*
p18
18
c-FLIPL
55
*
s.e.
43
p43
55
c-FLIPL
*
43
p43
l.e.
26
c-FLIPs
59/55
procaspase-10 d/a

## Slide 2
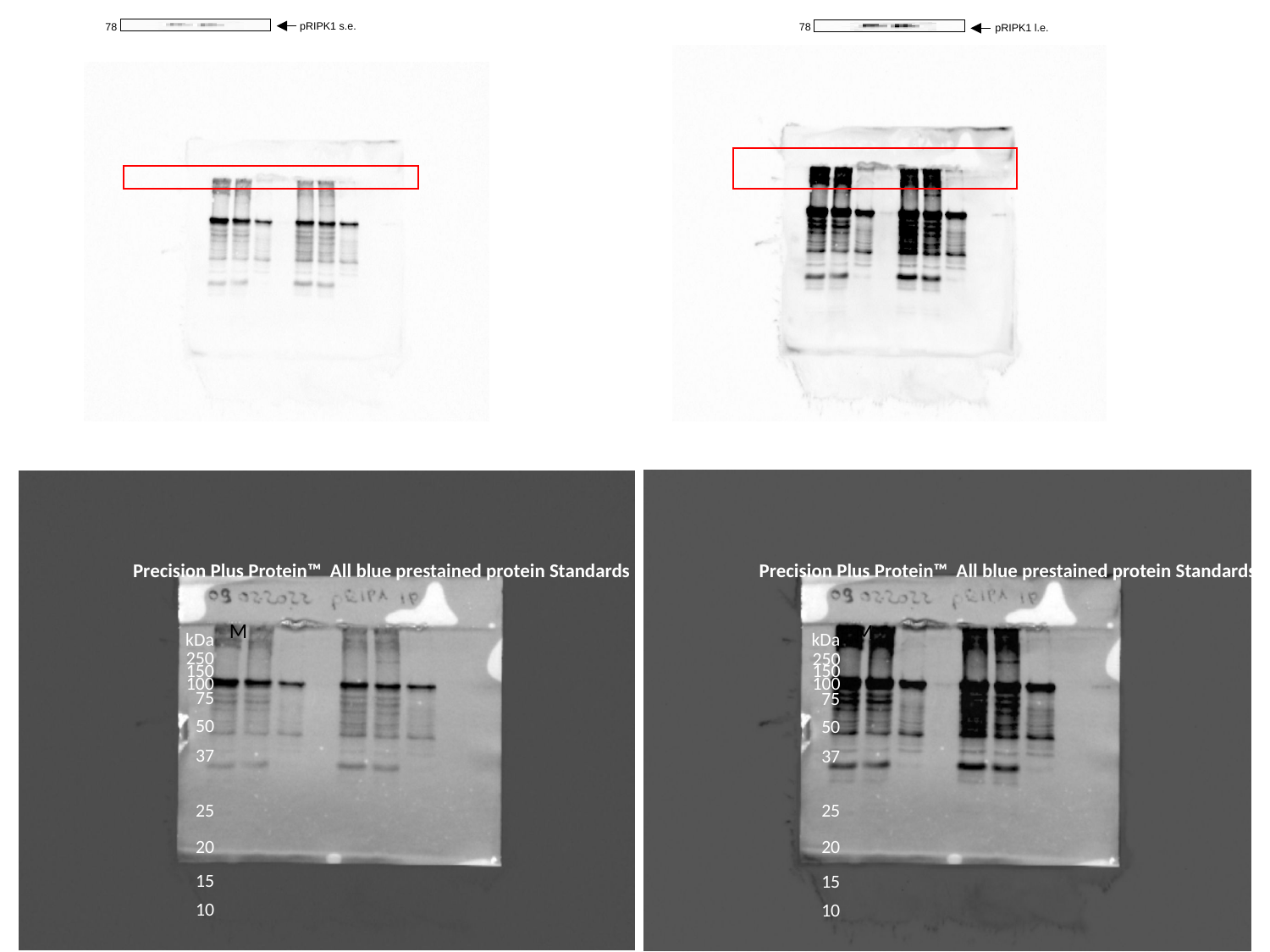

pRIPK1 s.e.
78
78
pRIPK1 l.e.
Precision Plus Protein™ All blue prestained protein Standards
Precision Plus Protein™ All blue prestained protein Standards
M
M
kDa
kDa
250
250
150
150
100
100
75
75
50
50
37
37
25
25
20
20
15
15
10
10

## Slide 3
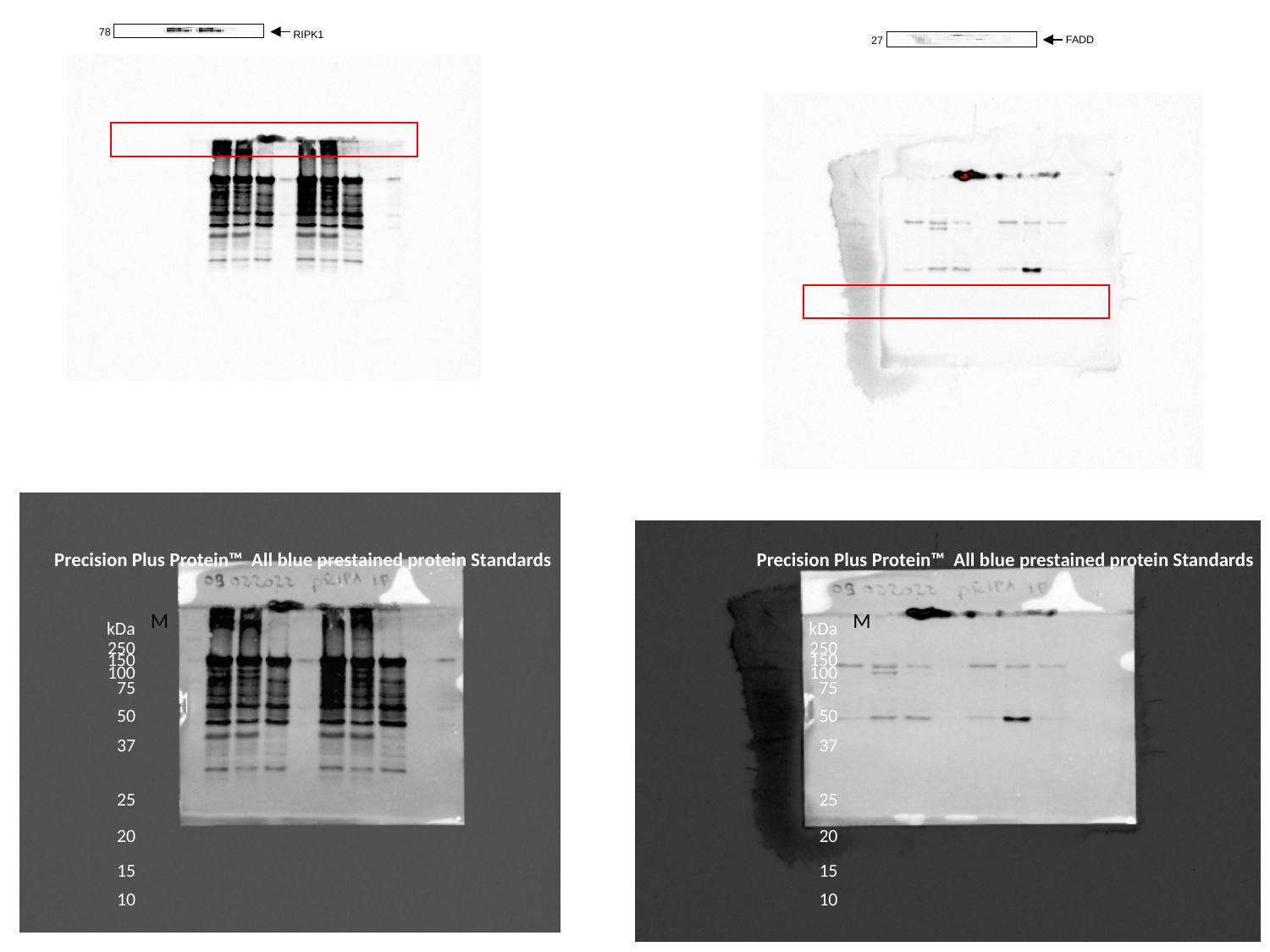

78
RIPK1
FADD
27
Precision Plus Protein™ All blue prestained protein Standards
Precision Plus Protein™ All blue prestained protein Standards
M
M
kDa
kDa
250
250
150
150
100
100
75
75
50
50
37
37
25
25
20
20
15
15
10
10

## Slide 4
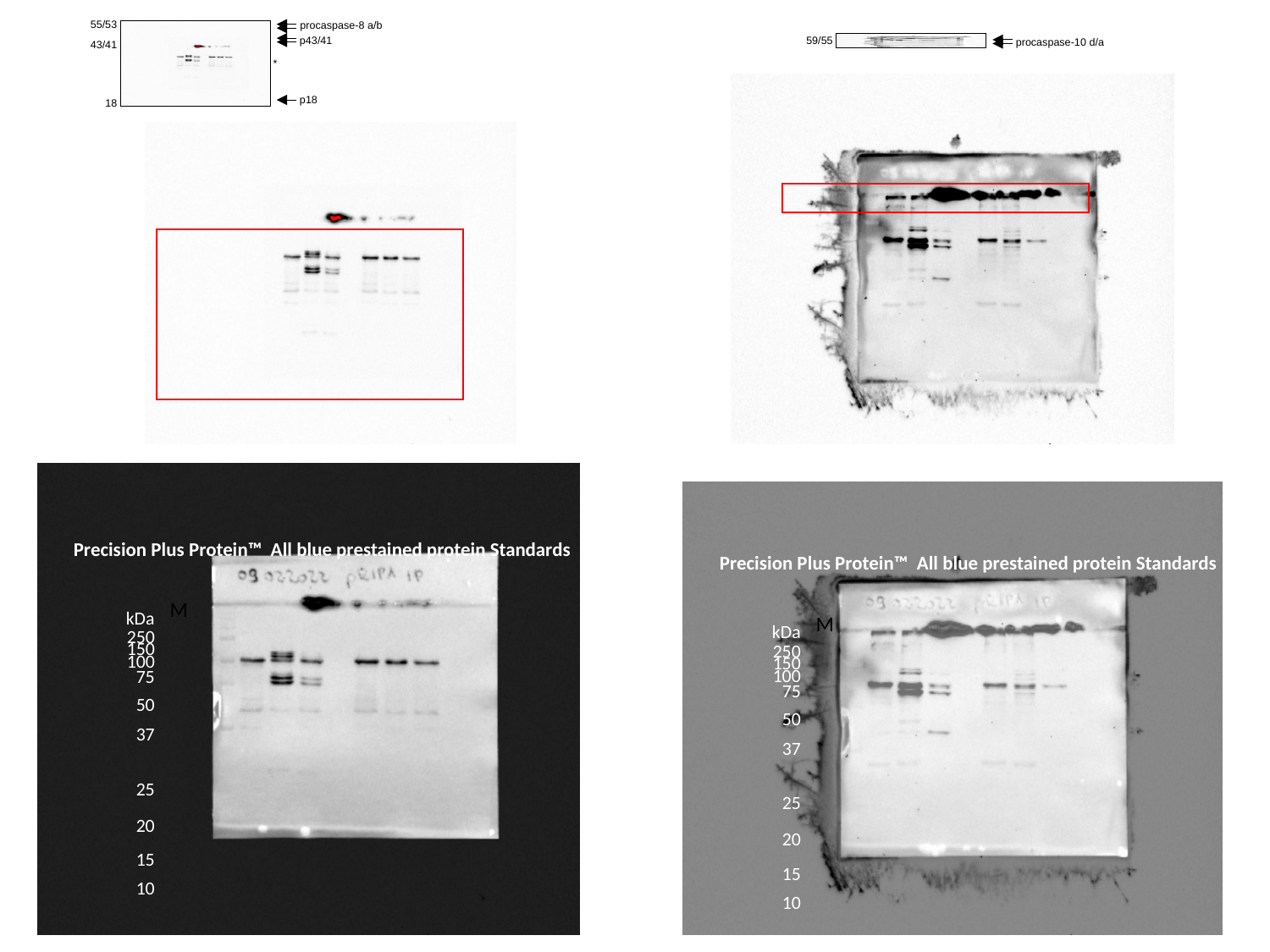

55/53
procaspase-8 a/b
p43/41
59/55
procaspase-10 d/a
43/41
*
p18
18
Precision Plus Protein™ All blue prestained protein Standards
Precision Plus Protein™ All blue prestained protein Standards
M
kDa
M
kDa
250
150
250
100
150
100
75
75
50
50
37
37
25
25
20
20
15
15
10
10

## Slide 5
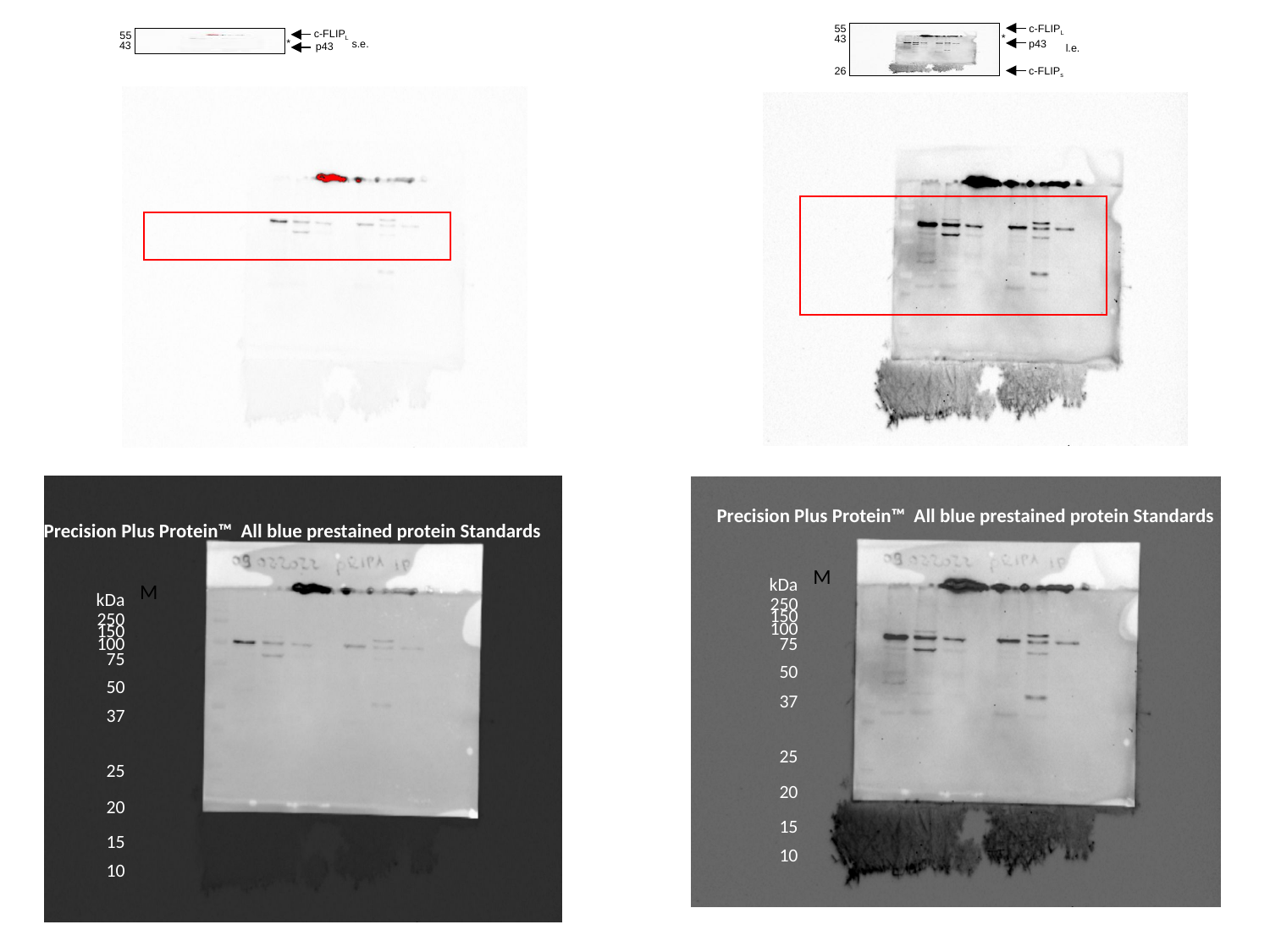

55
c-FLIPL
c-FLIPL
55
*
43
*
p43
s.e.
43
p43
l.e.
26
c-FLIPs
Precision Plus Protein™ All blue prestained protein Standards
Precision Plus Protein™ All blue prestained protein Standards
M
kDa
M
kDa
250
150
250
100
150
75
100
75
50
50
37
37
25
25
20
20
15
15
10
10
