# Supplementary material for: Oligomerised RIPK1 is the main core component of the CD95 necrosome
Source: EMBO J. 2025 Apr 16;44(11):3231–65. doi: 10.1038/s44318-025-00433-0 (PMC12130296; doi:10.1038/s44318-025-00433-0)
Supplement: Supplementary file 6 — Source data Fig. 2 [file 44318_2025_433_MOESM6_ESM.zip › figure2F.pptx]

## Slide 1
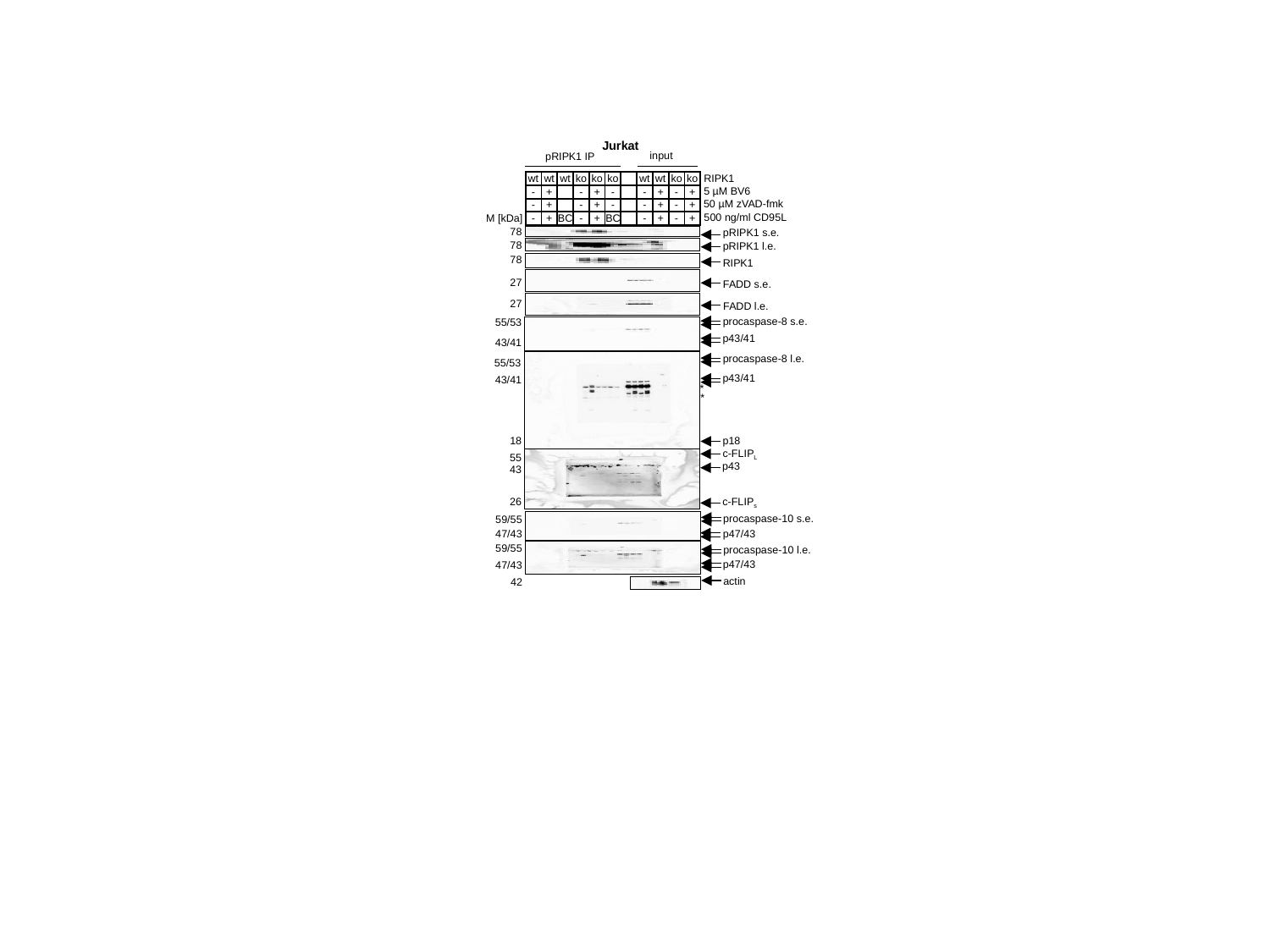

Jurkat
input
pRIPK1 IP
RIPK1
| wt | wt | wt | ko | ko | ko | | wt | wt | ko | ko |
| --- | --- | --- | --- | --- | --- | --- | --- | --- | --- | --- |
| - | + | | - | + | - | | - | + | - | + |
| - | + | | - | + | - | | - | + | - | + |
| - | + | BC | - | + | BC | | - | + | - | + |
5 µM BV6
50 µM zVAD-fmk
500 ng/ml CD95L
M [kDa]
78
pRIPK1 s.e.
78
pRIPK1 l.e.
78
RIPK1
27
FADD s.e.
27
FADD l.e.
procaspase-8 s.e.
55/53
p43/41
43/41
procaspase-8 l.e.
55/53
p43/41
43/41
*
*
p18
18
c-FLIPL
55
p43
43
26
c-FLIPs
procaspase-10 s.e.
59/55
47/43
p47/43
59/55
procaspase-10 l.e.
p47/43
47/43
actin
42

## Slide 2
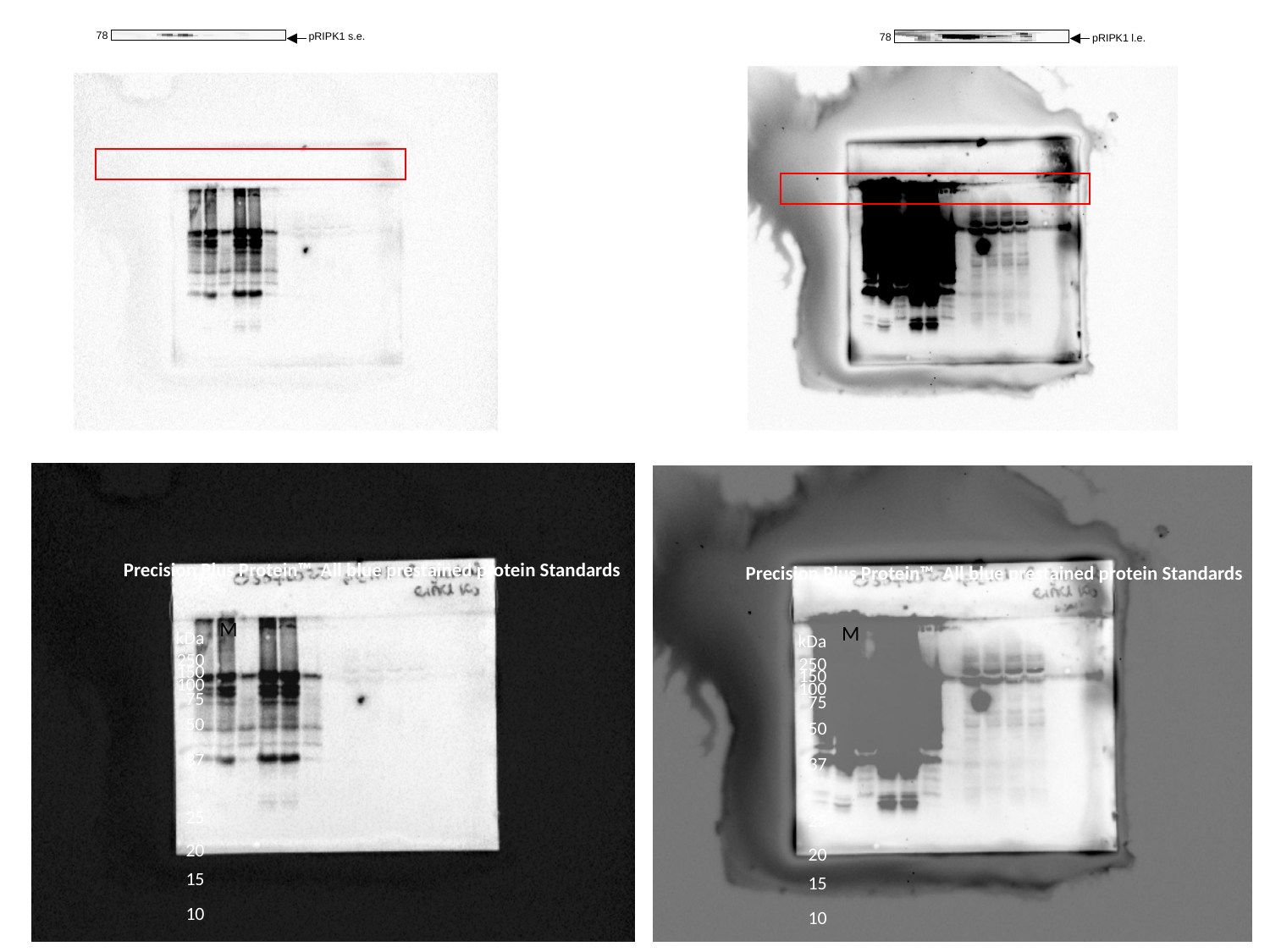

78
pRIPK1 s.e.
78
pRIPK1 l.e.
Precision Plus Protein™ All blue prestained protein Standards
Precision Plus Protein™ All blue prestained protein Standards
M
M
kDa
kDa
250
250
150
150
100
100
75
75
50
50
37
37
25
25
20
20
15
15
10
10

## Slide 3
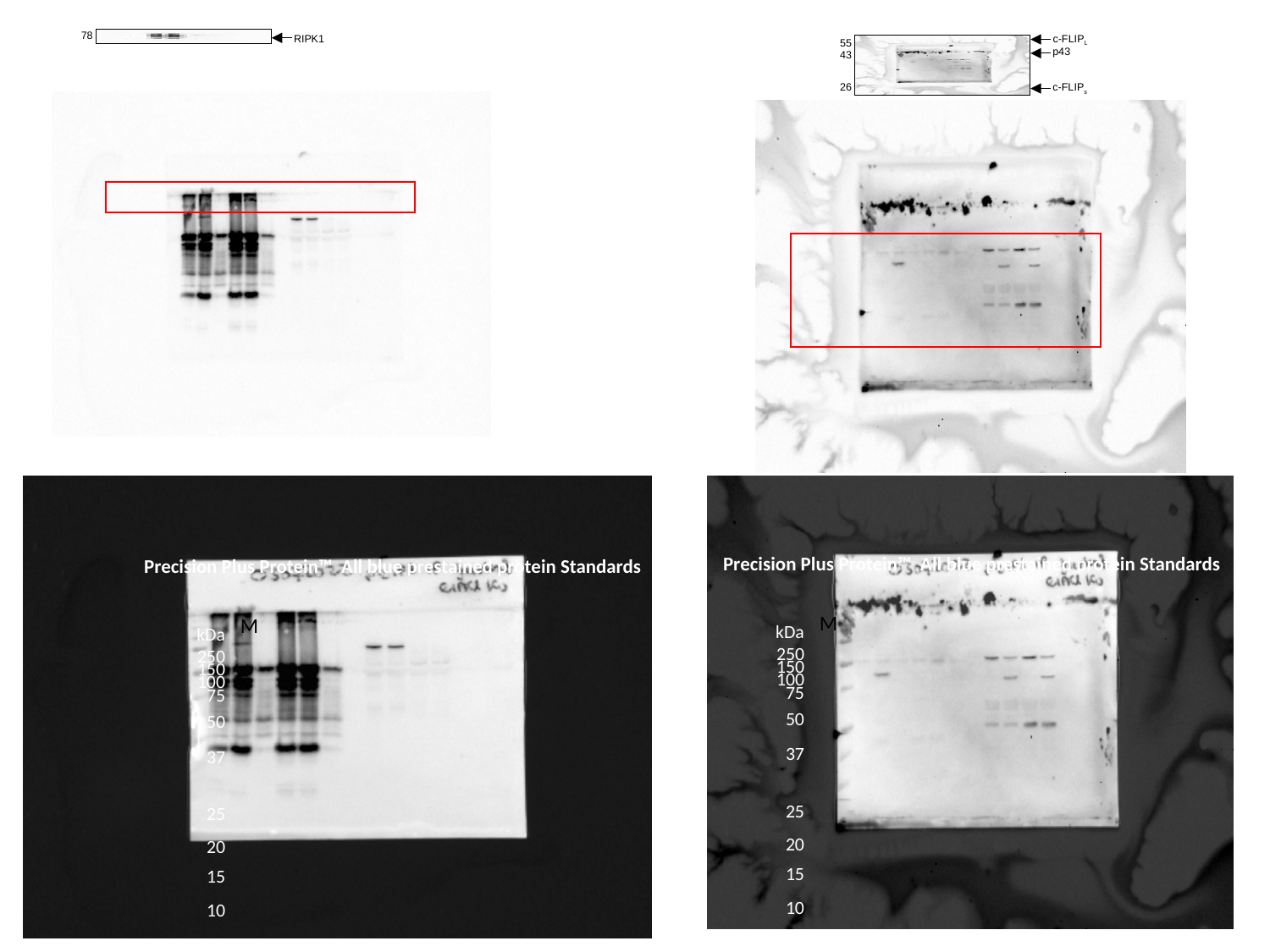

78
RIPK1
c-FLIPL
55
p43
43
26
c-FLIPs
Precision Plus Protein™ All blue prestained protein Standards
Precision Plus Protein™ All blue prestained protein Standards
M
M
kDa
kDa
250
250
150
150
100
100
75
75
50
50
37
37
25
25
20
20
15
15
10
10

## Slide 4
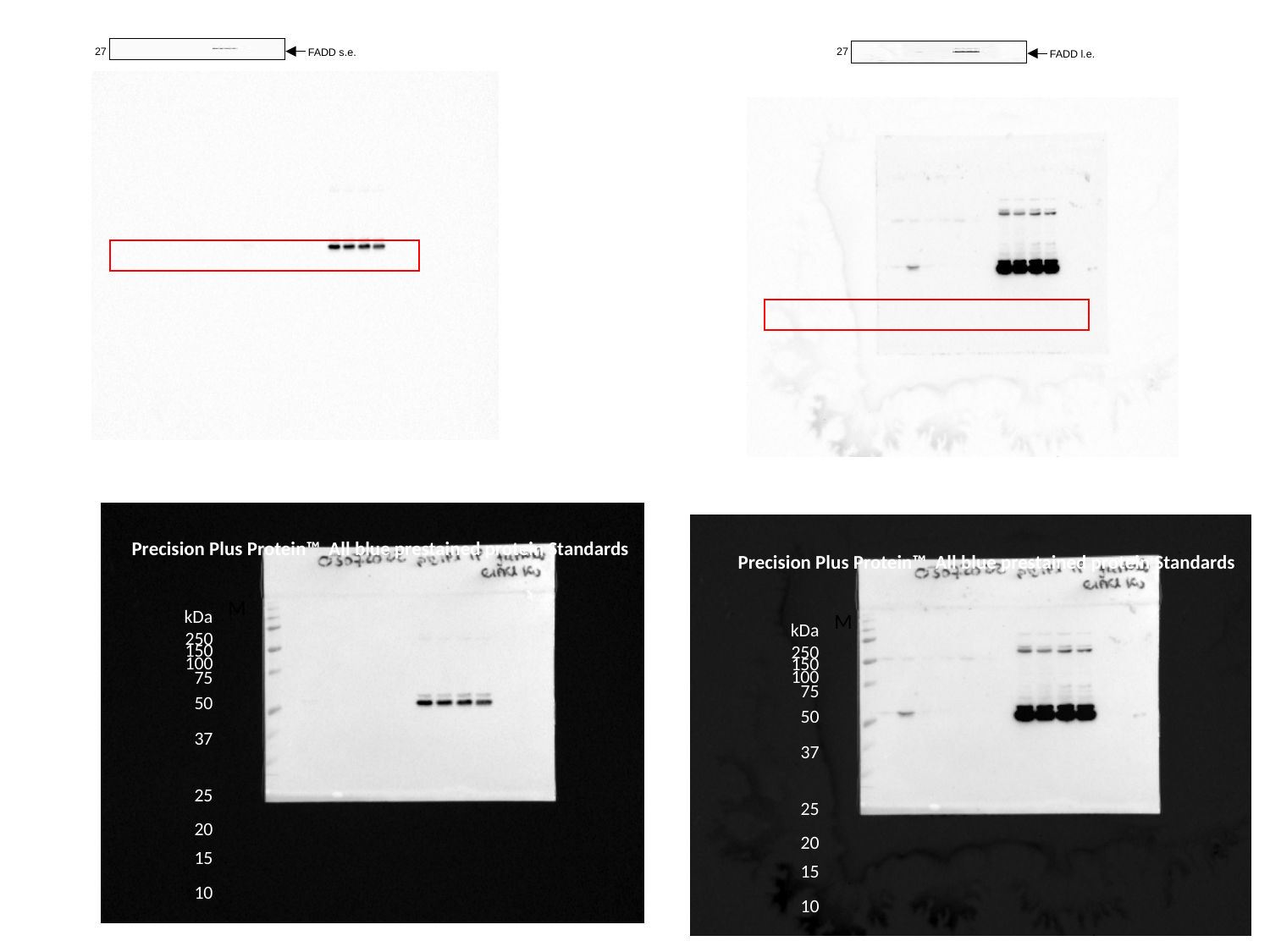

27
27
FADD s.e.
FADD l.e.
Precision Plus Protein™ All blue prestained protein Standards
Precision Plus Protein™ All blue prestained protein Standards
M
kDa
M
kDa
250
150
250
100
150
100
75
75
50
50
37
37
25
25
20
20
15
15
10
10

## Slide 5
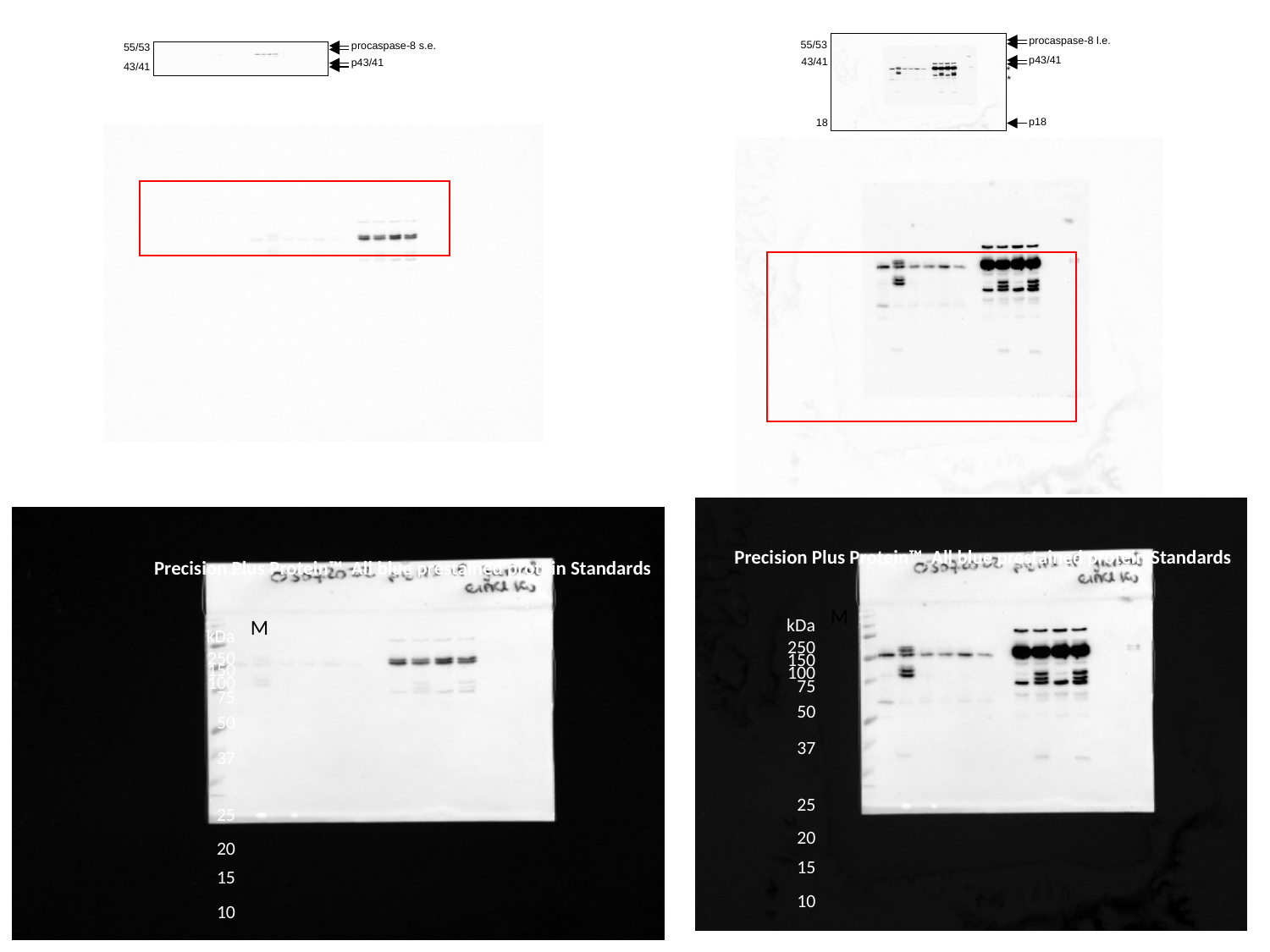

procaspase-8 l.e.
55/53
procaspase-8 s.e.
55/53
p43/41
43/41
p43/41
43/41
*
*
p18
18
Precision Plus Protein™ All blue prestained protein Standards
Precision Plus Protein™ All blue prestained protein Standards
M
kDa
M
kDa
250
250
150
150
100
100
75
75
50
50
37
37
25
25
20
20
15
15
10
10

## Slide 6
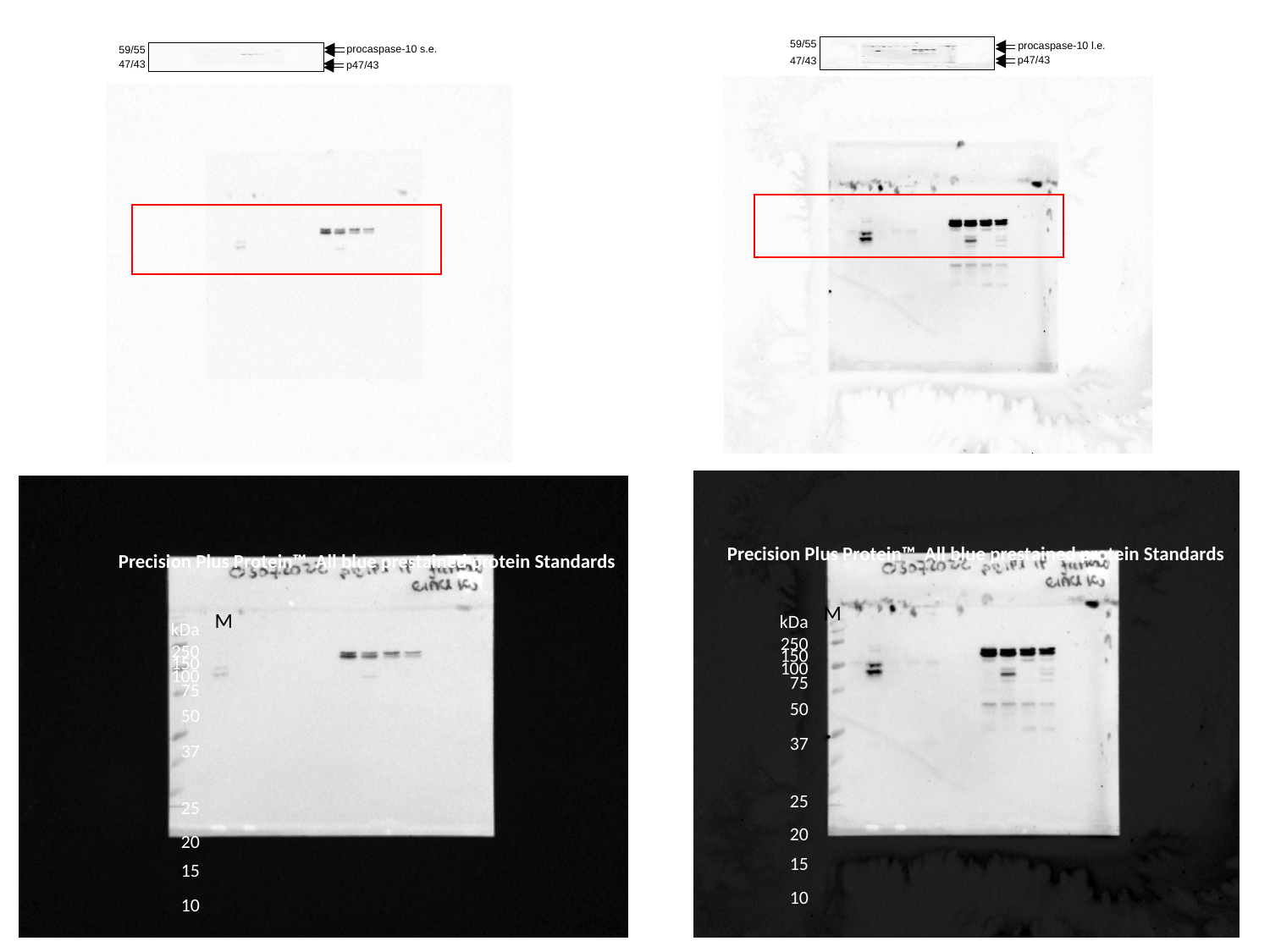

59/55
procaspase-10 l.e.
procaspase-10 s.e.
59/55
p47/43
47/43
47/43
p47/43
Precision Plus Protein™ All blue prestained protein Standards
Precision Plus Protein™ All blue prestained protein Standards
M
M
kDa
kDa
250
250
150
150
100
100
75
75
50
50
37
37
25
25
20
20
15
15
10
10

## Slide 7
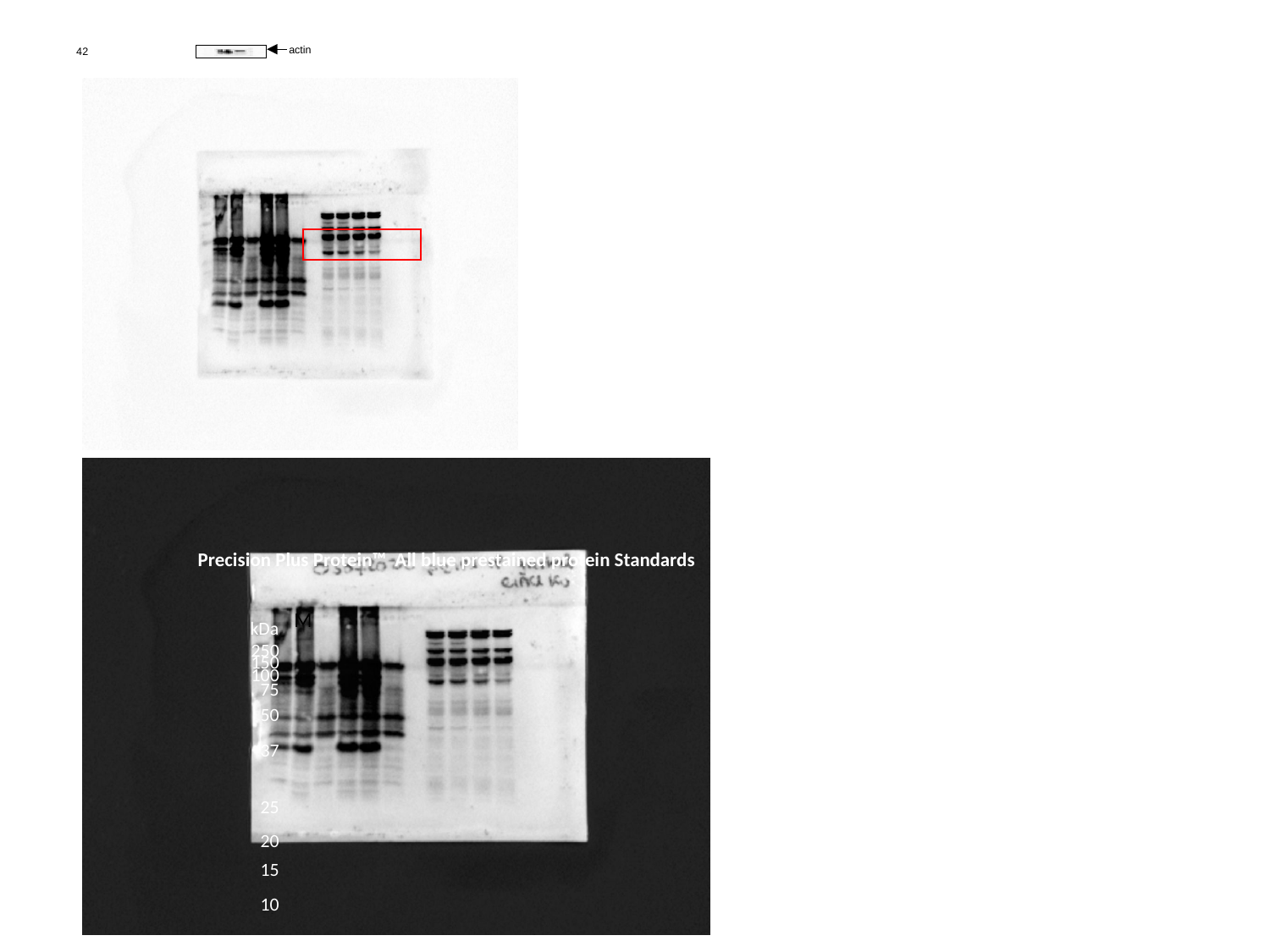

actin
42
Precision Plus Protein™ All blue prestained protein Standards
M
kDa
250
150
100
75
50
37
25
20
15
10
