# Supplementary material for: Oligomerised RIPK1 is the main core component of the CD95 necrosome
Source: EMBO J. 2025 Apr 16;44(11):3231–65. doi: 10.1038/s44318-025-00433-0 (PMC12130296; doi:10.1038/s44318-025-00433-0)
Supplement: Supplementary file 7 — Source data Fig. 3 [file 44318_2025_433_MOESM7_ESM.zip › figure3B.pptx]

## Slide 1
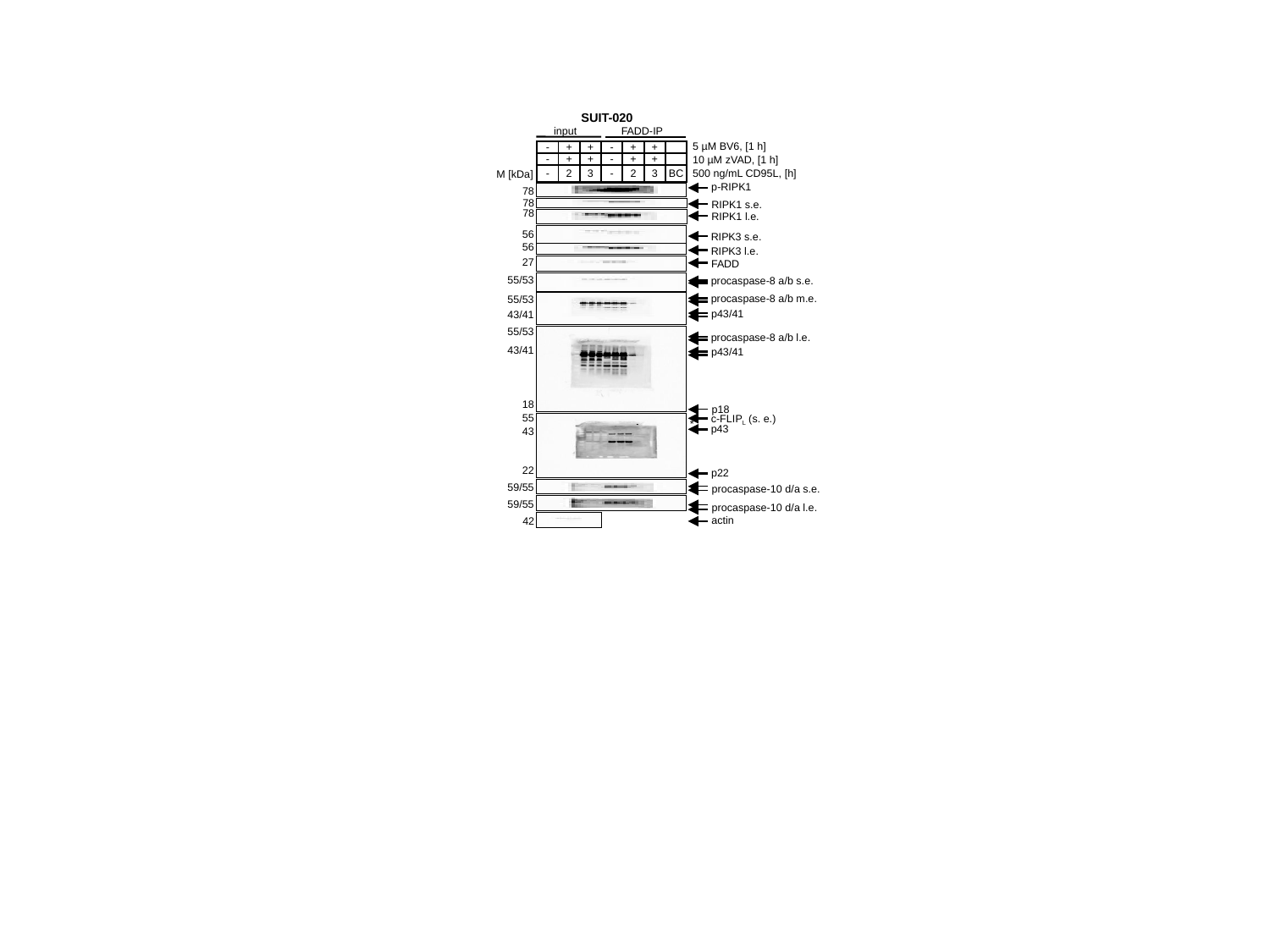

SUIT-020
input
FADD-IP
5 µM BV6, [1 h]
| - | + | + | - | + | + | |
| --- | --- | --- | --- | --- | --- | --- |
| - | + | + | - | + | + | |
| - | 2 | 3 | - | 2 | 3 | BC |
10 µM zVAD, [1 h]
500 ng/mL CD95L, [h]
M [kDa]
p-RIPK1
78
78
RIPK1 s.e.
78
RIPK1 l.e.
56
RIPK3 s.e.
56
RIPK3 l.e.
27
FADD
55/53
procaspase-8 a/b s.e.
procaspase-8 a/b m.e.
55/53
p43/41
43/41
55/53
procaspase-8 a/b l.e.
43/41
p43/41
18
p18
55
c-FLIPL (s. e.)
*
p43
43
22
p22
59/55
procaspase-10 d/a s.e.
59/55
procaspase-10 d/a l.e.
actin
42

## Slide 2
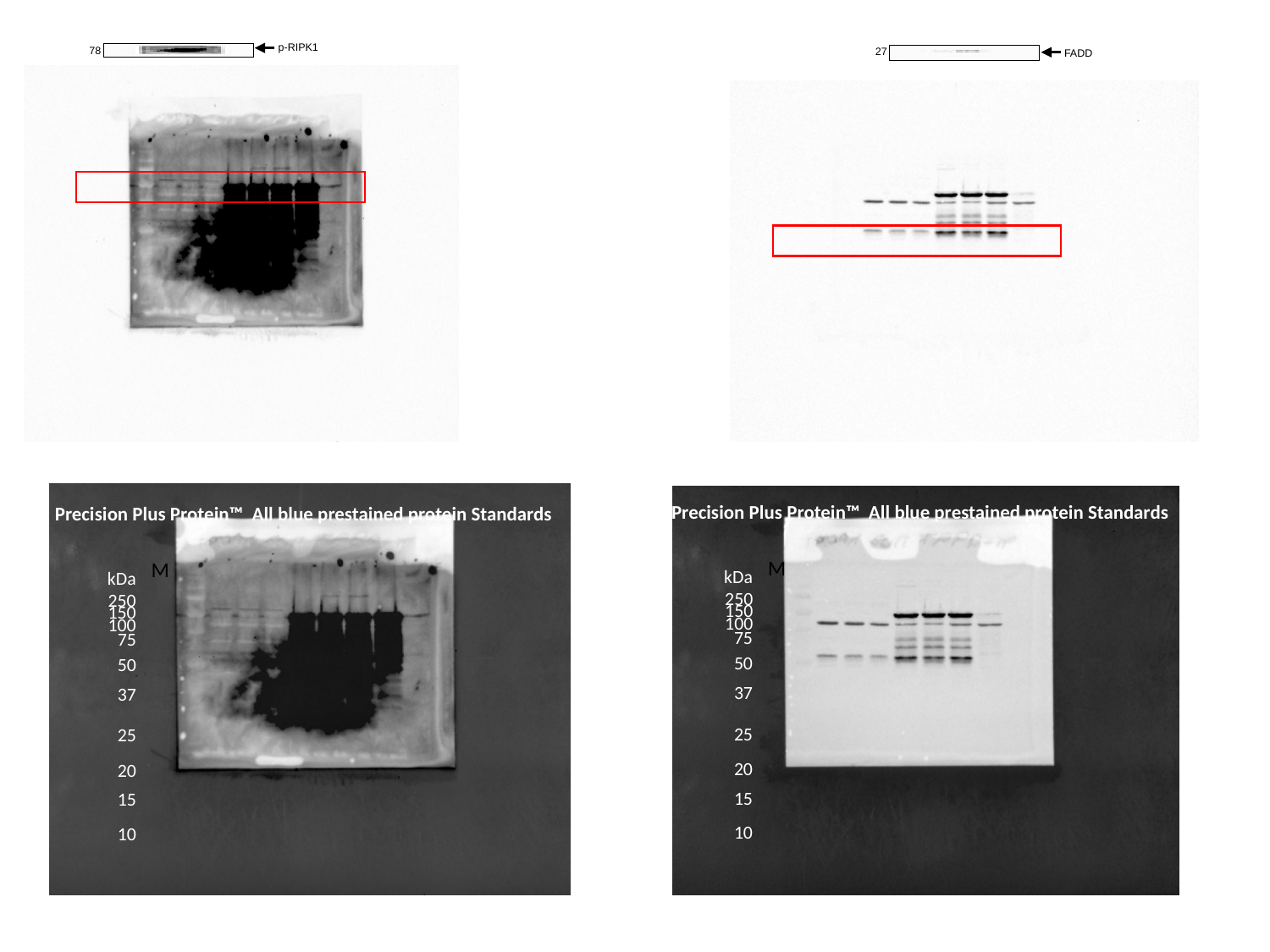

p-RIPK1
78
27
FADD
Precision Plus Protein™ All blue prestained protein Standards
Precision Plus Protein™ All blue prestained protein Standards
M
M
kDa
kDa
250
250
150
150
100
100
75
75
50
50
37
37
25
25
20
20
15
15
10
10

## Slide 3
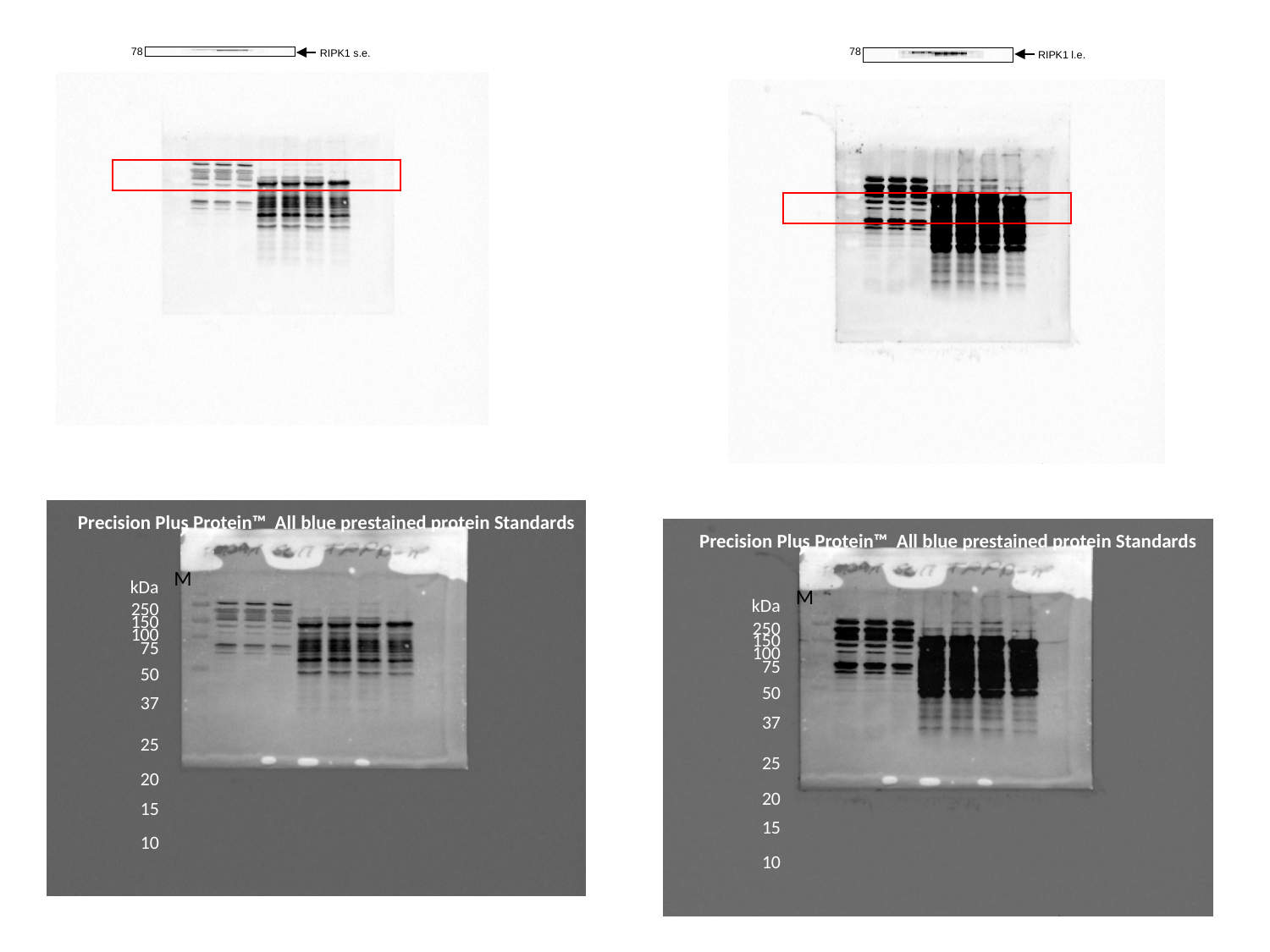

78
78
RIPK1 s.e.
RIPK1 l.e.
Precision Plus Protein™ All blue prestained protein Standards
Precision Plus Protein™ All blue prestained protein Standards
M
kDa
M
kDa
250
150
250
100
150
75
100
75
50
50
37
37
25
25
20
20
15
15
10
10

## Slide 4
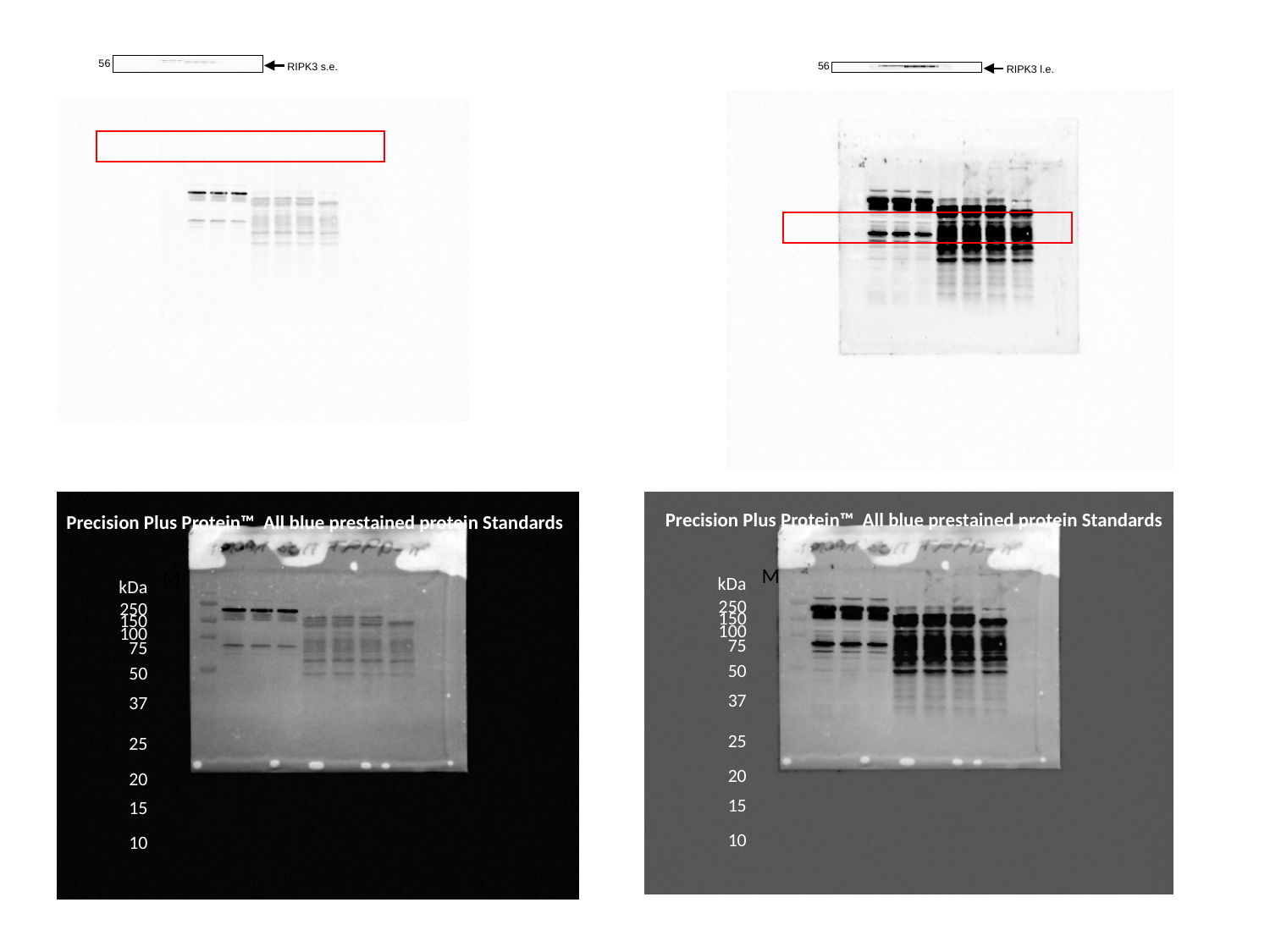

56
56
RIPK3 s.e.
RIPK3 l.e.
Precision Plus Protein™ All blue prestained protein Standards
Precision Plus Protein™ All blue prestained protein Standards
M
M
kDa
kDa
250
250
150
150
100
100
75
75
50
50
37
37
25
25
20
20
15
15
10
10

## Slide 5
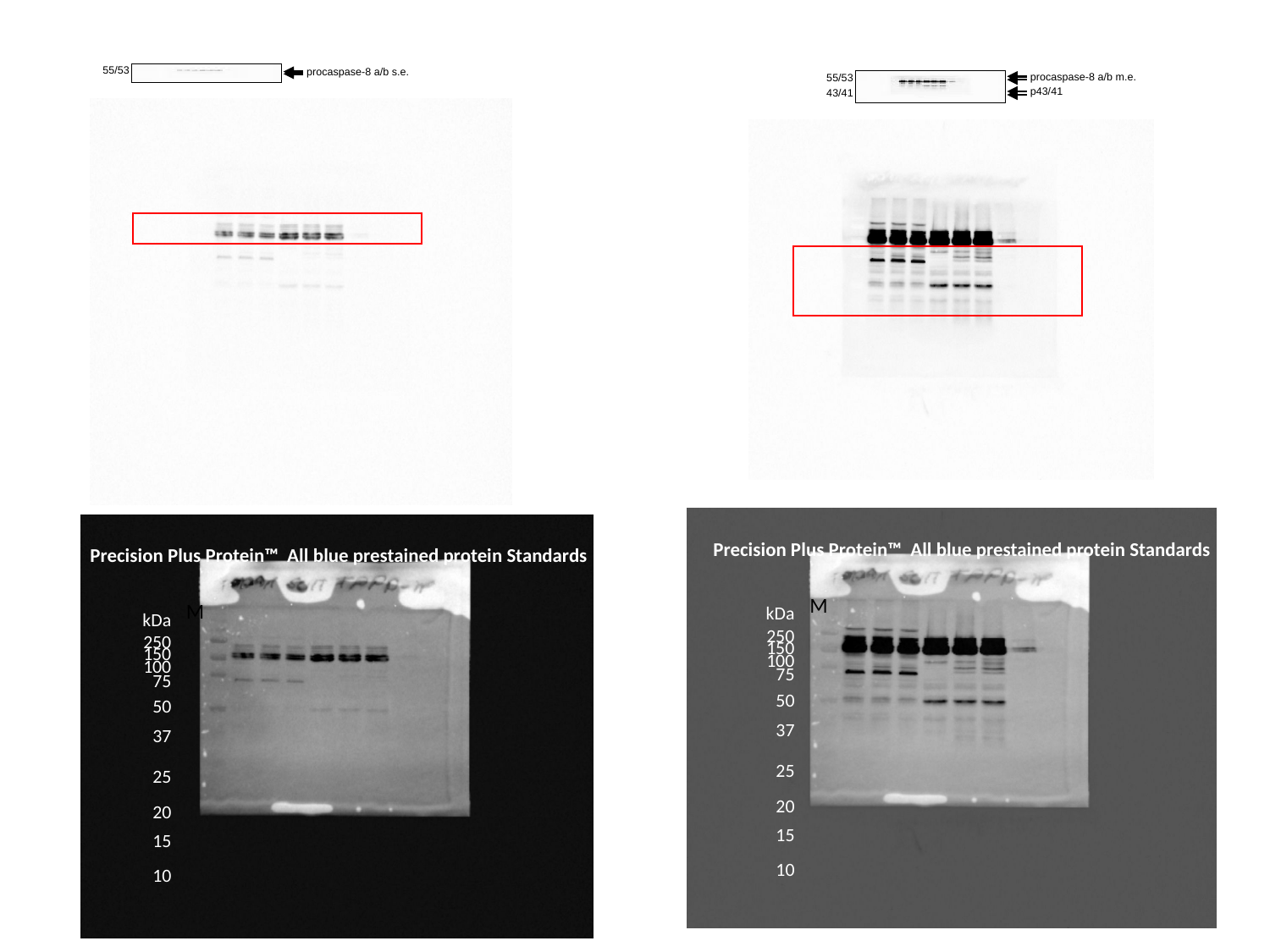

55/53
procaspase-8 a/b s.e.
procaspase-8 a/b m.e.
55/53
p43/41
43/41
Precision Plus Protein™ All blue prestained protein Standards
Precision Plus Protein™ All blue prestained protein Standards
M
M
kDa
kDa
250
250
150
150
100
100
75
75
50
50
37
37
25
25
20
20
15
15
10
10

## Slide 6
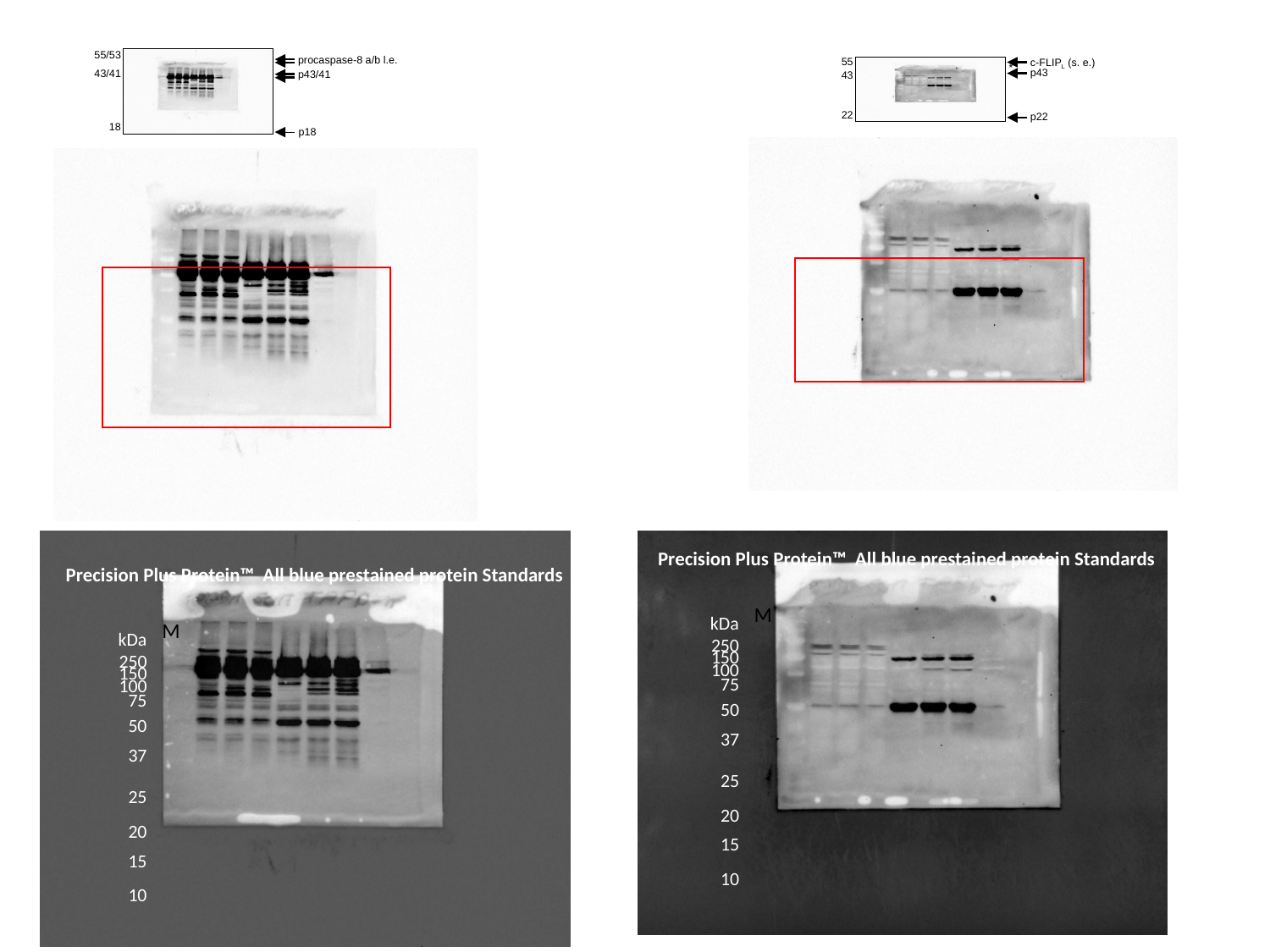

55/53
procaspase-8 a/b l.e.
55
c-FLIPL (s. e.)
*
p43
43/41
p43/41
43
22
p22
18
p18
Precision Plus Protein™ All blue prestained protein Standards
Precision Plus Protein™ All blue prestained protein Standards
M
kDa
M
kDa
250
150
250
100
150
75
100
75
50
50
37
37
25
25
20
20
15
15
10
10

## Slide 7
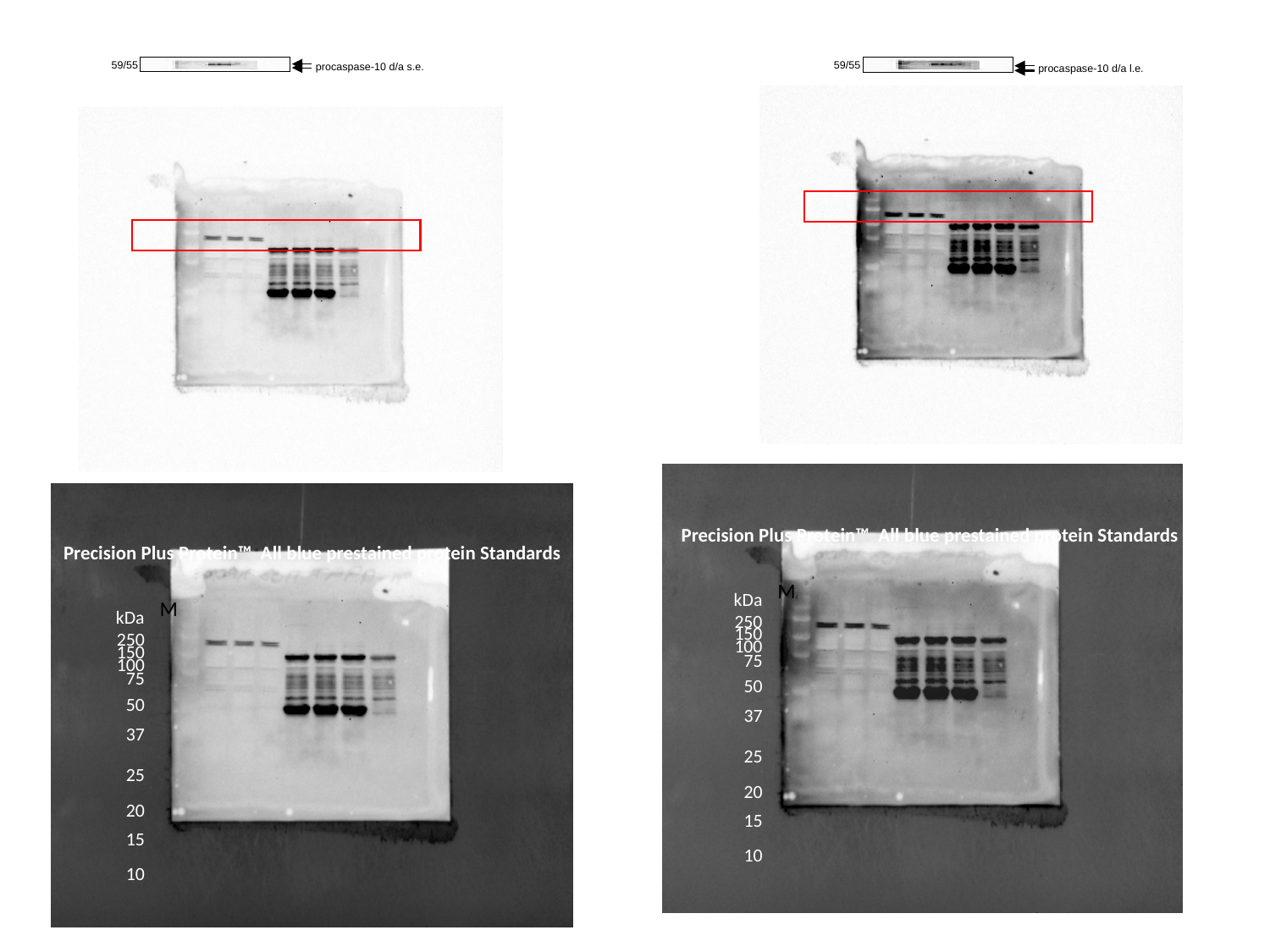

59/55
59/55
procaspase-10 d/a s.e.
procaspase-10 d/a l.e.
Precision Plus Protein™ All blue prestained protein Standards
Precision Plus Protein™ All blue prestained protein Standards
M
kDa
M
kDa
250
150
250
100
150
75
100
75
50
50
37
37
25
25
20
20
15
15
10
10

## Slide 8
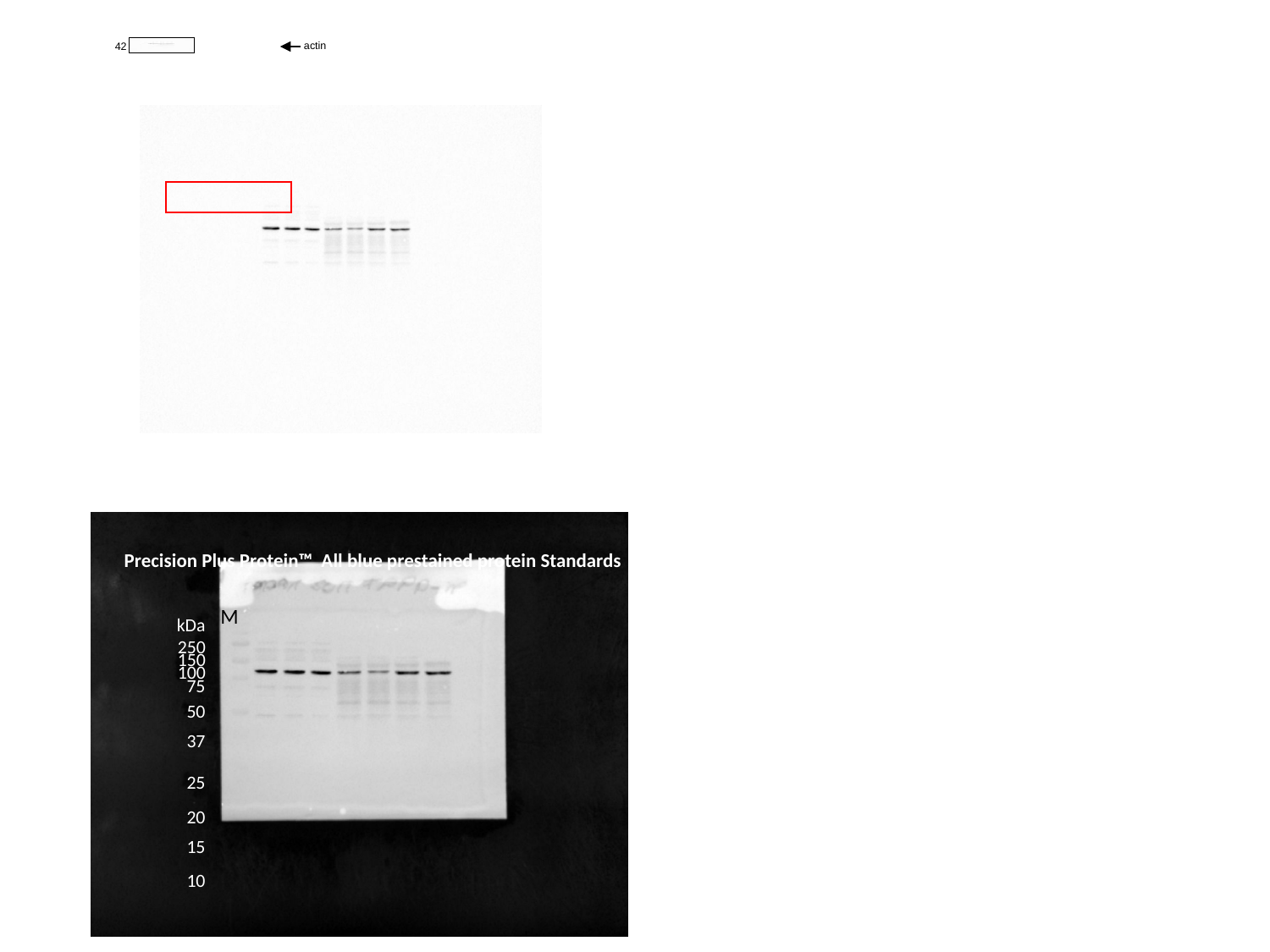

actin
42
Precision Plus Protein™ All blue prestained protein Standards
M
kDa
250
150
100
75
50
37
25
20
15
10
