# Supplementary material for: Oligomerised RIPK1 is the main core component of the CD95 necrosome
Source: EMBO J. 2025 Apr 16;44(11):3231–65. doi: 10.1038/s44318-025-00433-0 (PMC12130296; doi:10.1038/s44318-025-00433-0)
Supplement: Supplementary file 7 — Source data Fig. 3 [file 44318_2025_433_MOESM7_ESM.zip › figure3C.pptx]

## Slide 1
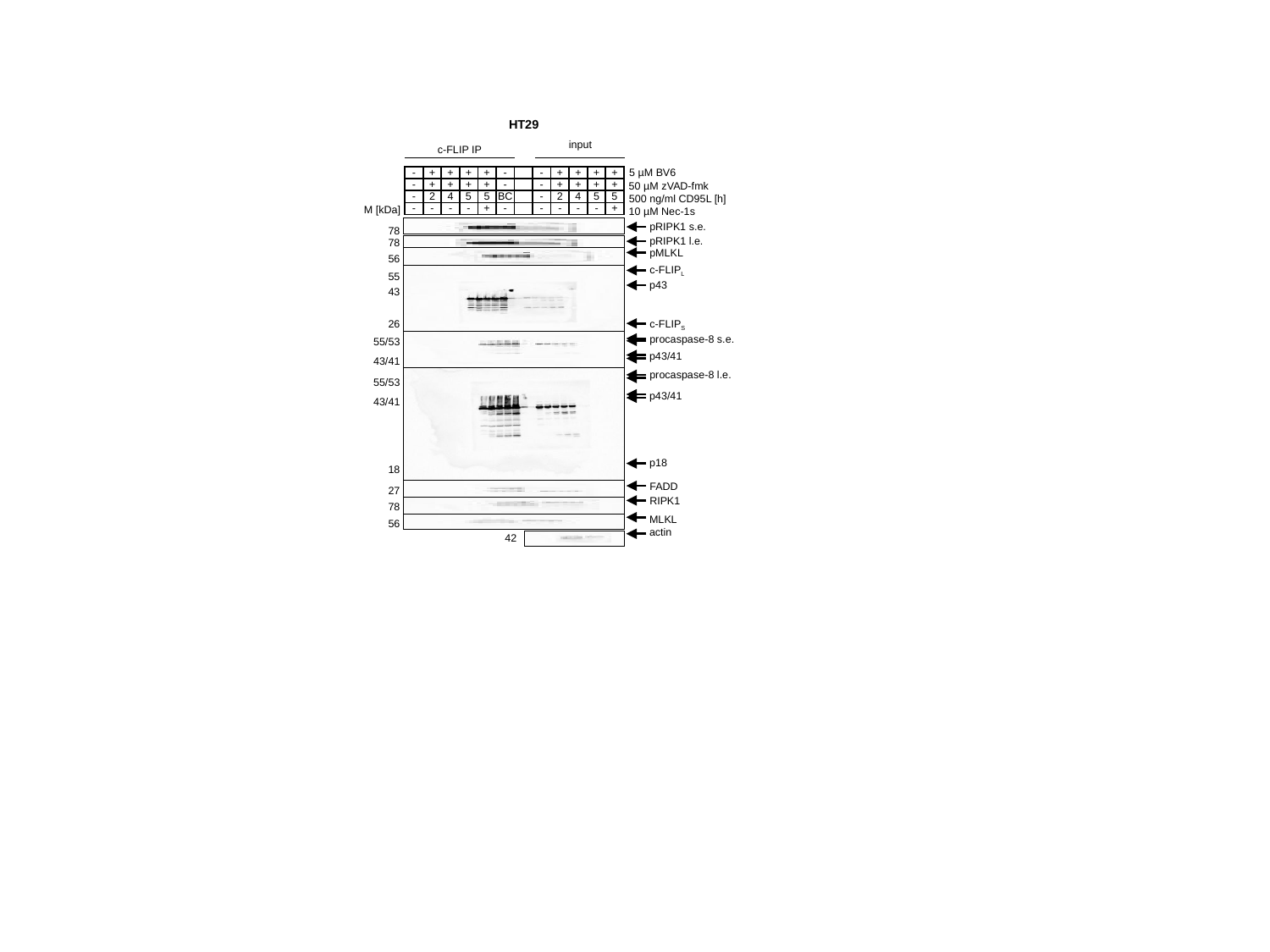

HT29
input
c-FLIP IP
5 µM BV6
| - | + | + | + | + | - | | - | + | + | + | + |
| --- | --- | --- | --- | --- | --- | --- | --- | --- | --- | --- | --- |
| - | + | + | + | + | - | | - | + | + | + | + |
| - | 2 | 4 | 5 | 5 | BC | | - | 2 | 4 | 5 | 5 |
| - | - | - | - | + | - | | - | - | - | - | + |
50 µM zVAD-fmk
500 ng/ml CD95L [h]
M [kDa]
10 µM Nec-1s
pRIPK1 s.e.
78
pRIPK1 l.e.
78
pMLKL
56
c-FLIPL
55
p43
43
c-FLIPS
26
procaspase-8 s.e.
55/53
p43/41
43/41
procaspase-8 l.e.
55/53
p43/41
43/41
p18
18
FADD
27
RIPK1
78
MLKL
56
actin
42

## Slide 2
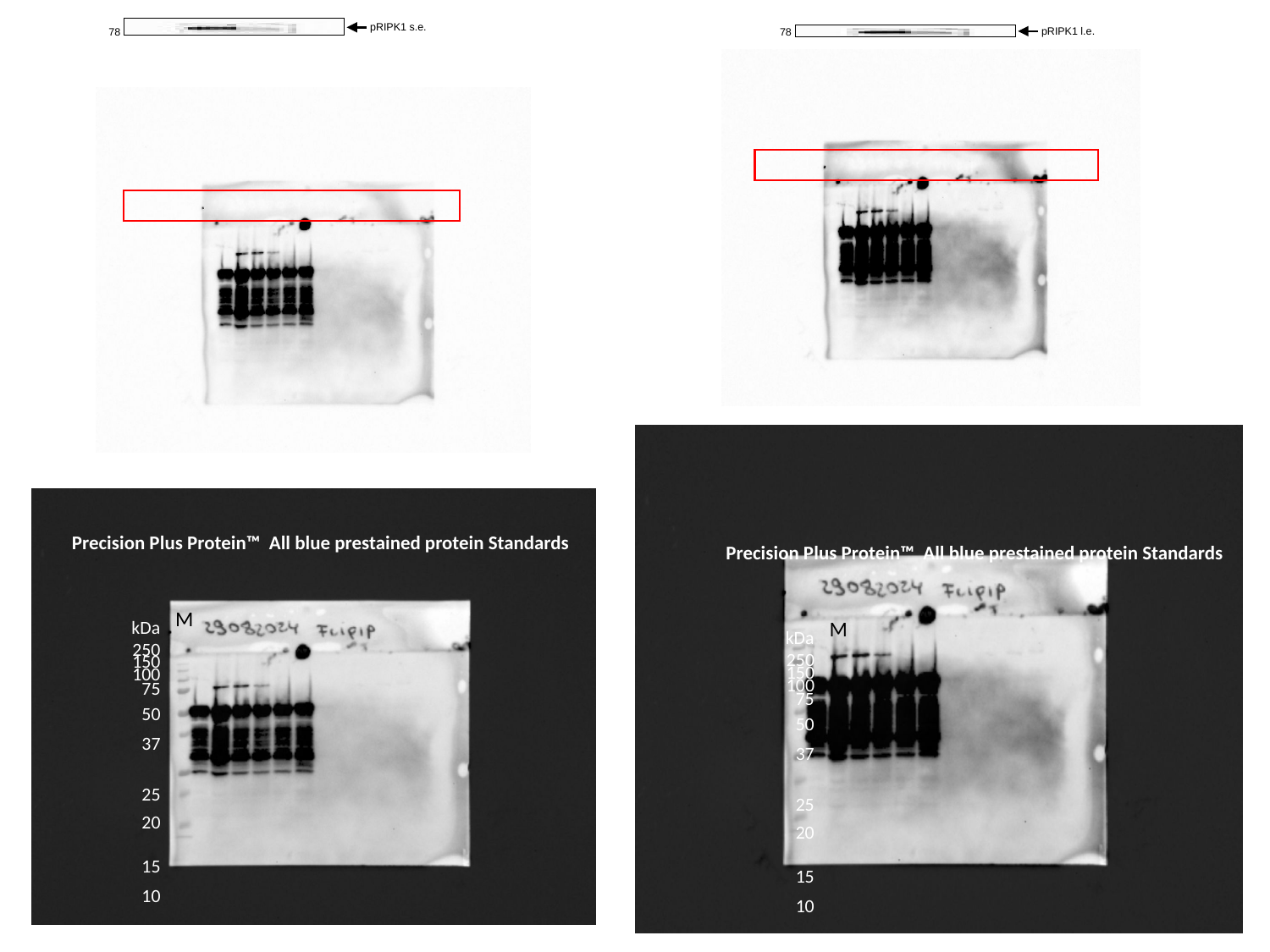

pRIPK1 s.e.
pRIPK1 l.e.
78
78
Precision Plus Protein™ All blue prestained protein Standards
Precision Plus Protein™ All blue prestained protein Standards
M
kDa
M
kDa
250
250
150
150
100
100
75
75
50
50
37
37
25
25
20
20
15
15
10
10

## Slide 3
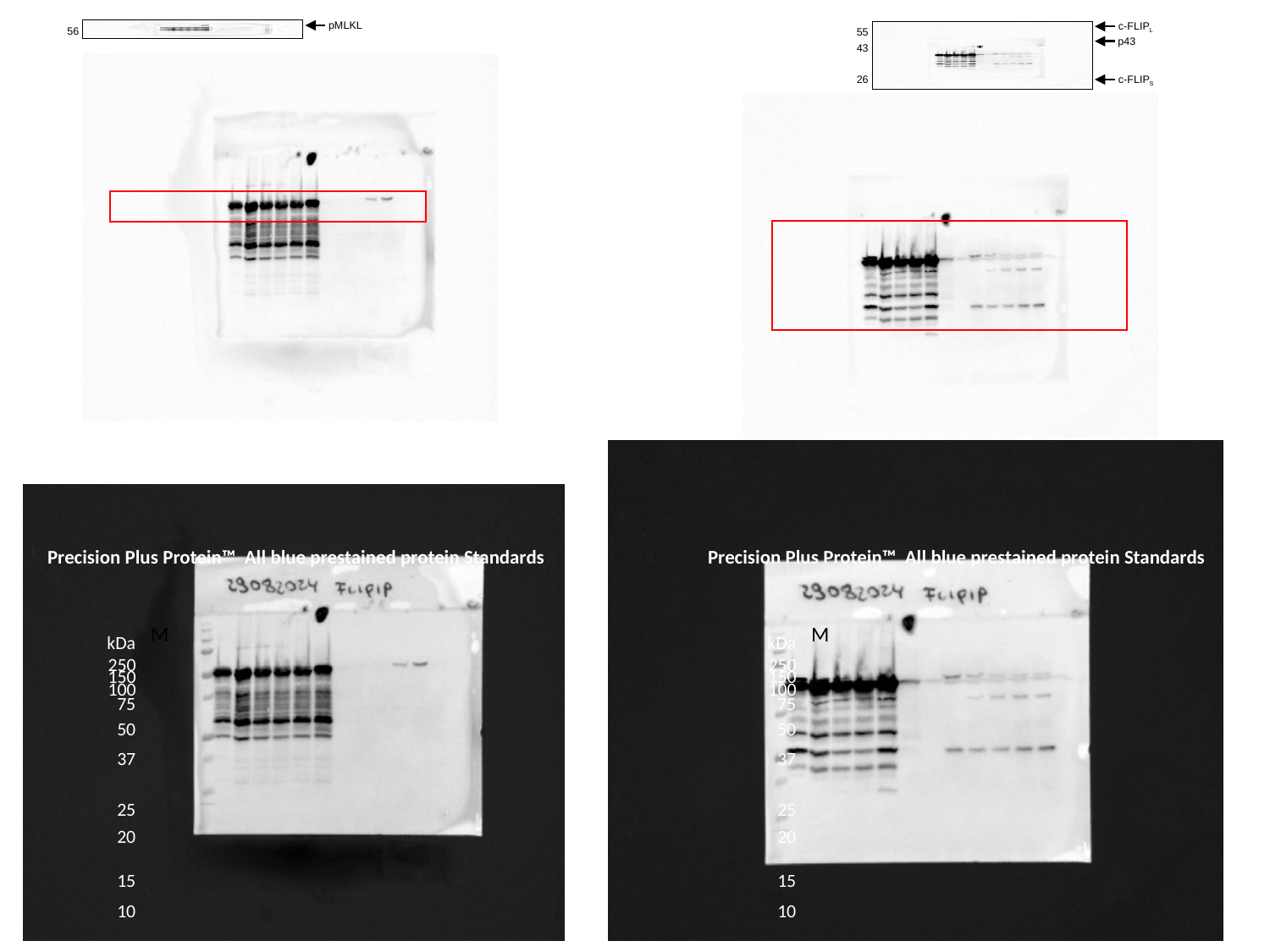

pMLKL
c-FLIPL
56
55
p43
43
c-FLIPS
26
Precision Plus Protein™ All blue prestained protein Standards
Precision Plus Protein™ All blue prestained protein Standards
M
M
kDa
kDa
250
250
150
150
100
100
75
75
50
50
37
37
25
25
20
20
15
15
10
10

## Slide 4
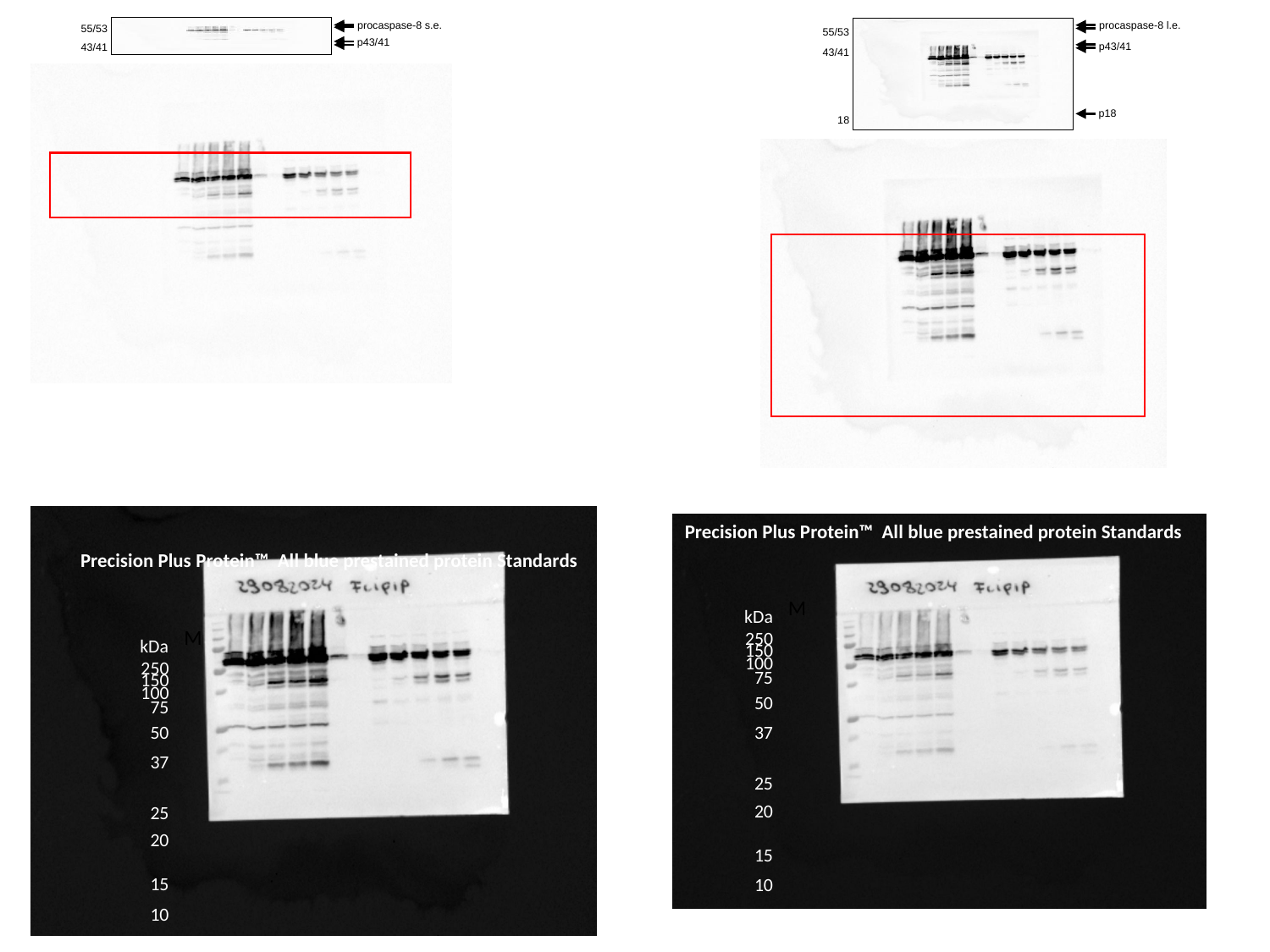

procaspase-8 l.e.
procaspase-8 s.e.
55/53
55/53
p43/41
p43/41
43/41
43/41
p18
18
Precision Plus Protein™ All blue prestained protein Standards
Precision Plus Protein™ All blue prestained protein Standards
M
kDa
M
250
kDa
150
100
250
75
150
100
50
75
50
37
37
25
20
25
20
15
15
10
10

## Slide 5
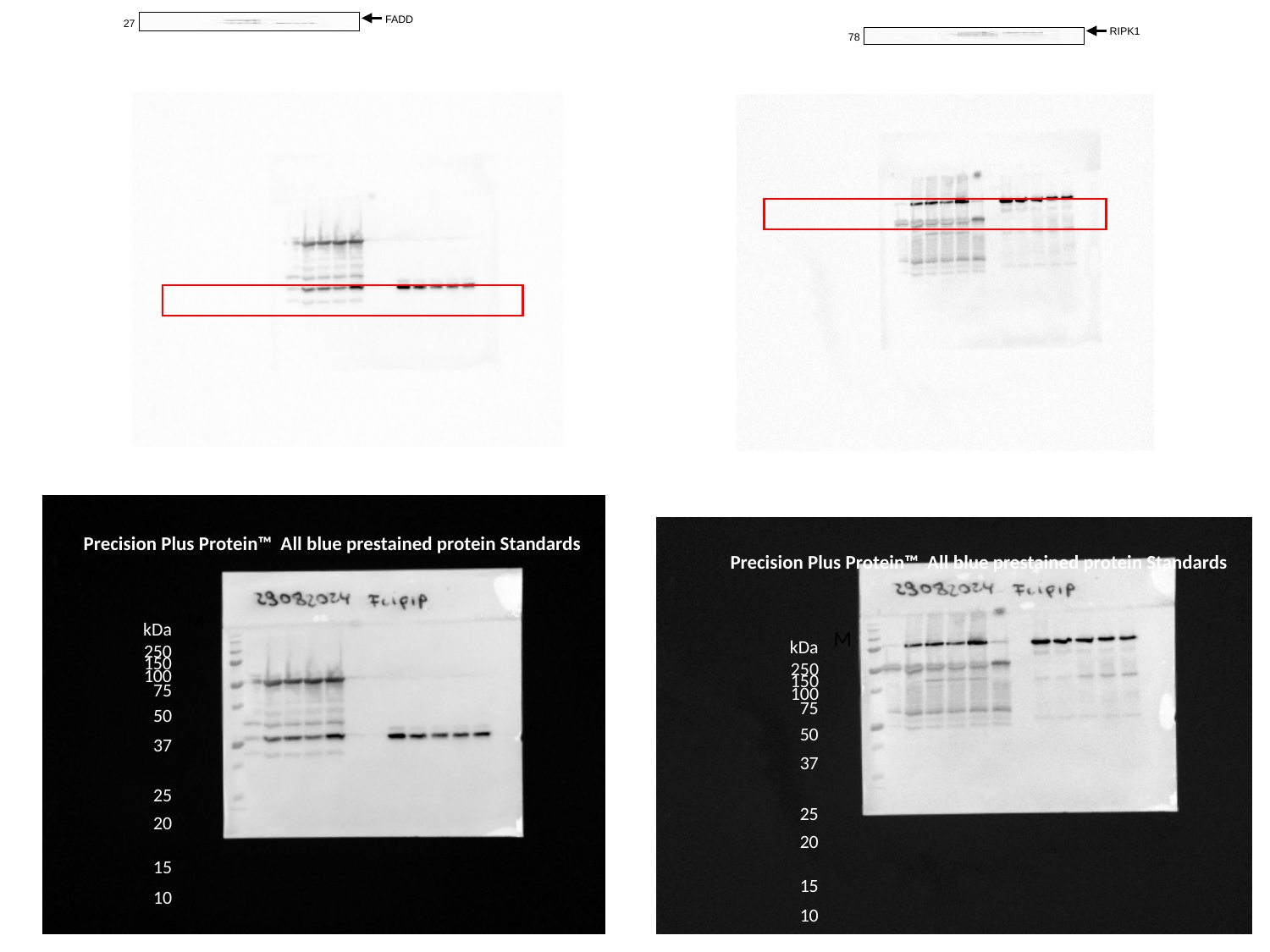

FADD
27
RIPK1
78
Precision Plus Protein™ All blue prestained protein Standards
Precision Plus Protein™ All blue prestained protein Standards
M
kDa
M
kDa
250
150
250
100
150
75
100
75
50
50
37
37
25
25
20
20
15
15
10
10

## Slide 6
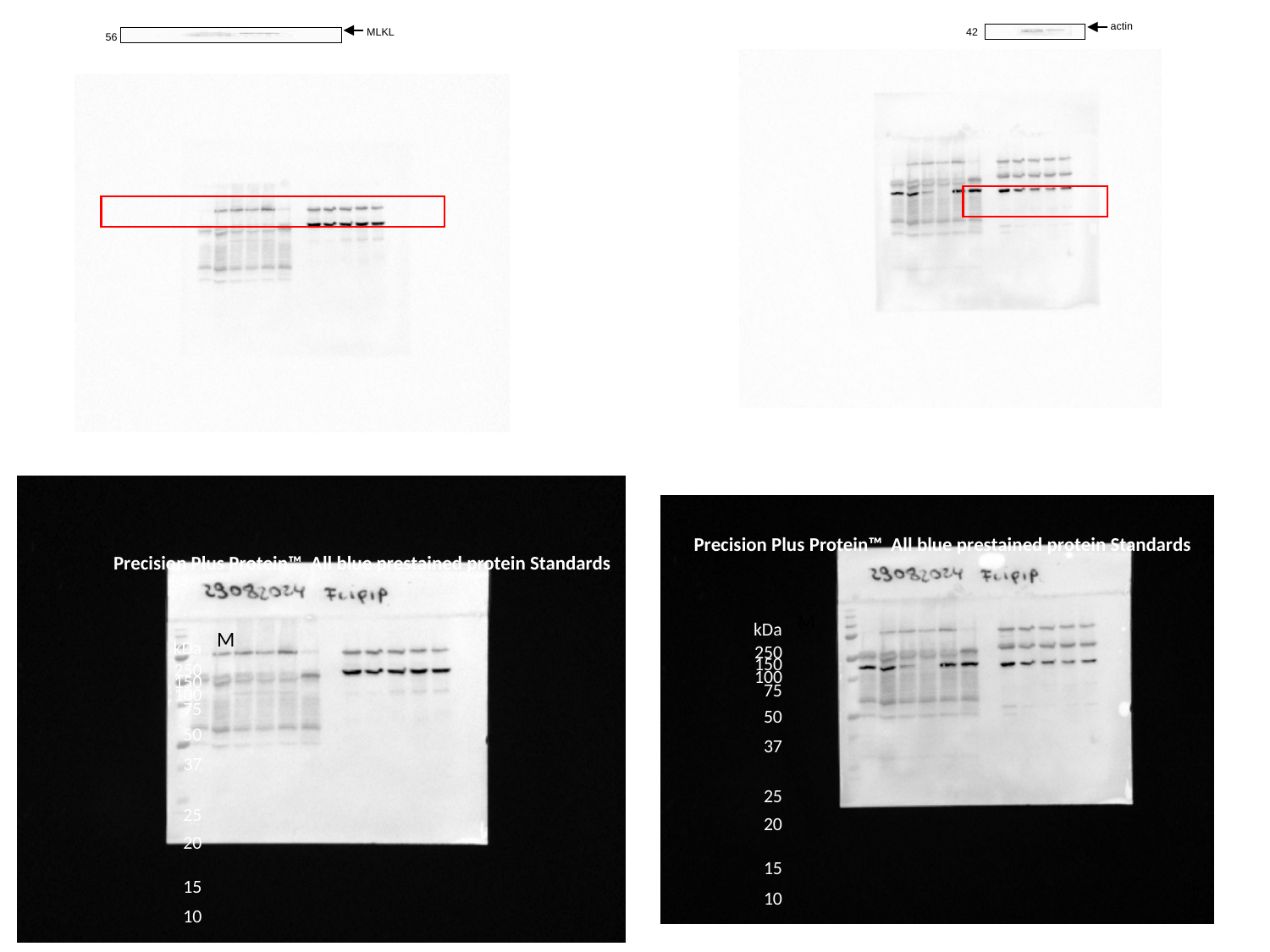

actin
42
MLKL
56
Precision Plus Protein™ All blue prestained protein Standards
Precision Plus Protein™ All blue prestained protein Standards
M
kDa
M
kDa
250
150
250
100
150
75
100
75
50
50
37
37
25
25
20
20
15
15
10
10
