# Supplementary material for: Oligomerised RIPK1 is the main core component of the CD95 necrosome
Source: EMBO J. 2025 Apr 16;44(11):3231–65. doi: 10.1038/s44318-025-00433-0 (PMC12130296; doi:10.1038/s44318-025-00433-0)
Supplement: Supplementary file 7 — Source data Fig. 3 [file 44318_2025_433_MOESM7_ESM.zip › figure3D.pptx]

## Slide 1
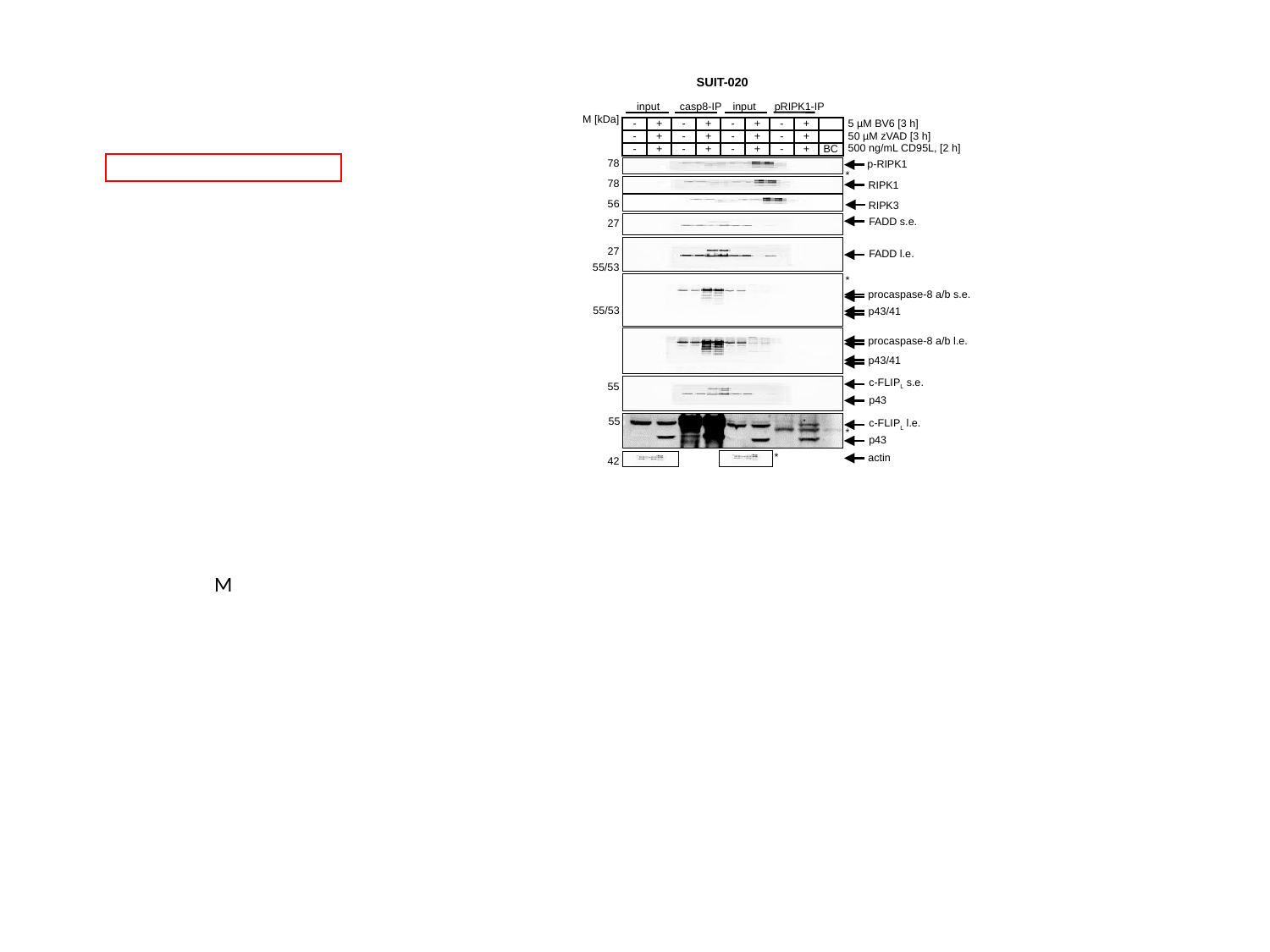

SUIT-020
input
casp8-IP
input
pRIPK1-IP
M [kDa]
5 µM BV6 [3 h]
| - | + | - | + | - | + | - | + | |
| --- | --- | --- | --- | --- | --- | --- | --- | --- |
| - | + | - | + | - | + | - | + | |
| - | + | - | + | - | + | - | + | BC |
50 µM zVAD [3 h]
500 ng/mL CD95L, [2 h]
78
p-RIPK1
*
78
RIPK1
56
RIPK3
FADD s.e.
27
27
FADD l.e.
55/53
*
procaspase-8 a/b s.e.
55/53
p43/41
procaspase-8 a/b l.e.
p43/41
c-FLIPL s.e.
55
p43
55
c-FLIPL l.e.
*
p43
*
actin
42
Precision Plus Protein™ All blue prestained protein Standards
M
kDa
250
150
100
75
50
37
25
20
15
10

## Slide 2
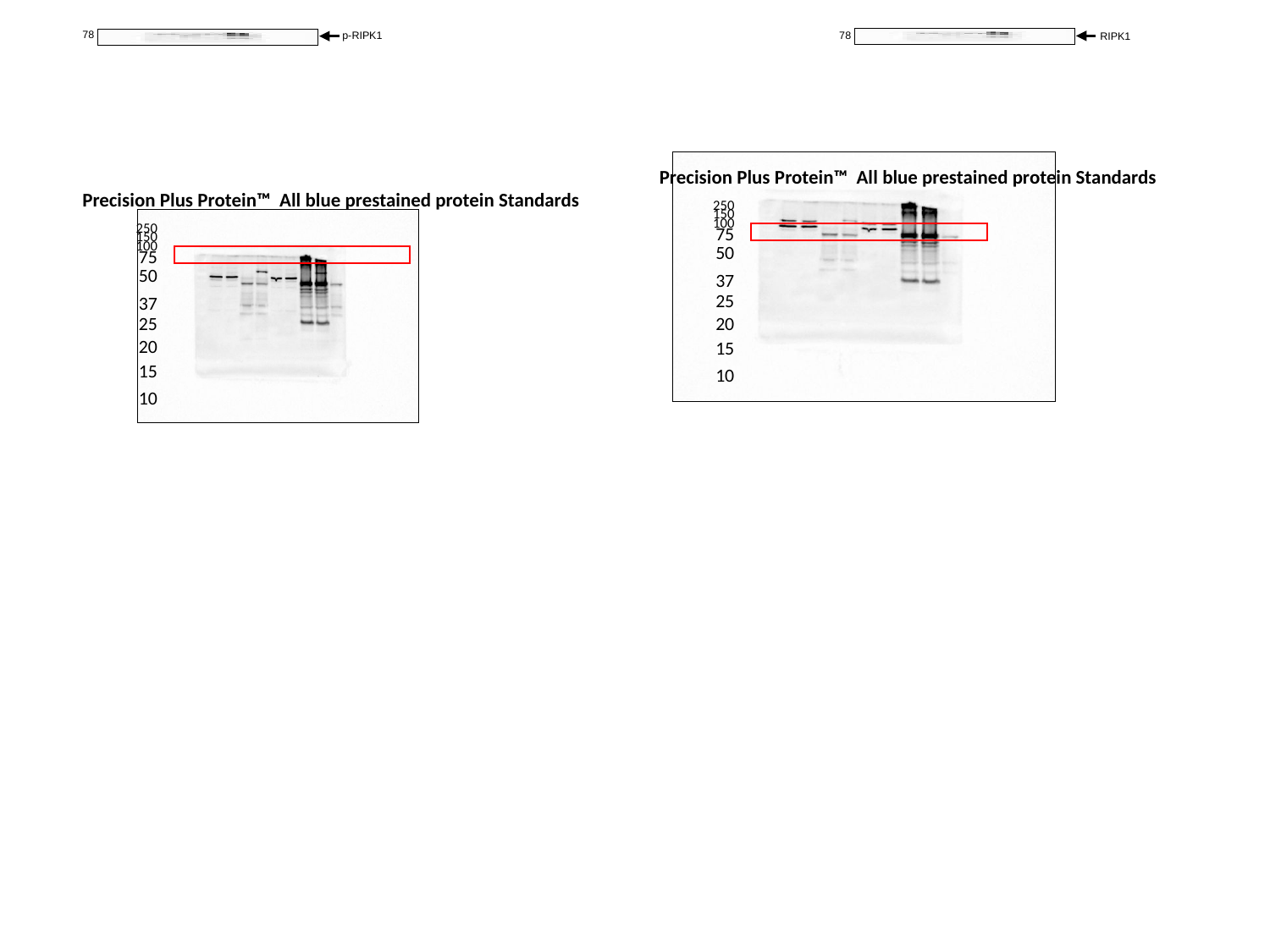

78
78
p-RIPK1
RIPK1
Precision Plus Protein™ All blue prestained protein Standards
Precision Plus Protein™ All blue prestained protein Standards
250
150
100
250
75
150
100
50
75
50
37
25
37
25
20
20
15
15
10
10

## Slide 3
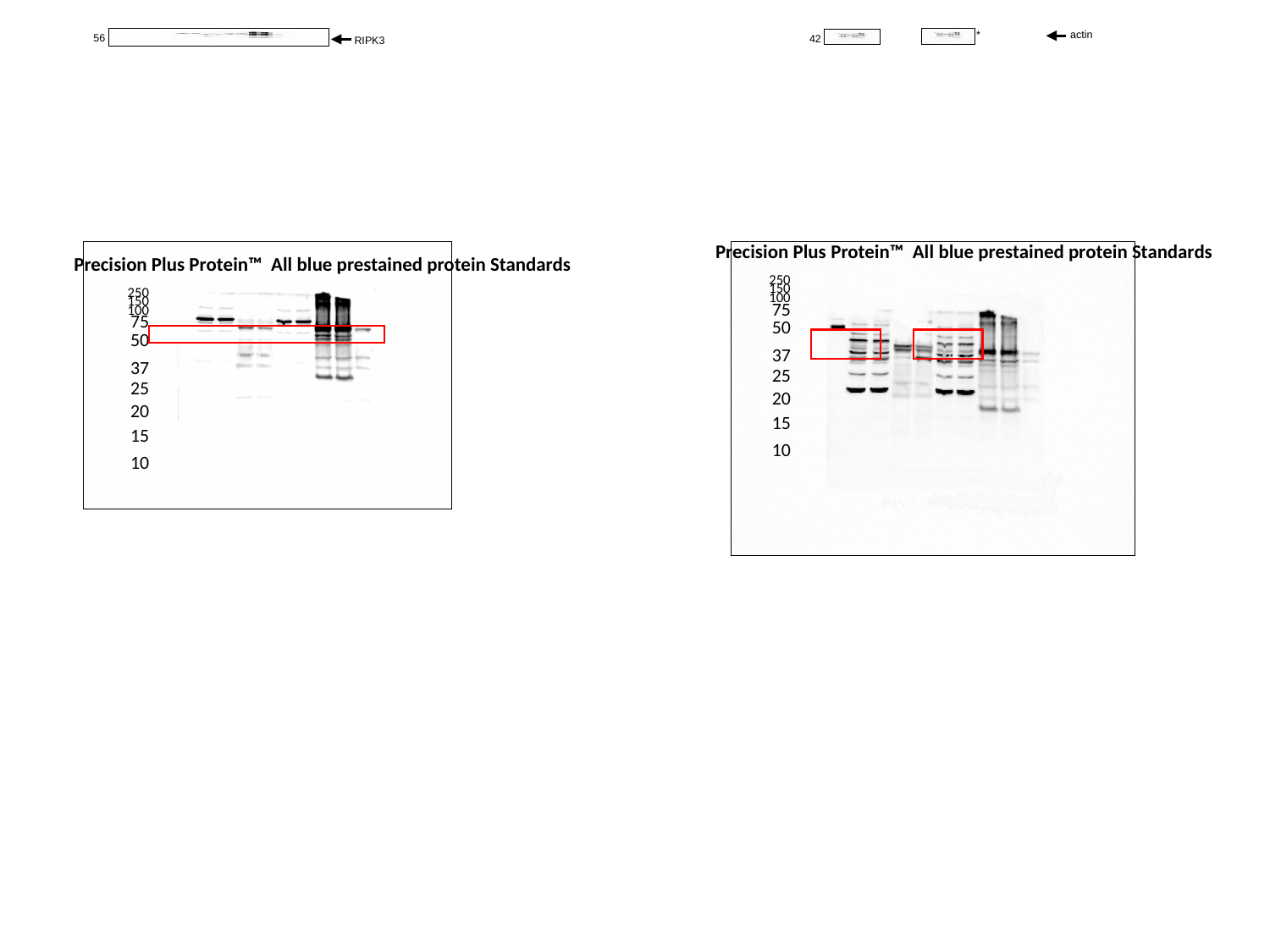

*
actin
56
42
RIPK3
Precision Plus Protein™ All blue prestained protein Standards
Precision Plus Protein™ All blue prestained protein Standards
250
150
250
100
150
75
100
75
50
50
37
37
25
25
20
20
15
15
10
10

## Slide 4
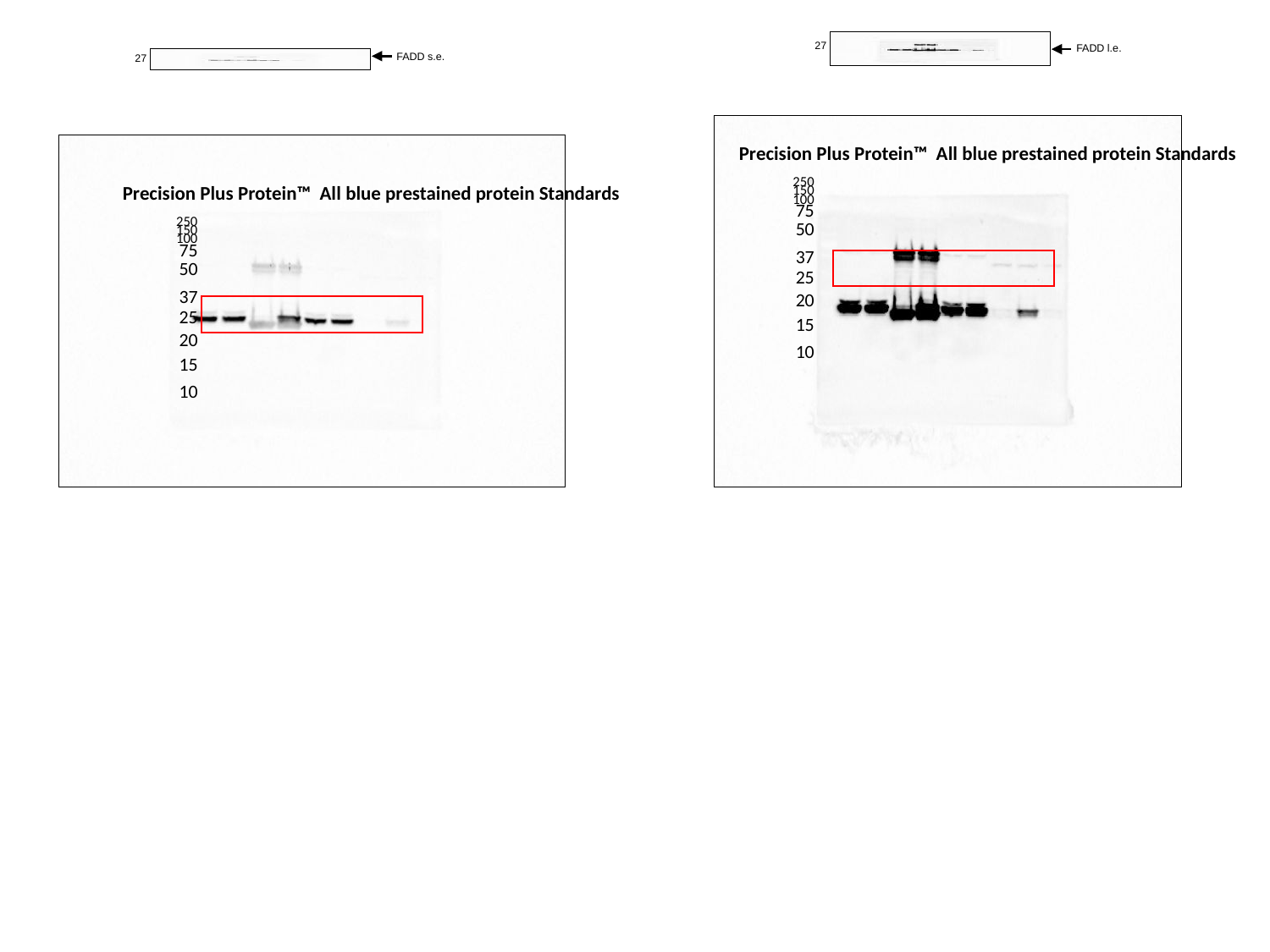

27
FADD l.e.
FADD s.e.
27
Precision Plus Protein™ All blue prestained protein Standards
250
Precision Plus Protein™ All blue prestained protein Standards
150
100
75
250
50
150
100
75
37
50
25
37
20
25
15
20
10
15
10

## Slide 5
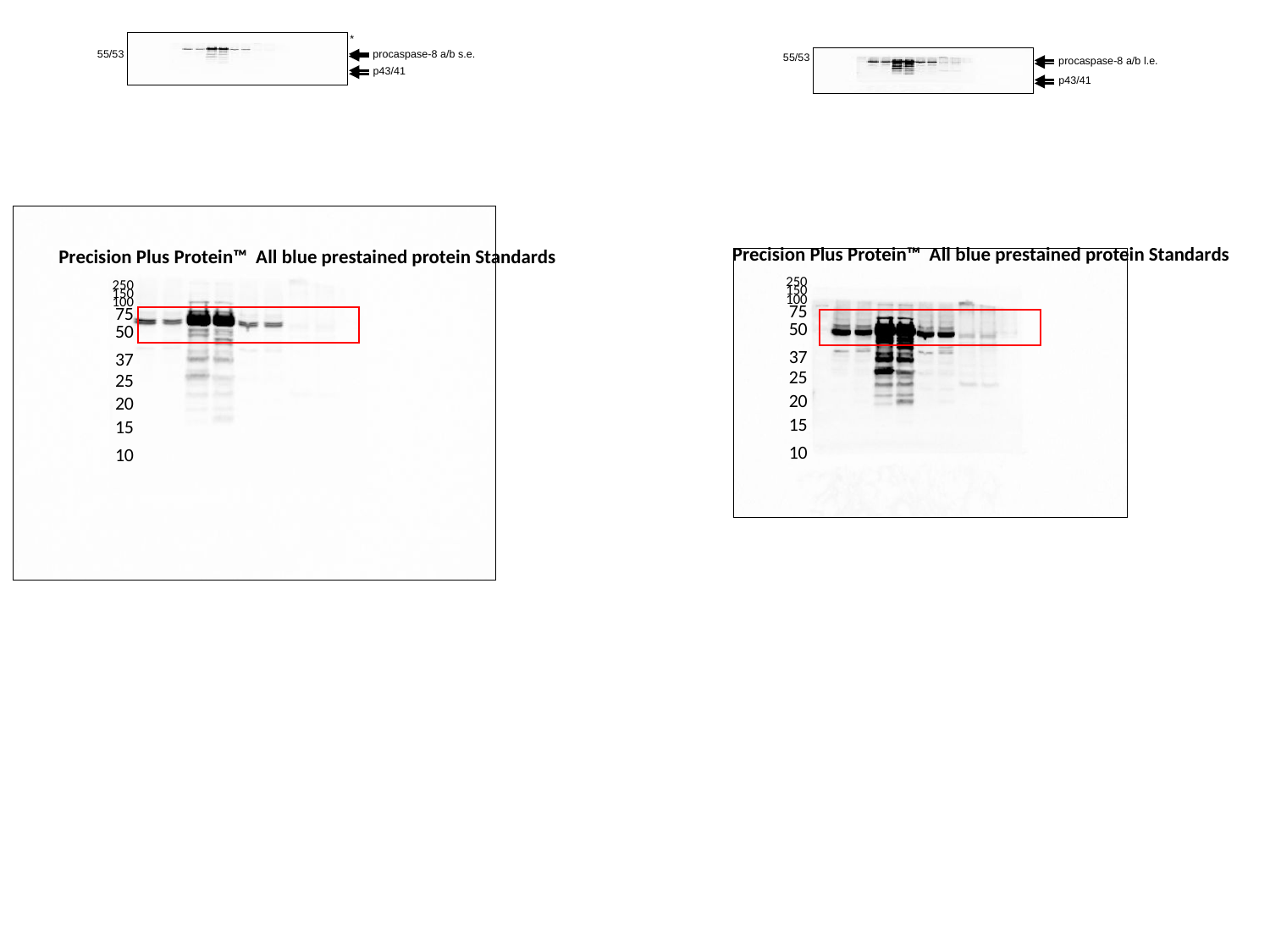

*
procaspase-8 a/b s.e.
55/53
55/53
procaspase-8 a/b l.e.
p43/41
p43/41
Precision Plus Protein™ All blue prestained protein Standards
Precision Plus Protein™ All blue prestained protein Standards
250
250
150
150
100
100
75
75
50
50
37
37
25
25
20
20
15
15
10
10

## Slide 6
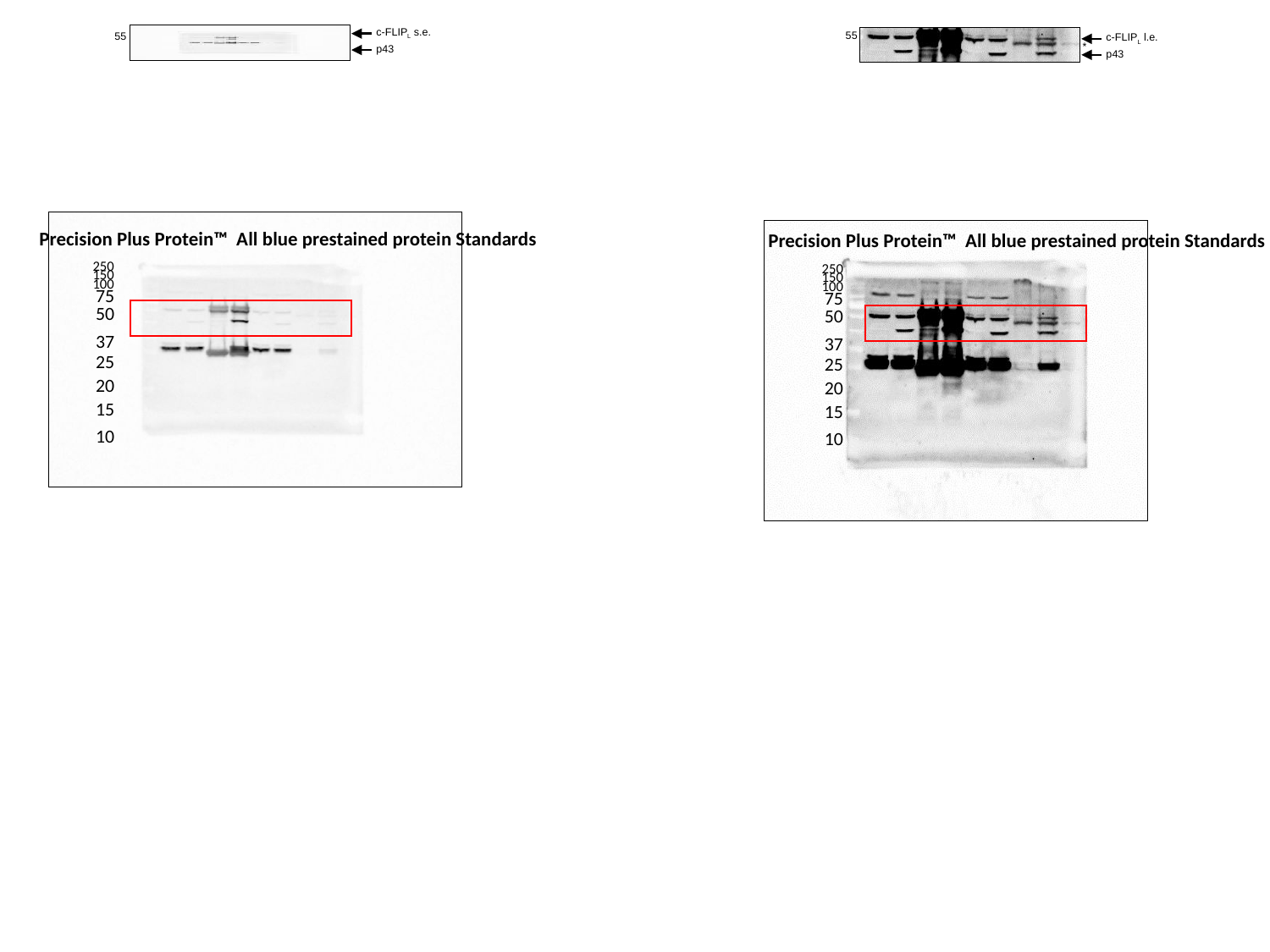

c-FLIPL s.e.
55
55
c-FLIPL l.e.
*
p43
p43
Precision Plus Protein™ All blue prestained protein Standards
Precision Plus Protein™ All blue prestained protein Standards
250
250
150
150
100
100
75
75
50
50
37
37
25
25
20
20
15
15
10
10
