# Supplementary material for: Oligomerised RIPK1 is the main core component of the CD95 necrosome
Source: EMBO J. 2025 Apr 16;44(11):3231–65. doi: 10.1038/s44318-025-00433-0 (PMC12130296; doi:10.1038/s44318-025-00433-0)
Supplement: Supplementary file 7 — Source data Fig. 3 [file 44318_2025_433_MOESM7_ESM.zip › figure3E.pptx]

## Slide 1
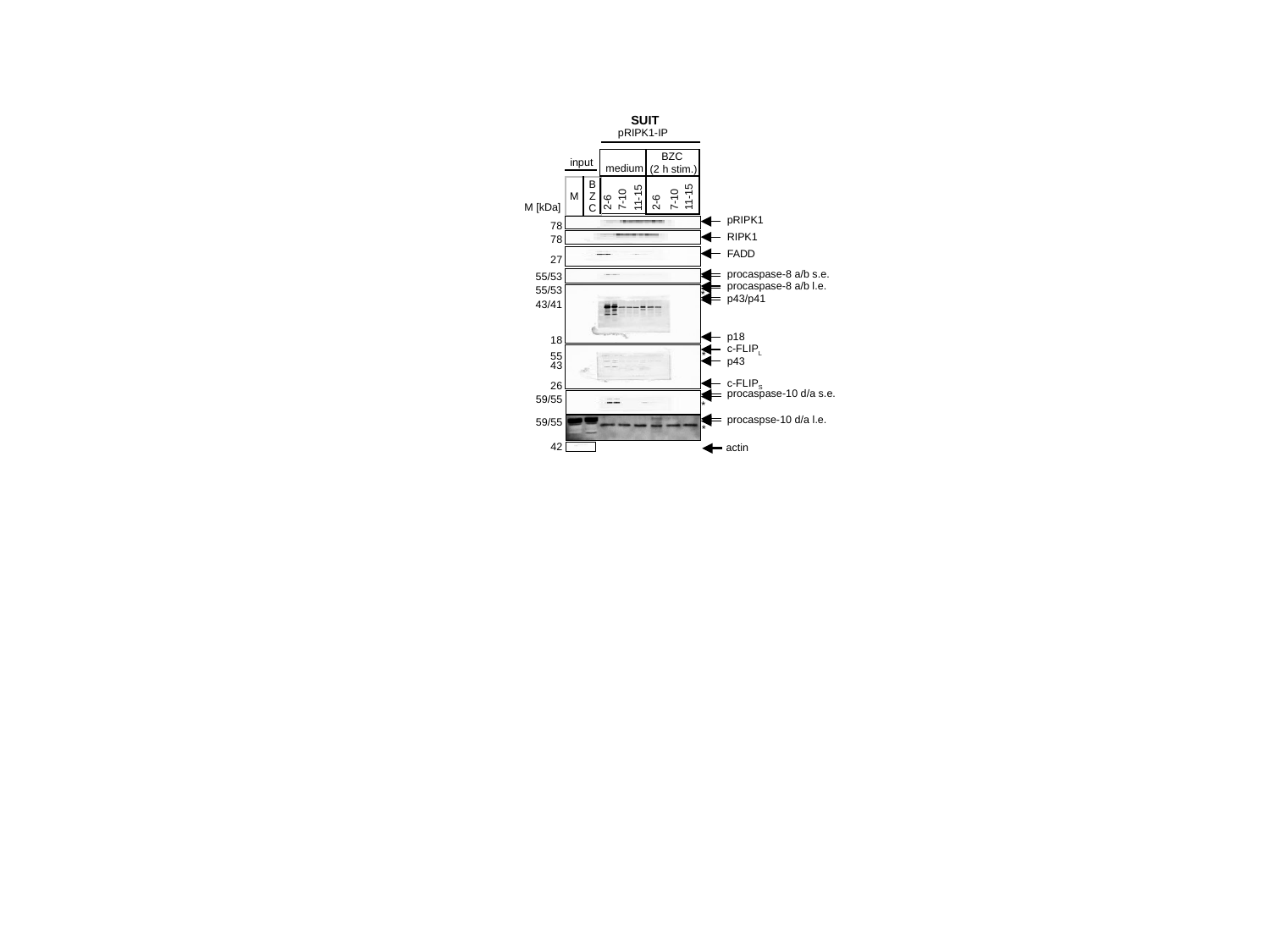

SUIT
pRIPK1-IP
BZC
(2 h stim.)
input
medium
11-15
2-6
7-10
| M | B Z C |
| --- | --- |
2-6
7-10
11-15
M [kDa]
pRIPK1
78
RIPK1
78
FADD
27
procaspase-8 a/b s.e.
55/53
procaspase-8 a/b l.e.
55/53
*
p43/p41
43/41
p18
18
c-FLIPL
*
55
p43
43
c-FLIPS
26
procaspase-10 d/a s.e.
59/55
*
procaspse-10 d/a l.e.
59/55
*
42
actin

## Slide 2
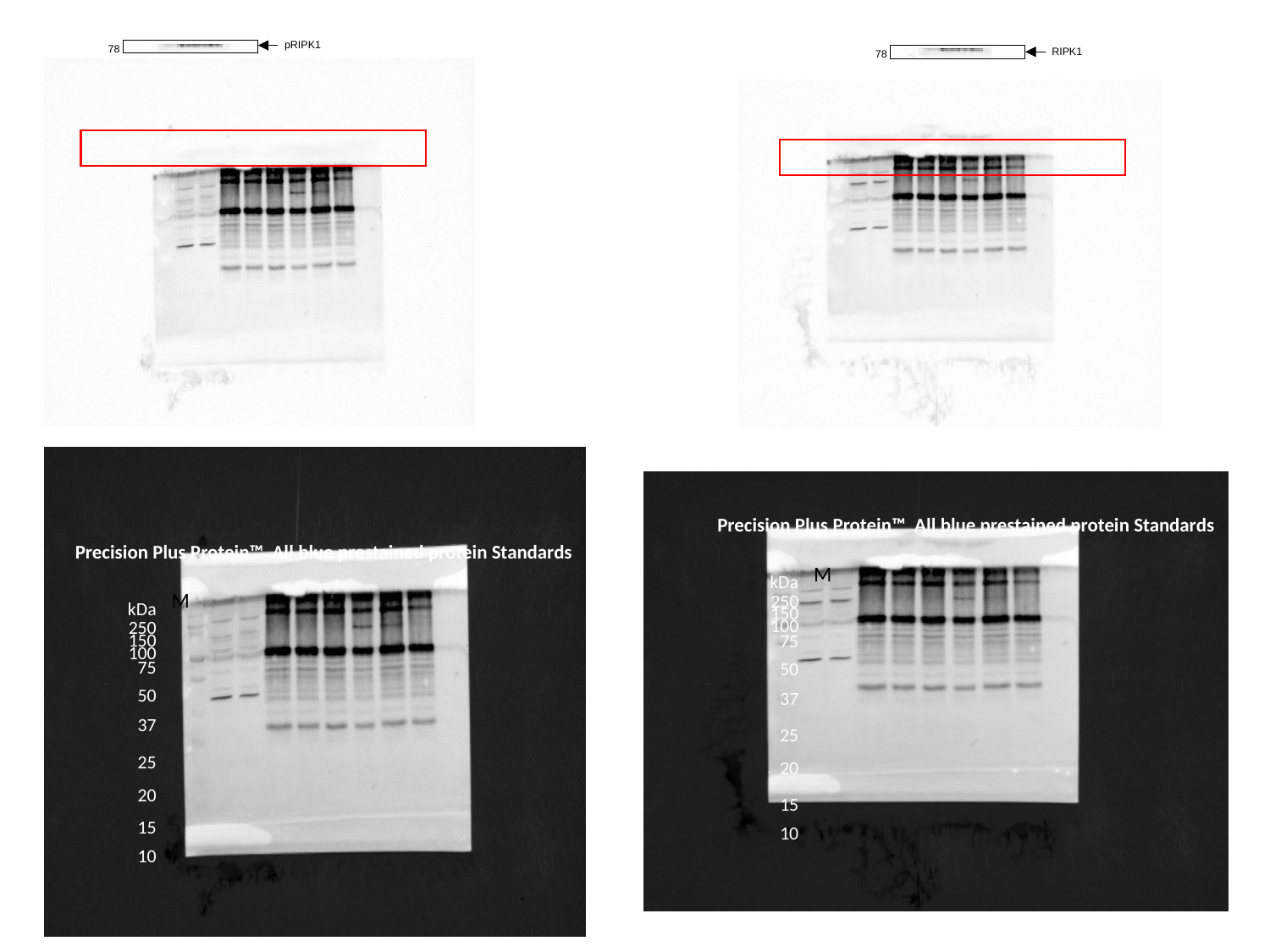

pRIPK1
78
RIPK1
78
Precision Plus Protein™ All blue prestained protein Standards
Precision Plus Protein™ All blue prestained protein Standards
M
kDa
M
250
kDa
150
100
250
150
75
100
75
50
50
37
37
25
25
20
20
15
15
10
10

## Slide 3
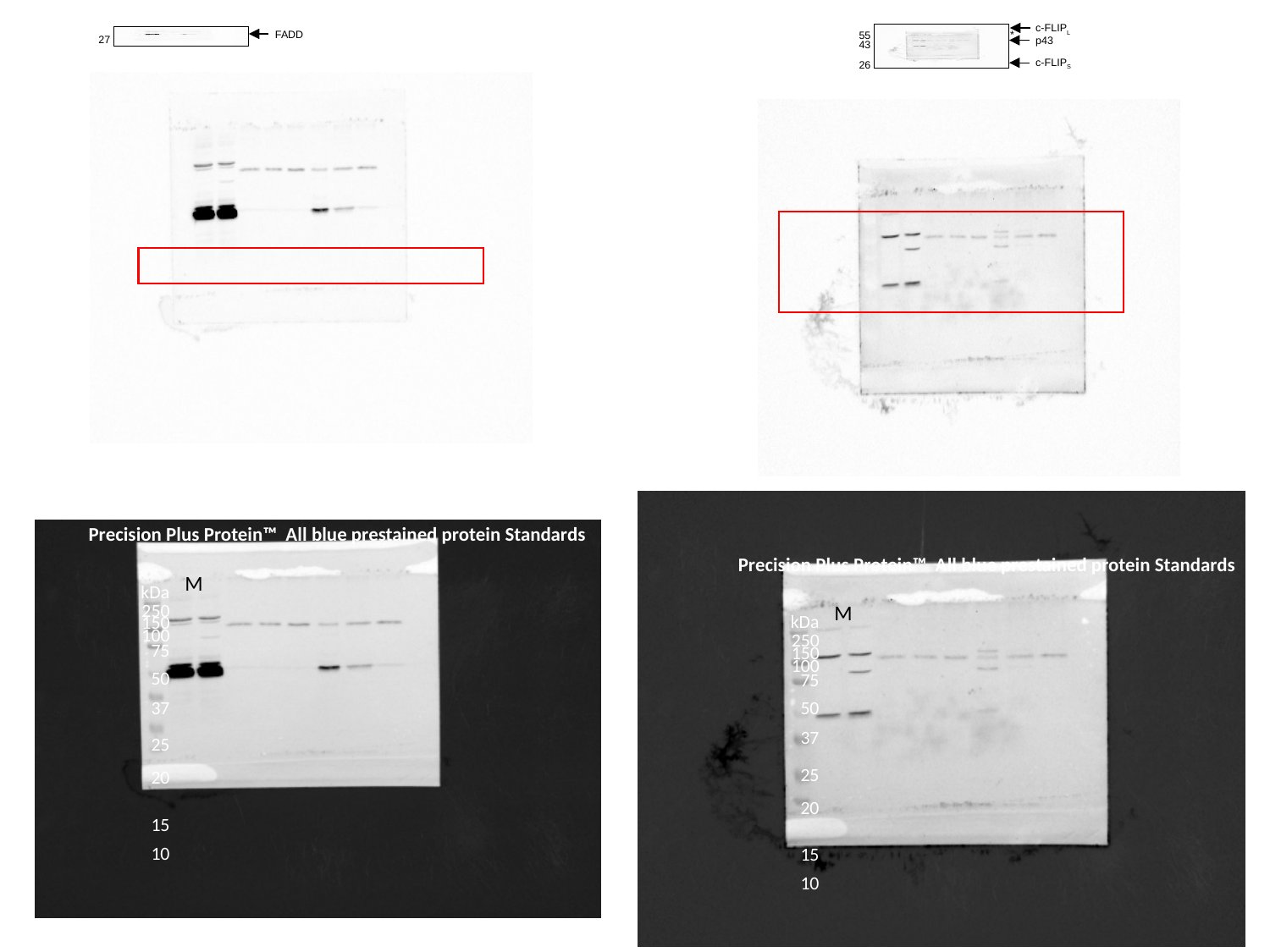

c-FLIPL
FADD
*
55
27
p43
43
c-FLIPS
26
Precision Plus Protein™ All blue prestained protein Standards
Precision Plus Protein™ All blue prestained protein Standards
M
kDa
250
M
kDa
150
100
250
75
150
100
50
75
37
50
37
25
25
20
20
15
10
15
10

## Slide 4
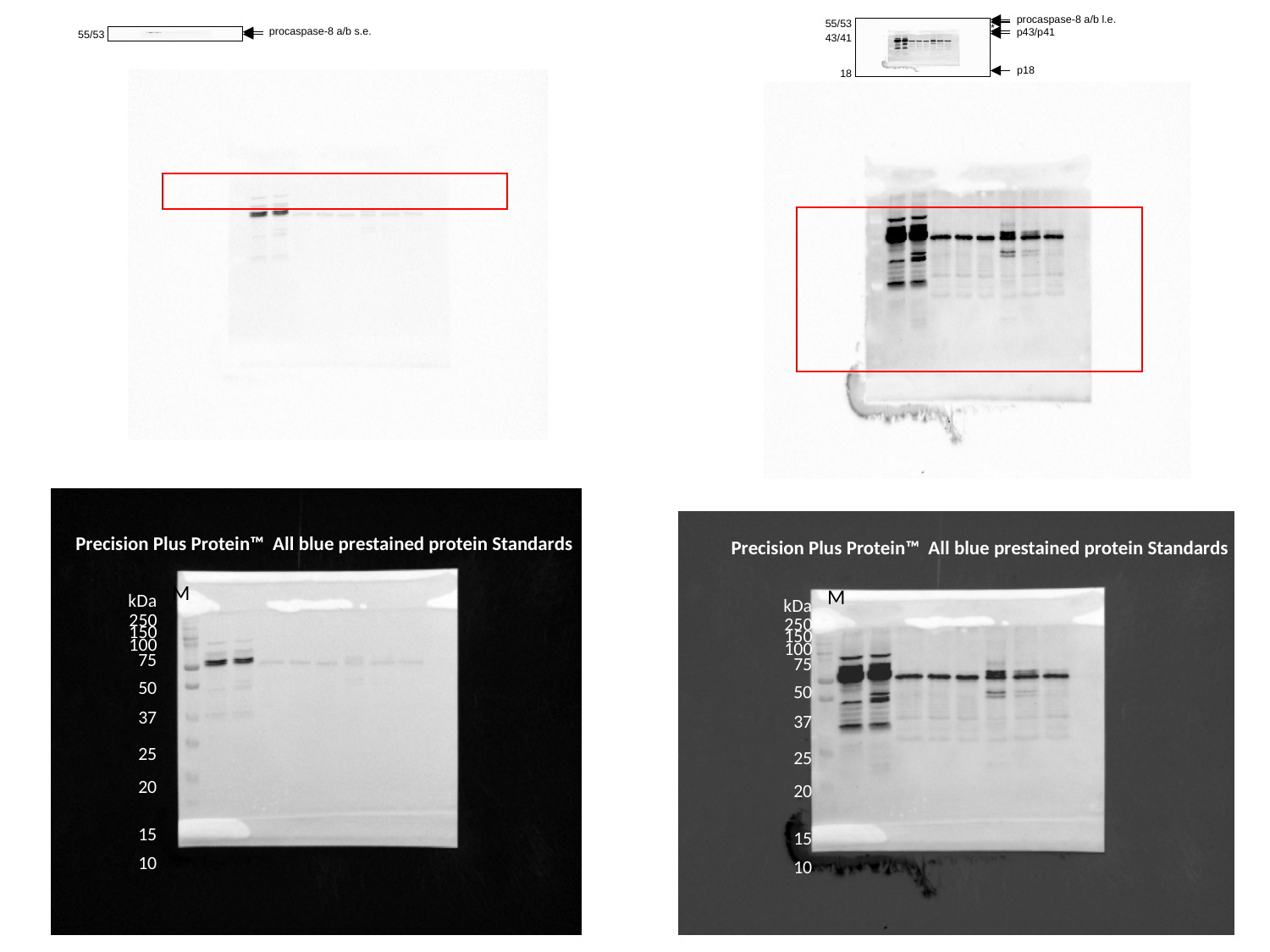

procaspase-8 a/b l.e.
55/53
*
procaspase-8 a/b s.e.
p43/p41
55/53
43/41
p18
18
Precision Plus Protein™ All blue prestained protein Standards
Precision Plus Protein™ All blue prestained protein Standards
M
M
kDa
kDa
250
250
150
150
100
100
75
75
50
50
37
37
25
25
20
20
15
15
10
10

## Slide 5
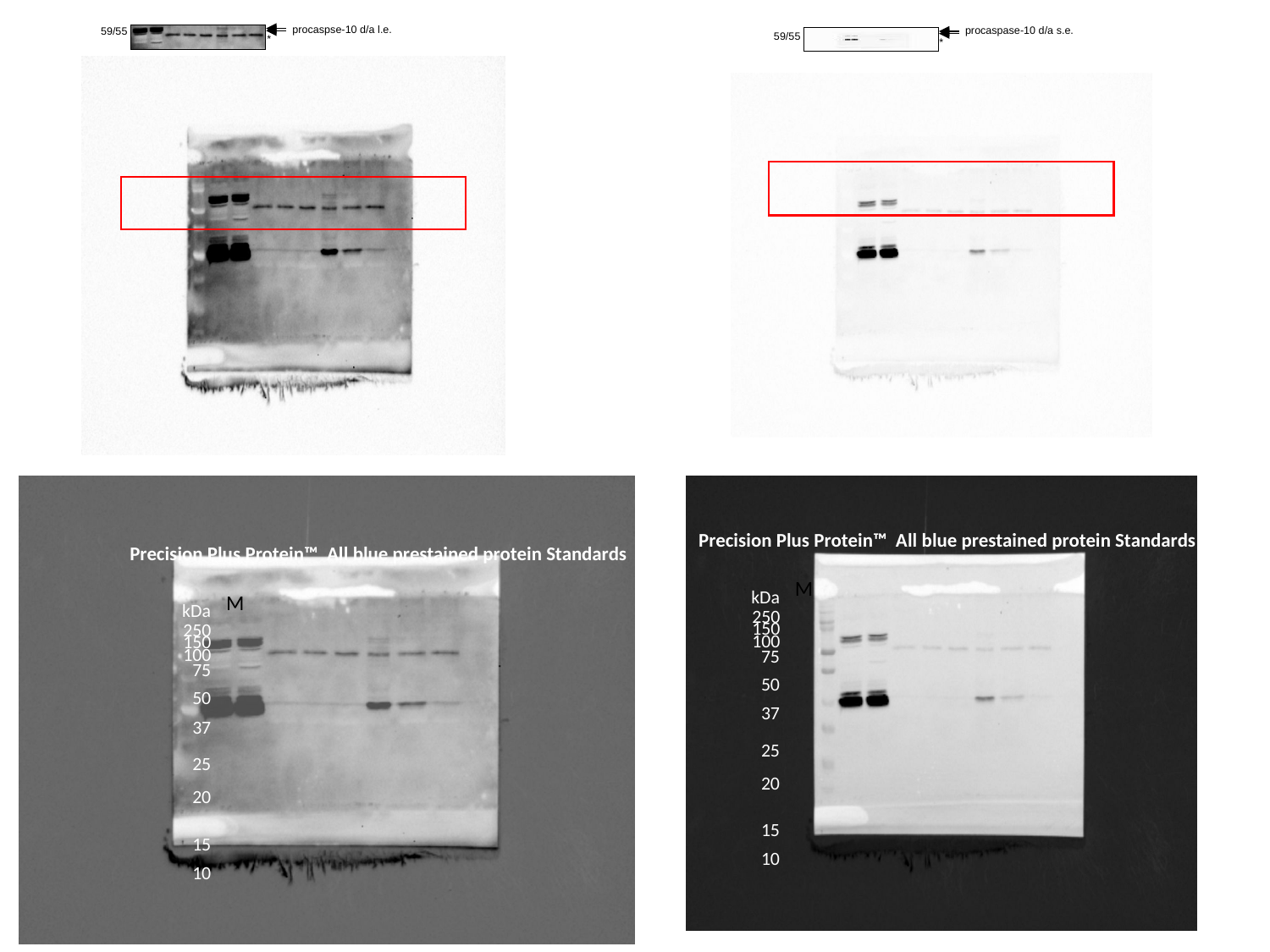

procaspse-10 d/a l.e.
procaspase-10 d/a s.e.
59/55
59/55
*
*
Precision Plus Protein™ All blue prestained protein Standards
Precision Plus Protein™ All blue prestained protein Standards
M
kDa
M
kDa
250
150
250
100
150
100
75
75
50
50
37
37
25
25
20
20
15
15
10
10

## Slide 6
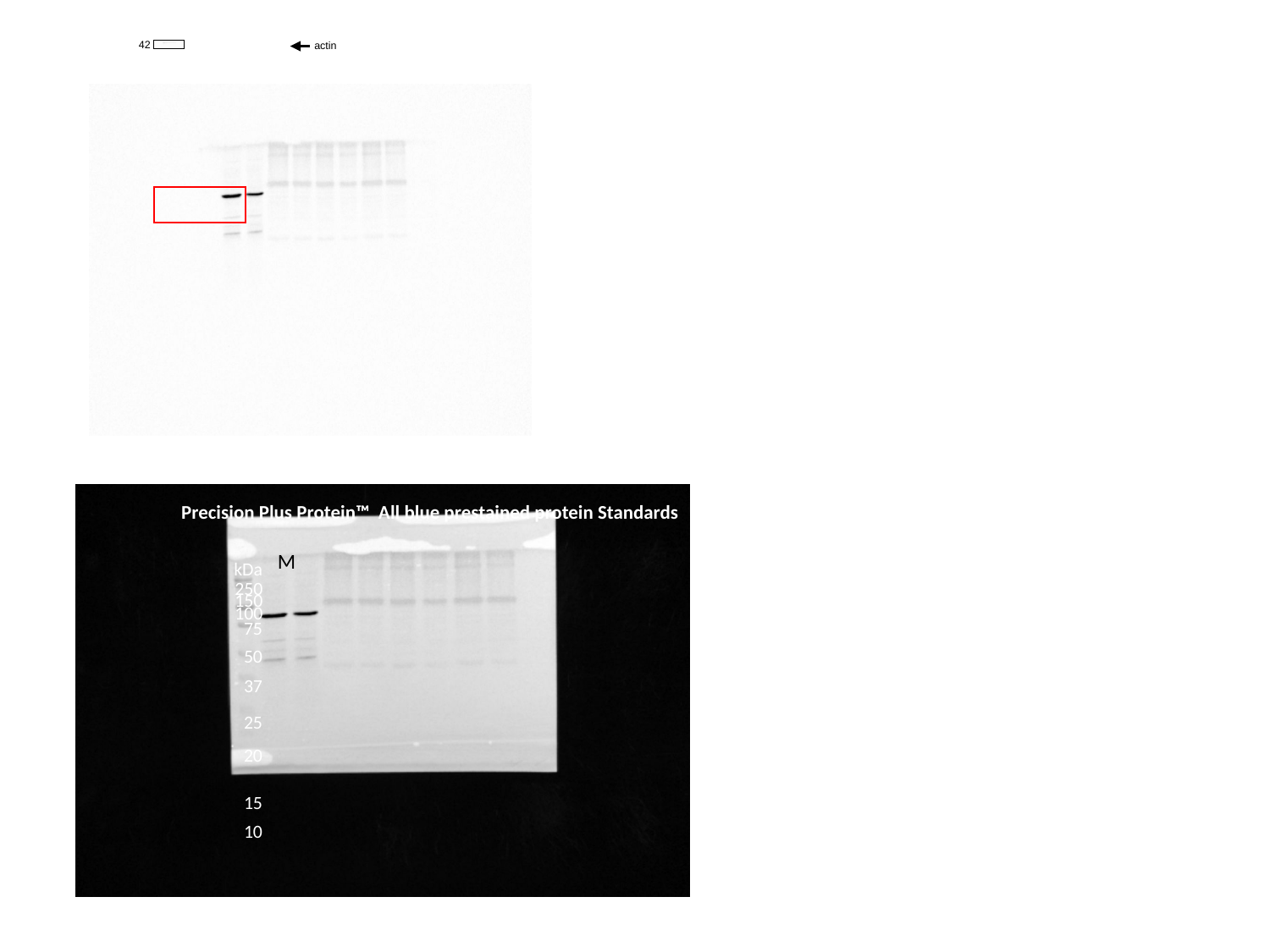

42
actin
Precision Plus Protein™ All blue prestained protein Standards
M
kDa
250
150
100
75
50
37
25
20
15
10
