# Supplementary material for: Oligomerised RIPK1 is the main core component of the CD95 necrosome
Source: EMBO J. 2025 Apr 16;44(11):3231–65. doi: 10.1038/s44318-025-00433-0 (PMC12130296; doi:10.1038/s44318-025-00433-0)
Supplement: Supplementary file 8 — Source data Fig. 8 [file 44318_2025_433_MOESM8_ESM.zip › figure8C-D.pptx]

## Slide 1
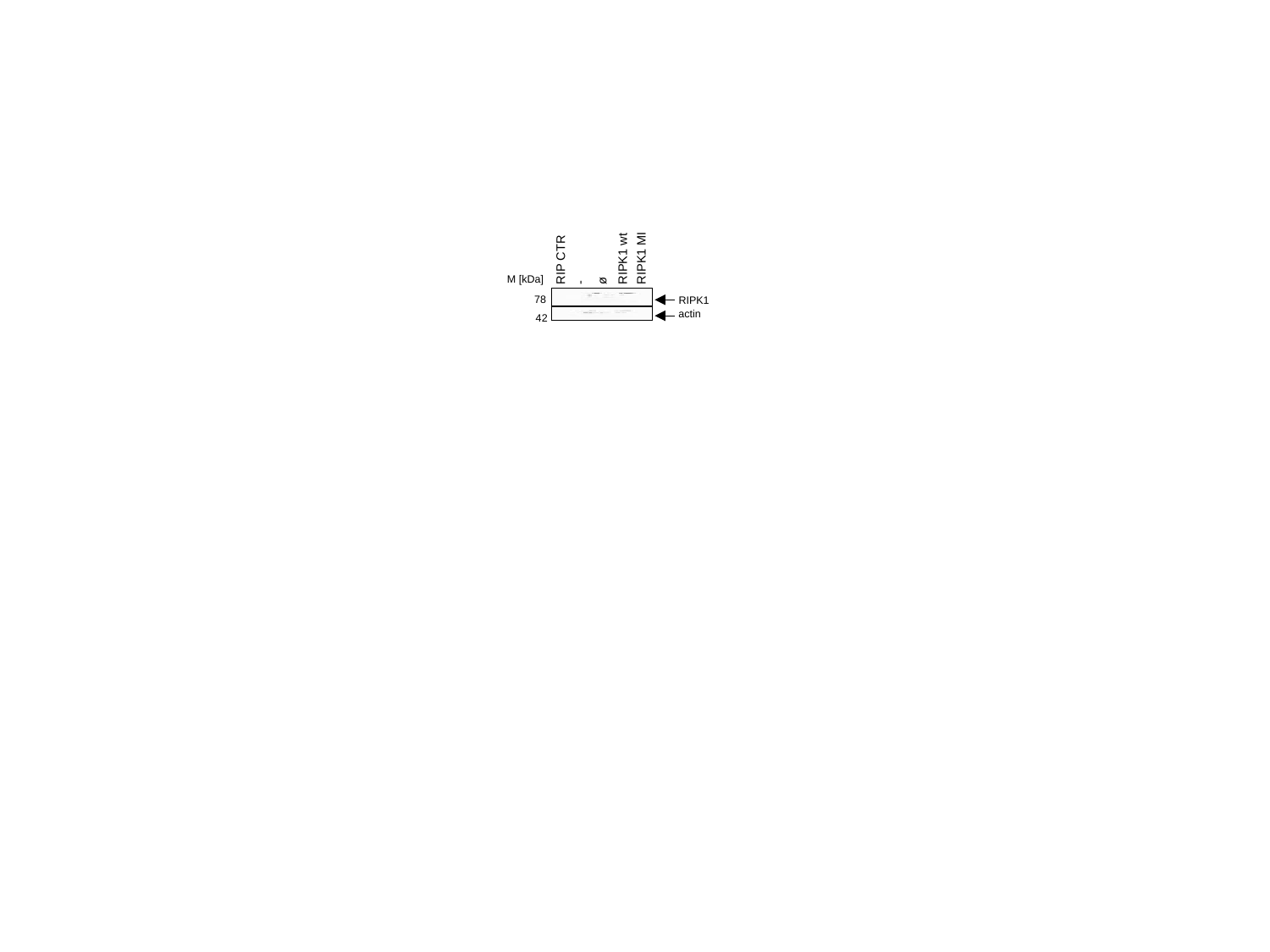

RIPK1 MI
RIPK1 wt
RIP CTR
M [kDa]
ø
-
78
RIPK1
actin
42

## Slide 2
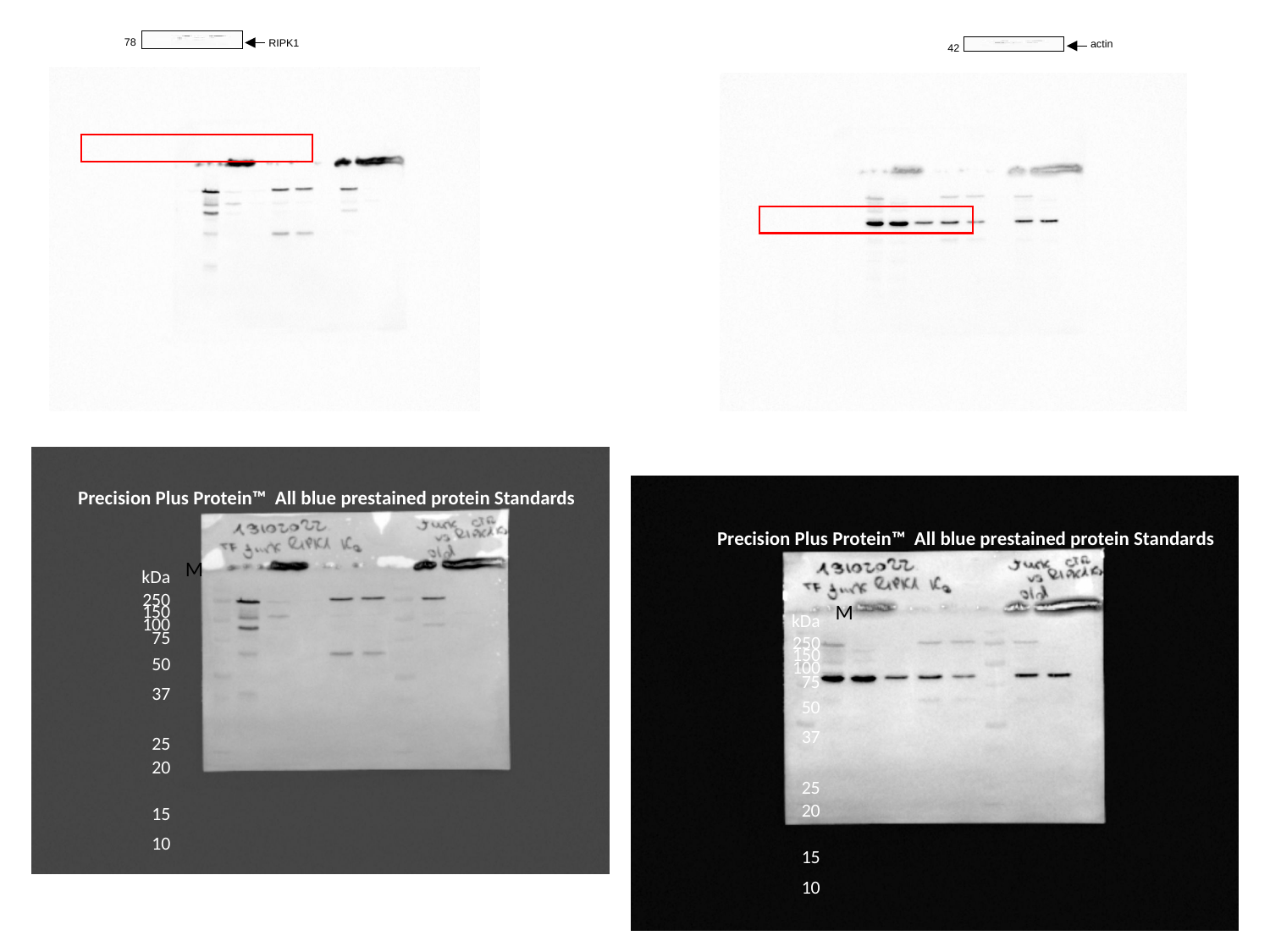

78
RIPK1
actin
42
Precision Plus Protein™ All blue prestained protein Standards
Precision Plus Protein™ All blue prestained protein Standards
M
kDa
250
M
150
kDa
100
75
250
150
50
100
75
37
50
37
25
20
25
20
15
10
15
10

## Slide 3
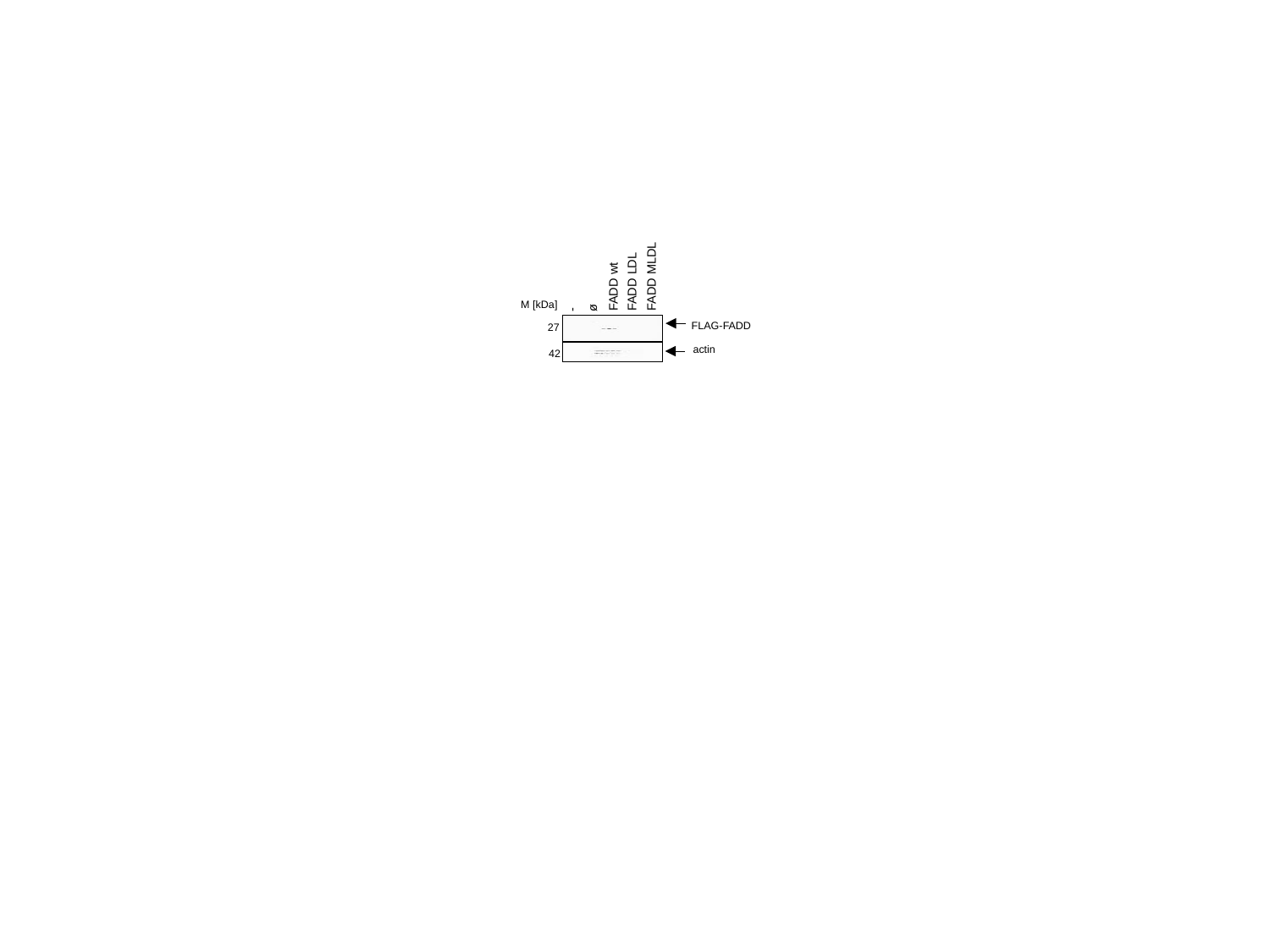

FADD MLDL
FADD LDL
FADD wt
M [kDa]
ø
-
FLAG-FADD
27
actin
42

## Slide 4
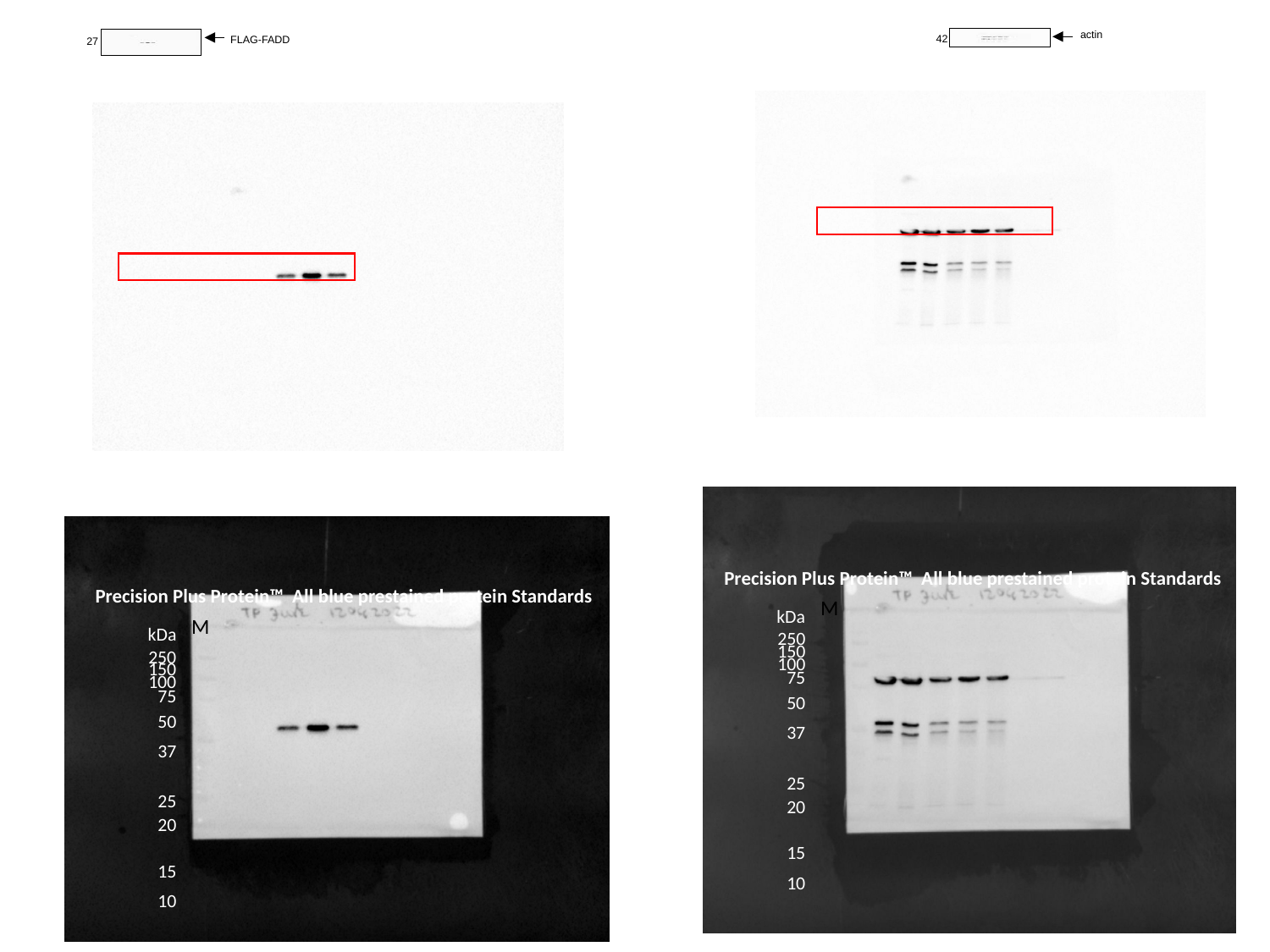

actin
42
FLAG-FADD
27
Precision Plus Protein™ All blue prestained protein Standards
Precision Plus Protein™ All blue prestained protein Standards
M
kDa
M
kDa
250
150
250
100
150
75
100
75
50
50
37
37
25
25
20
20
15
15
10
10
