# Supplementary material for: Oligomerised RIPK1 is the main core component of the CD95 necrosome
Source: EMBO J. 2025 Apr 16;44(11):3231–65. doi: 10.1038/s44318-025-00433-0 (PMC12130296; doi:10.1038/s44318-025-00433-0)
Supplement: Supplementary file 10 — Figure EV1 Source Data [file 44318_2025_433_MOESM10_ESM.zip › EV1B.pptx]

## Slide 1
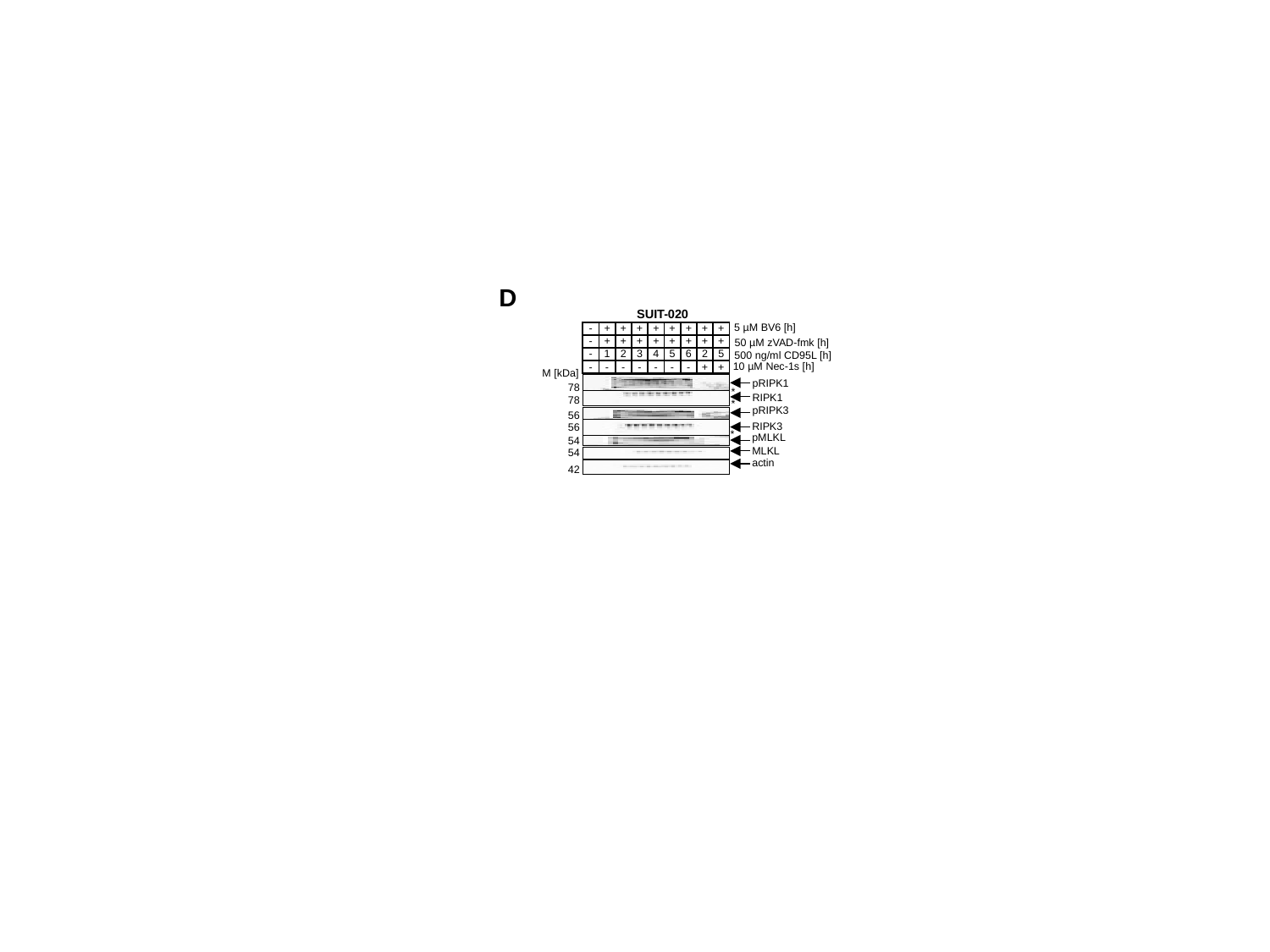

D
SUIT-020
5 µM BV6 [h]
| - | + | + | + | + | + | + | + | + |
| --- | --- | --- | --- | --- | --- | --- | --- | --- |
| - | + | + | + | + | + | + | + | + |
| - | 1 | 2 | 3 | 4 | 5 | 6 | 2 | 5 |
| - | - | - | - | - | - | - | + | + |
50 µM zVAD-fmk [h]
500 ng/ml CD95L [h]
10 µM Nec-1s [h]
M [kDa]
pRIPK1
78
*
RIPK1
78
*
pRIPK3
56
RIPK3
56
*
pMLKL
54
MLKL
54
actin
42

## Slide 2
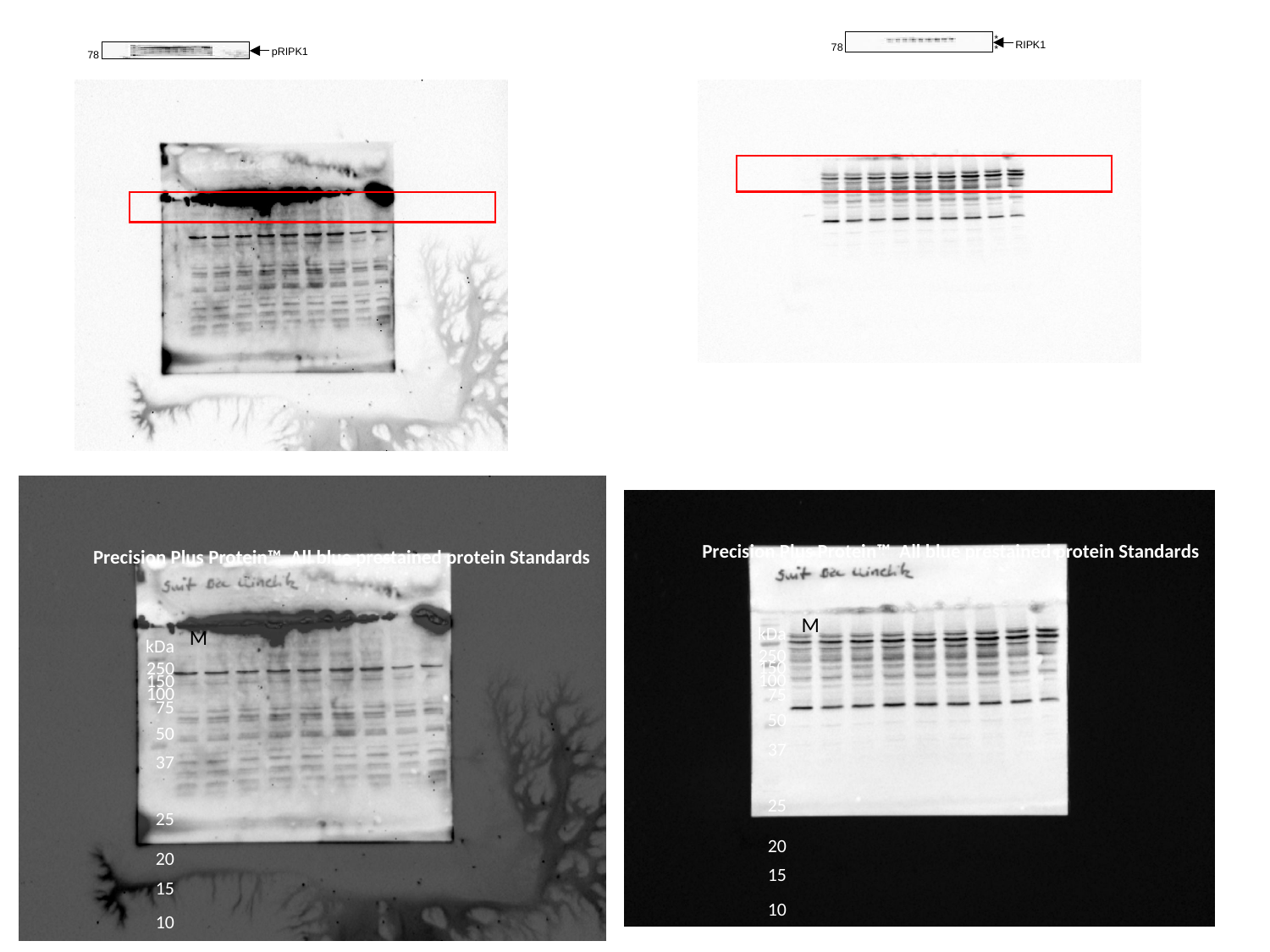

*
RIPK1
78
*
pRIPK1
78
Precision Plus Protein™ All blue prestained protein Standards
Precision Plus Protein™ All blue prestained protein Standards
M
kDa
M
kDa
250
150
250
100
150
100
75
75
50
50
37
37
25
25
20
20
15
15
10
10

## Slide 3
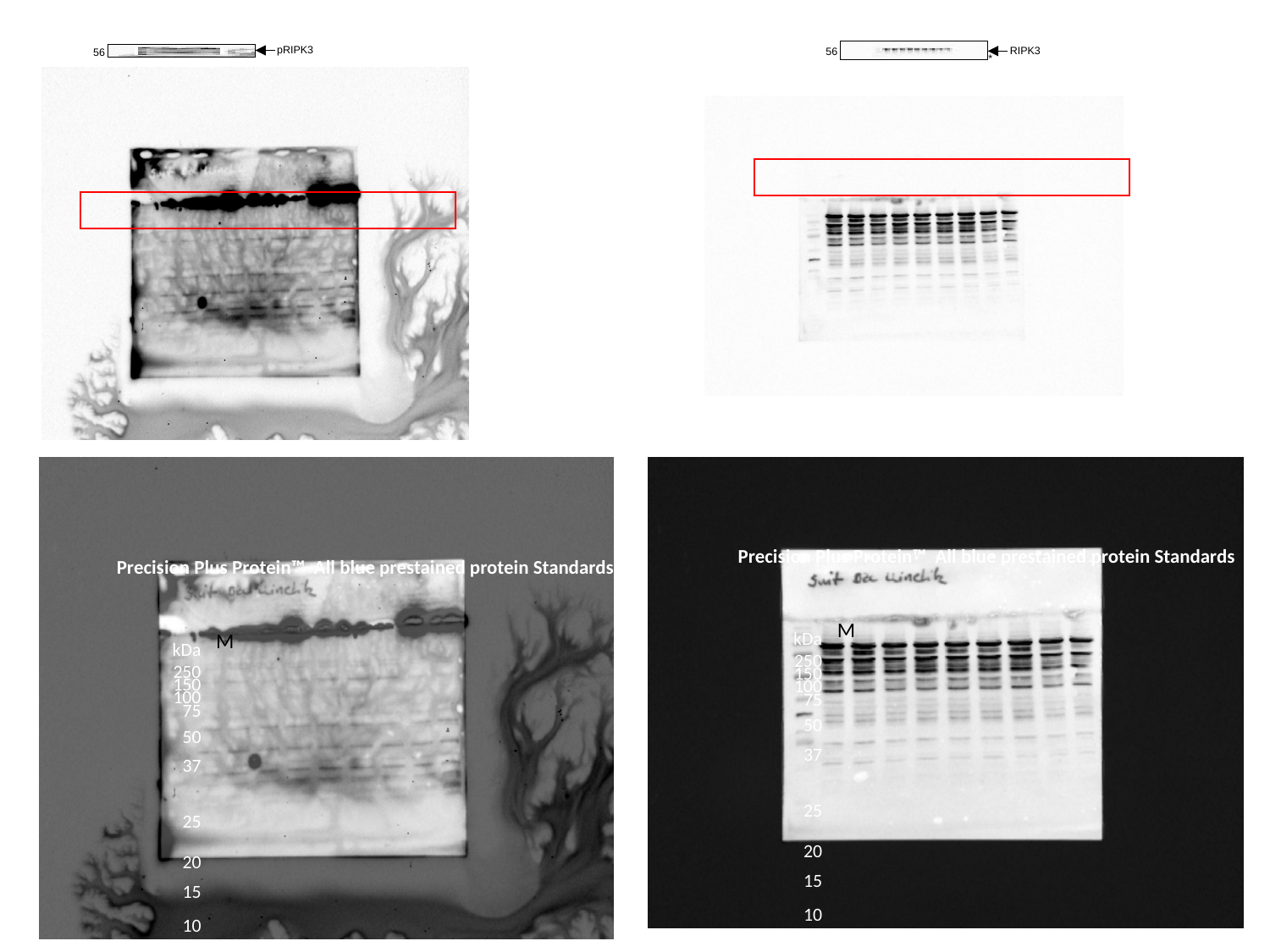

pRIPK3
RIPK3
56
56
*
Precision Plus Protein™ All blue prestained protein Standards
Precision Plus Protein™ All blue prestained protein Standards
M
kDa
M
kDa
250
250
150
150
100
100
75
75
50
50
37
37
25
25
20
20
15
15
10
10

## Slide 4
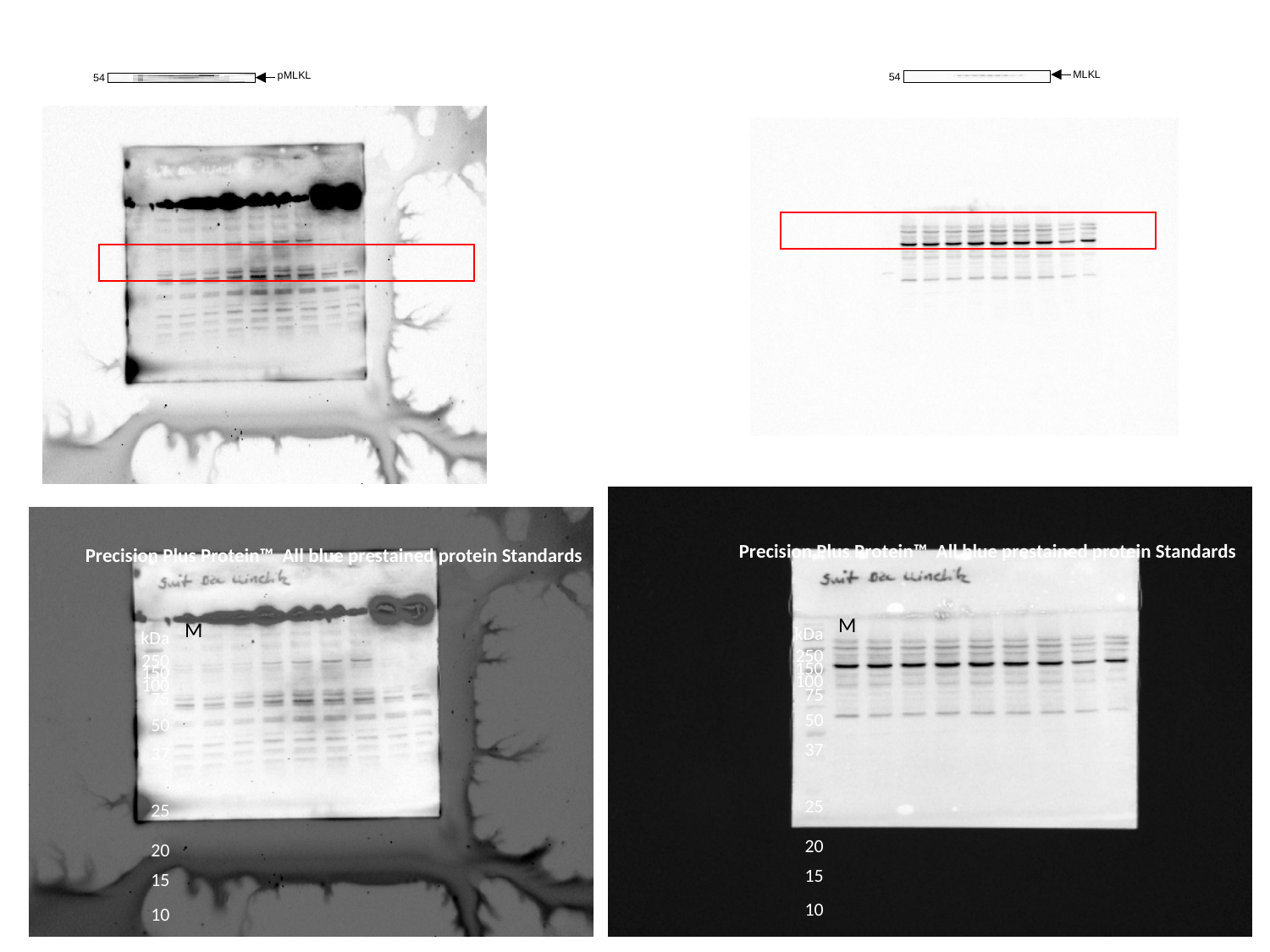

MLKL
pMLKL
54
54
Precision Plus Protein™ All blue prestained protein Standards
Precision Plus Protein™ All blue prestained protein Standards
M
M
kDa
kDa
250
250
150
150
100
100
75
75
50
50
37
37
25
25
20
20
15
15
10
10

## Slide 5
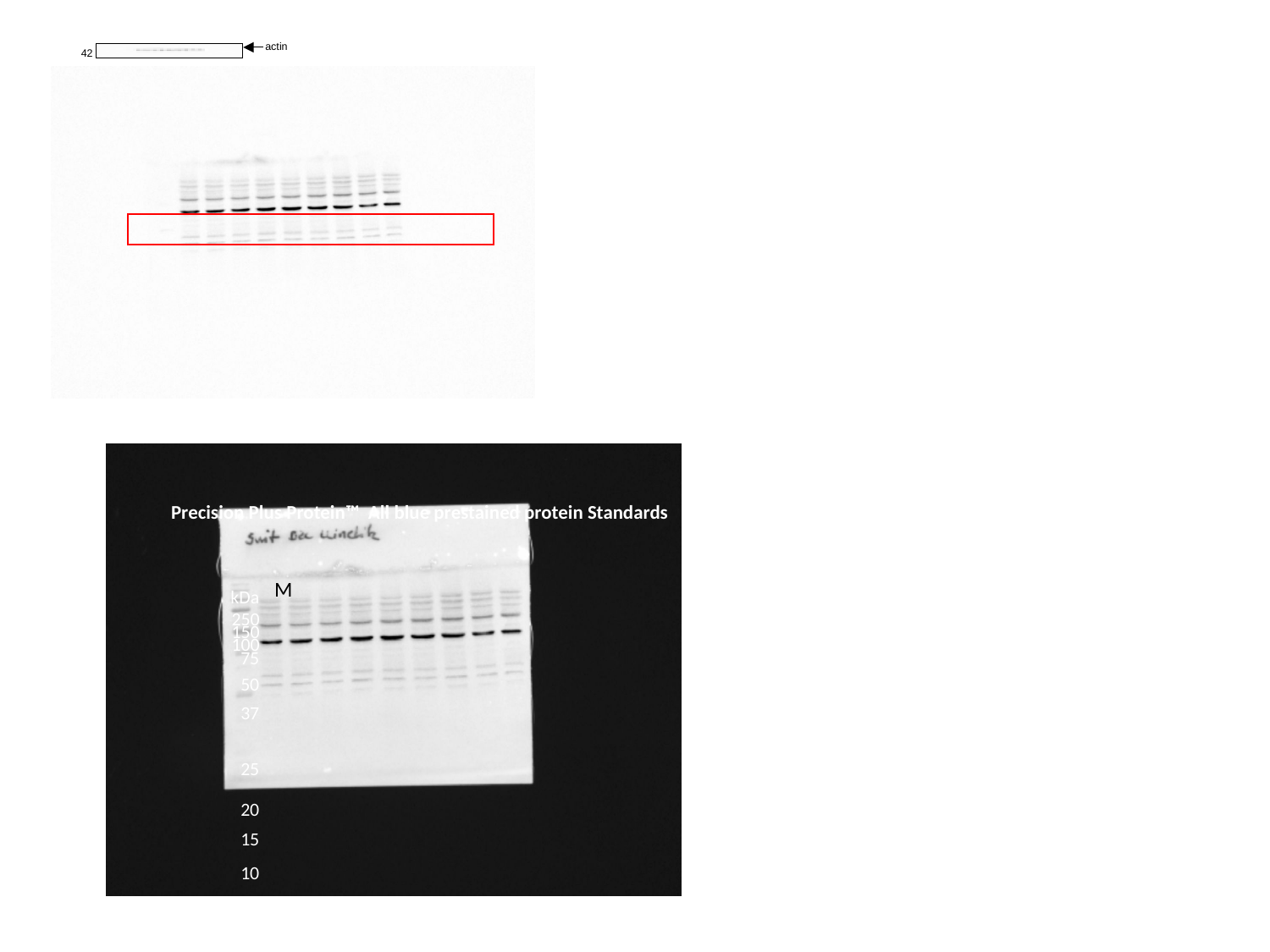

actin
42
Precision Plus Protein™ All blue prestained protein Standards
M
kDa
250
150
100
75
50
37
25
20
15
10
