# Supplementary material for: Oligomerised RIPK1 is the main core component of the CD95 necrosome
Source: EMBO J. 2025 Apr 16;44(11):3231–65. doi: 10.1038/s44318-025-00433-0 (PMC12130296; doi:10.1038/s44318-025-00433-0)
Supplement: Supplementary file 11 — Figure EV2 Source Data [file 44318_2025_433_MOESM11_ESM.zip › EV2G.pptx]

## Slide 1
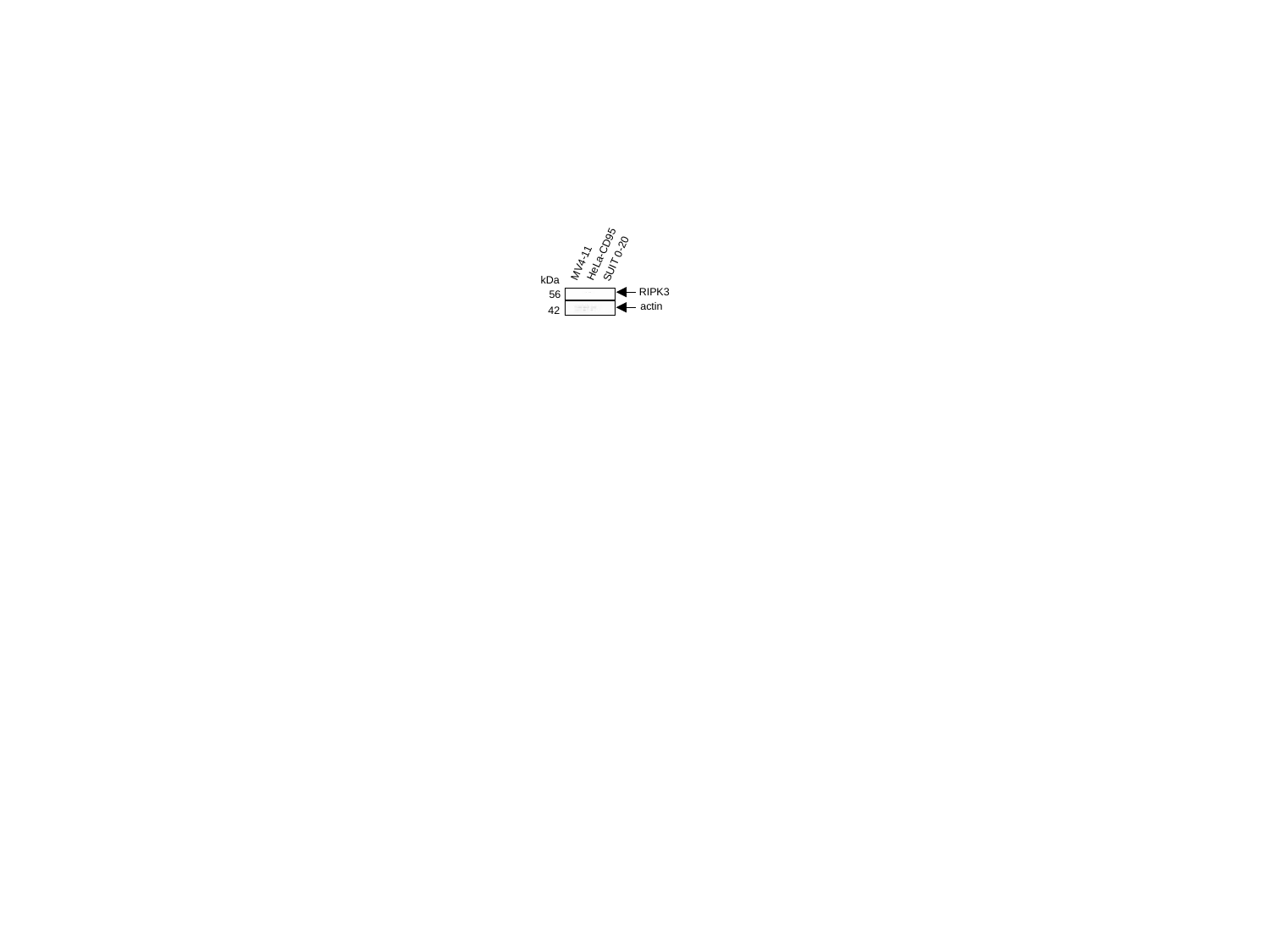

HeLa-CD95
SUIT 0-20
MV4-11
kDa
RIPK3
56
actin
42

## Slide 2
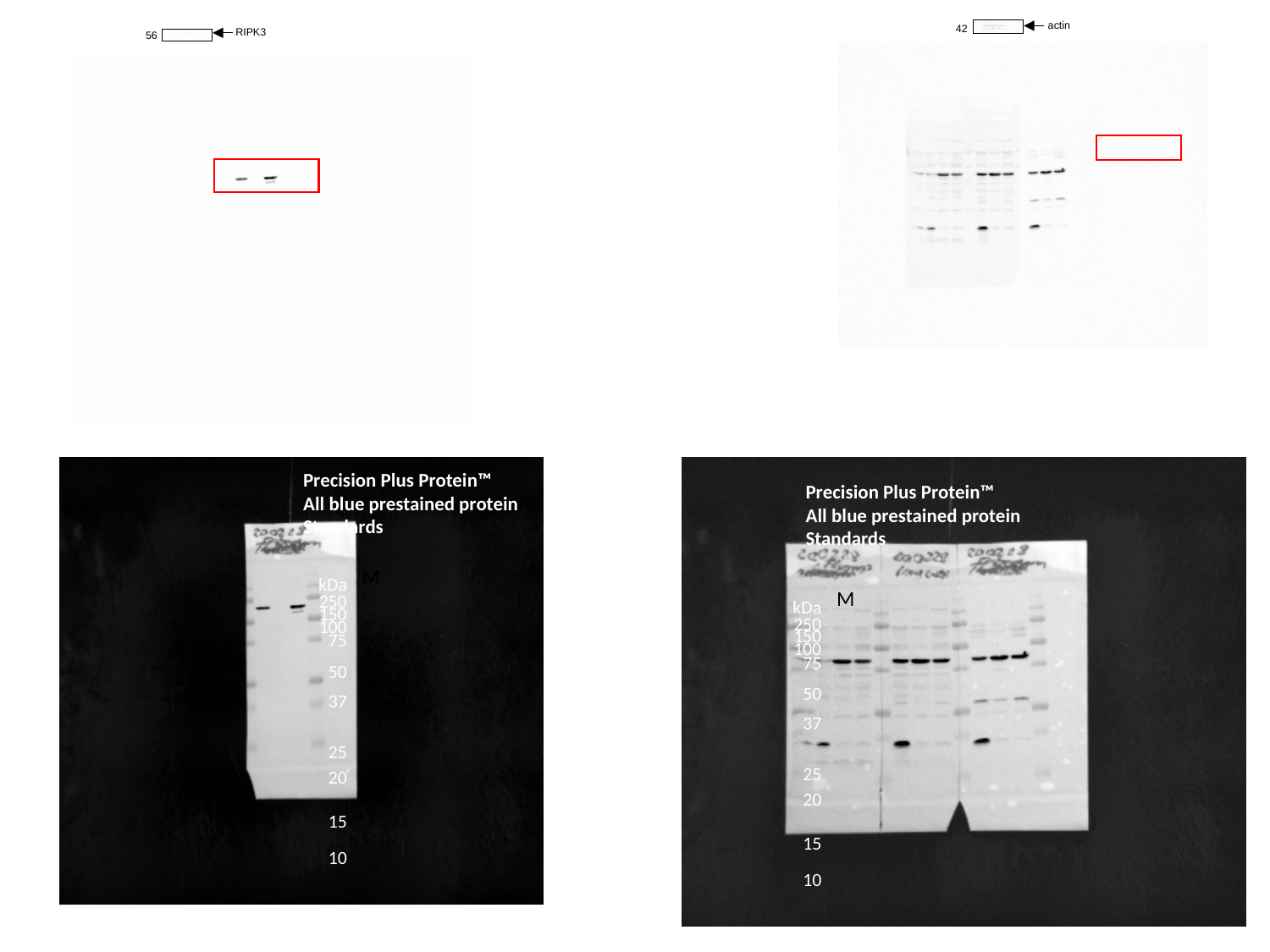

actin
42
RIPK3
56
Precision Plus Protein™
All blue prestained protein
Standards
Precision Plus Protein™
All blue prestained protein
Standards
M
kDa
M
250
kDa
150
250
100
150
75
100
75
50
50
37
37
25
25
20
20
15
15
10
10
