# Supplementary material for: Oligomerised RIPK1 is the main core component of the CD95 necrosome
Source: EMBO J. 2025 Apr 16;44(11):3231–65. doi: 10.1038/s44318-025-00433-0 (PMC12130296; doi:10.1038/s44318-025-00433-0)
Supplement: Supplementary file 11 — Figure EV2 Source Data [file 44318_2025_433_MOESM11_ESM.zip › EV2H.pptx]

## Slide 1
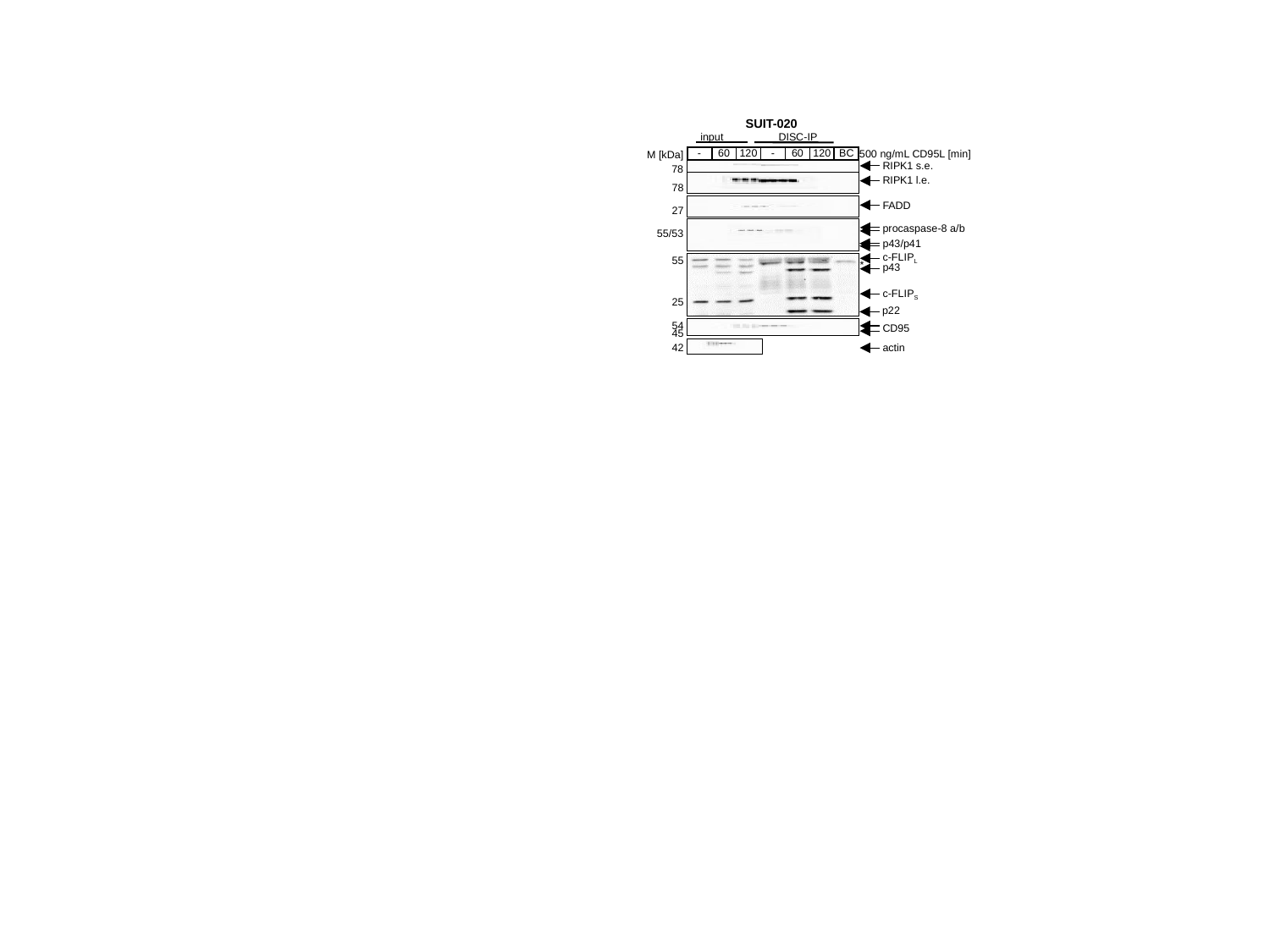

SUIT-020
input
DISC-IP
500 ng/mL CD95L [min]
M [kDa]
78
78
27
55/53
55
25
54
45
42
| - | 60 | 120 | - | 60 | 120 | BC |
| --- | --- | --- | --- | --- | --- | --- |
RIPK1 s.e.
RIPK1 l.e.
FADD
procaspase-8 a/b
p43/p41
c-FLIPL
*
p43
c-FLIPS
p22
CD95
actin
10

## Slide 2
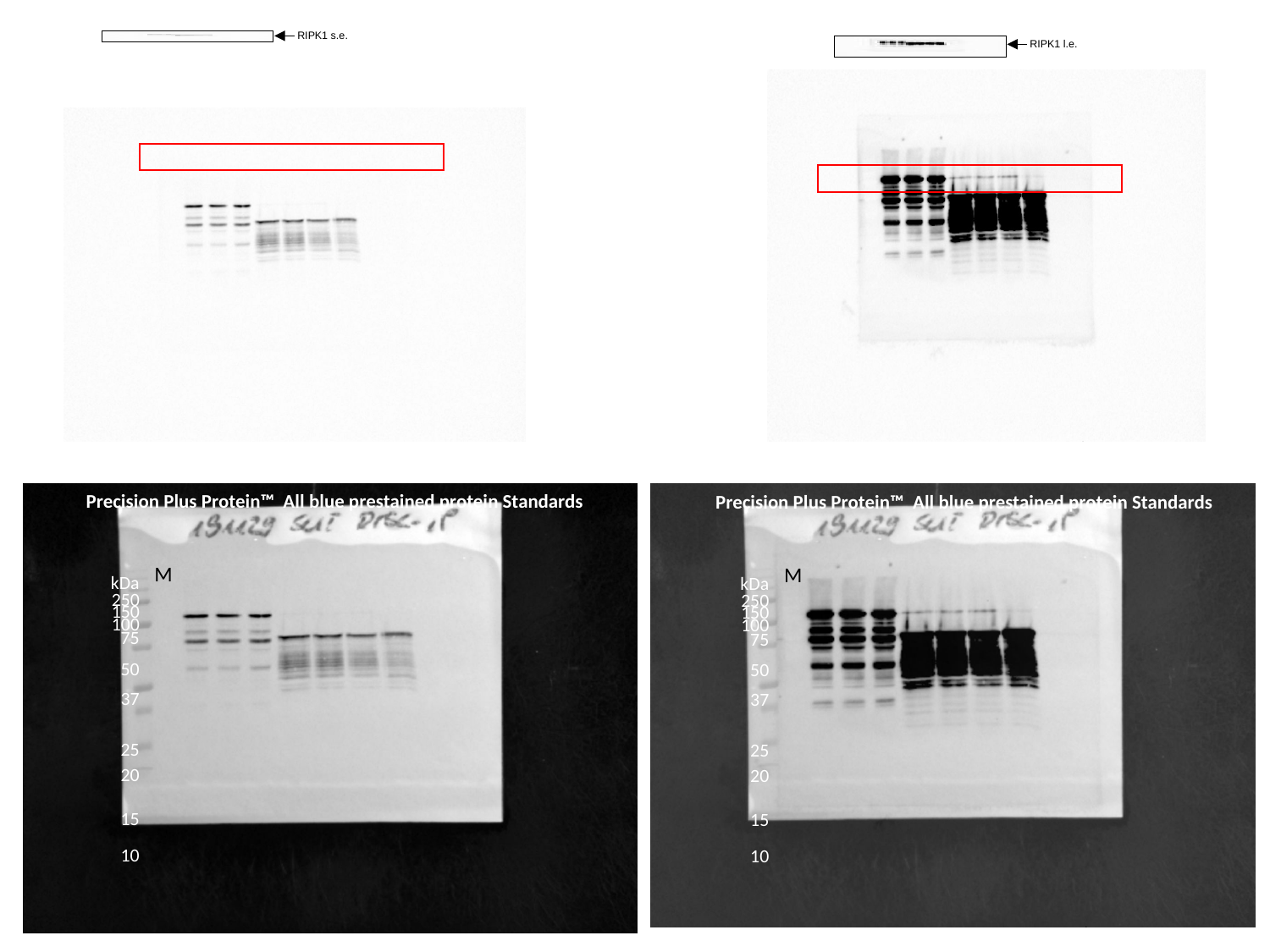

RIPK1 s.e.
RIPK1 l.e.
Precision Plus Protein™ All blue prestained protein Standards
Precision Plus Protein™ All blue prestained protein Standards
M
M
kDa
kDa
250
250
150
150
100
100
75
75
50
50
37
37
25
25
20
20
15
15
10
10

## Slide 3
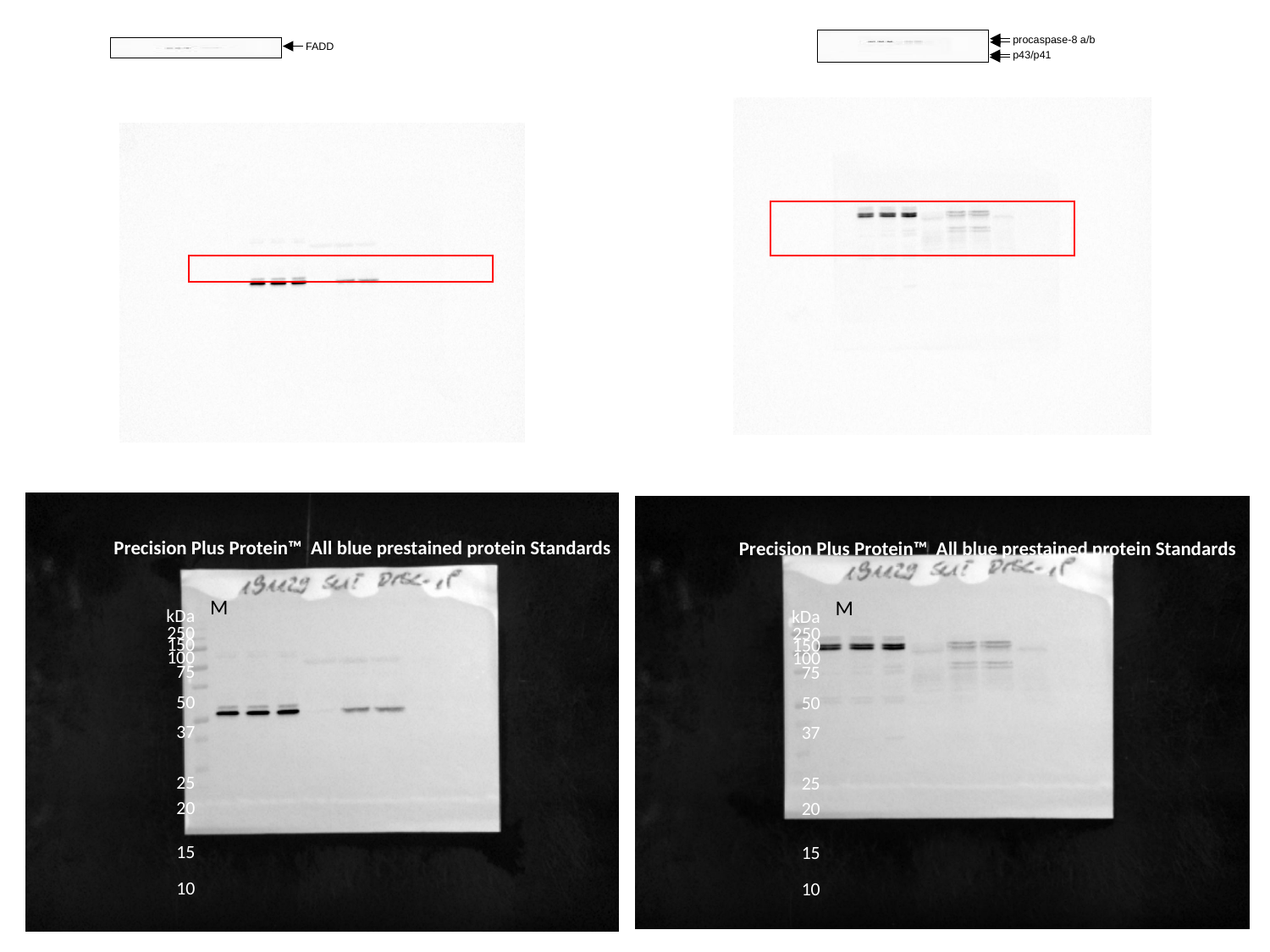

procaspase-8 a/b
FADD
p43/p41
Precision Plus Protein™ All blue prestained protein Standards
Precision Plus Protein™ All blue prestained protein Standards
M
M
kDa
kDa
250
250
150
150
100
100
75
75
50
50
37
37
25
25
20
20
15
15
10
10

## Slide 4
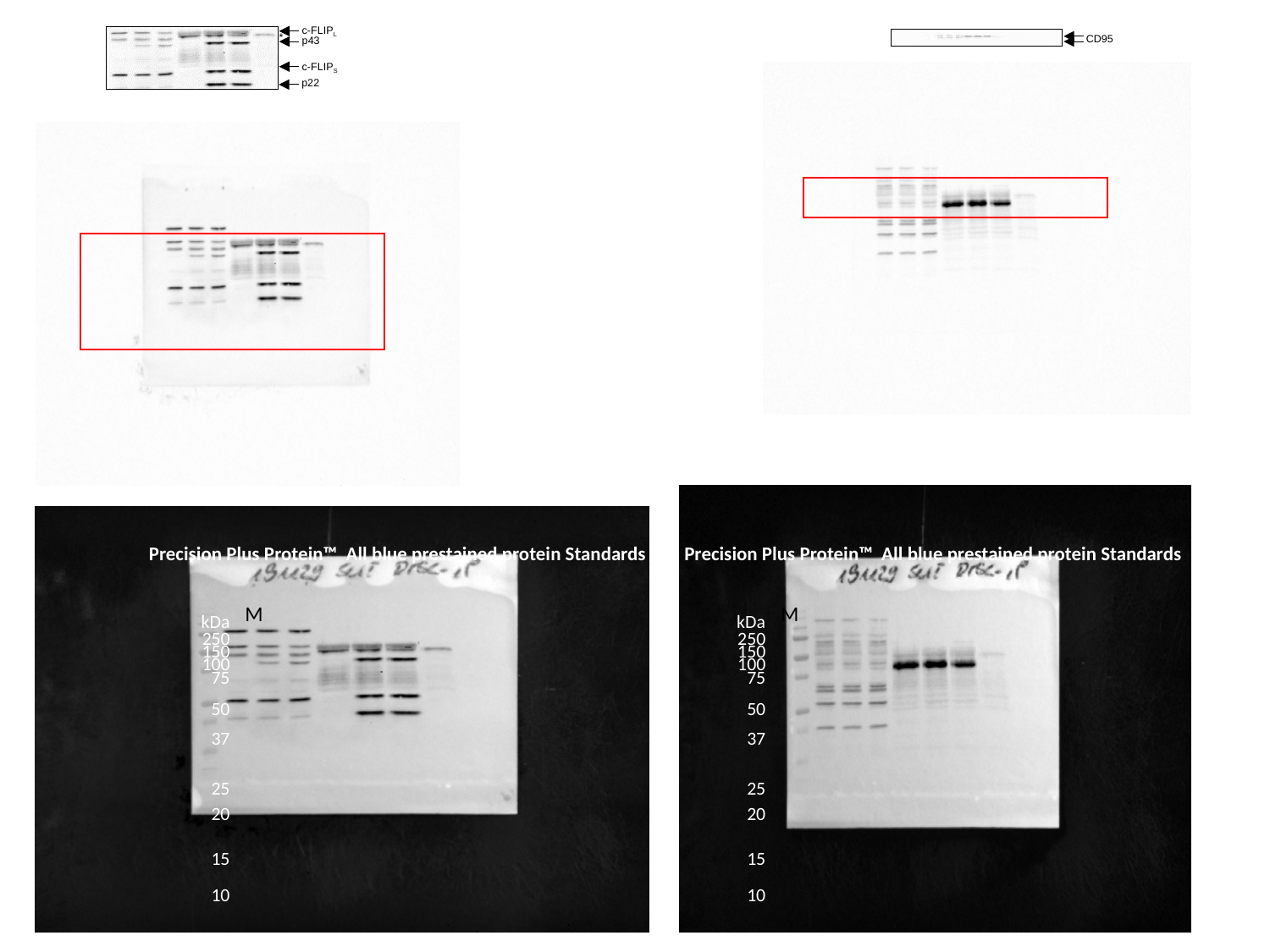

c-FLIPL
*
CD95
p43
c-FLIPS
p22
Precision Plus Protein™ All blue prestained protein Standards
Precision Plus Protein™ All blue prestained protein Standards
M
M
kDa
kDa
250
250
150
150
100
100
75
75
50
50
37
37
25
25
20
20
15
15
10
10

## Slide 5
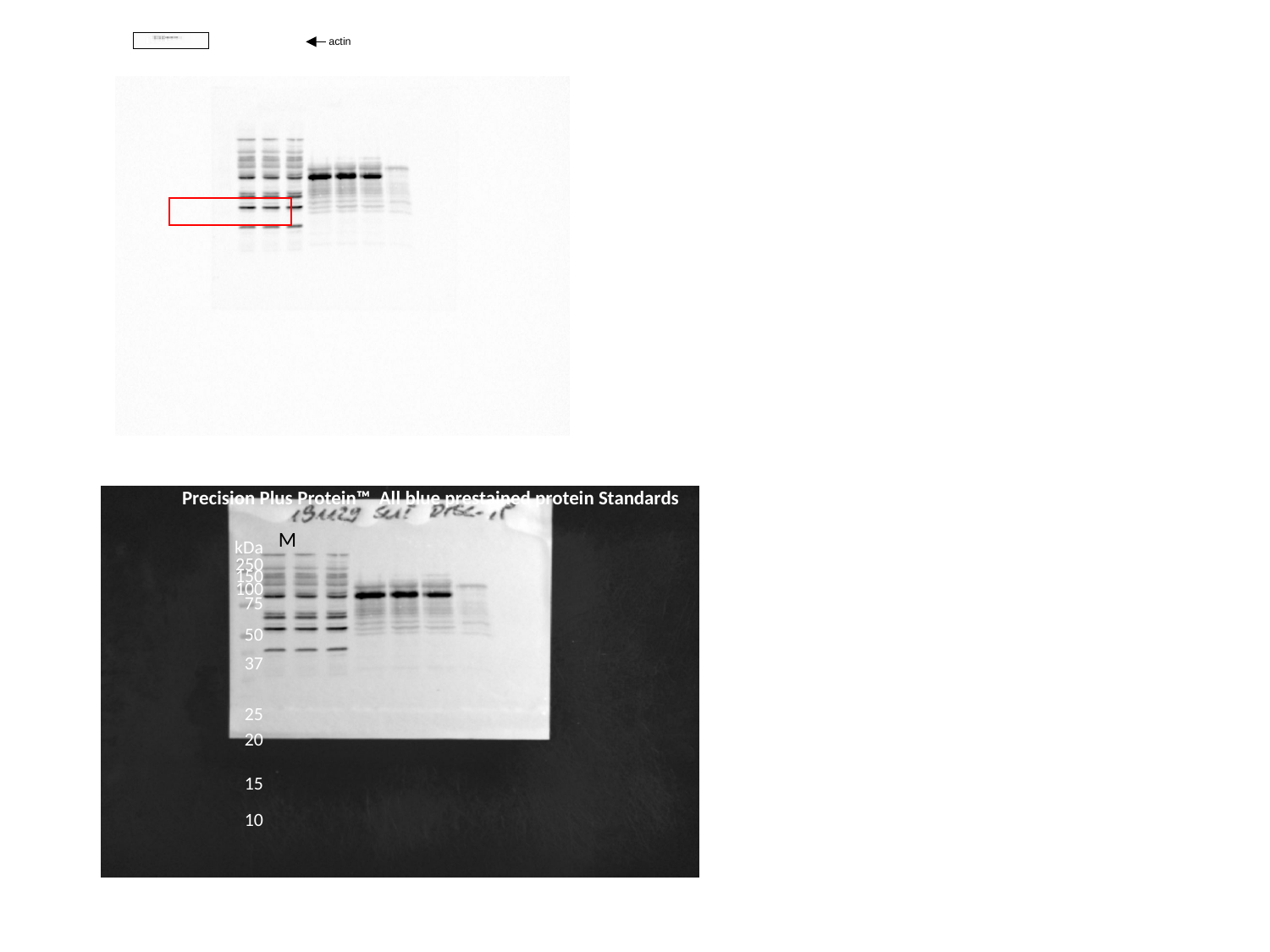

actin
Precision Plus Protein™ All blue prestained protein Standards
M
kDa
250
150
100
75
50
37
25
20
15
10
