# Supplementary material for: Oligomerised RIPK1 is the main core component of the CD95 necrosome
Source: EMBO J. 2025 Apr 16;44(11):3231–65. doi: 10.1038/s44318-025-00433-0 (PMC12130296; doi:10.1038/s44318-025-00433-0)
Supplement: Supplementary file 12 — Figure EV3 Source Data [file 44318_2025_433_MOESM12_ESM.zip › EV3B.pptx]

## Slide 1
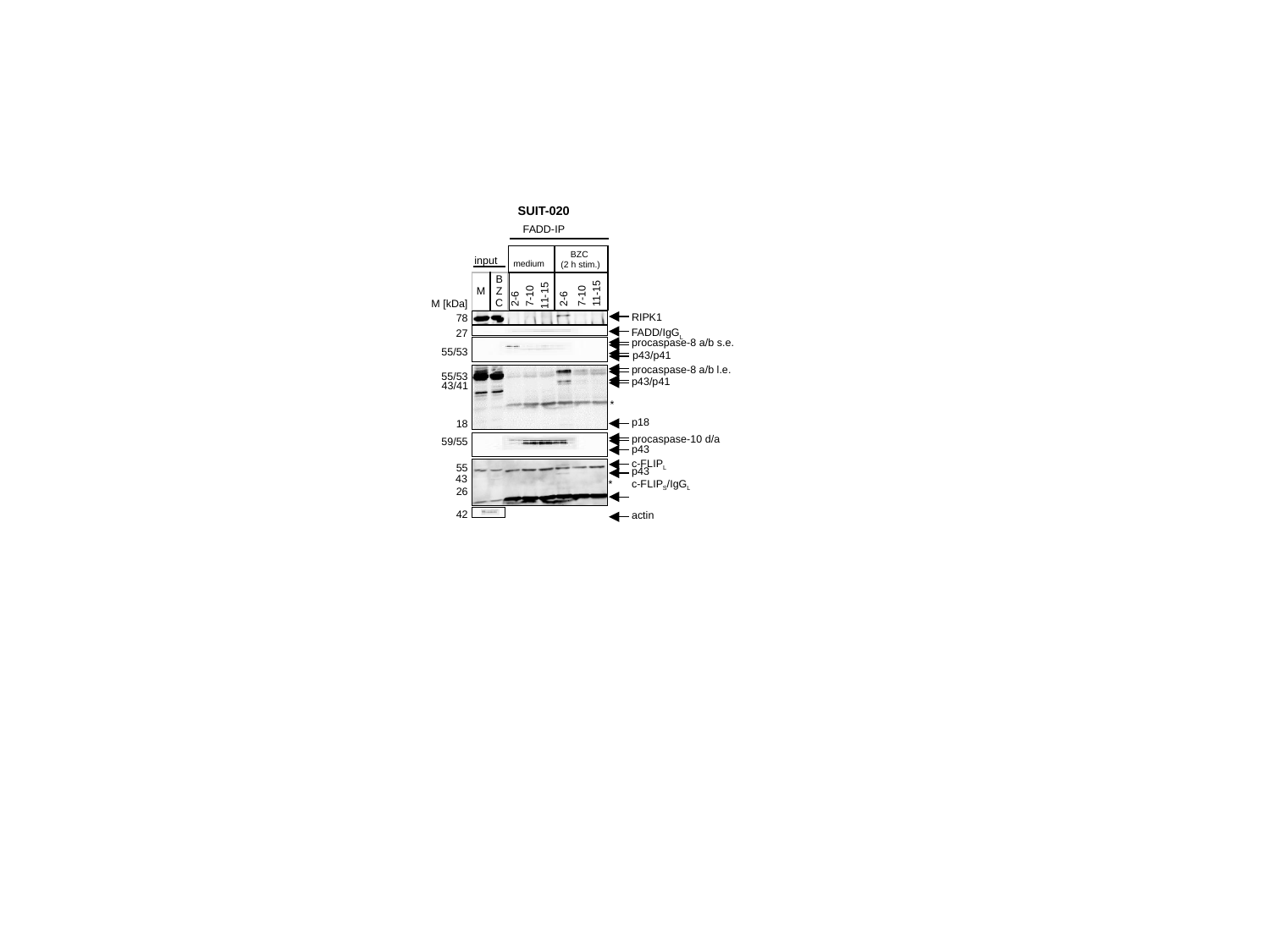

SUIT-020
FADD-IP
BZC
(2 h stim.)
input
medium
11-15
2-6
7-10
| M | B Z C |
| --- | --- |
2-6
7-10
11-15
M [kDa]
RIPK1
78
FADD/IgGL
27
procaspase-8 a/b s.e.
55/53
p43/p41
procaspase-8 a/b l.e.
55/53
p43/p41
43/41
*
p18
18
procaspase-10 d/a
59/55
p43
c-FLIPL
55
p43
43
*
c-FLIPS/IgGL
26
42
actin

## Slide 2
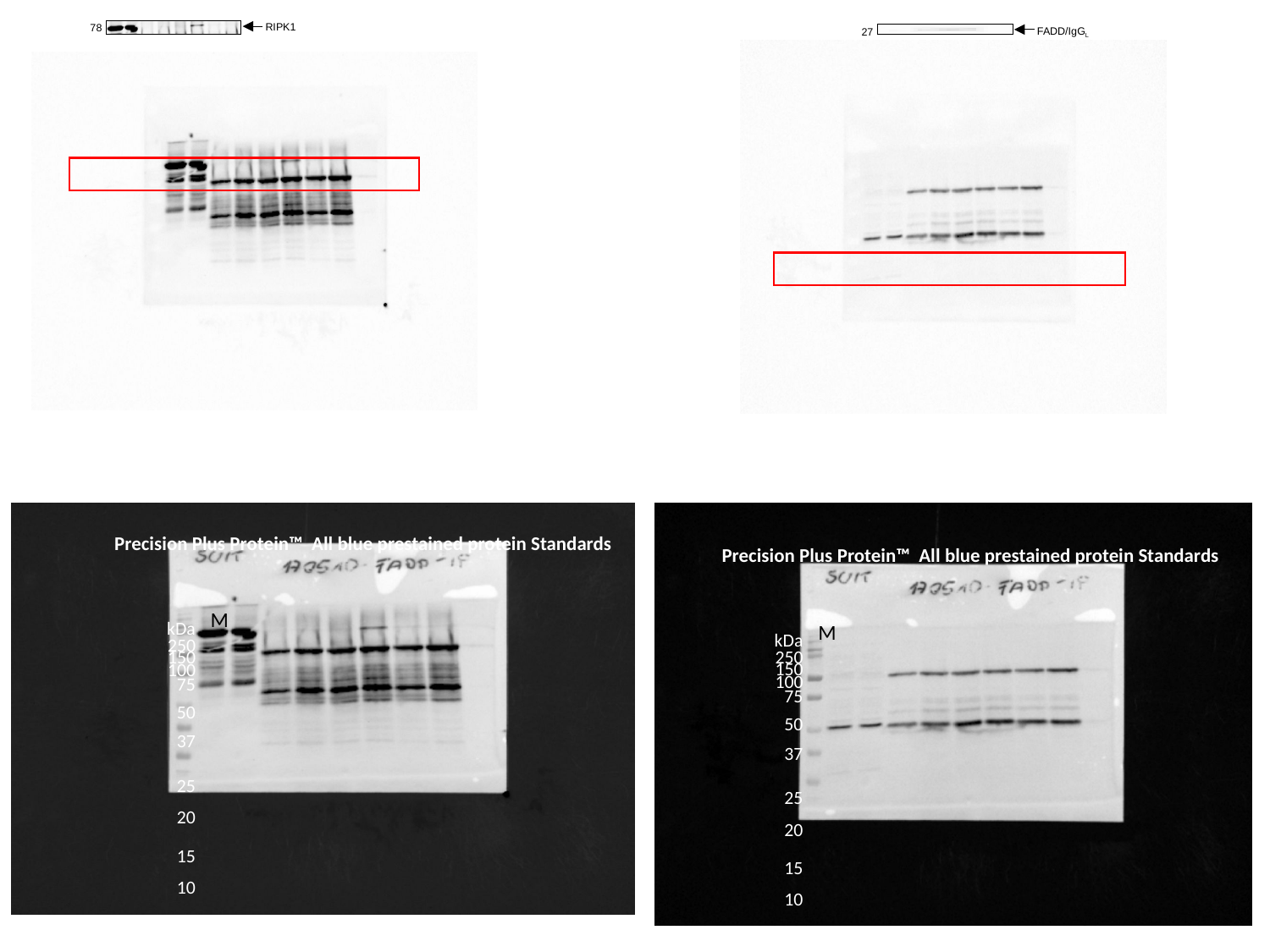

RIPK1
78
FADD/IgGL
27
Precision Plus Protein™ All blue prestained protein Standards
Precision Plus Protein™ All blue prestained protein Standards
M
kDa
M
kDa
250
250
150
150
100
100
75
75
50
50
37
37
25
25
20
20
15
15
10
10

## Slide 3
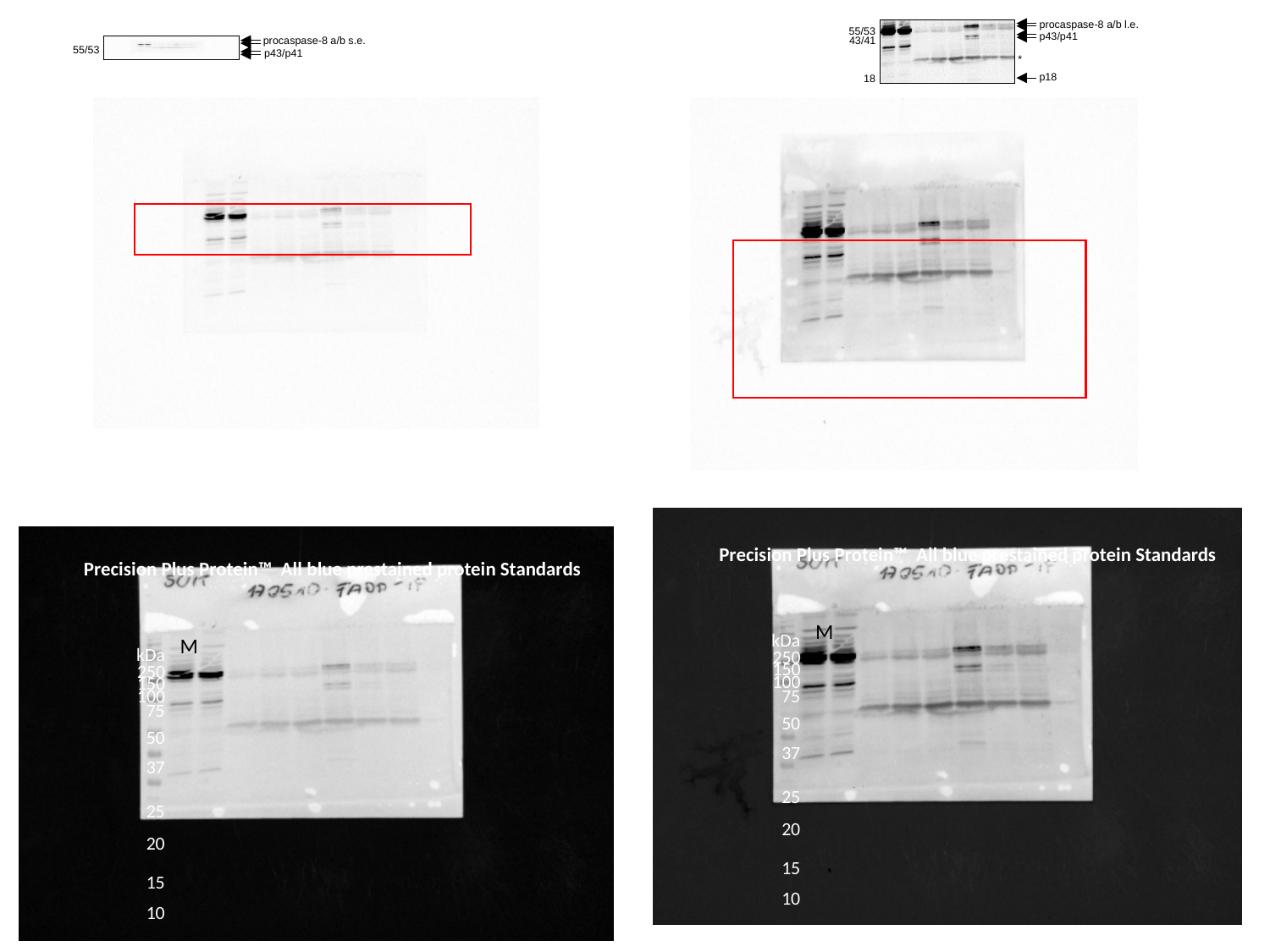

procaspase-8 a/b l.e.
55/53
p43/p41
43/41
procaspase-8 a/b s.e.
55/53
p43/p41
*
p18
18
Precision Plus Protein™ All blue prestained protein Standards
Precision Plus Protein™ All blue prestained protein Standards
M
kDa
M
kDa
250
150
250
100
150
75
100
75
50
50
37
37
25
25
20
20
15
15
10
10

## Slide 4
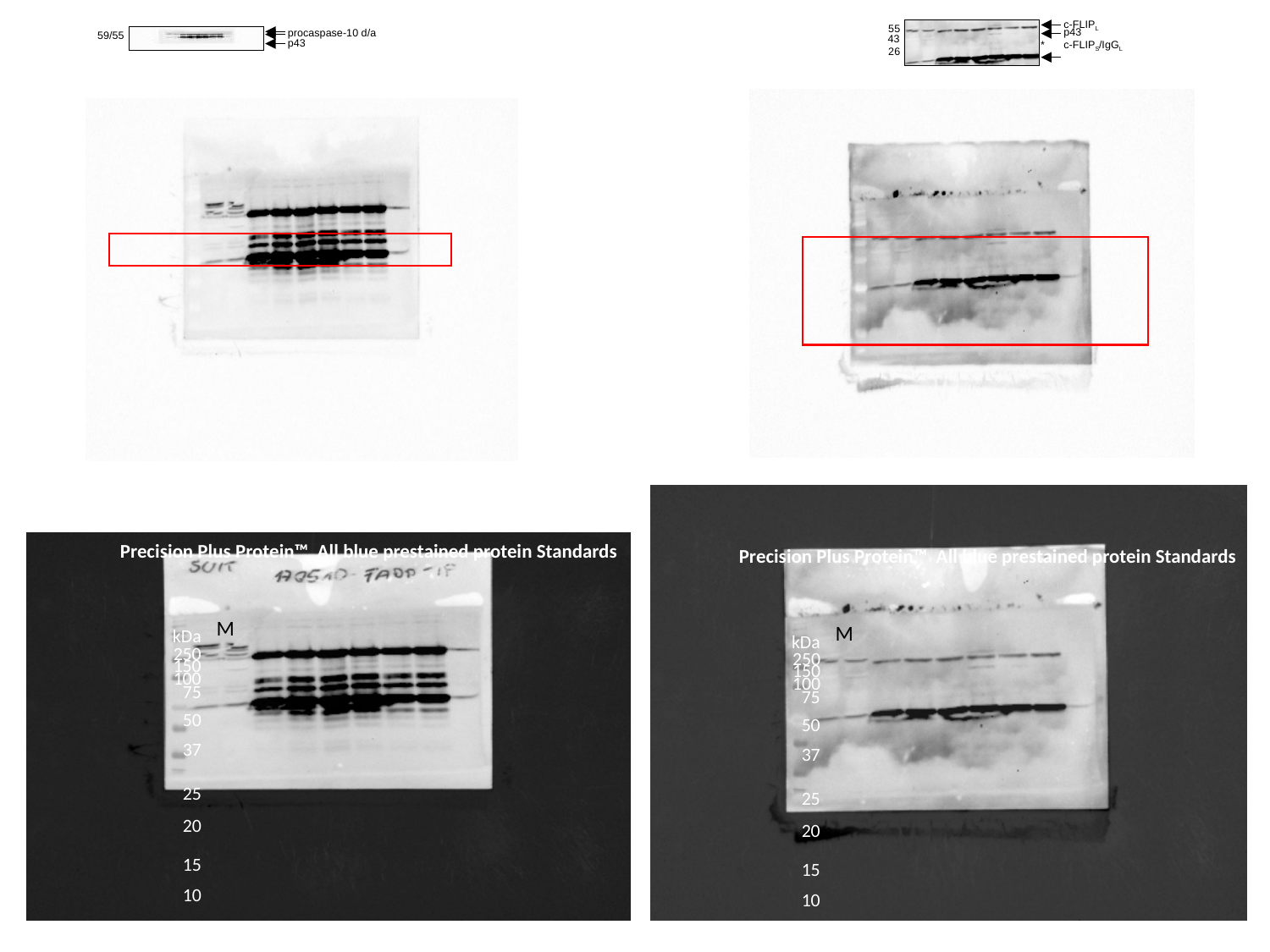

c-FLIPL
55
p43
procaspase-10 d/a
59/55
43
p43
*
c-FLIPS/IgGL
26
Precision Plus Protein™ All blue prestained protein Standards
Precision Plus Protein™ All blue prestained protein Standards
M
M
kDa
kDa
250
250
150
150
100
100
75
75
50
50
37
37
25
25
20
20
15
15
10
10

## Slide 5
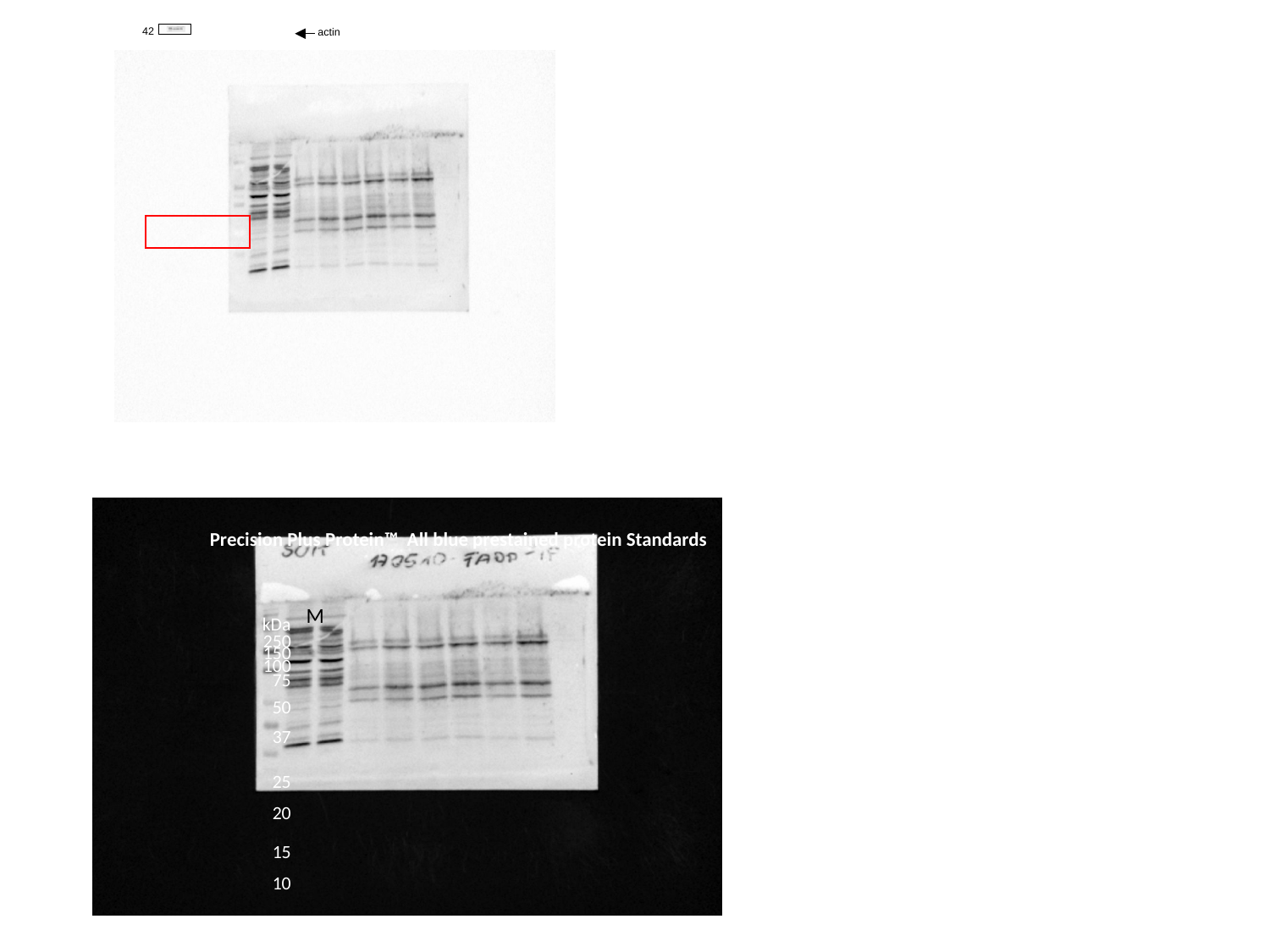

42
actin
Precision Plus Protein™ All blue prestained protein Standards
M
kDa
250
150
100
75
50
37
25
20
15
10
