# Supplementary material for: Oligomerised RIPK1 is the main core component of the CD95 necrosome
Source: EMBO J. 2025 Apr 16;44(11):3231–65. doi: 10.1038/s44318-025-00433-0 (PMC12130296; doi:10.1038/s44318-025-00433-0)
Supplement: Supplementary file 12 — Figure EV3 Source Data [file 44318_2025_433_MOESM12_ESM.zip › EV3C.pptx]

## Slide 1
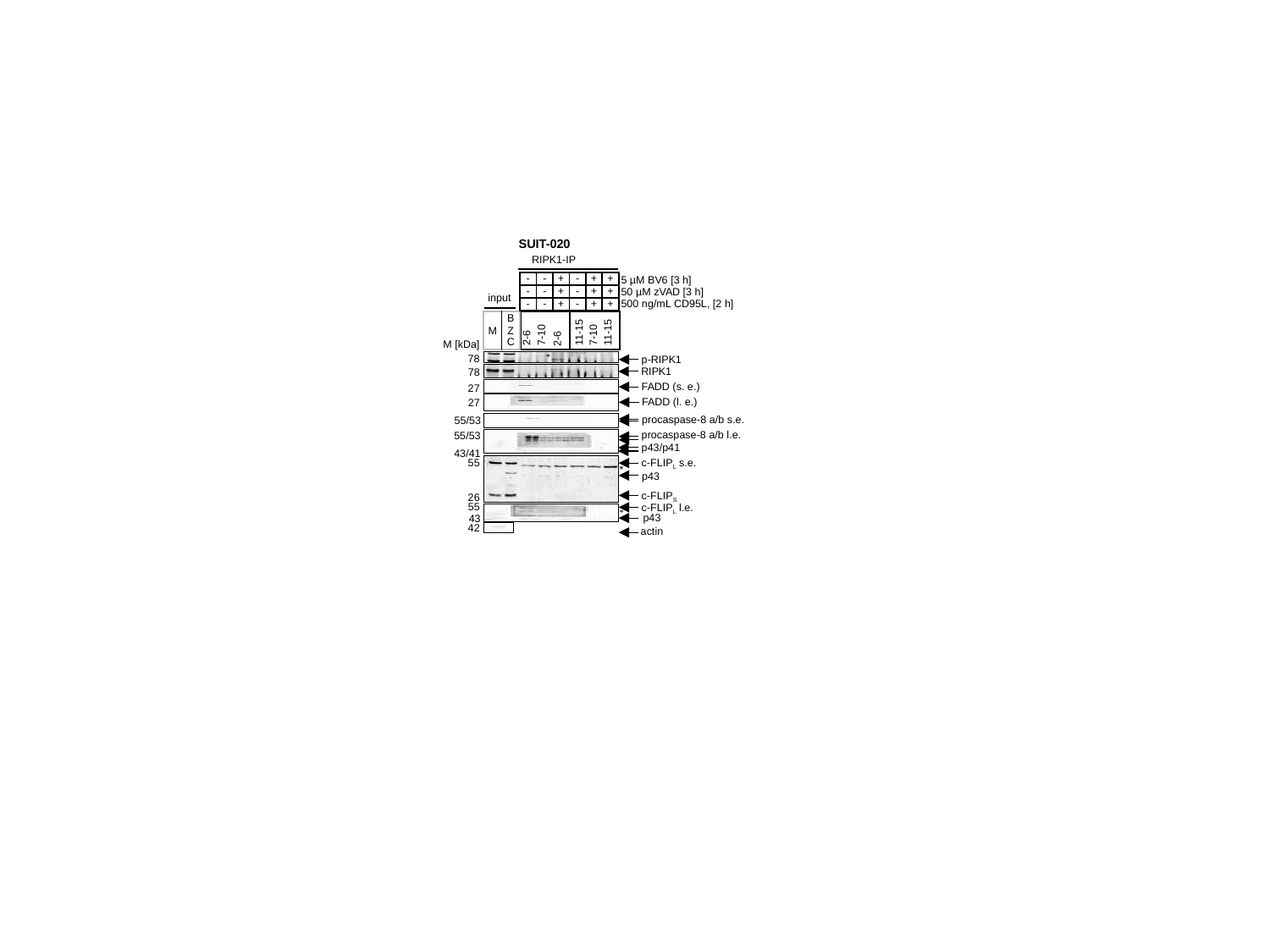

SUIT-020
RIPK1-IP
input
5 µM BV6 [3 h]
| - | - | + | - | + | + |
| --- | --- | --- | --- | --- | --- |
| - | - | + | - | + | + |
| - | - | + | - | + | + |
50 µM zVAD [3 h]
500 ng/mL CD95L, [2 h]
2-6
7-10
| M | B Z C |
| --- | --- |
2-6
11-15
7-10
11-15
M [kDa]
*
78
p-RIPK1
RIPK1
78
FADD (s. e.)
27
FADD (l. e.)
27
procaspase-8 a/b s.e.
55/53
procaspase-8 a/b l.e.
55/53
p43/p41
43/41
55
c-FLIPL s.e.
*
p43
c-FLIPS
26
55
c-FLIPL l.e.
*
p43
43
42
actin

## Slide 2
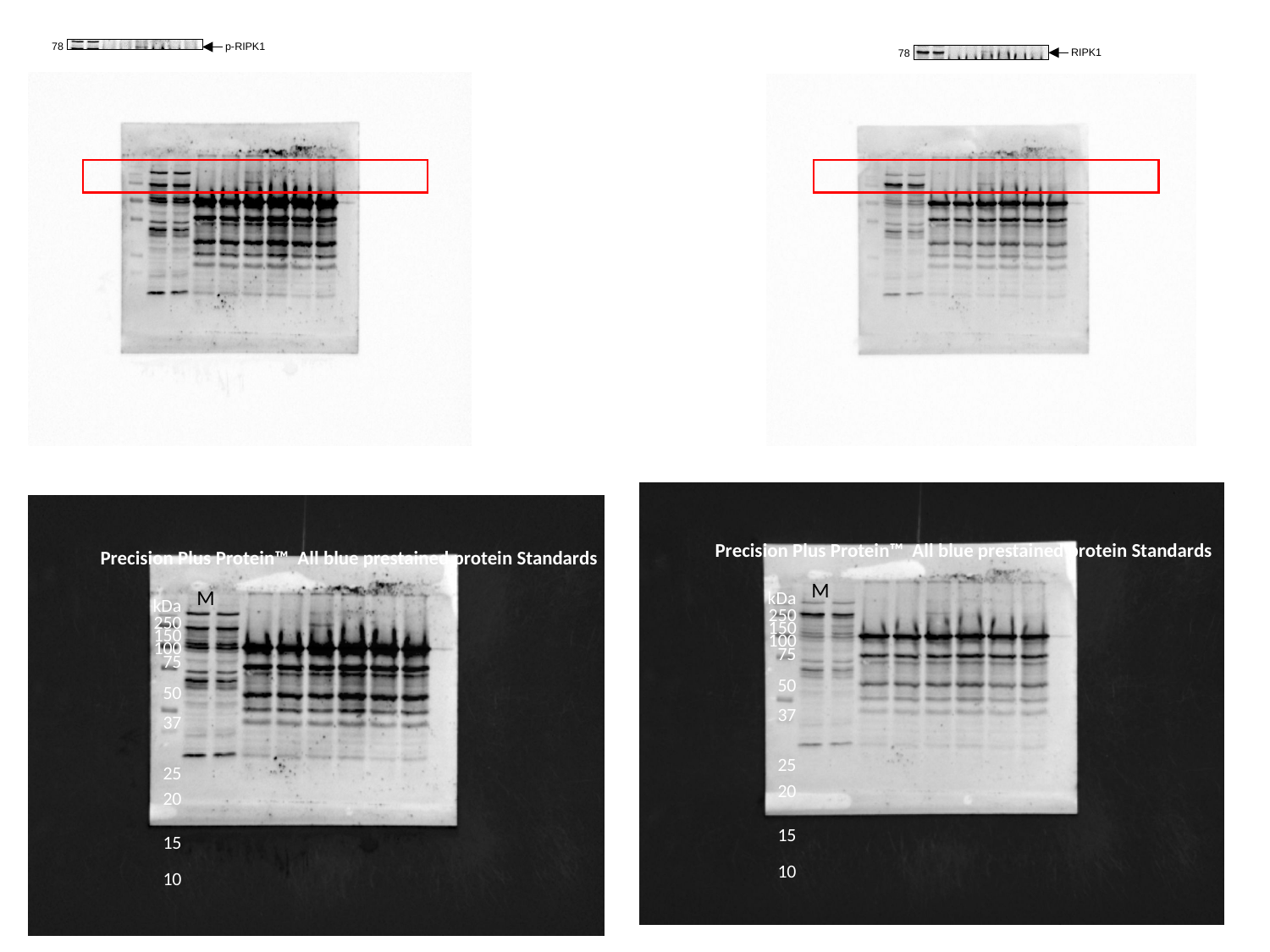

78
p-RIPK1
RIPK1
78
Precision Plus Protein™ All blue prestained protein Standards
Precision Plus Protein™ All blue prestained protein Standards
M
M
kDa
kDa
250
250
150
150
100
100
75
75
50
50
37
37
25
25
20
20
15
15
10
10

## Slide 3
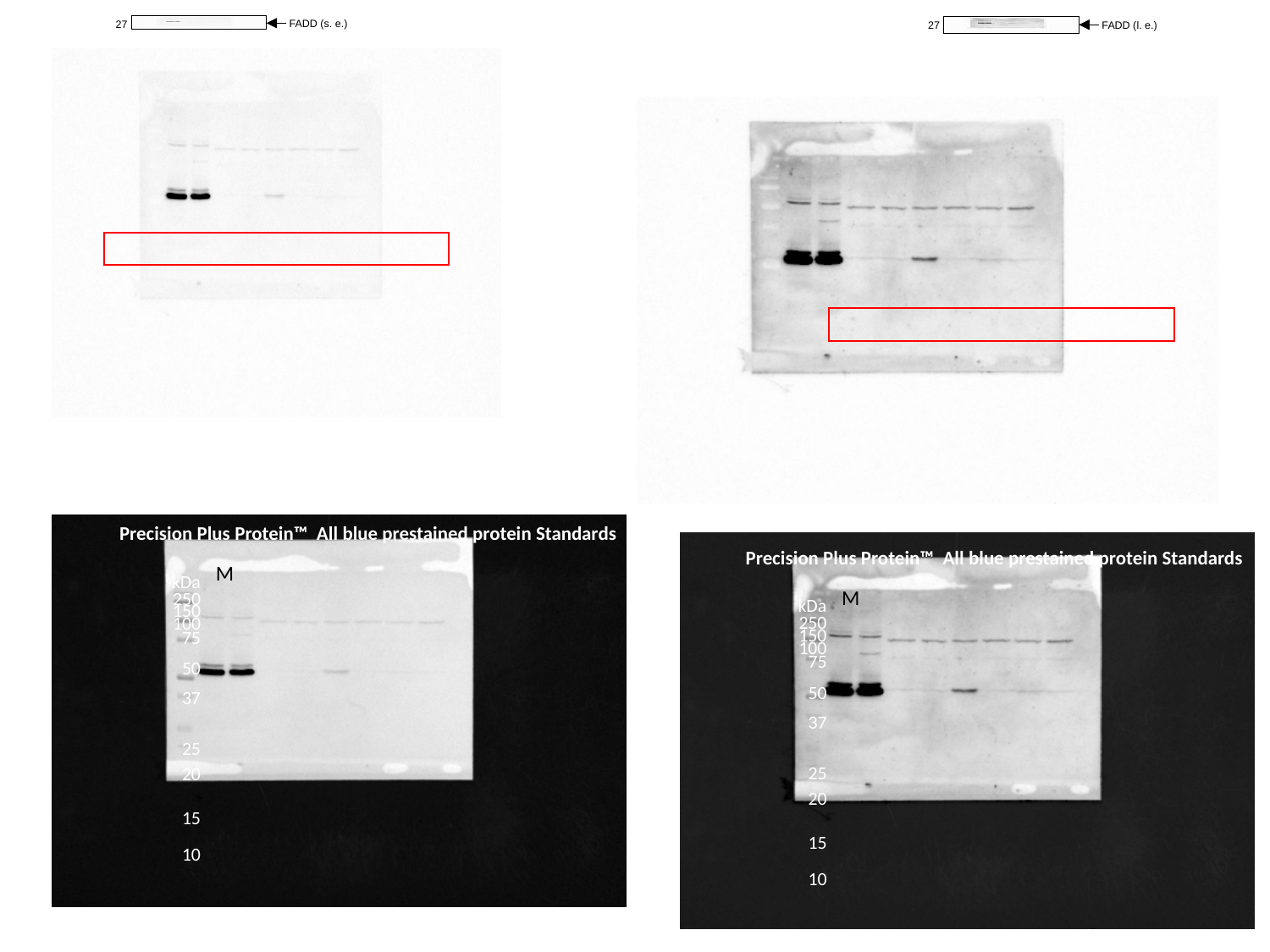

FADD (s. e.)
27
FADD (l. e.)
27
Precision Plus Protein™ All blue prestained protein Standards
Precision Plus Protein™ All blue prestained protein Standards
M
kDa
M
250
kDa
150
250
100
150
75
100
75
50
50
37
37
25
25
20
20
15
15
10
10

## Slide 4
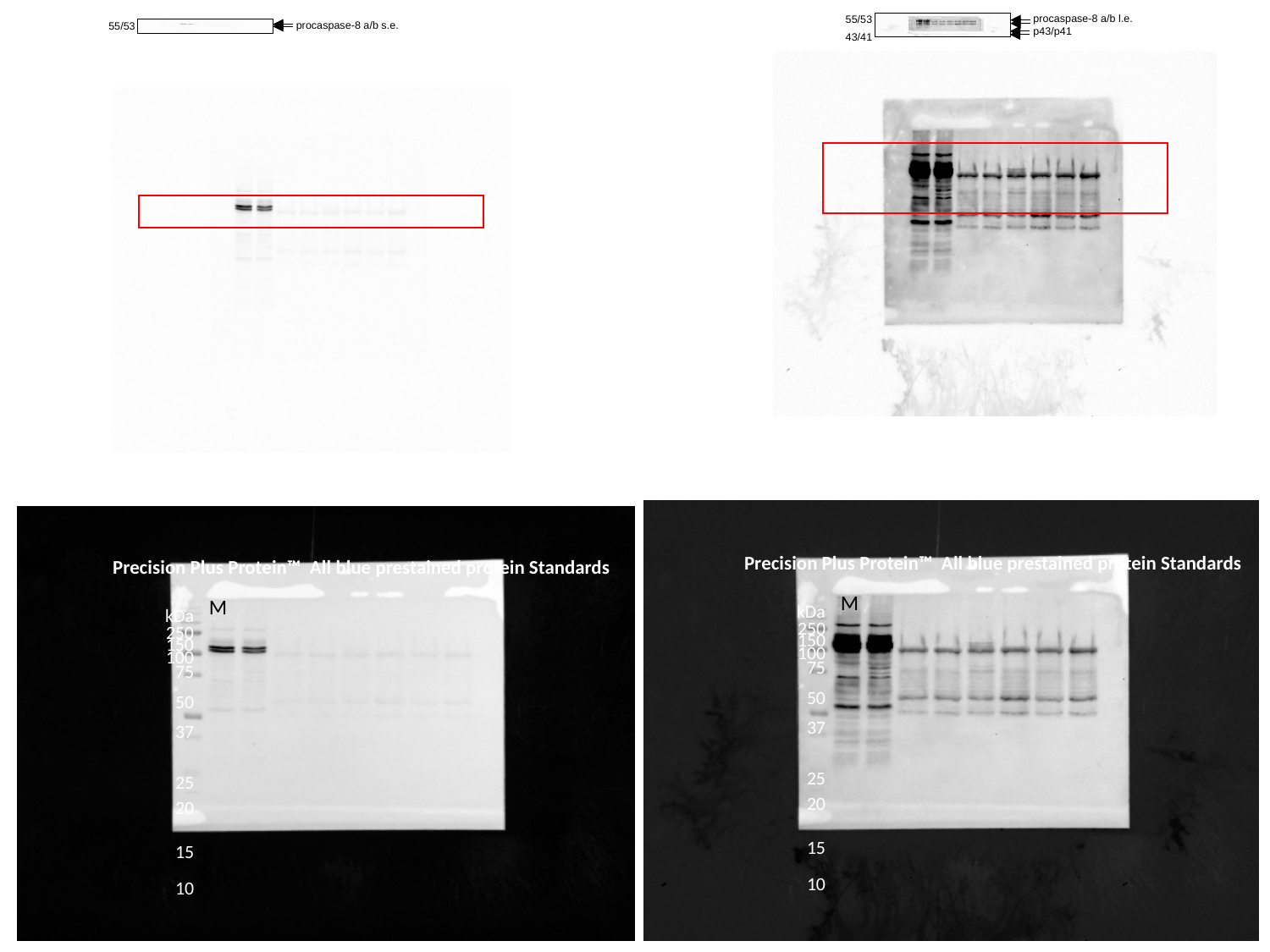

procaspase-8 a/b l.e.
55/53
procaspase-8 a/b s.e.
55/53
p43/p41
43/41
Precision Plus Protein™ All blue prestained protein Standards
Precision Plus Protein™ All blue prestained protein Standards
M
M
kDa
kDa
250
250
150
150
100
100
75
75
50
50
37
37
25
25
20
20
15
15
10
10

## Slide 5
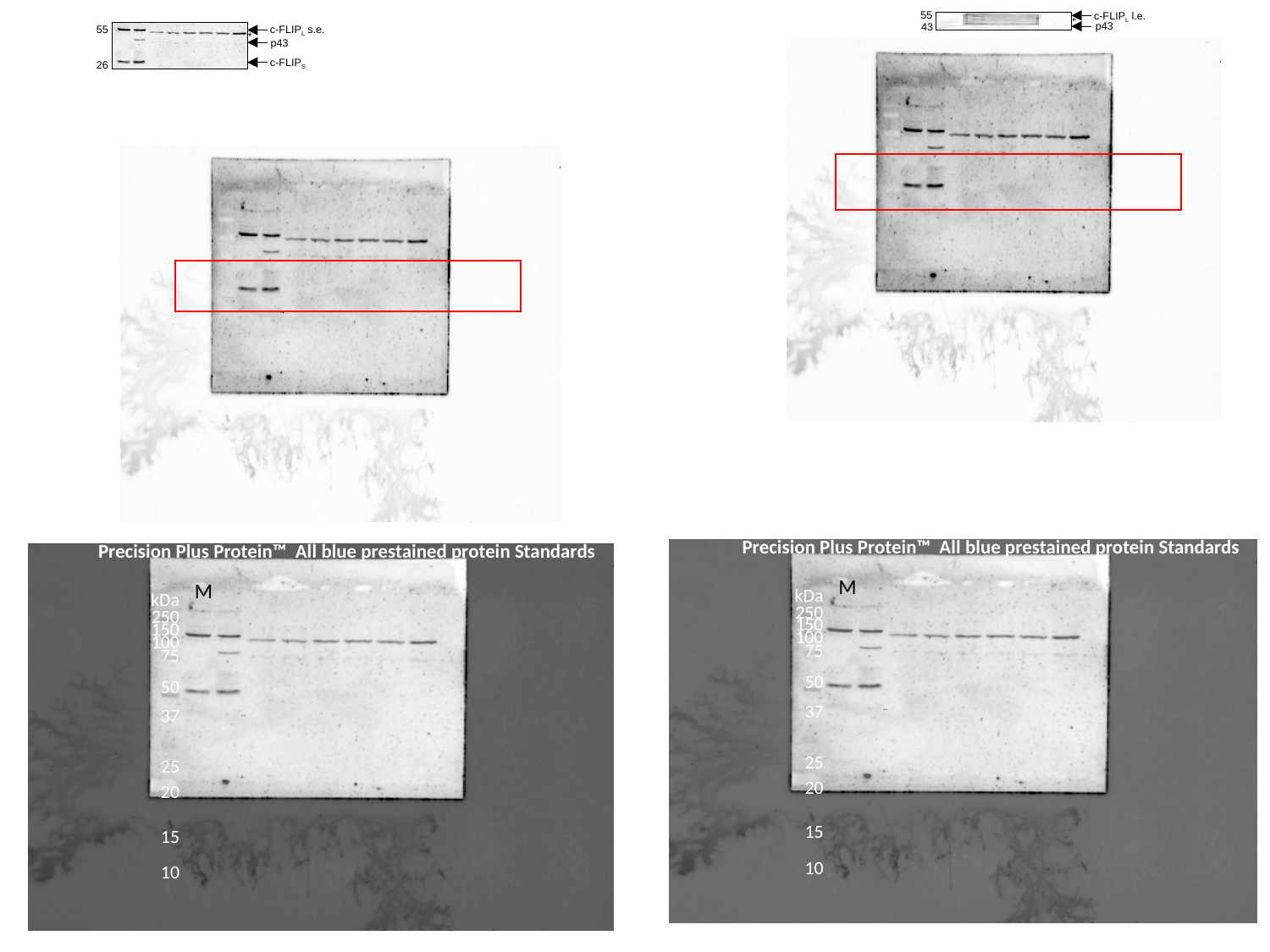

55
c-FLIPL l.e.
*
p43
43
55
c-FLIPL s.e.
*
p43
c-FLIPS
26
Precision Plus Protein™ All blue prestained protein Standards
Precision Plus Protein™ All blue prestained protein Standards
M
M
kDa
kDa
250
250
150
150
100
100
75
75
50
50
37
37
25
25
20
20
15
15
10
10

## Slide 6
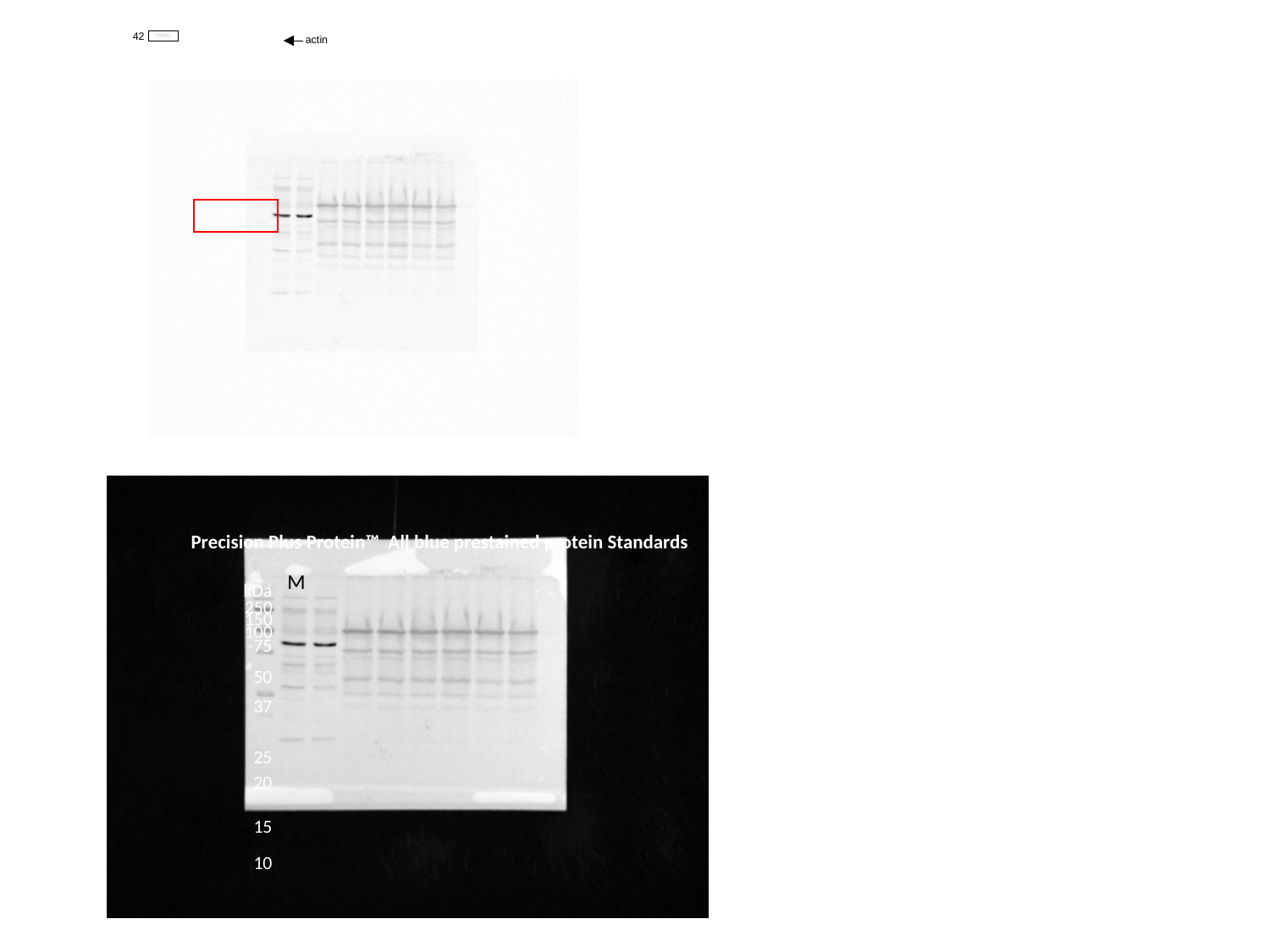

42
actin
Precision Plus Protein™ All blue prestained protein Standards
M
kDa
250
150
100
75
50
37
25
20
15
10
