# Supplementary material for: Oligomerised RIPK1 is the main core component of the CD95 necrosome
Source: EMBO J. 2025 Apr 16;44(11):3231–65. doi: 10.1038/s44318-025-00433-0 (PMC12130296; doi:10.1038/s44318-025-00433-0)
Supplement: Supplementary file 13 — Figure EV5 Source Data [file 44318_2025_433_MOESM13_ESM.zip › EV5E.pptx]

## Slide 1
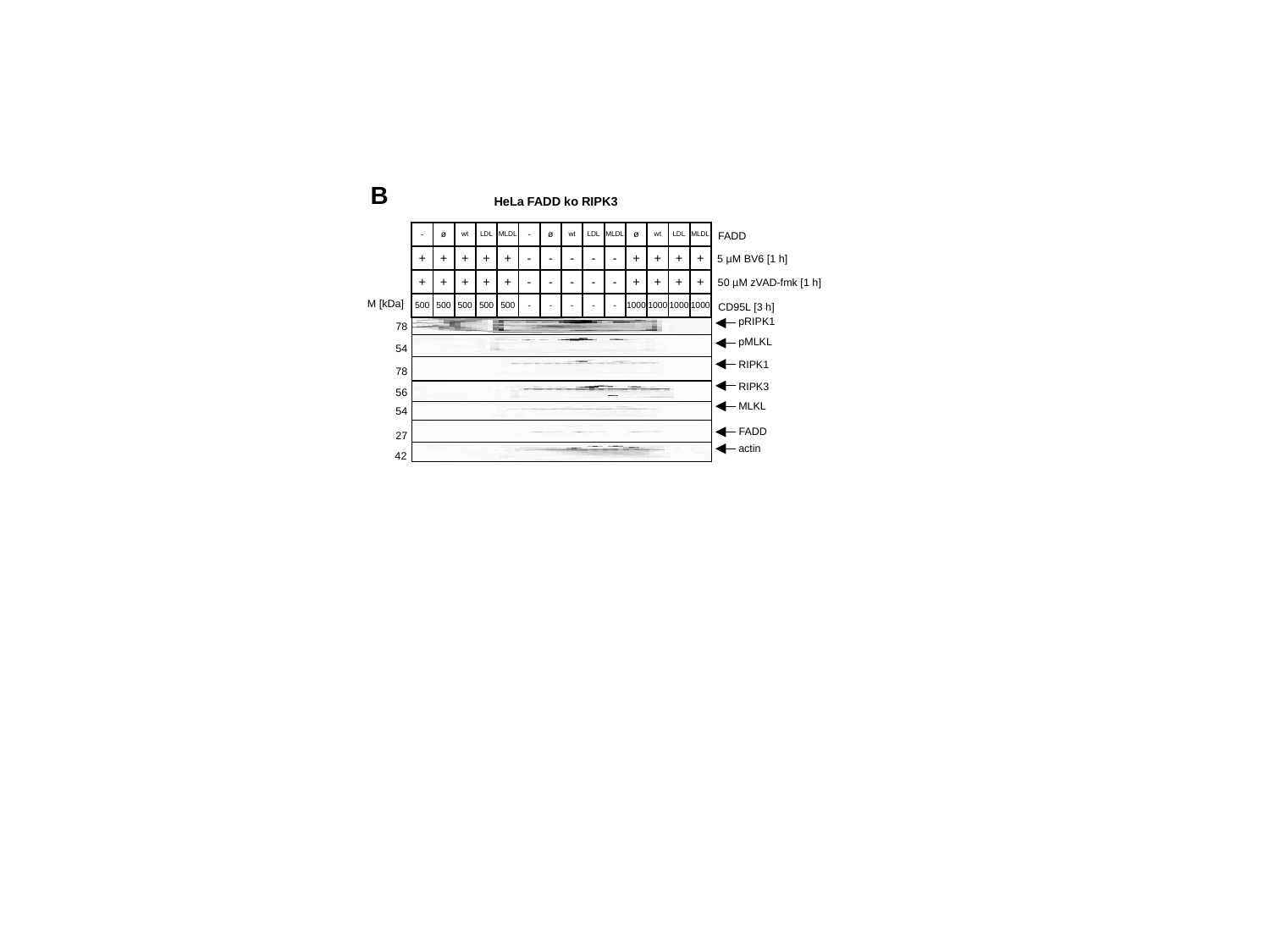

B
HeLa FADD ko RIPK3
| - | ø | wt | LDL | MLDL | - | ø | wt | LDL | MLDL | ø | wt | LDL | MLDL |
| --- | --- | --- | --- | --- | --- | --- | --- | --- | --- | --- | --- | --- | --- |
| + | + | + | + | + | - | - | - | - | - | + | + | + | + |
| + | + | + | + | + | - | - | - | - | - | + | + | + | + |
| 500 | 500 | 500 | 500 | 500 | - | - | - | - | - | 1000 | 1000 | 1000 | 1000 |
FADD
5 µM BV6 [1 h]
50 µM zVAD-fmk [1 h]
M [kDa]
CD95L [3 h]
pRIPK1
78
pMLKL
54
RIPK1
78
RIPK3
56
MLKL
54
FADD
27
actin
42

## Slide 2
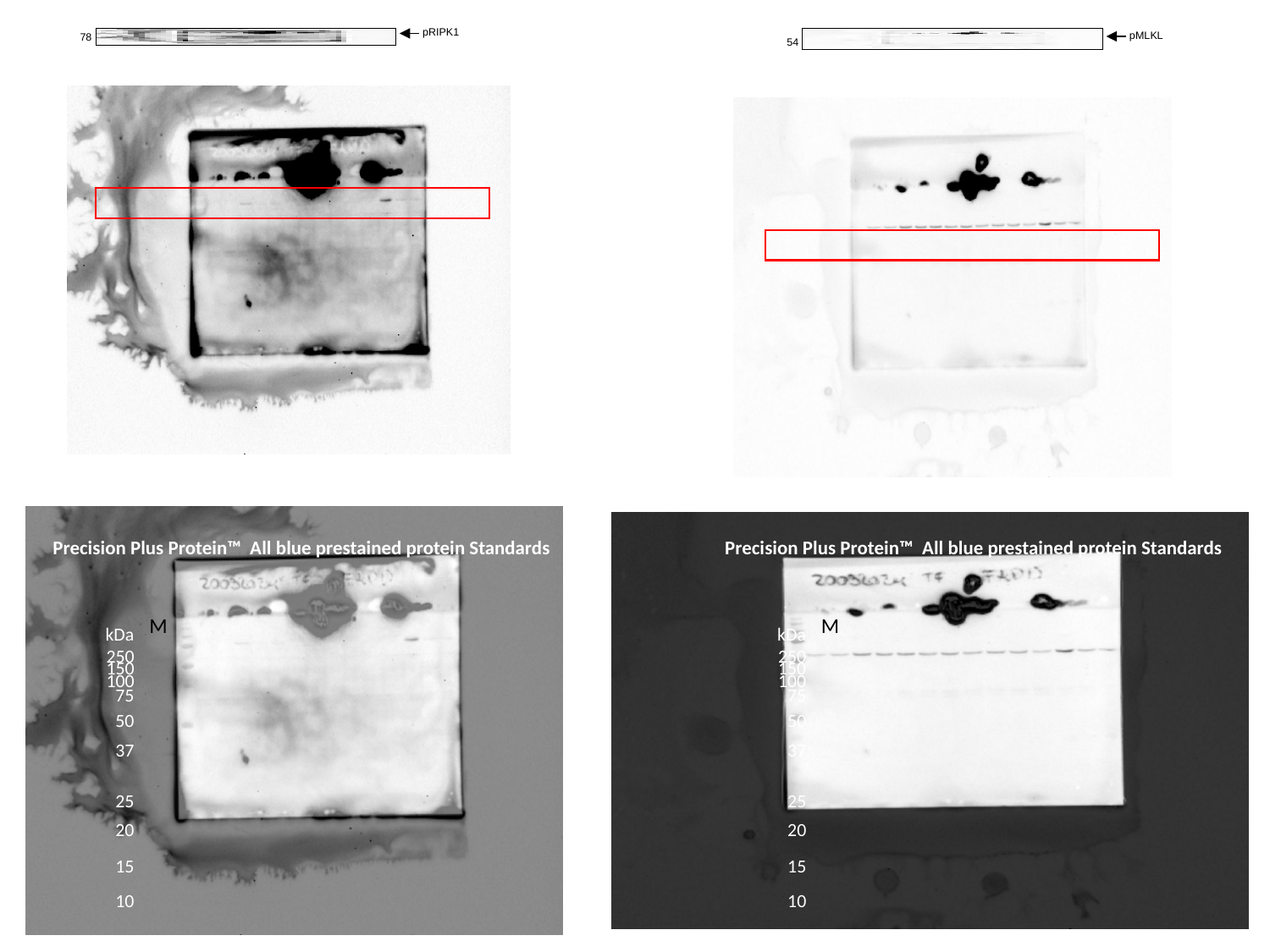

pRIPK1
pMLKL
78
54
Precision Plus Protein™ All blue prestained protein Standards
Precision Plus Protein™ All blue prestained protein Standards
M
M
kDa
kDa
250
250
150
150
100
100
75
75
50
50
37
37
25
25
20
20
15
15
10
10

## Slide 3
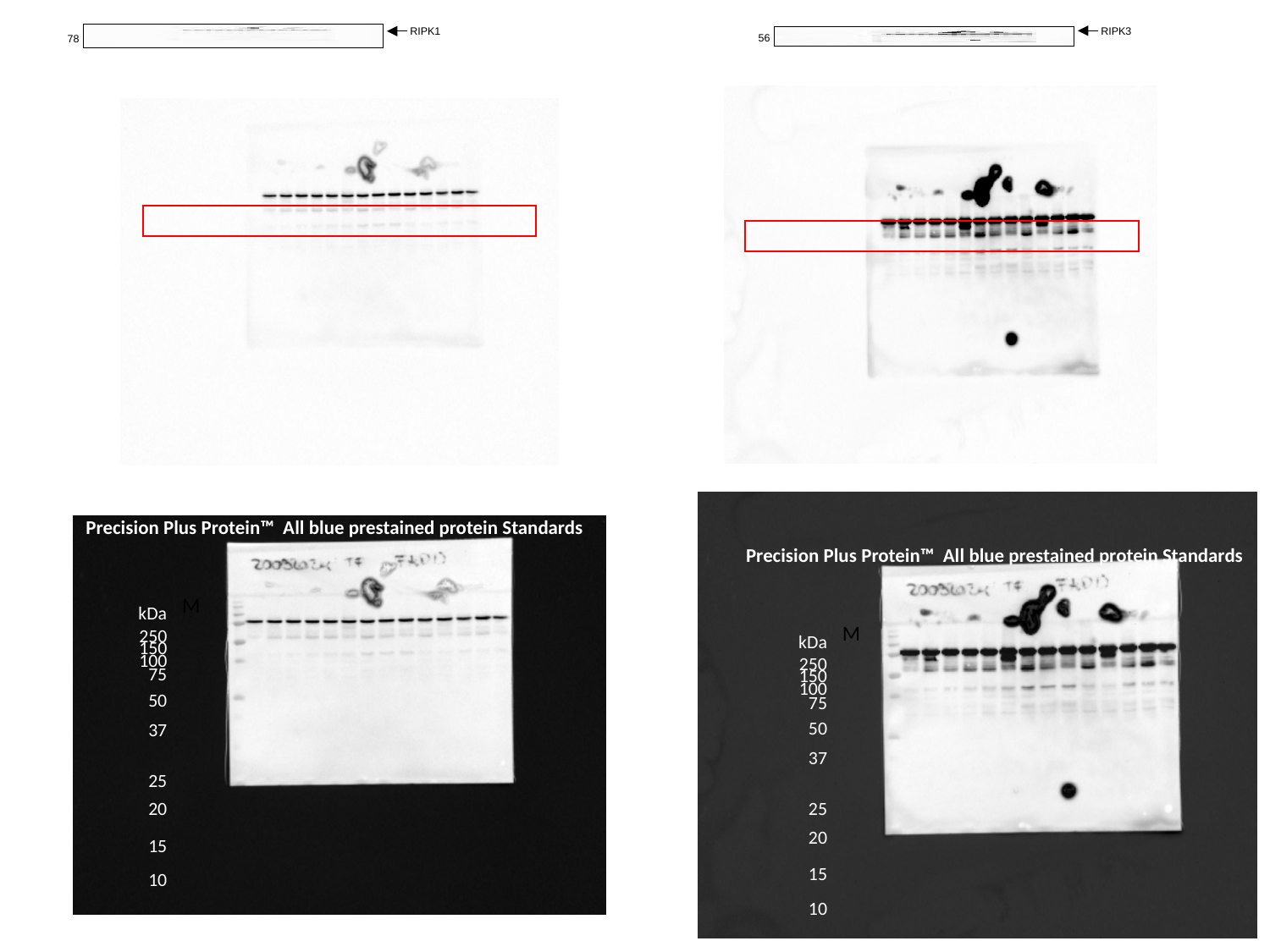

RIPK3
RIPK1
56
78
Precision Plus Protein™ All blue prestained protein Standards
Precision Plus Protein™ All blue prestained protein Standards
M
kDa
M
250
kDa
150
100
250
75
150
100
50
75
50
37
37
25
25
20
20
15
15
10
10

## Slide 4
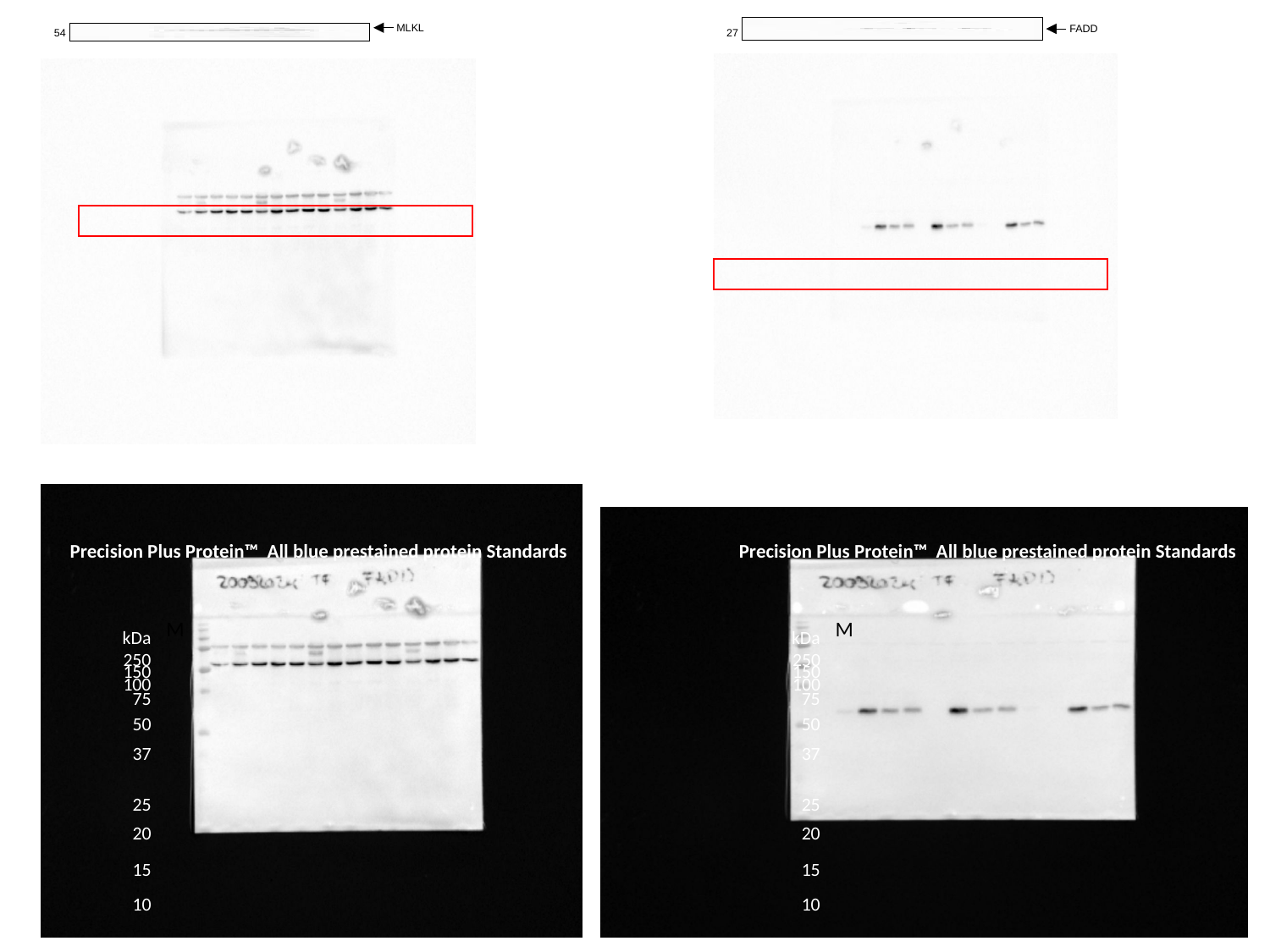

MLKL
FADD
27
54
Precision Plus Protein™ All blue prestained protein Standards
Precision Plus Protein™ All blue prestained protein Standards
M
M
kDa
kDa
250
250
150
150
100
100
75
75
50
50
37
37
25
25
20
20
15
15
10
10

## Slide 5
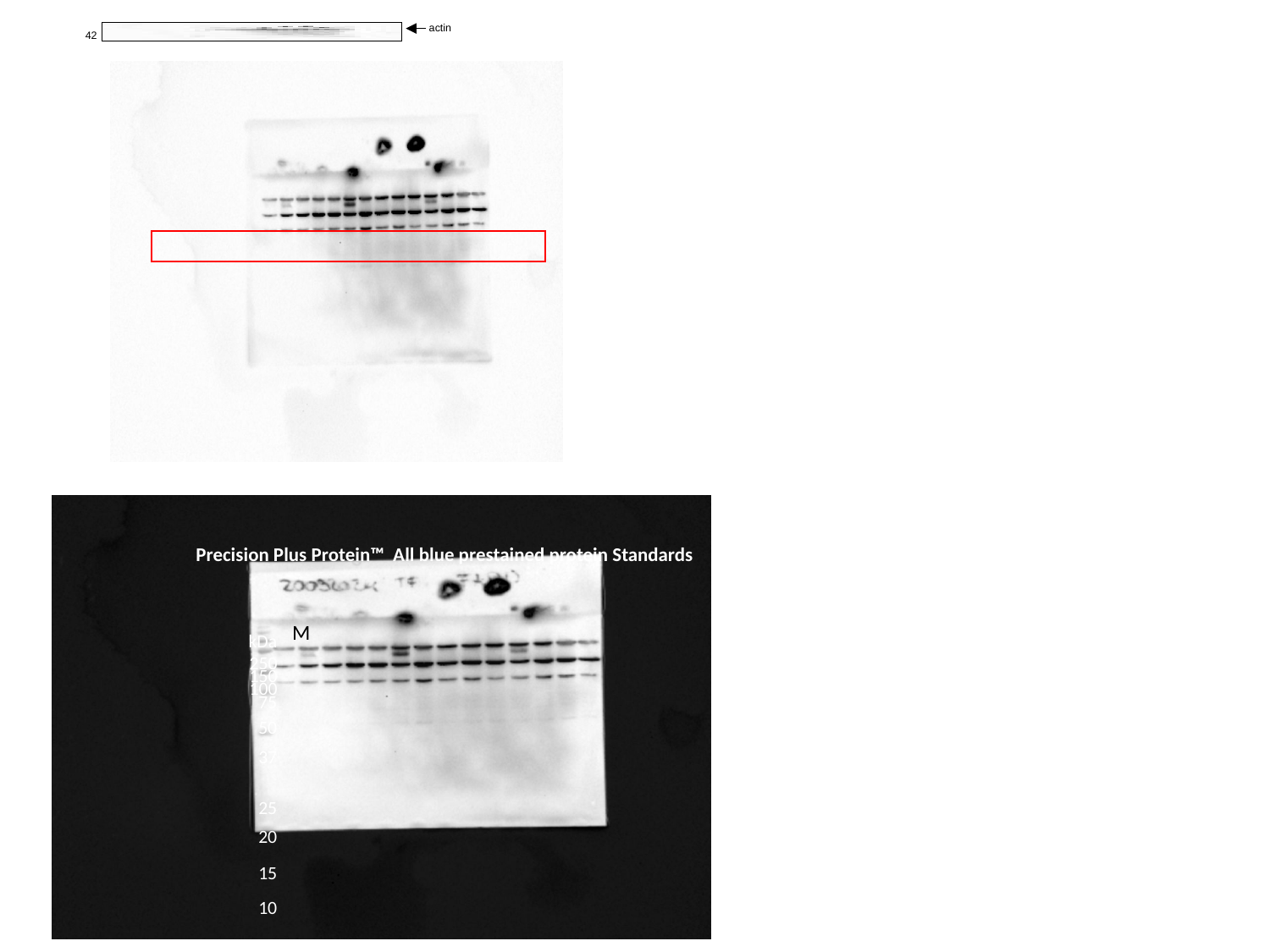

actin
42
Precision Plus Protein™ All blue prestained protein Standards
M
kDa
250
150
100
75
50
37
25
20
15
10
