# Supplementary material for: Oligomerised RIPK1 is the main core component of the CD95 necrosome
Source: EMBO J. 2025 Apr 16;44(11):3231–65. doi: 10.1038/s44318-025-00433-0 (PMC12130296; doi:10.1038/s44318-025-00433-0)
Supplement: Supplementary file 14 — Appendix Source Data [file 44318_2025_433_MOESM14_ESM.zip › S1A.pptx]

## Slide 1
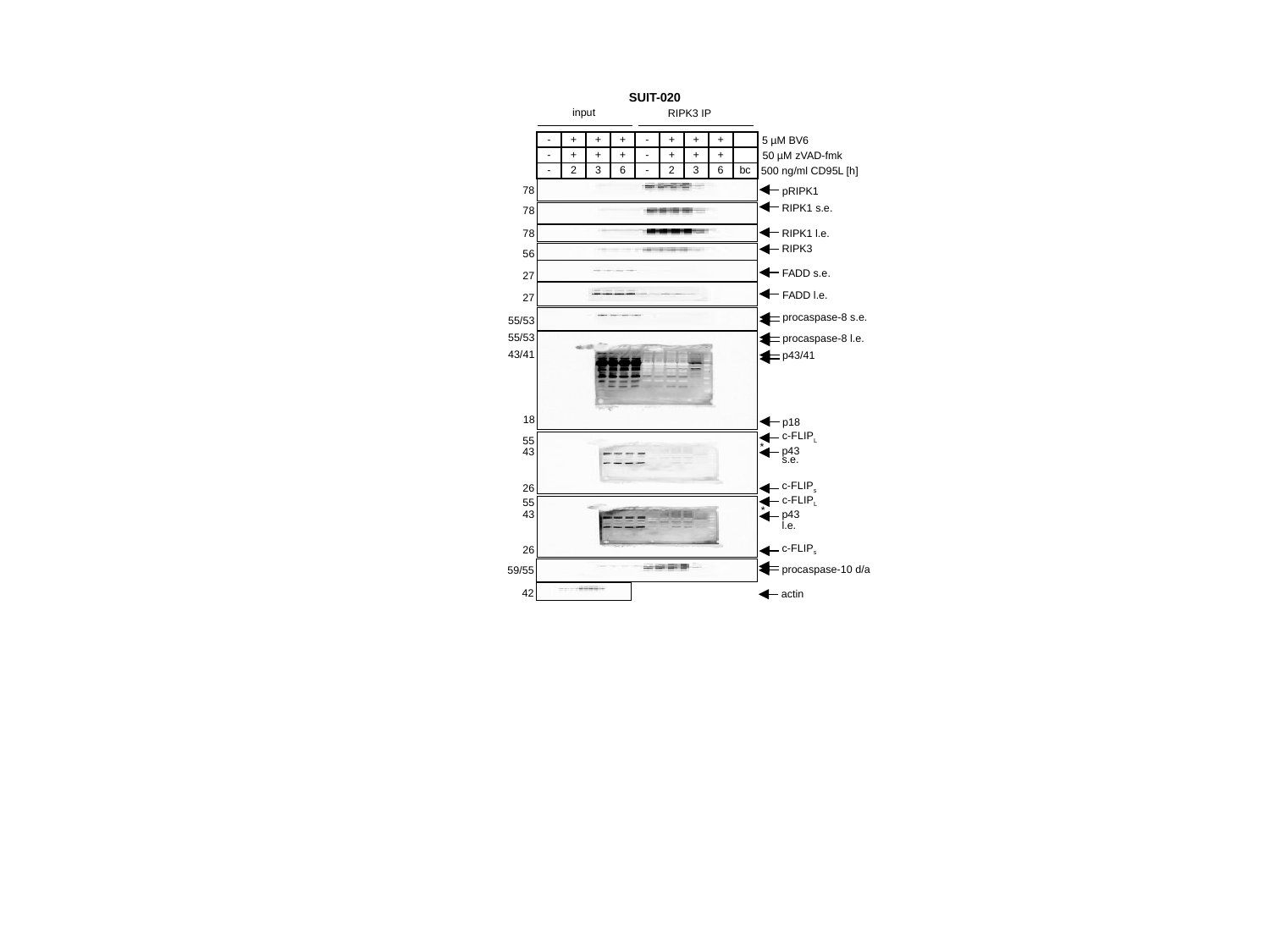

SUIT-020
input
RIPK3 IP
5 µM BV6
| - | + | + | + | - | + | + | + | |
| --- | --- | --- | --- | --- | --- | --- | --- | --- |
| - | + | + | + | - | + | + | + | |
| - | 2 | 3 | 6 | - | 2 | 3 | 6 | bc |
50 µM zVAD-fmk
500 ng/ml CD95L [h]
78
pRIPK1
RIPK1 s.e.
78
78
RIPK1 l.e.
RIPK3
56
FADD s.e.
27
FADD l.e.
27
procaspase-8 s.e.
55/53
55/53
procaspase-8 l.e.
43/41
p43/41
18
p18
c-FLIPL
55
*
p43
43
s.e.
c-FLIPs
26
c-FLIPL
55
*
43
p43
l.e.
c-FLIPs
26
procaspase-10 d/a
59/55
42
actin

## Slide 2
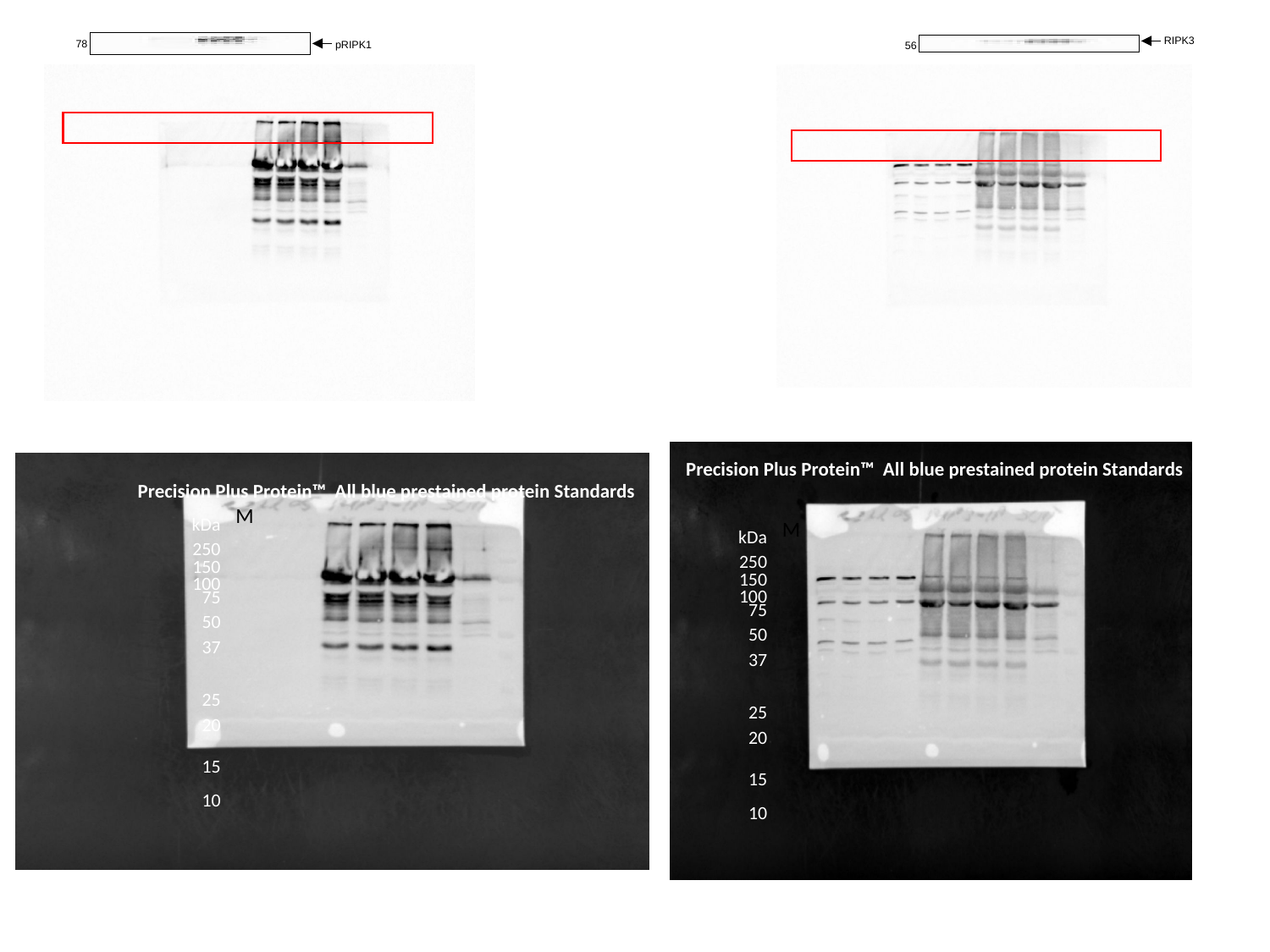

RIPK3
78
pRIPK1
56
Precision Plus Protein™ All blue prestained protein Standards
Precision Plus Protein™ All blue prestained protein Standards
M
kDa
M
kDa
250
250
150
150
100
100
75
75
50
50
37
37
25
25
20
20
15
15
10
10

## Slide 3
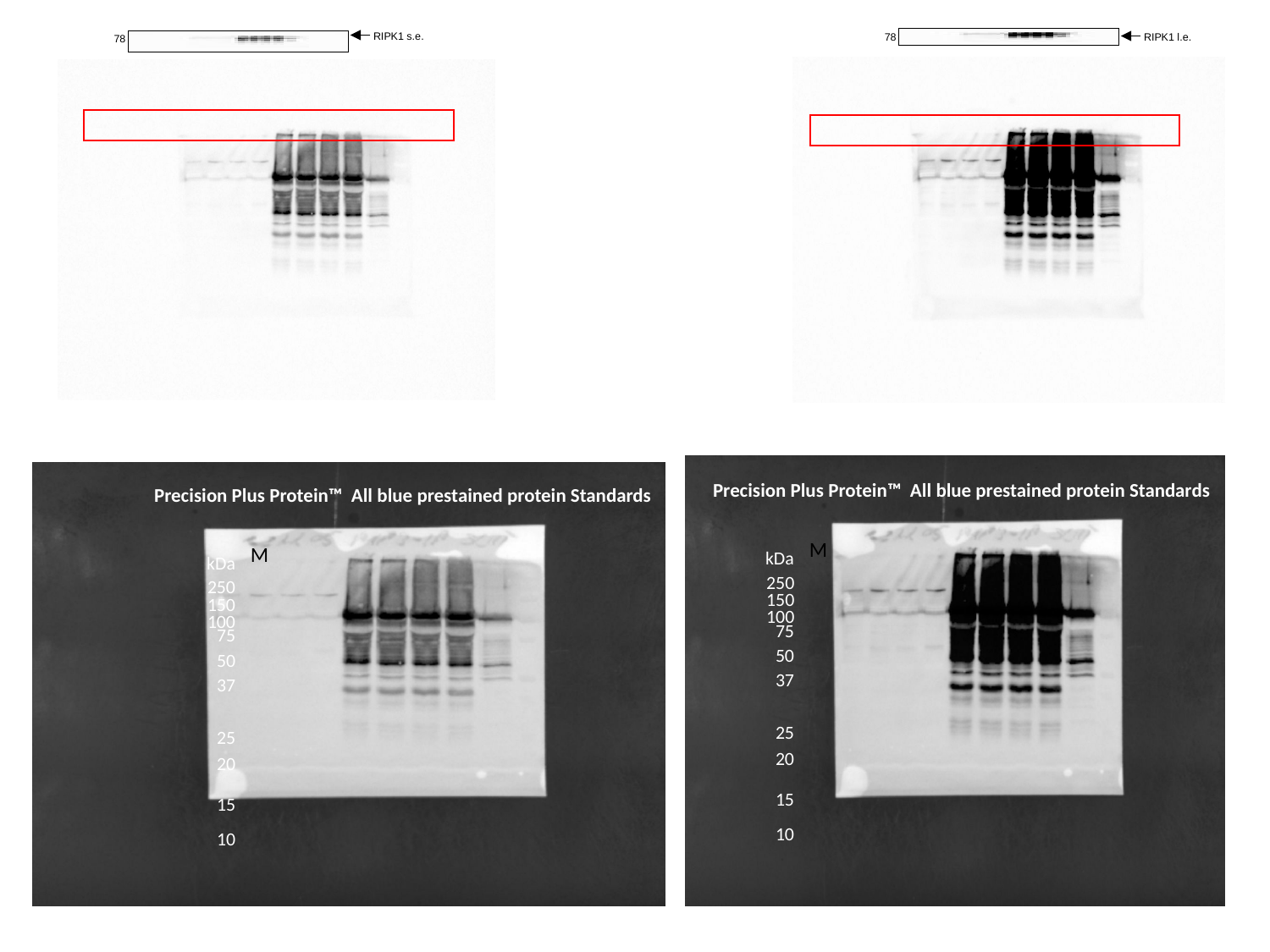

RIPK1 s.e.
78
RIPK1 l.e.
78
Precision Plus Protein™ All blue prestained protein Standards
Precision Plus Protein™ All blue prestained protein Standards
M
M
kDa
kDa
250
250
150
150
100
100
75
75
50
50
37
37
25
25
20
20
15
15
10
10

## Slide 4
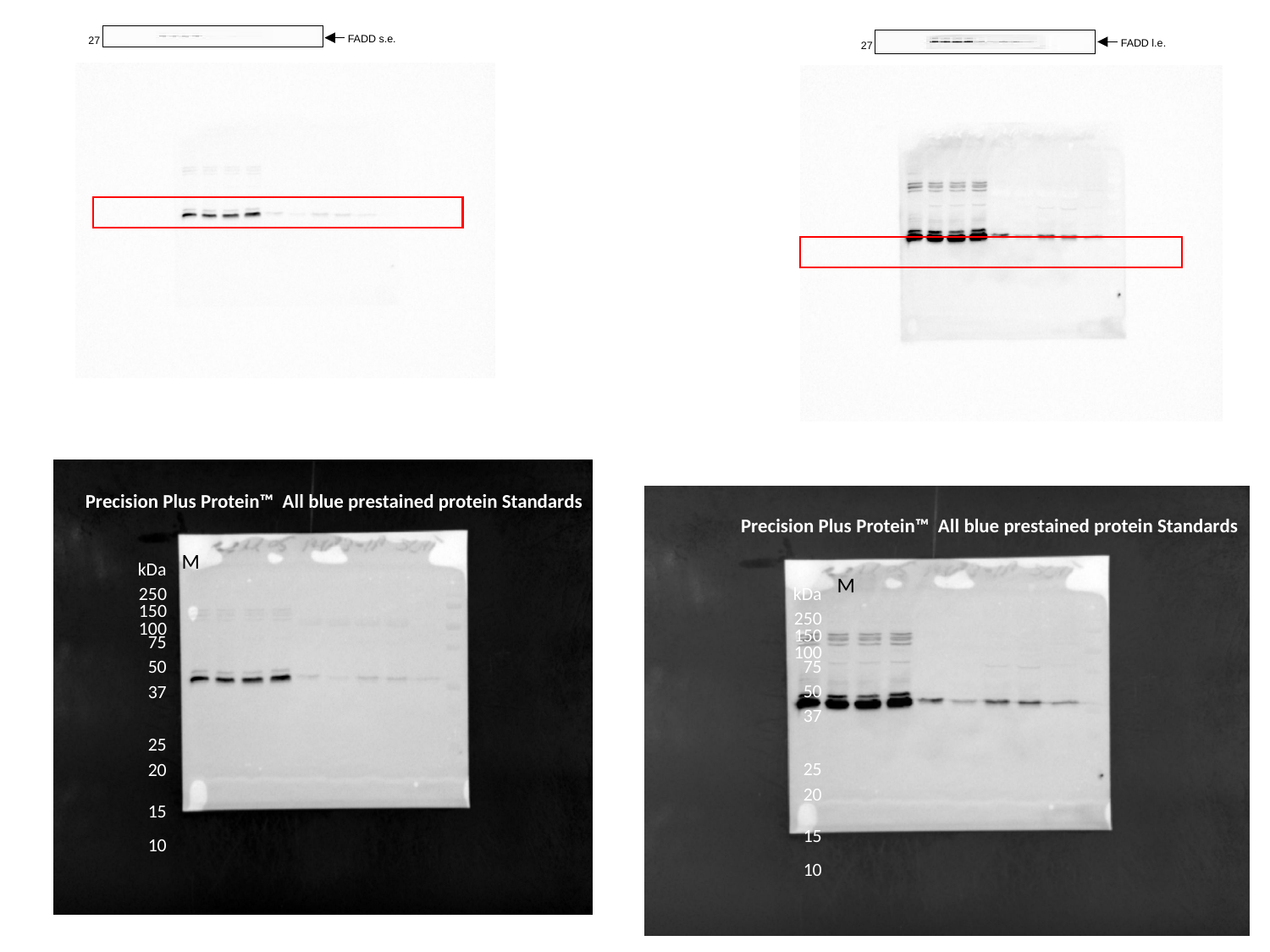

FADD s.e.
27
FADD l.e.
27
Precision Plus Protein™ All blue prestained protein Standards
Precision Plus Protein™ All blue prestained protein Standards
M
kDa
M
250
kDa
150
250
100
150
75
100
75
50
50
37
37
25
25
20
20
15
15
10
10

## Slide 5
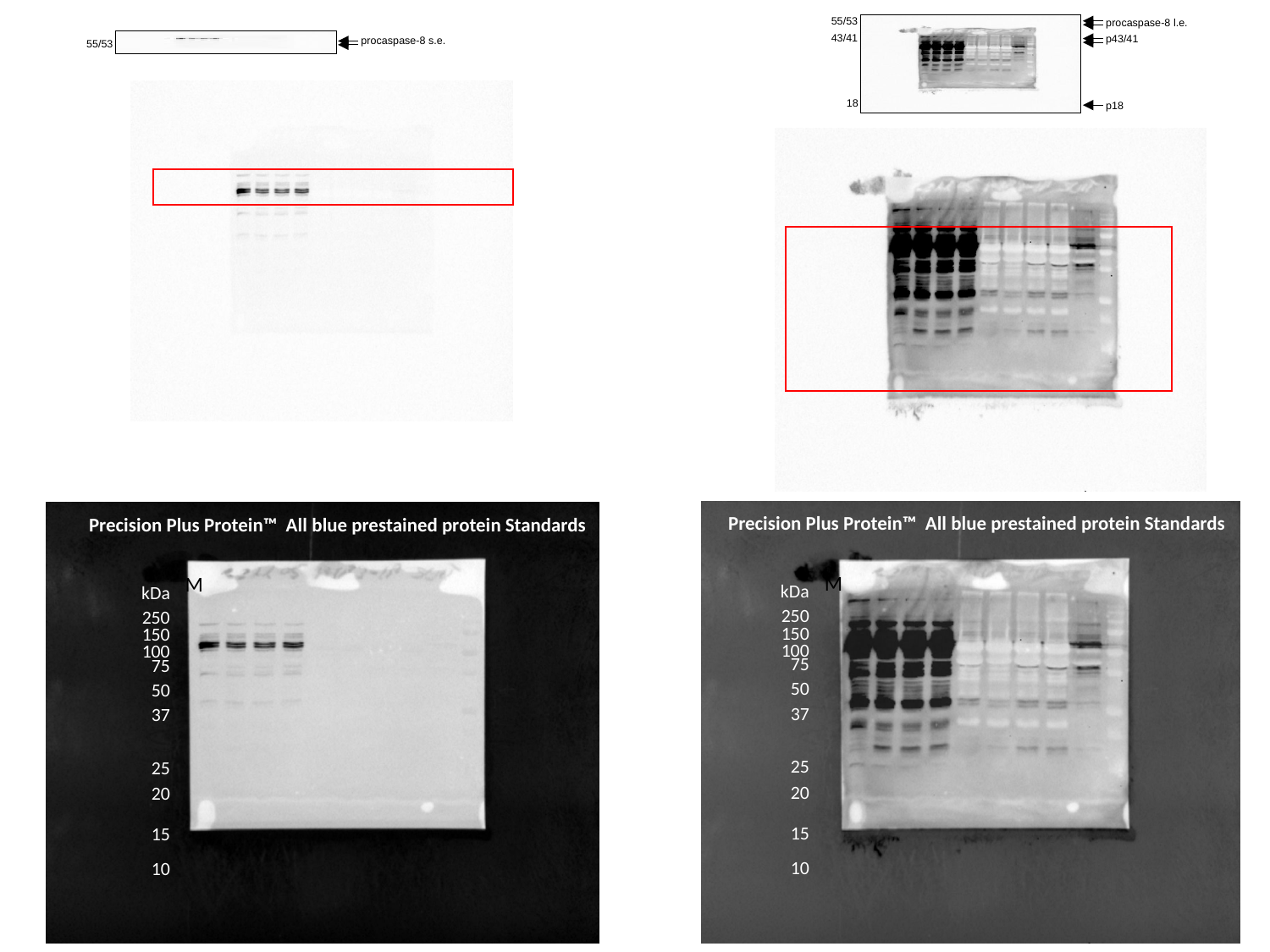

55/53
procaspase-8 l.e.
43/41
p43/41
procaspase-8 s.e.
55/53
18
p18
Precision Plus Protein™ All blue prestained protein Standards
Precision Plus Protein™ All blue prestained protein Standards
M
M
kDa
kDa
250
250
150
150
100
100
75
75
50
50
37
37
25
25
20
20
15
15
10
10

## Slide 6
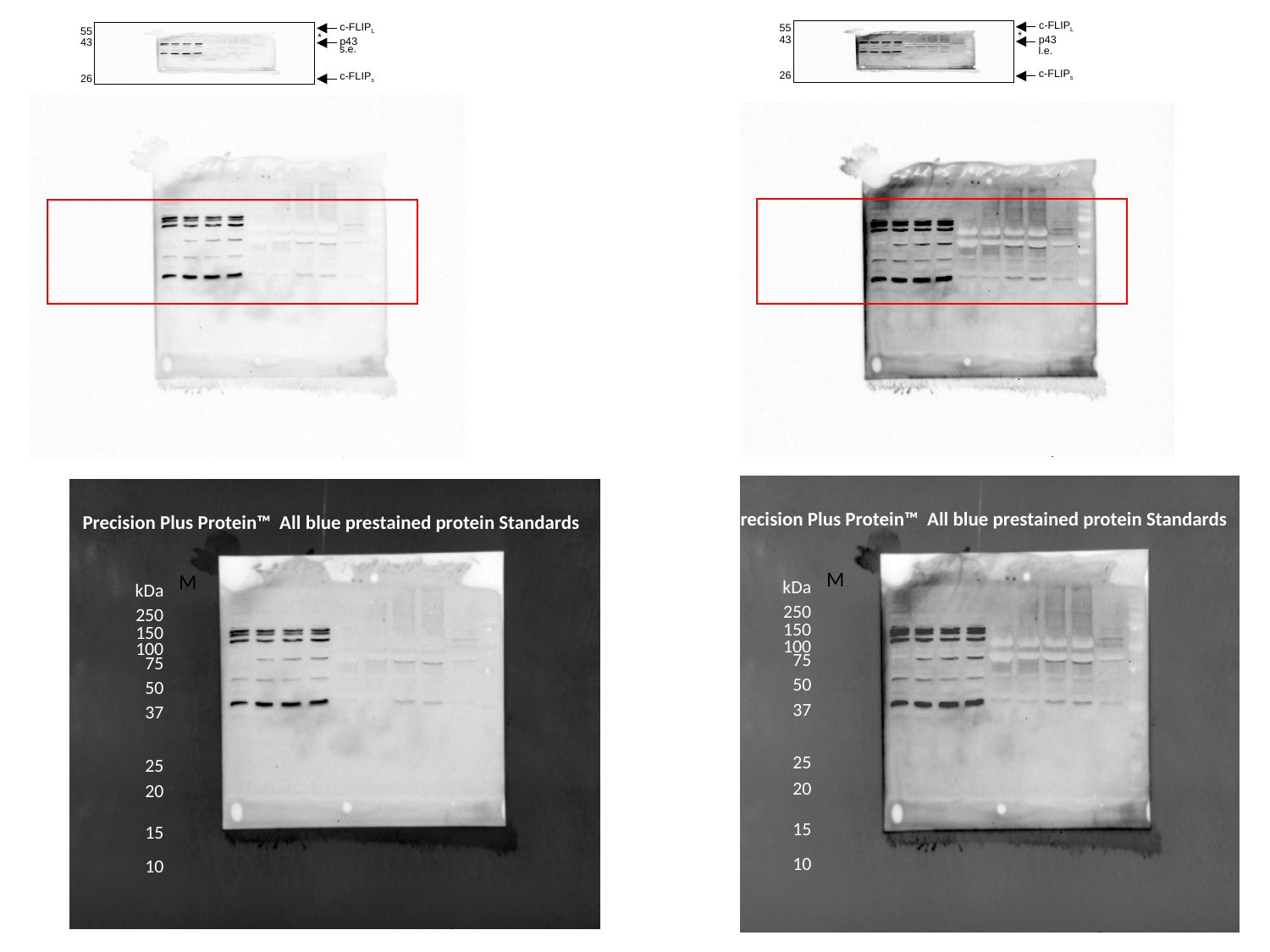

c-FLIPL
c-FLIPL
55
55
*
*
43
p43
p43
43
s.e.
l.e.
c-FLIPs
26
c-FLIPs
26
Precision Plus Protein™ All blue prestained protein Standards
Precision Plus Protein™ All blue prestained protein Standards
M
M
kDa
kDa
250
250
150
150
100
100
75
75
50
50
37
37
25
25
20
20
15
15
10
10

## Slide 7
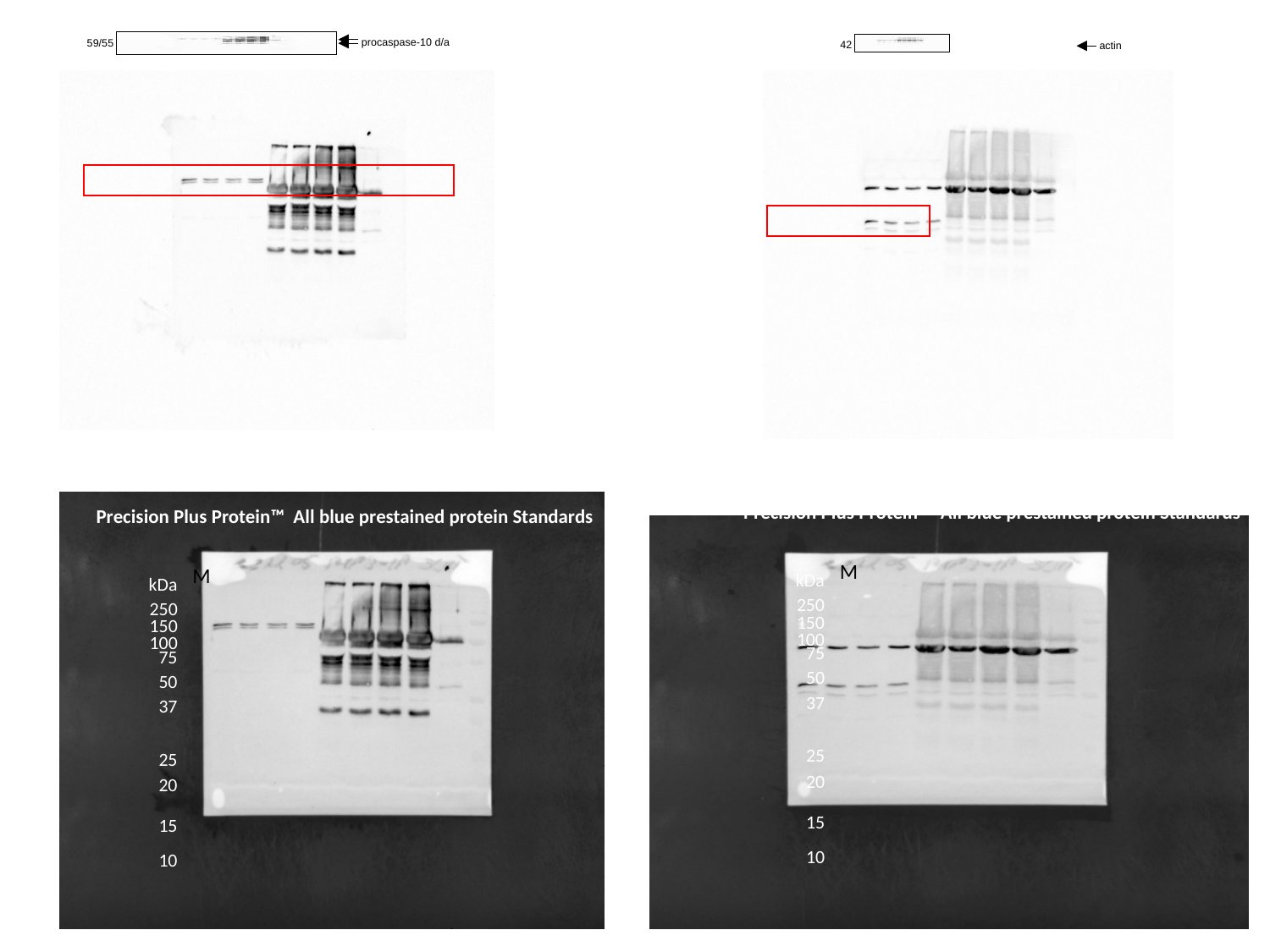

procaspase-10 d/a
59/55
42
actin
Precision Plus Protein™ All blue prestained protein Standards
Precision Plus Protein™ All blue prestained protein Standards
M
M
kDa
kDa
250
250
150
150
100
100
75
75
50
50
37
37
25
25
20
20
15
15
10
10
