# Supplementary material for: Oligomerised RIPK1 is the main core component of the CD95 necrosome
Source: EMBO J. 2025 Apr 16;44(11):3231–65. doi: 10.1038/s44318-025-00433-0 (PMC12130296; doi:10.1038/s44318-025-00433-0)
Supplement: Supplementary file 14 — Appendix Source Data [file 44318_2025_433_MOESM14_ESM.zip › S1B.pptx]

## Slide 1
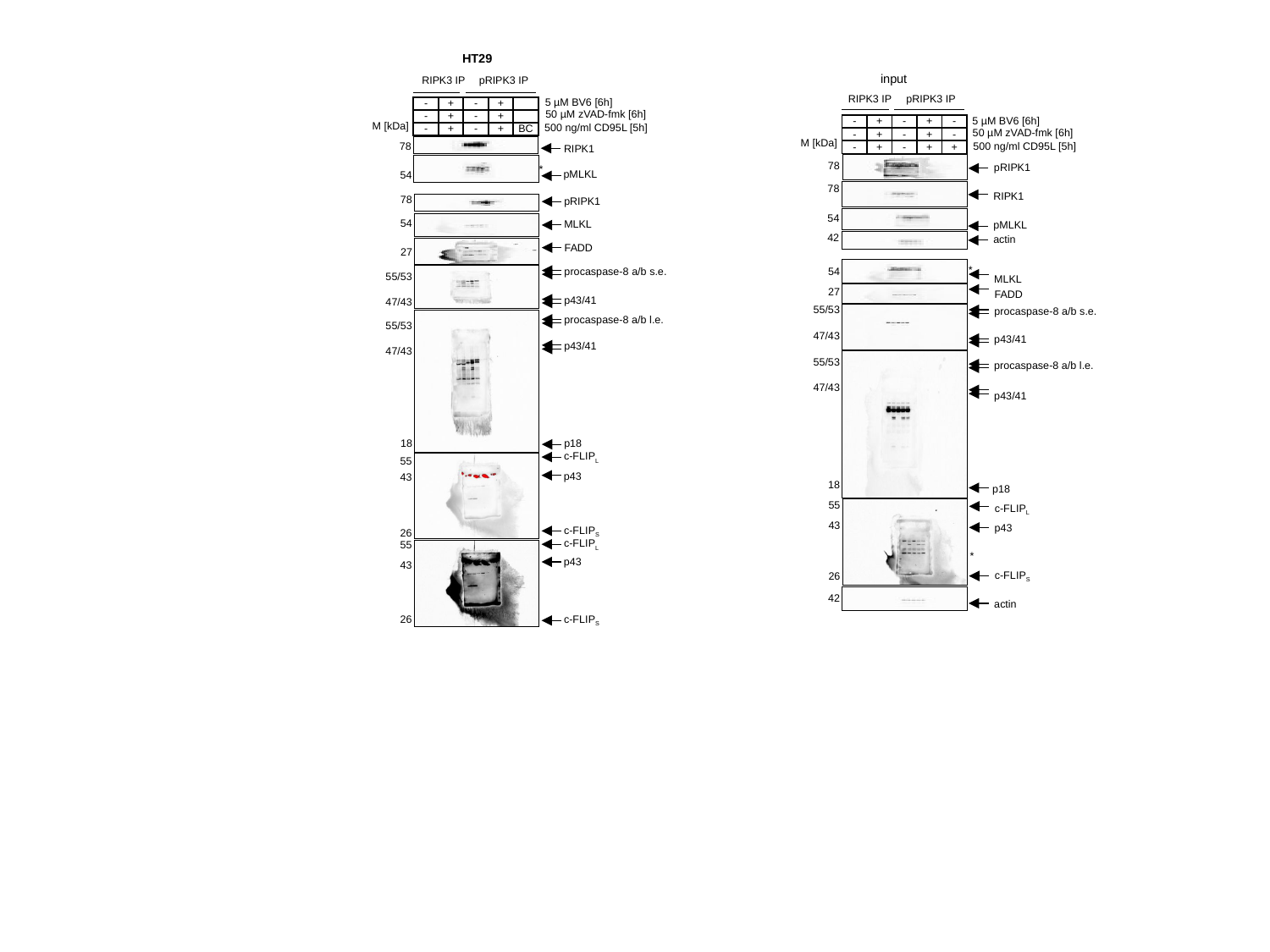

HT29
input
RIPK3 IP
pRIPK3 IP
RIPK3 IP
pRIPK3 IP
5 µM BV6 [6h]
| - | + | - | + | |
| --- | --- | --- | --- | --- |
| - | + | - | + | |
| - | + | - | + | BC |
50 µM zVAD-fmk [6h]
5 µM BV6 [6h]
M [kDa]
500 ng/ml CD95L [5h]
| - | + | - | + | - |
| --- | --- | --- | --- | --- |
| - | + | - | + | - |
| - | + | - | + | + |
50 µM zVAD-fmk [6h]
M [kDa]
500 ng/ml CD95L [5h]
78
RIPK1
78
pRIPK1
*
pMLKL
54
78
RIPK1
78
pRIPK1
54
54
MLKL
pMLKL
42
actin
FADD
27
*
54
procaspase-8 a/b s.e.
55/53
MLKL
27
FADD
p43/41
47/43
55/53
procaspase-8 a/b s.e.
procaspase-8 a/b l.e.
55/53
47/43
p43/41
p43/41
47/43
55/53
procaspase-8 a/b l.e.
47/43
p43/41
18
p18
c-FLIPL
55
p43
43
18
p18
55
c-FLIPL
43
p43
c-FLIPS
26
c-FLIPL
55
*
p43
43
c-FLIPS
26
42
actin
c-FLIPS
26

## Slide 2
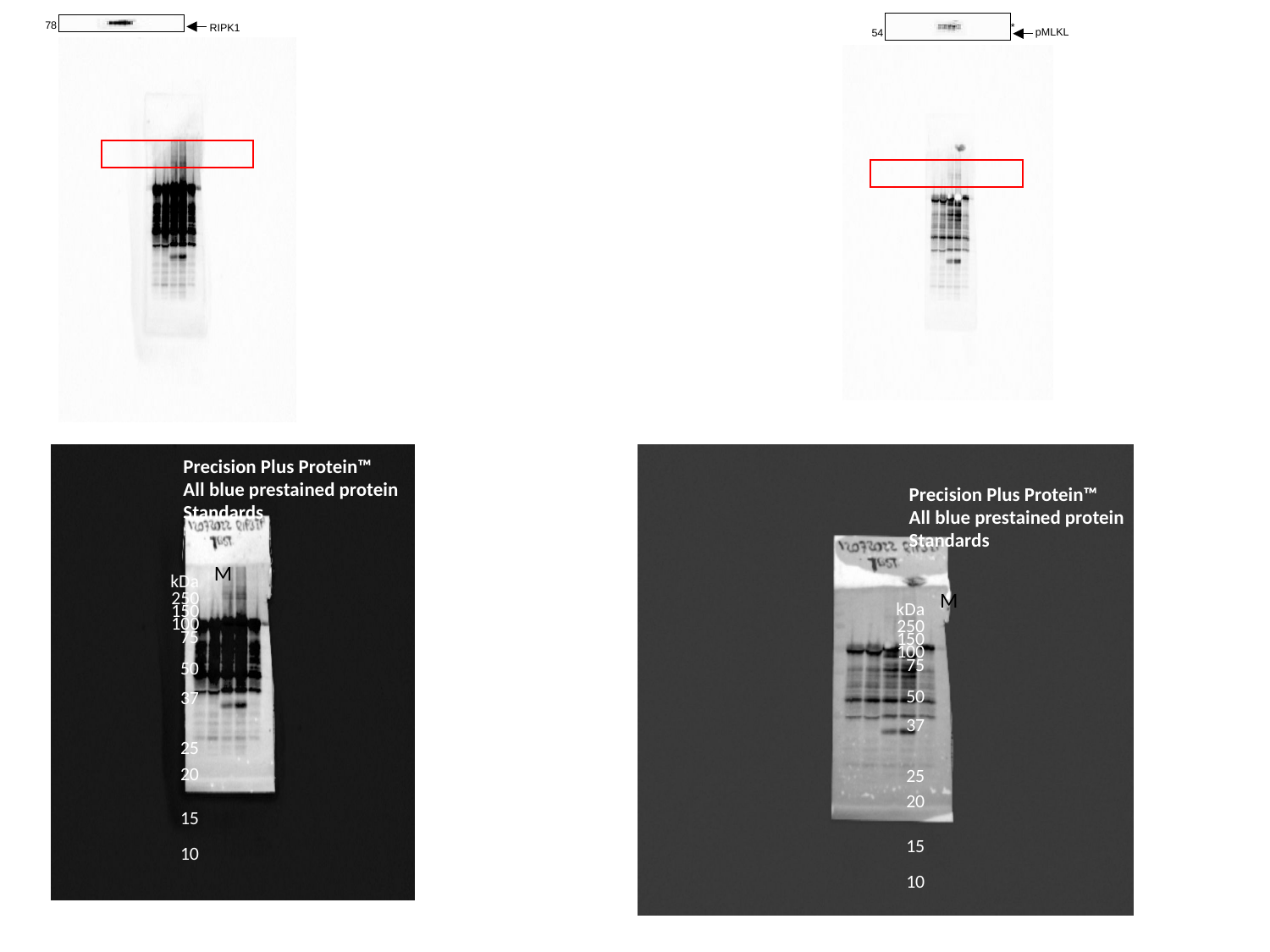

78
*
RIPK1
pMLKL
54
Precision Plus Protein™
All blue prestained protein
Standards
Precision Plus Protein™
All blue prestained protein
Standards
M
kDa
250
M
kDa
150
100
250
75
150
100
75
50
50
37
37
25
20
25
20
15
15
10
10

## Slide 3
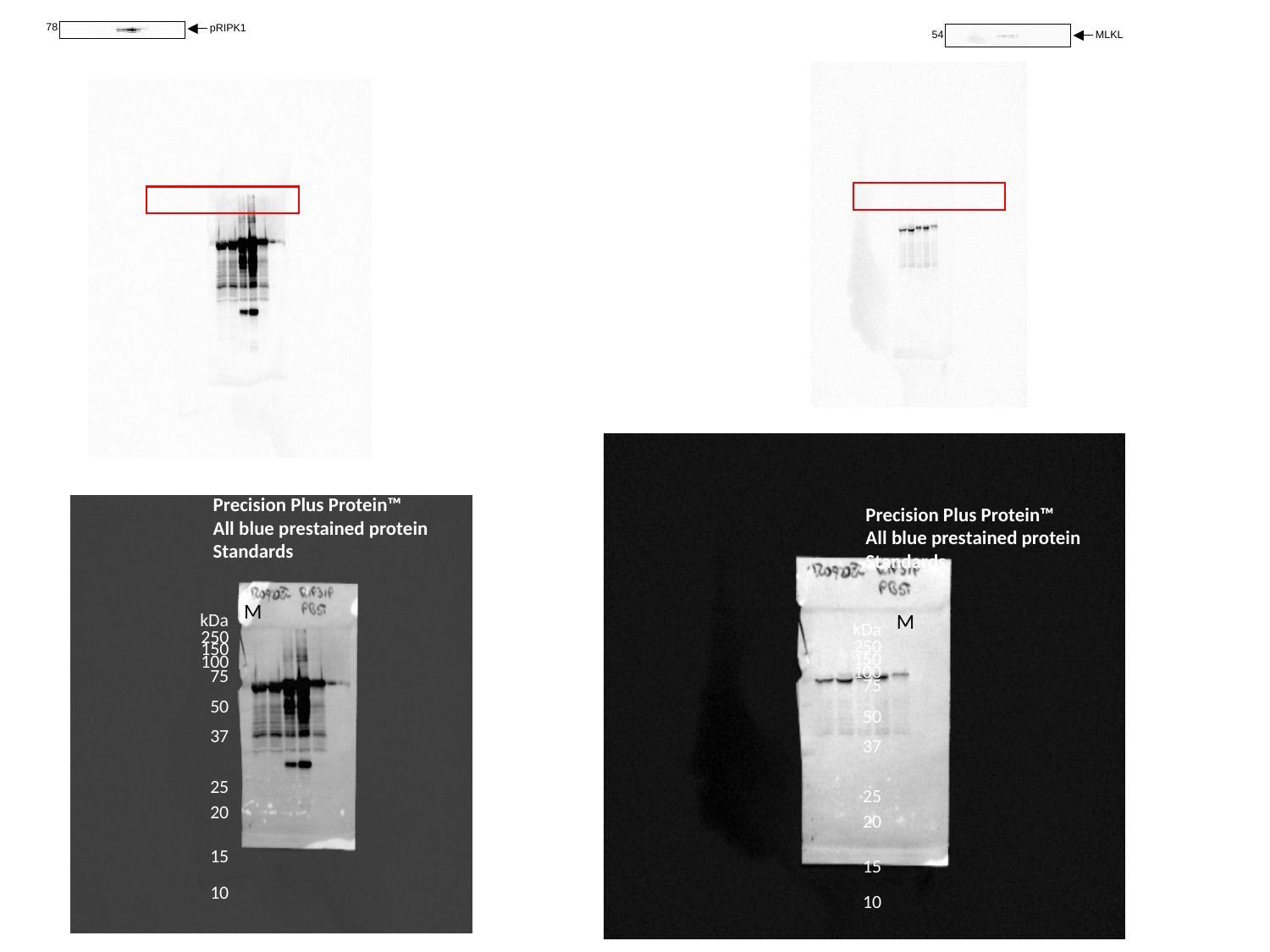

78
pRIPK1
54
MLKL
Precision Plus Protein™
All blue prestained protein
Standards
Precision Plus Protein™
All blue prestained protein
Standards
M
M
kDa
kDa
250
250
150
150
100
100
75
75
50
50
37
37
25
25
20
20
15
15
10
10

## Slide 4
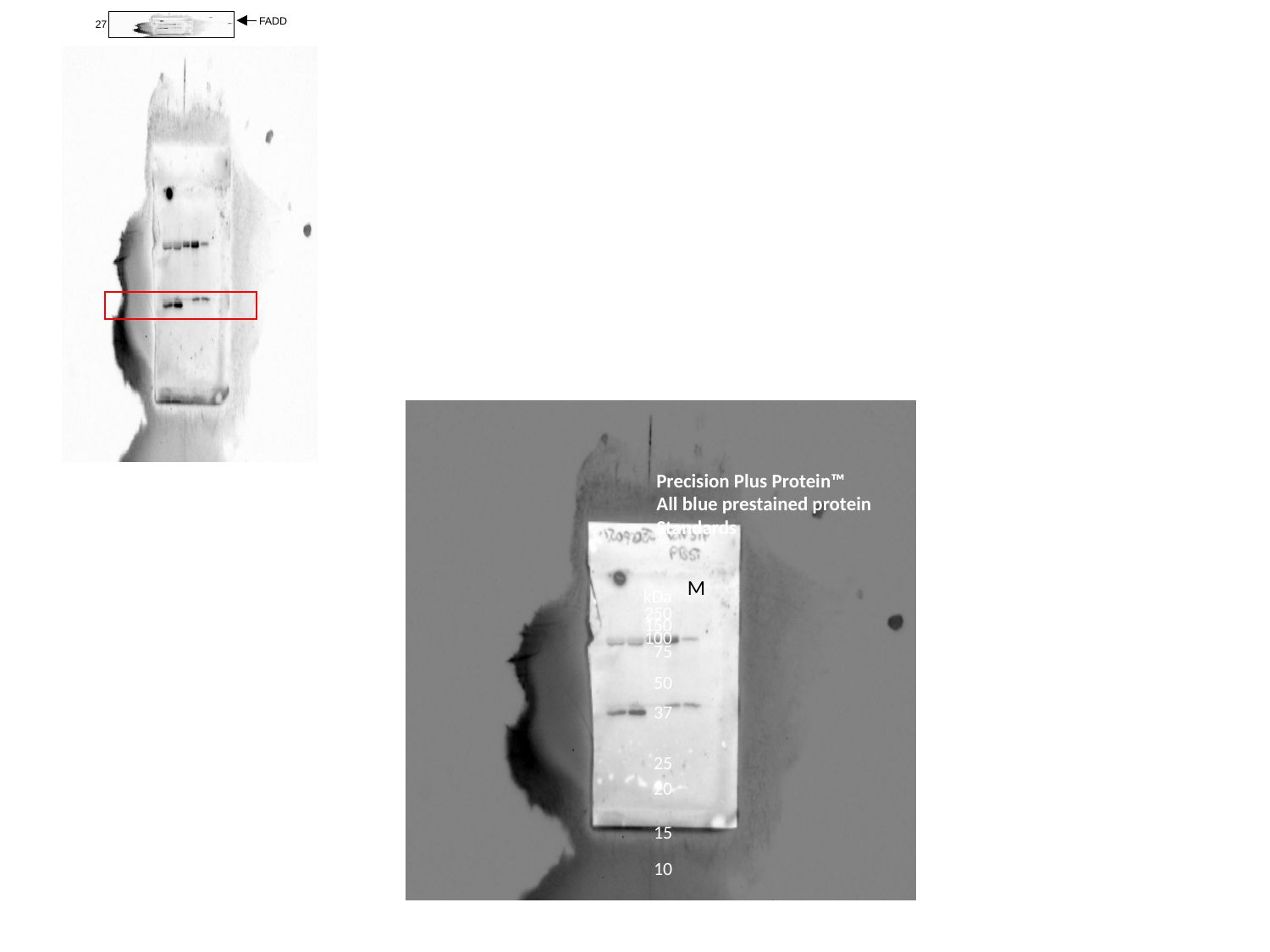

FADD
27
Precision Plus Protein™
All blue prestained protein
Standards
M
kDa
250
150
100
75
50
37
25
20
15
10

## Slide 5
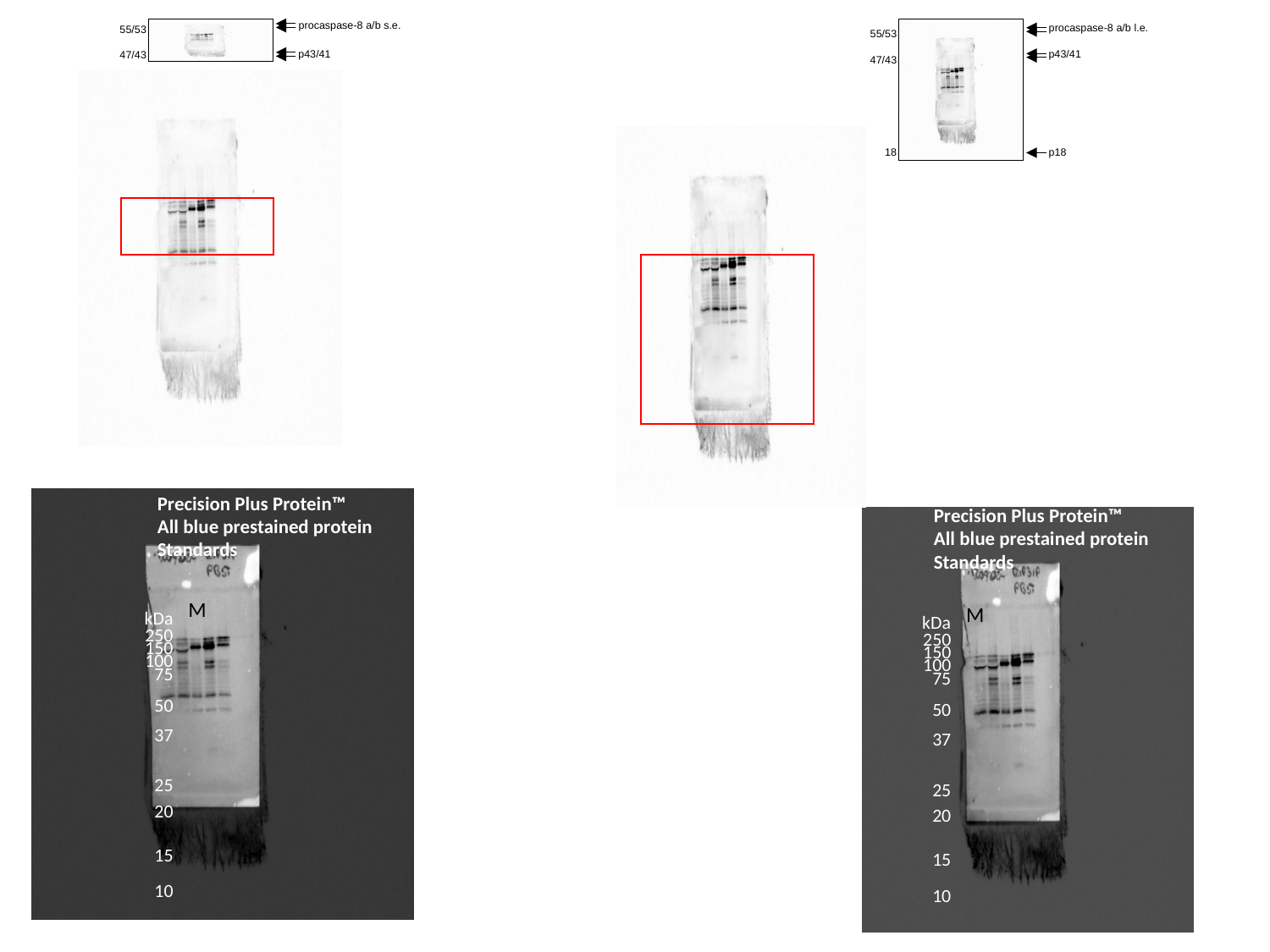

procaspase-8 a/b s.e.
procaspase-8 a/b l.e.
55/53
55/53
p43/41
p43/41
47/43
47/43
18
p18
Precision Plus Protein™
All blue prestained protein
Standards
Precision Plus Protein™
All blue prestained protein
Standards
M
M
kDa
kDa
250
250
150
150
100
100
75
75
50
50
37
37
25
25
20
20
15
15
10
10

## Slide 6
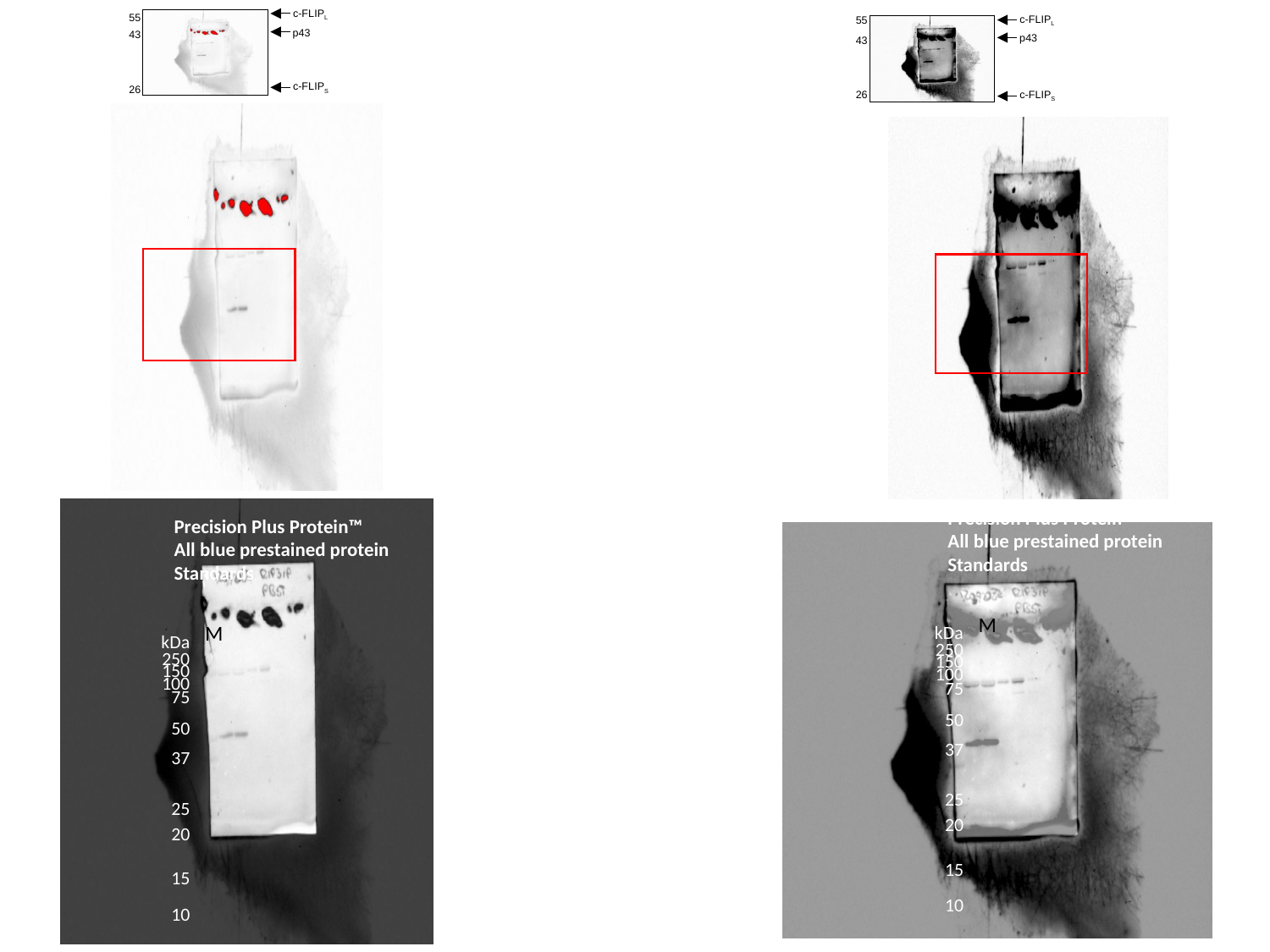

c-FLIPL
55
c-FLIPL
55
p43
43
p43
43
c-FLIPS
26
c-FLIPS
26
Precision Plus Protein™
All blue prestained protein
Standards
Precision Plus Protein™
All blue prestained protein
Standards
M
M
kDa
kDa
250
250
150
150
100
100
75
75
50
50
37
37
25
25
20
20
15
15
10
10

## Slide 7
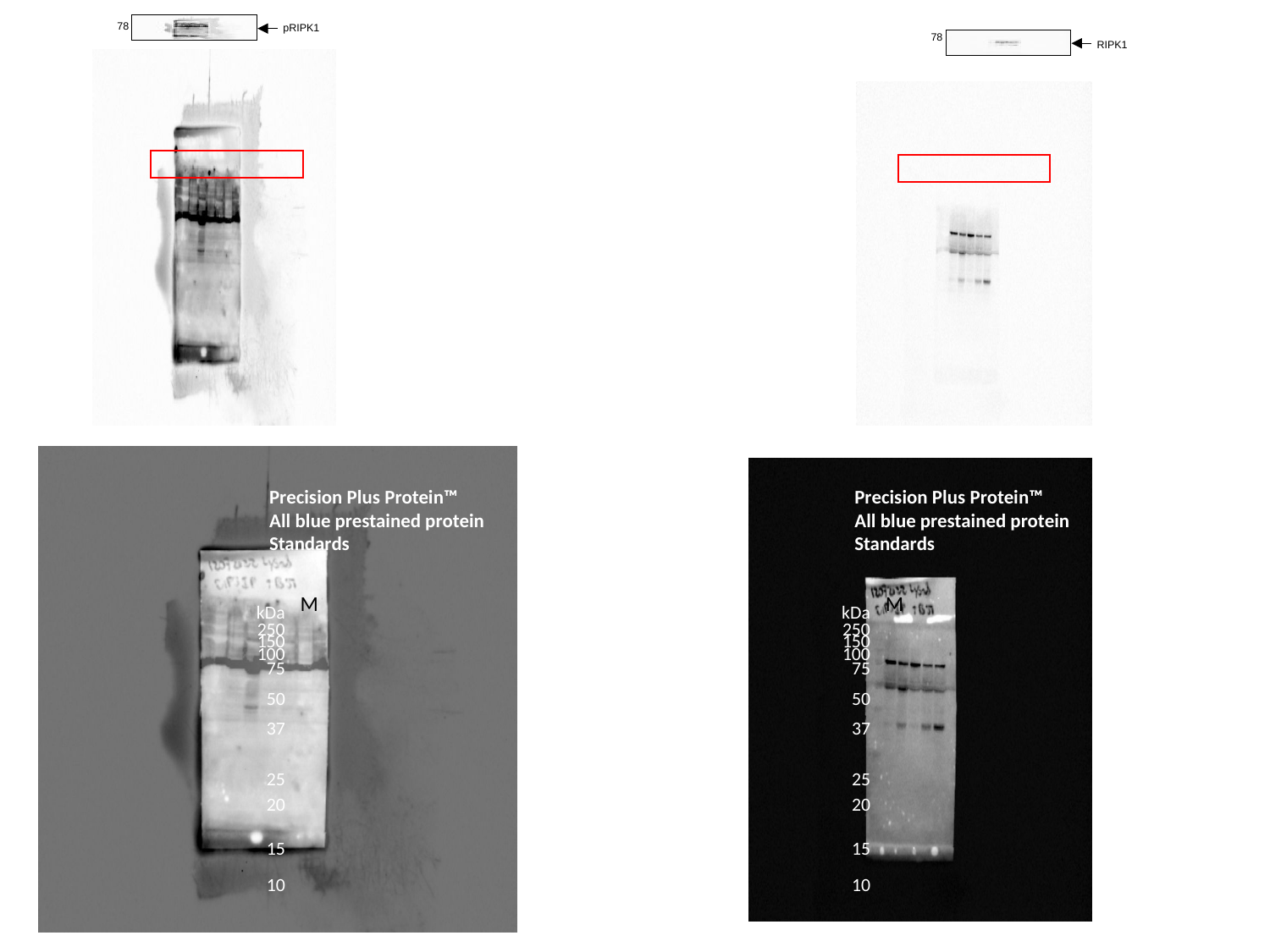

78
pRIPK1
78
RIPK1
Precision Plus Protein™
All blue prestained protein
Standards
Precision Plus Protein™
All blue prestained protein
Standards
M
M
kDa
kDa
250
250
150
150
100
100
75
75
50
50
37
37
25
25
20
20
15
15
10
10

## Slide 8
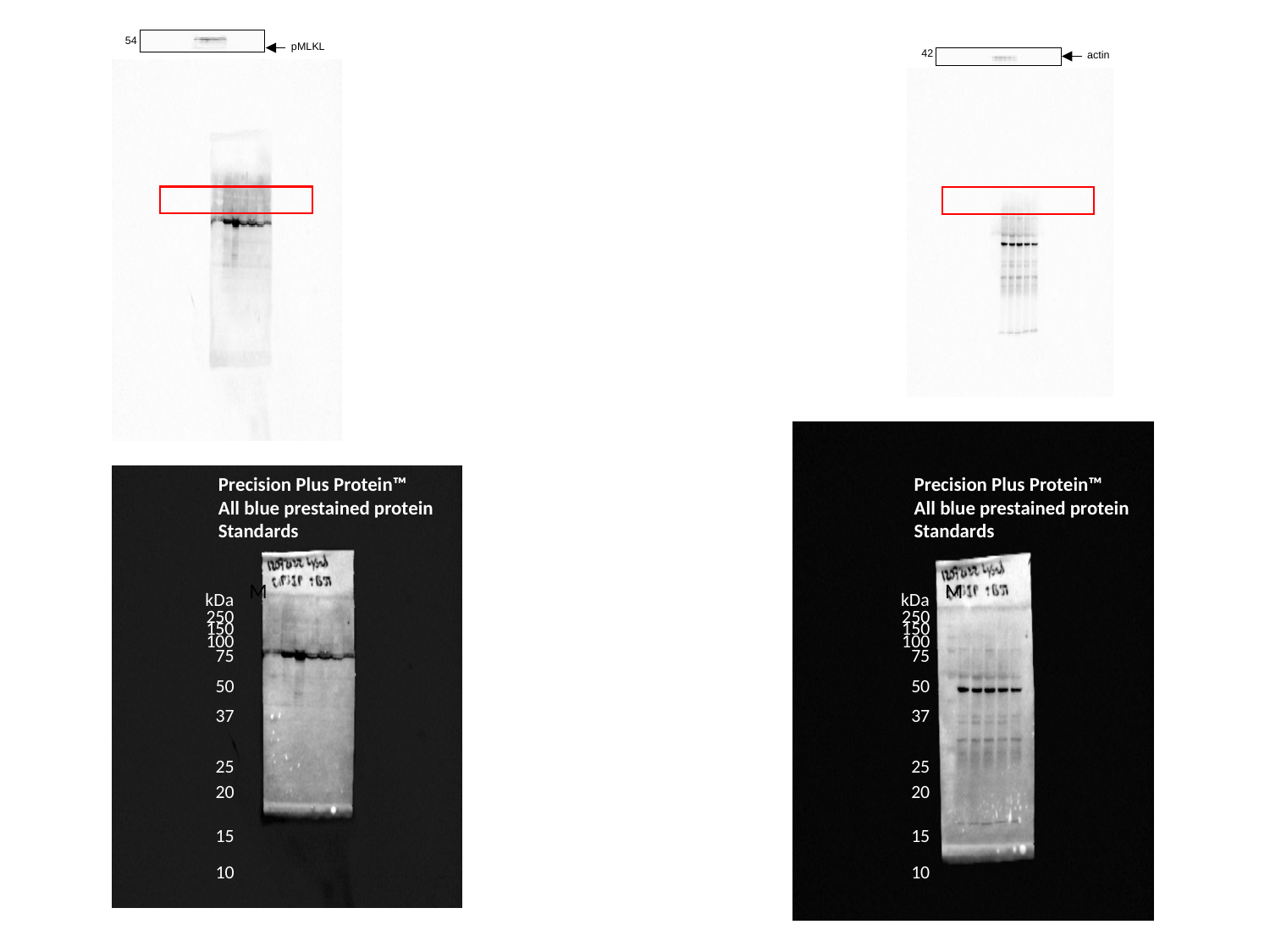

54
pMLKL
42
actin
Precision Plus Protein™
All blue prestained protein
Standards
Precision Plus Protein™
All blue prestained protein
Standards
M
M
kDa
kDa
250
250
150
150
100
100
75
75
50
50
37
37
25
25
20
20
15
15
10
10

## Slide 9
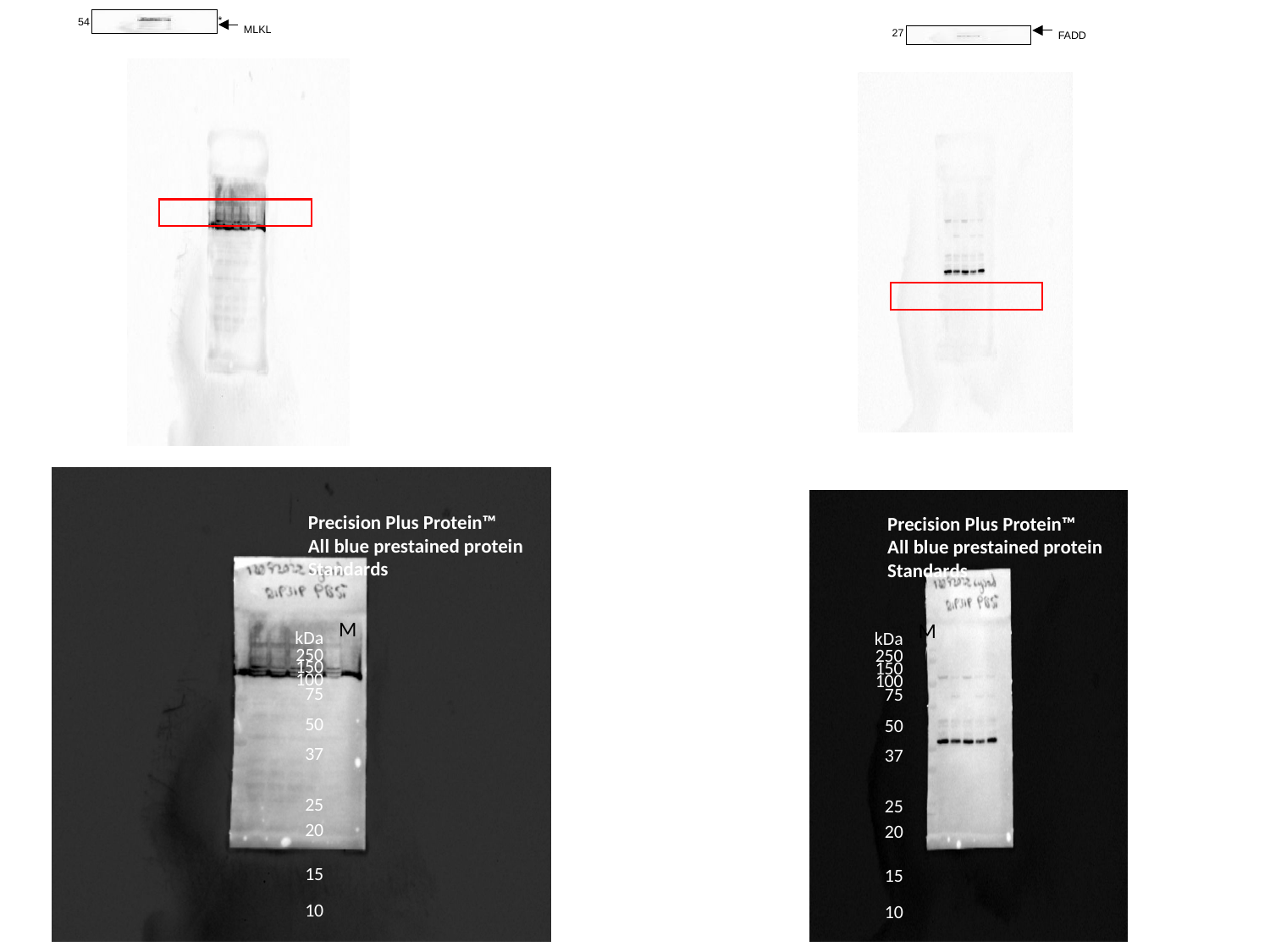

*
54
MLKL
27
FADD
Precision Plus Protein™
All blue prestained protein
Standards
Precision Plus Protein™
All blue prestained protein
Standards
M
M
kDa
kDa
250
250
150
150
100
100
75
75
50
50
37
37
25
25
20
20
15
15
10
10

## Slide 10
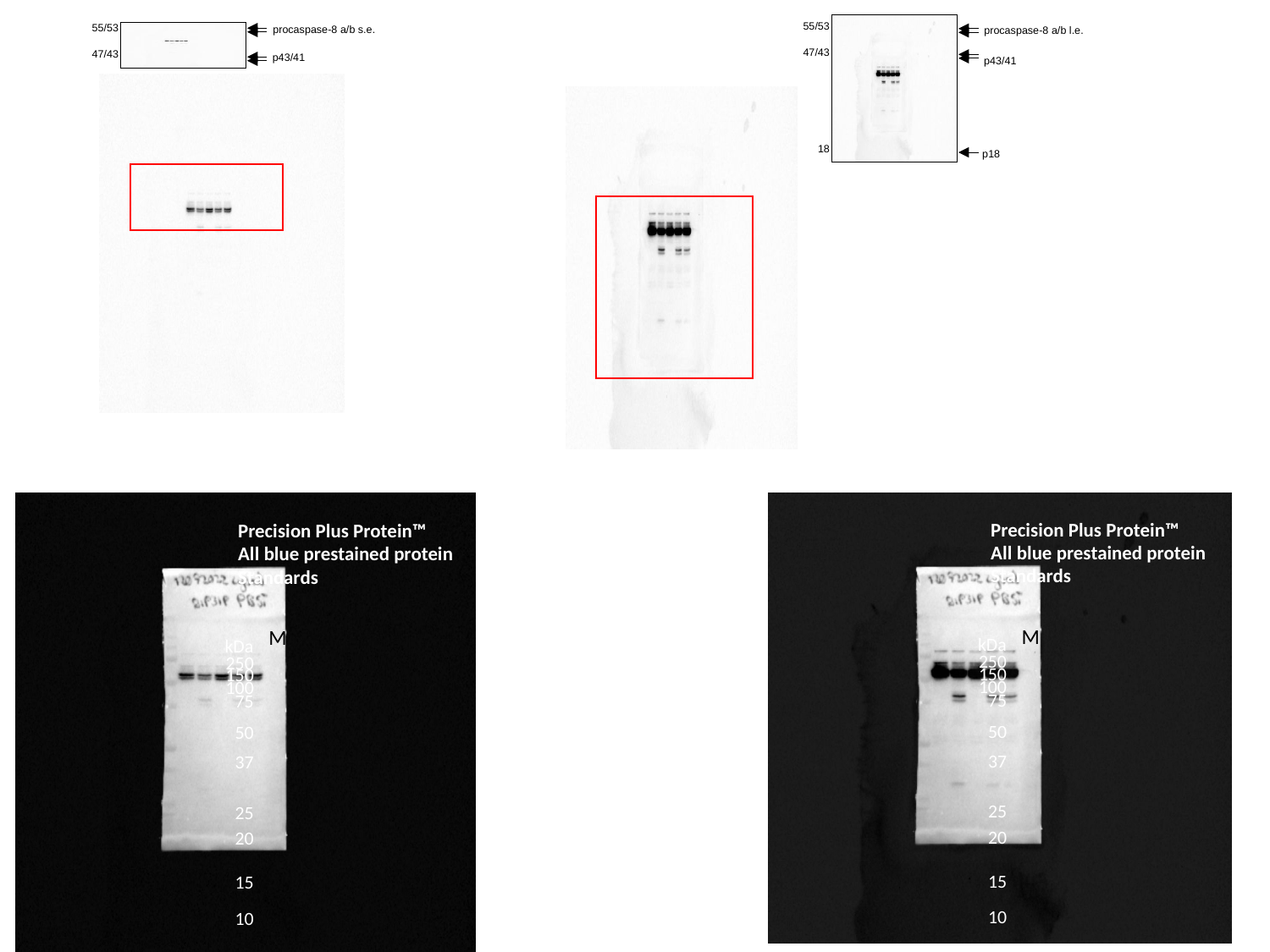

55/53
55/53
procaspase-8 a/b s.e.
procaspase-8 a/b l.e.
47/43
47/43
p43/41
p43/41
18
p18
Precision Plus Protein™
All blue prestained protein
Standards
Precision Plus Protein™
All blue prestained protein
Standards
M
M
kDa
kDa
250
250
150
150
100
100
75
75
50
50
37
37
25
25
20
20
15
15
10
10

## Slide 11
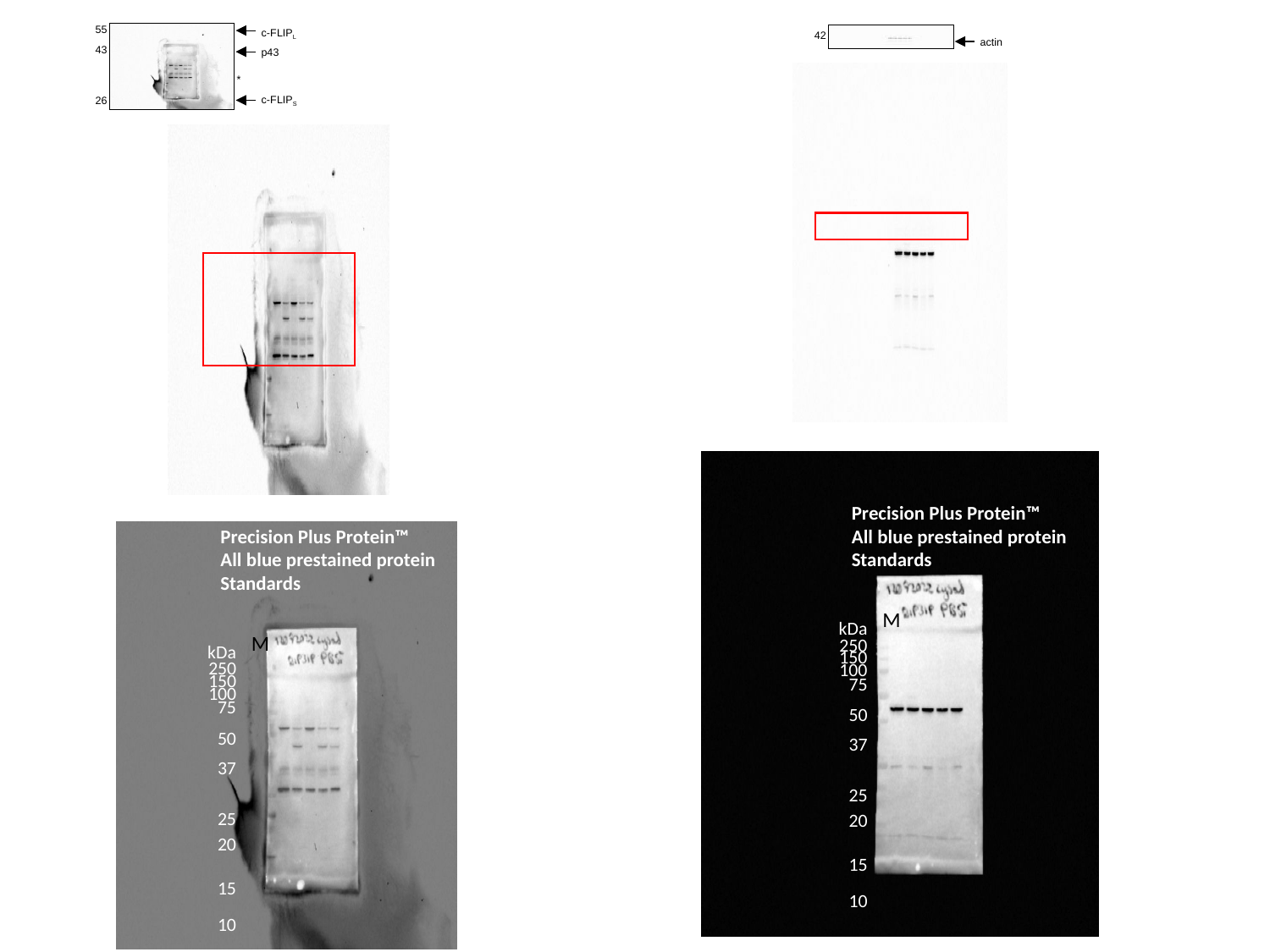

55
c-FLIPL
42
actin
43
p43
*
c-FLIPS
26
Precision Plus Protein™
All blue prestained protein
Standards
Precision Plus Protein™
All blue prestained protein
Standards
M
kDa
M
250
kDa
150
250
100
150
75
100
75
50
50
37
37
25
25
20
20
15
15
10
10
