# Supplementary material for: Oligomerised RIPK1 is the main core component of the CD95 necrosome
Source: EMBO J. 2025 Apr 16;44(11):3231–65. doi: 10.1038/s44318-025-00433-0 (PMC12130296; doi:10.1038/s44318-025-00433-0)
Supplement: Supplementary file 14 — Appendix Source Data [file 44318_2025_433_MOESM14_ESM.zip › S1C.pptx]

## Slide 1
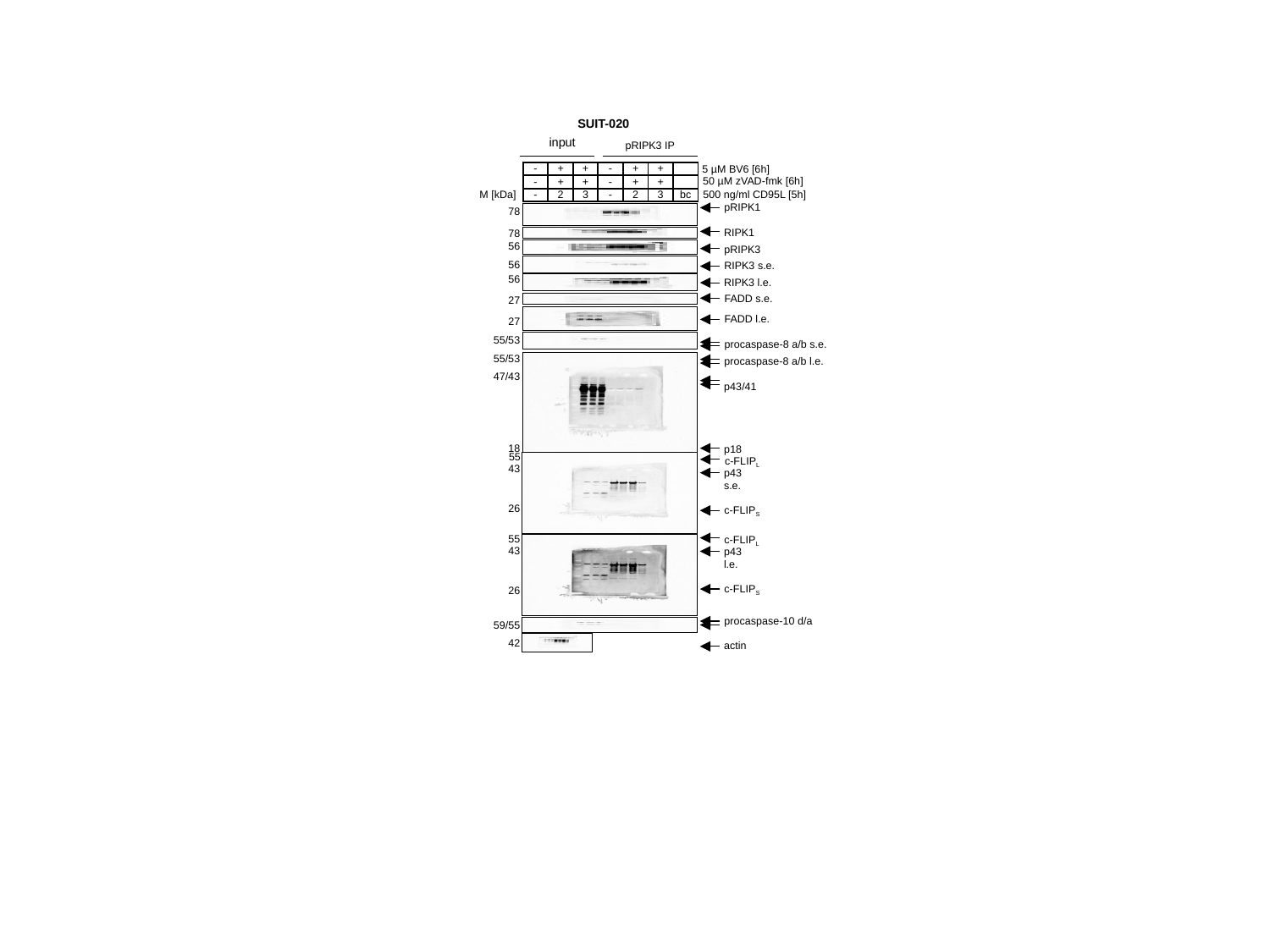

SUIT-020
input
pRIPK3 IP
5 µM BV6 [6h]
| - | + | + | - | + | + | |
| --- | --- | --- | --- | --- | --- | --- |
| - | + | + | - | + | + | |
| - | 2 | 3 | - | 2 | 3 | bc |
50 µM zVAD-fmk [6h]
M [kDa]
500 ng/ml CD95L [5h]
pRIPK1
78
RIPK1
78
56
pRIPK3
56
RIPK3 s.e.
56
RIPK3 l.e.
FADD s.e.
27
FADD l.e.
27
55/53
procaspase-8 a/b s.e.
55/53
procaspase-8 a/b l.e.
47/43
p43/41
18
p18
55
c-FLIPL
43
p43
s.e.
26
c-FLIPS
55
c-FLIPL
43
p43
l.e.
c-FLIPS
26
procaspase-10 d/a
59/55
42
actin

## Slide 2
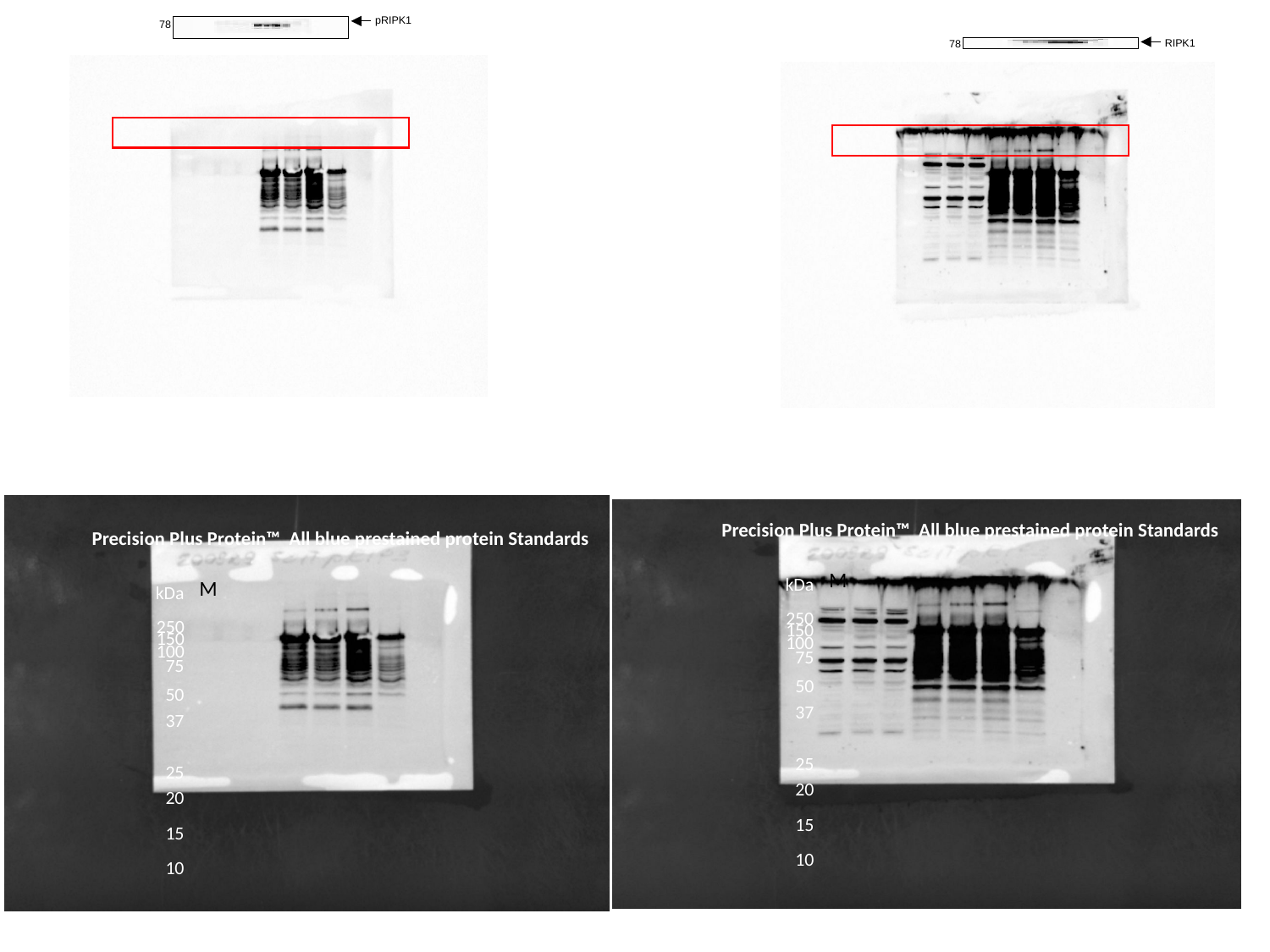

pRIPK1
78
RIPK1
78
Precision Plus Protein™ All blue prestained protein Standards
Precision Plus Protein™ All blue prestained protein Standards
M
kDa
M
kDa
250
250
150
150
100
100
75
75
50
50
37
37
25
25
20
20
15
15
10
10

## Slide 3
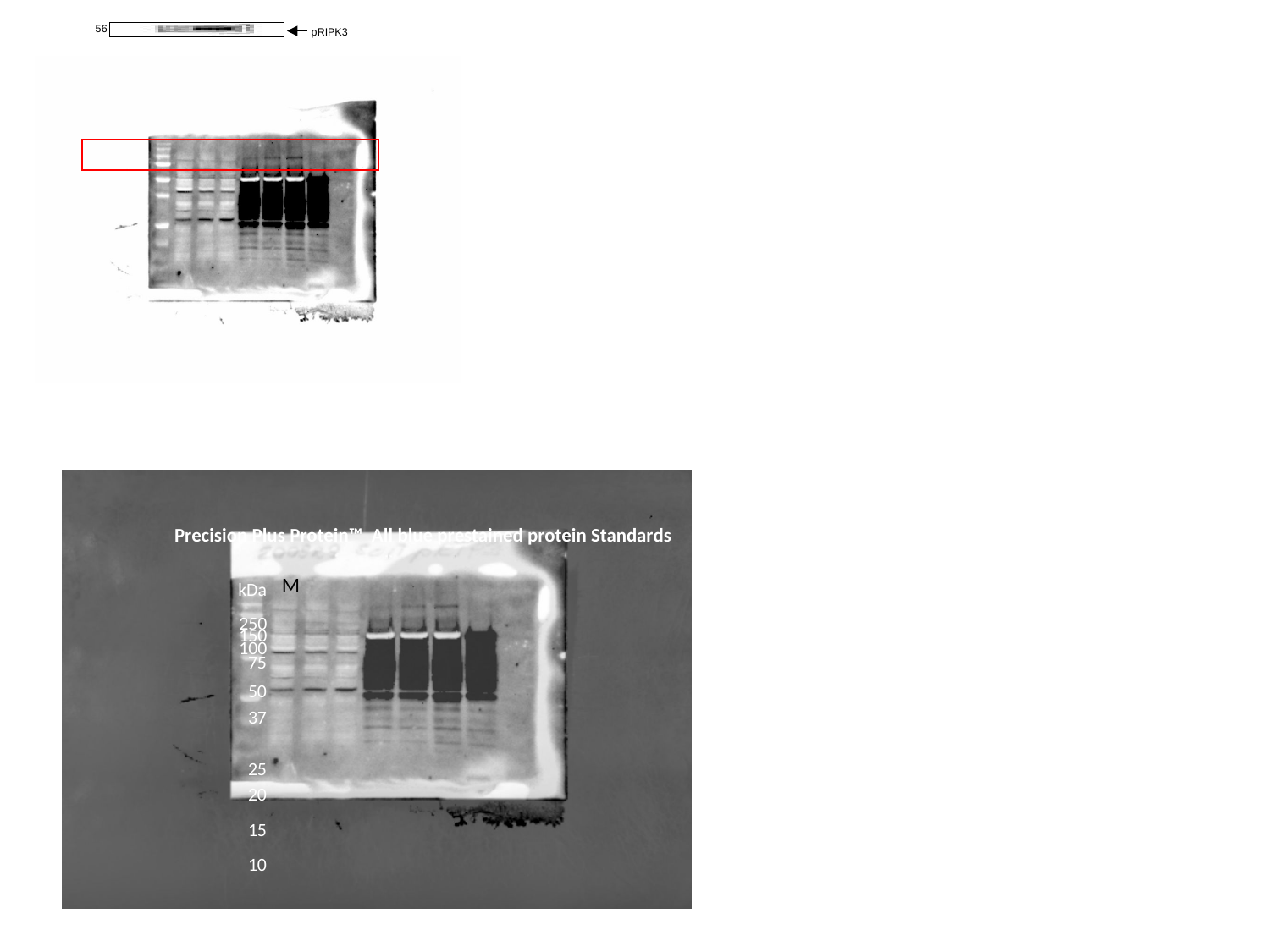

56
pRIPK3
Precision Plus Protein™ All blue prestained protein Standards
M
kDa
250
150
100
75
50
37
25
20
15
10

## Slide 4
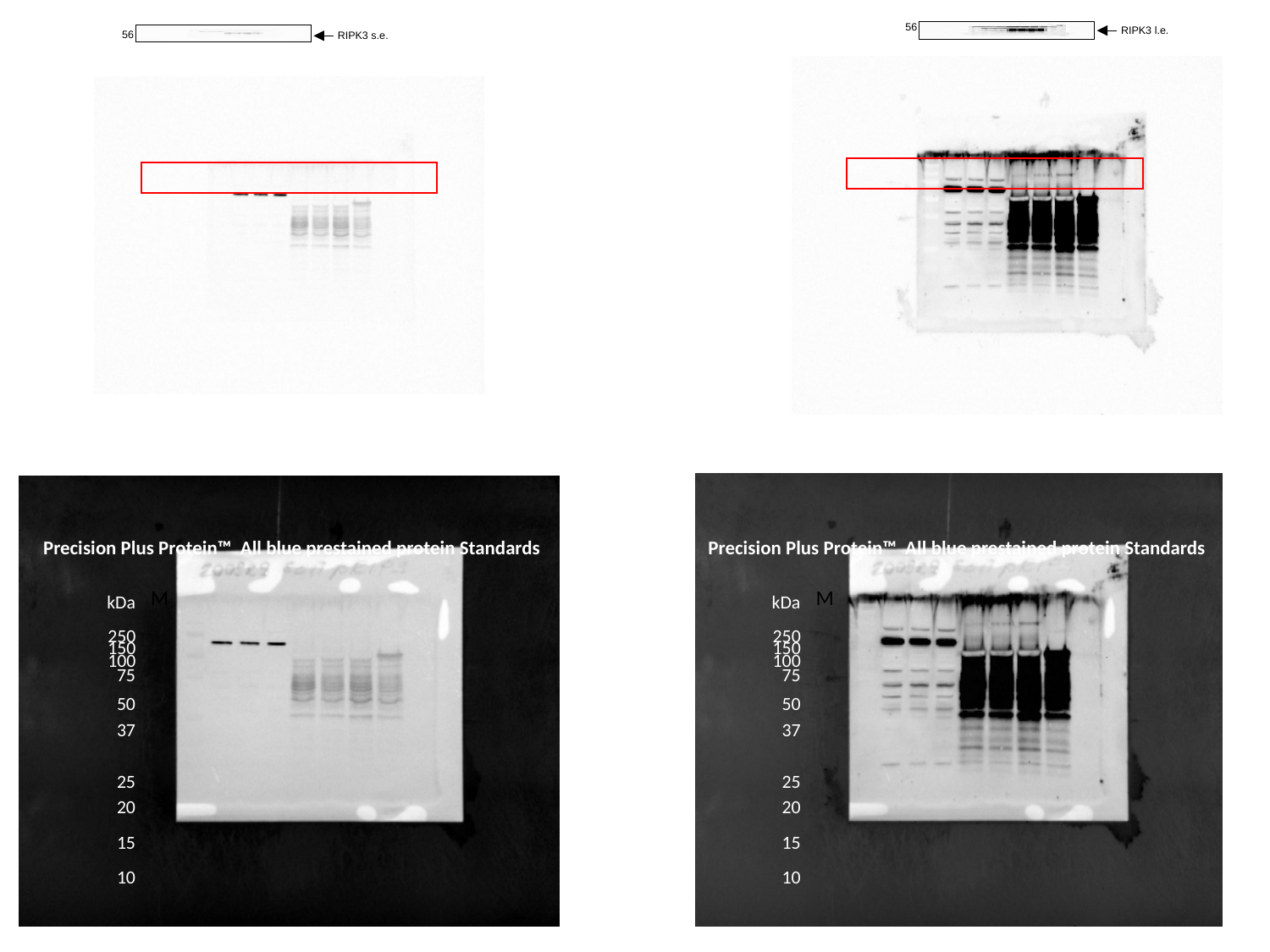

56
RIPK3 l.e.
56
RIPK3 s.e.
Precision Plus Protein™ All blue prestained protein Standards
Precision Plus Protein™ All blue prestained protein Standards
M
M
kDa
kDa
250
250
150
150
100
100
75
75
50
50
37
37
25
25
20
20
15
15
10
10

## Slide 5
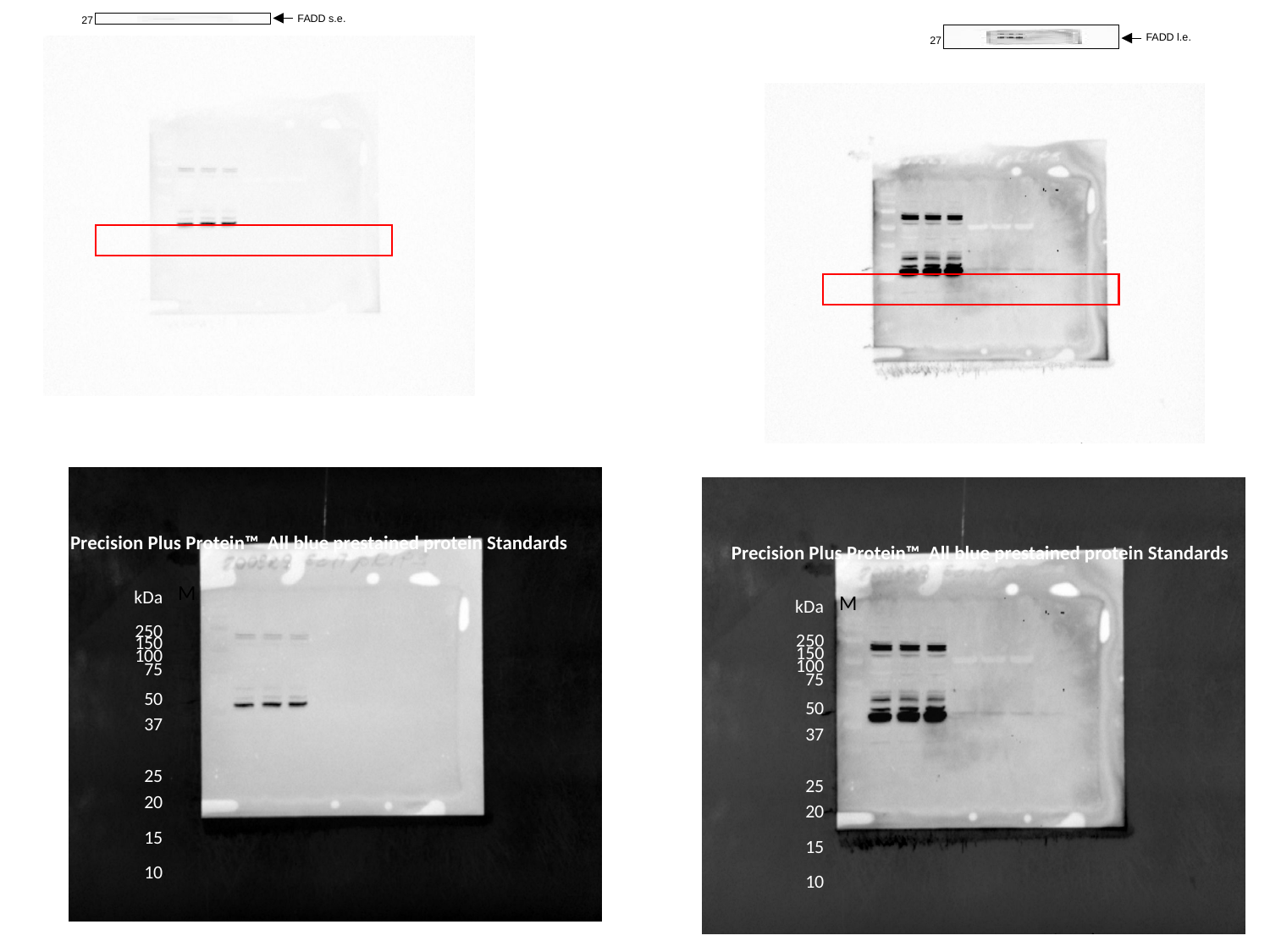

FADD s.e.
27
FADD l.e.
27
Precision Plus Protein™ All blue prestained protein Standards
Precision Plus Protein™ All blue prestained protein Standards
M
kDa
M
kDa
250
250
150
150
100
100
75
75
50
50
37
37
25
25
20
20
15
15
10
10

## Slide 6
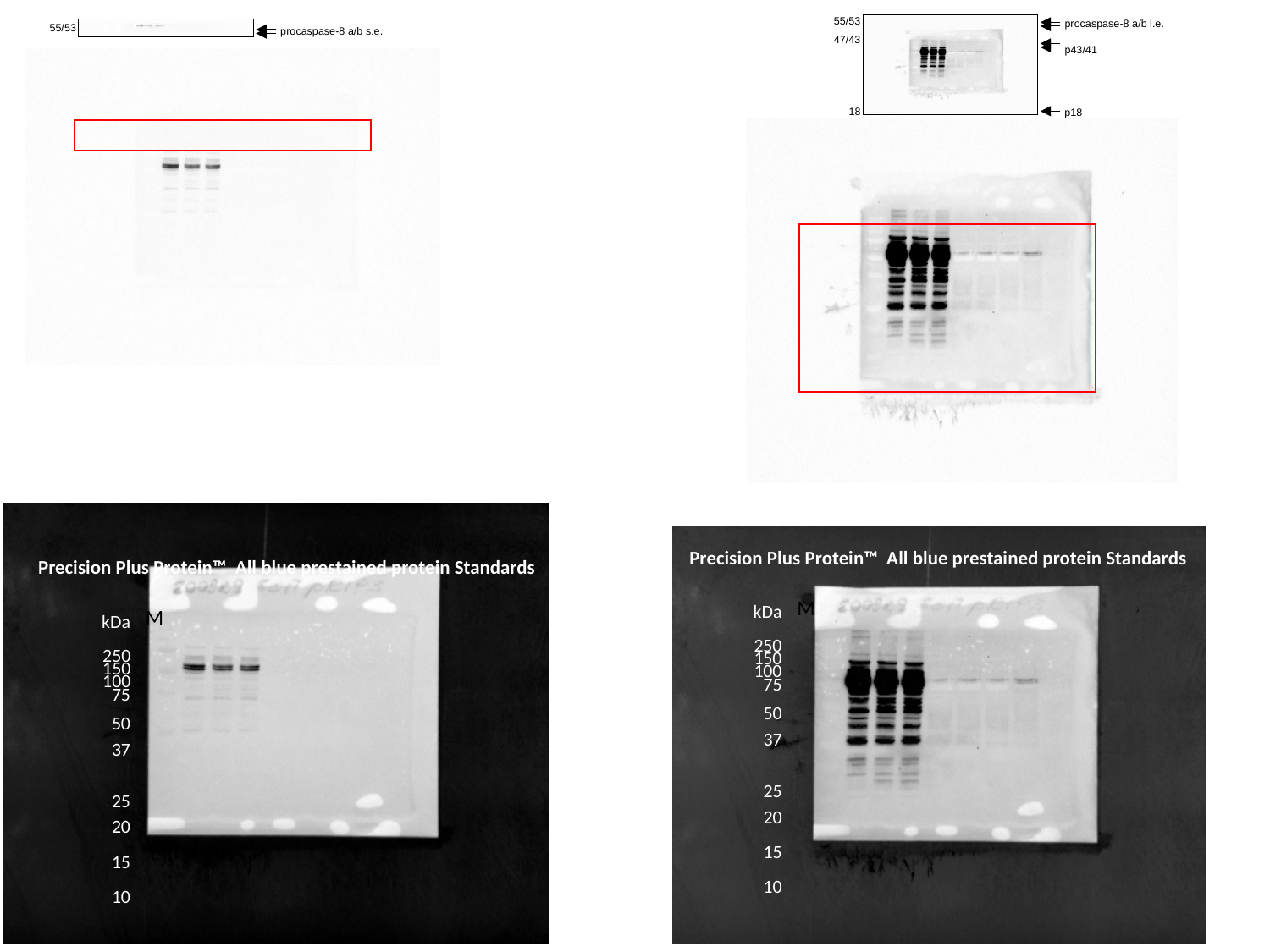

55/53
procaspase-8 a/b l.e.
55/53
procaspase-8 a/b s.e.
47/43
p43/41
18
p18
Precision Plus Protein™ All blue prestained protein Standards
Precision Plus Protein™ All blue prestained protein Standards
M
kDa
M
kDa
250
250
150
150
100
100
75
75
50
50
37
37
25
25
20
20
15
15
10
10

## Slide 7
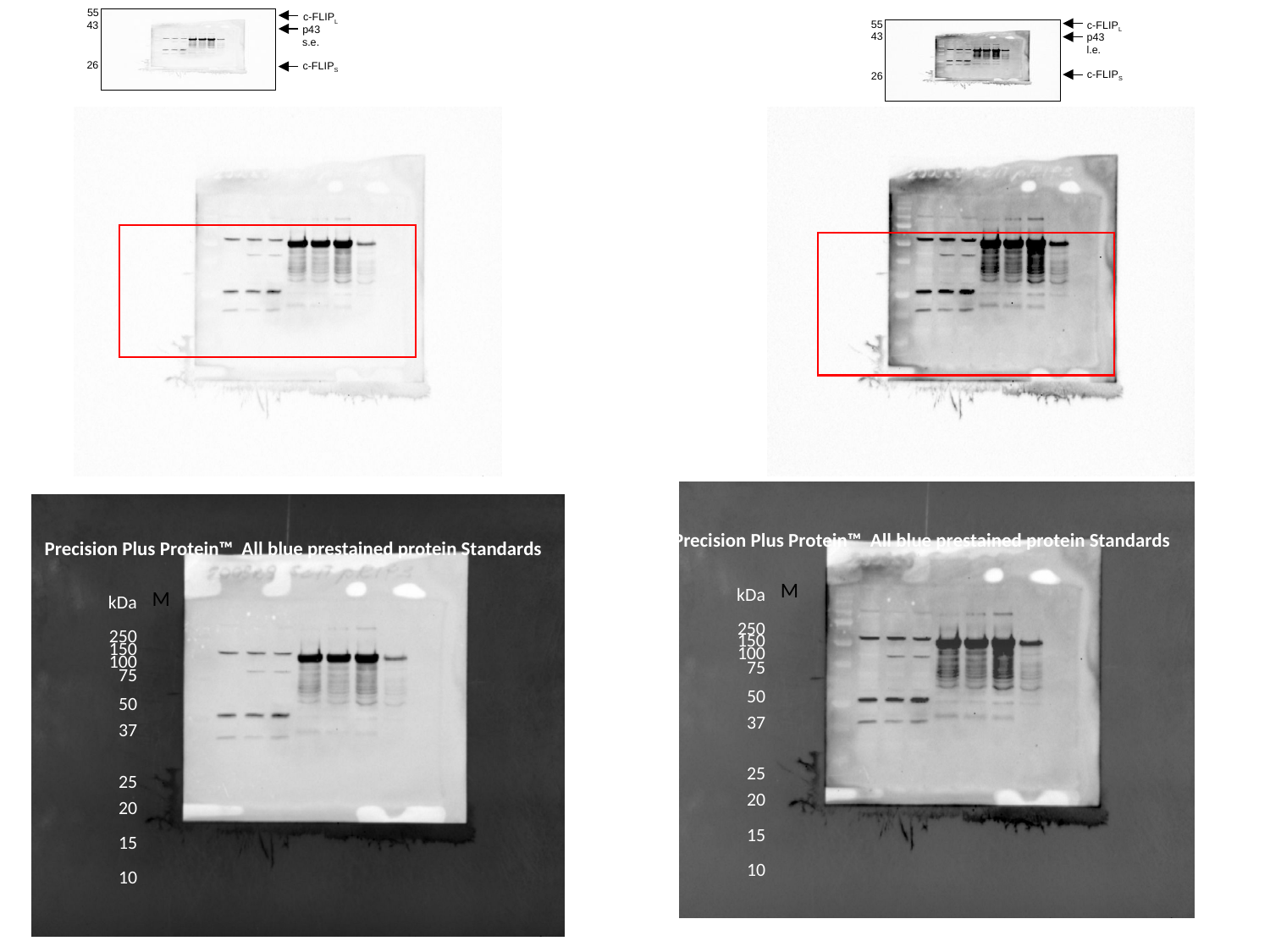

55
c-FLIPL
55
43
c-FLIPL
p43
43
p43
s.e.
l.e.
26
c-FLIPS
c-FLIPS
26
Precision Plus Protein™ All blue prestained protein Standards
Precision Plus Protein™ All blue prestained protein Standards
M
kDa
M
kDa
250
250
150
150
100
100
75
75
50
50
37
37
25
25
20
20
15
15
10
10

## Slide 8
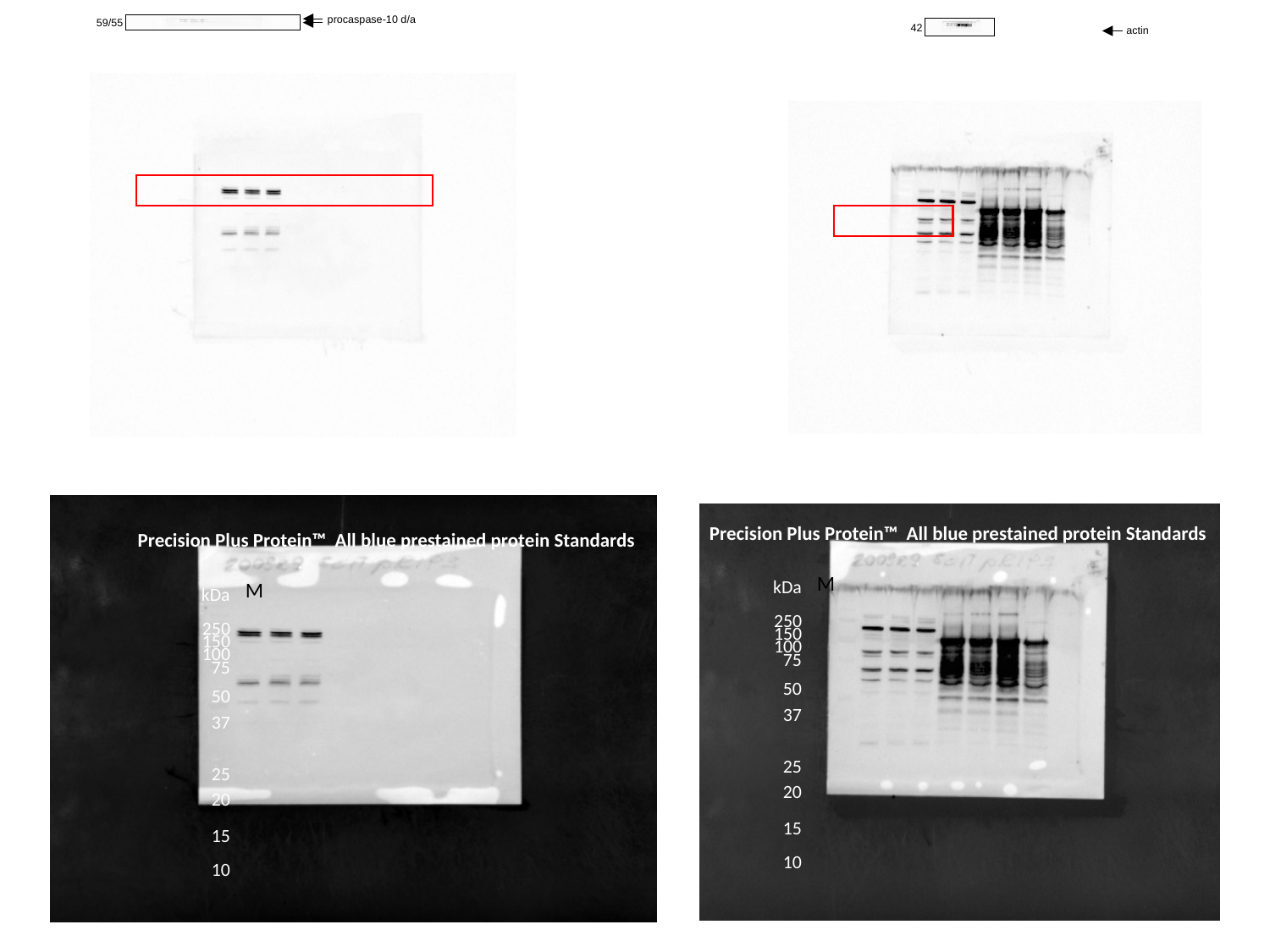

procaspase-10 d/a
59/55
42
actin
Precision Plus Protein™ All blue prestained protein Standards
Precision Plus Protein™ All blue prestained protein Standards
M
kDa
M
kDa
250
250
150
150
100
100
75
75
50
50
37
37
25
25
20
20
15
15
10
10
