# Supplementary material for: Oligomerised RIPK1 is the main core component of the CD95 necrosome
Source: EMBO J. 2025 Apr 16;44(11):3231–65. doi: 10.1038/s44318-025-00433-0 (PMC12130296; doi:10.1038/s44318-025-00433-0)
Supplement: Supplementary file 14 — Appendix Source Data [file 44318_2025_433_MOESM14_ESM.zip › S1D.pptx]

## Slide 1
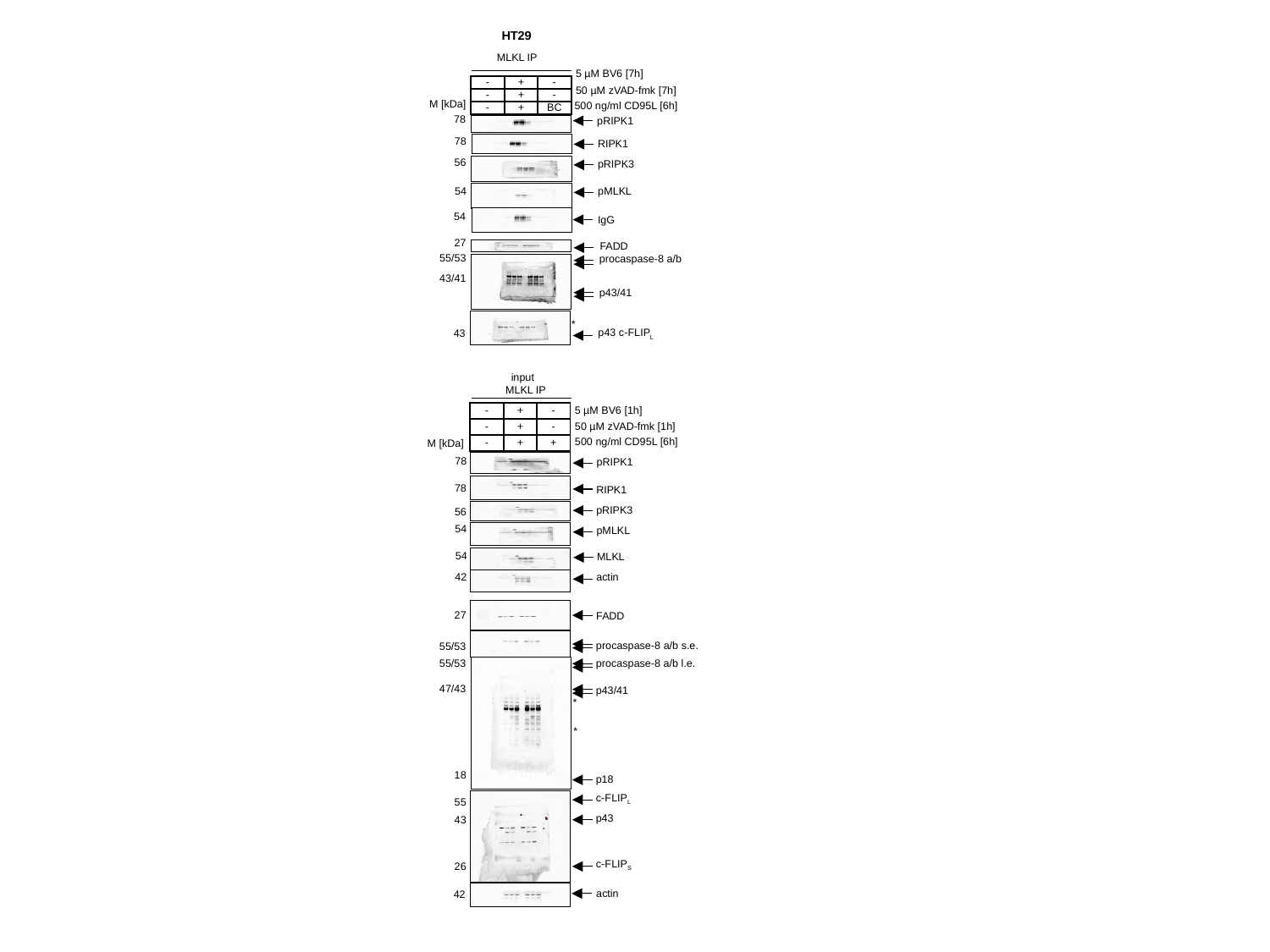

HT29
MLKL IP
5 µM BV6 [7h]
| - | + | - |
| --- | --- | --- |
| - | + | - |
| - | + | BC |
50 µM zVAD-fmk [7h]
M [kDa]
500 ng/ml CD95L [6h]
78
pRIPK1
78
RIPK1
56
pRIPK3
54
pMLKL
54
IgG
27
FADD
55/53
procaspase-8 a/b
43/41
p43/41
*
p43 c-FLIPL
43
input
MLKL IP
5 µM BV6 [1h]
| - | + | - |
| --- | --- | --- |
| - | + | - |
| - | + | + |
50 µM zVAD-fmk [1h]
500 ng/ml CD95L [6h]
M [kDa]
78
pRIPK1
78
RIPK1
pRIPK3
56
54
pMLKL
54
MLKL
42
actin
27
FADD
procaspase-8 a/b s.e.
55/53
55/53
procaspase-8 a/b l.e.
47/43
p43/41
*
*
18
p18
c-FLIPL
55
p43
43
c-FLIPS
26
actin
42

## Slide 2
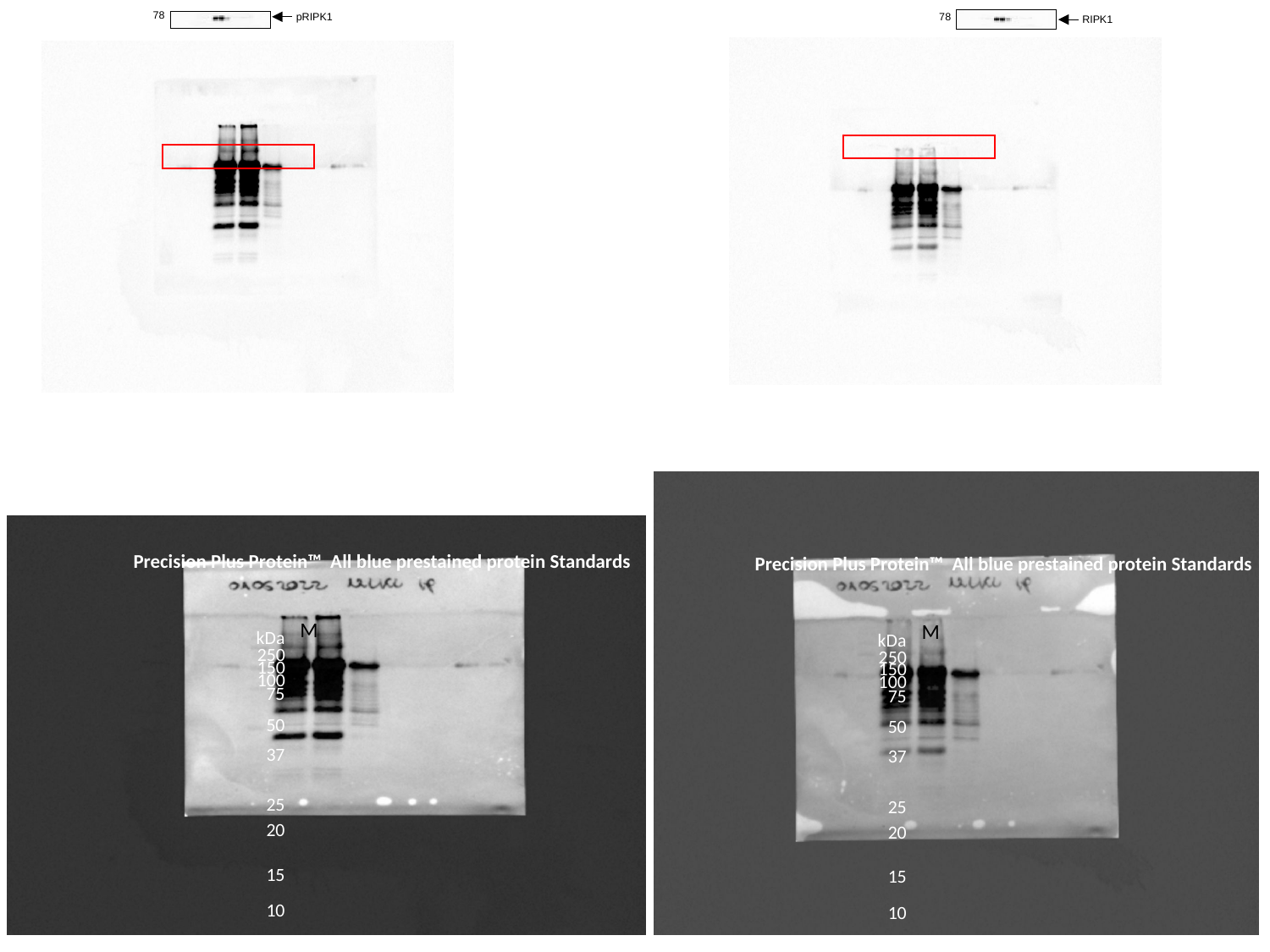

78
pRIPK1
78
RIPK1
Precision Plus Protein™ All blue prestained protein Standards
Precision Plus Protein™ All blue prestained protein Standards
M
M
kDa
kDa
250
250
150
150
100
100
75
75
50
50
37
37
25
25
20
20
15
15
10
10

## Slide 3
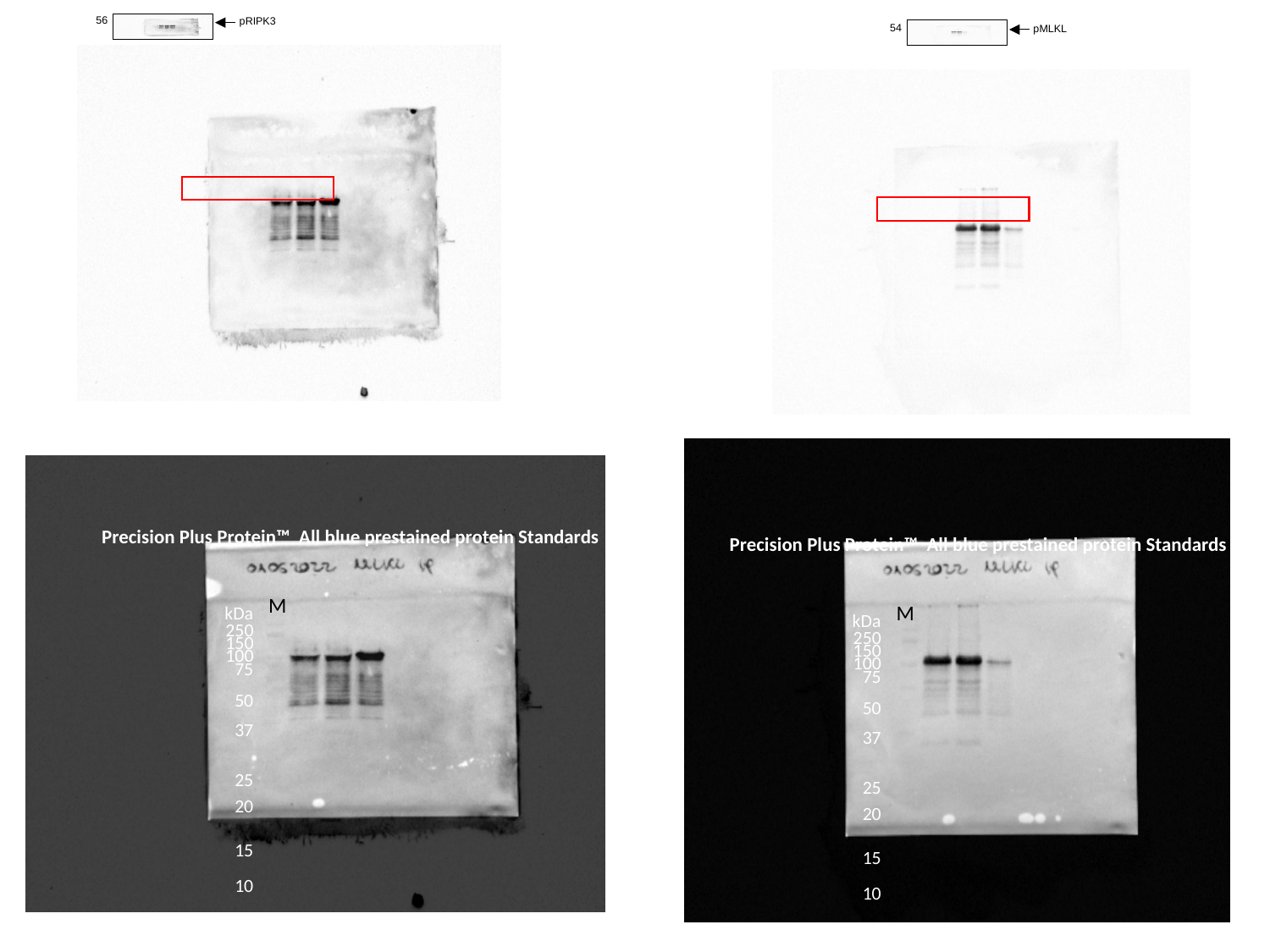

56
pRIPK3
54
pMLKL
Precision Plus Protein™ All blue prestained protein Standards
Precision Plus Protein™ All blue prestained protein Standards
M
M
kDa
kDa
250
250
150
150
100
100
75
75
50
50
37
37
25
25
20
20
15
15
10
10

## Slide 4
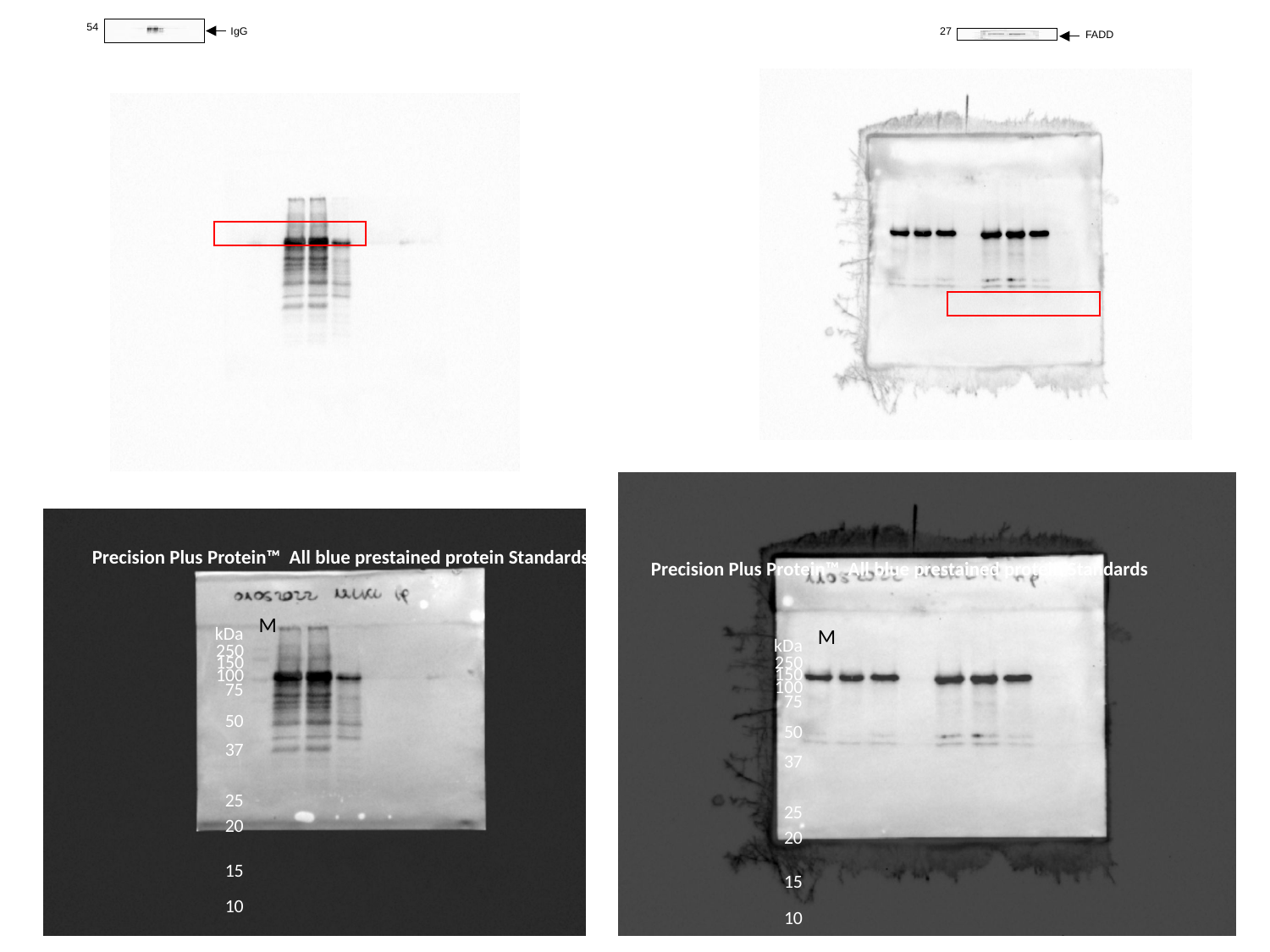

54
IgG
27
FADD
Precision Plus Protein™ All blue prestained protein Standards
Precision Plus Protein™ All blue prestained protein Standards
M
kDa
M
kDa
250
250
150
150
100
100
75
75
50
50
37
37
25
25
20
20
15
15
10
10

## Slide 5
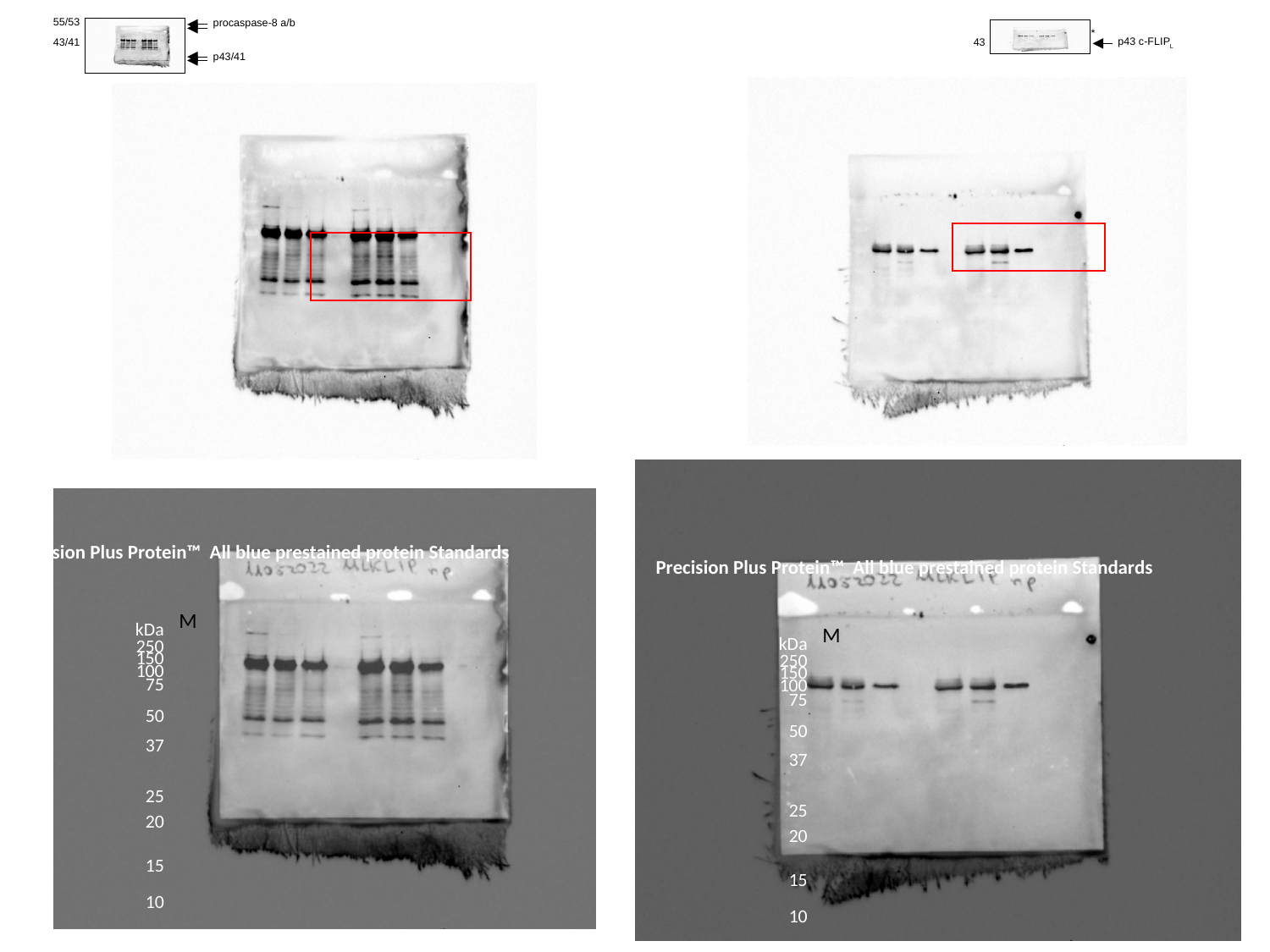

55/53
procaspase-8 a/b
*
p43 c-FLIPL
43
43/41
p43/41
Precision Plus Protein™ All blue prestained protein Standards
Precision Plus Protein™ All blue prestained protein Standards
M
kDa
M
kDa
250
150
250
100
150
75
100
75
50
50
37
37
25
25
20
20
15
15
10
10

## Slide 6
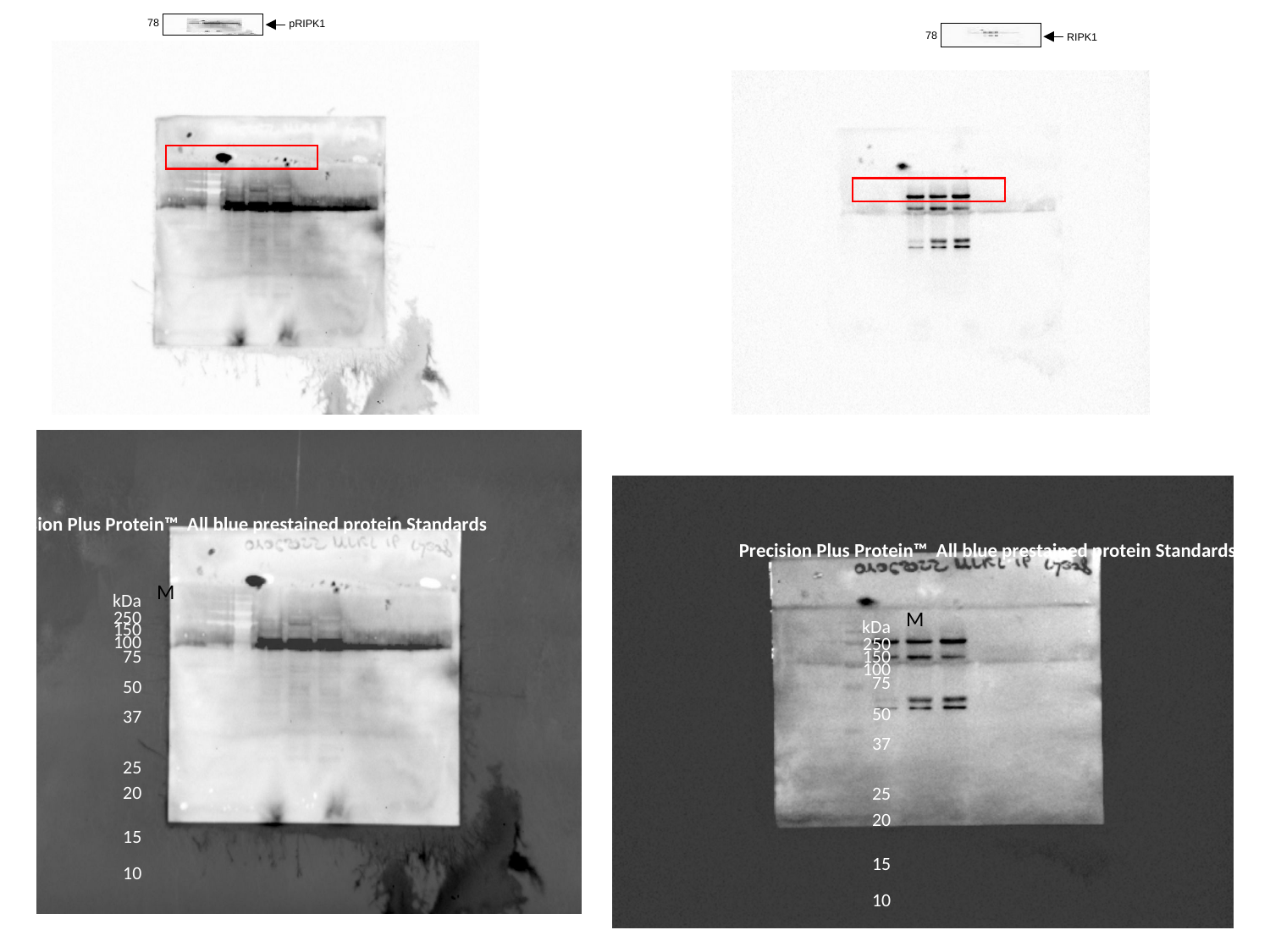

78
pRIPK1
78
RIPK1
Precision Plus Protein™ All blue prestained protein Standards
Precision Plus Protein™ All blue prestained protein Standards
M
kDa
M
250
kDa
150
100
250
150
75
100
75
50
50
37
37
25
20
25
20
15
15
10
10

## Slide 7
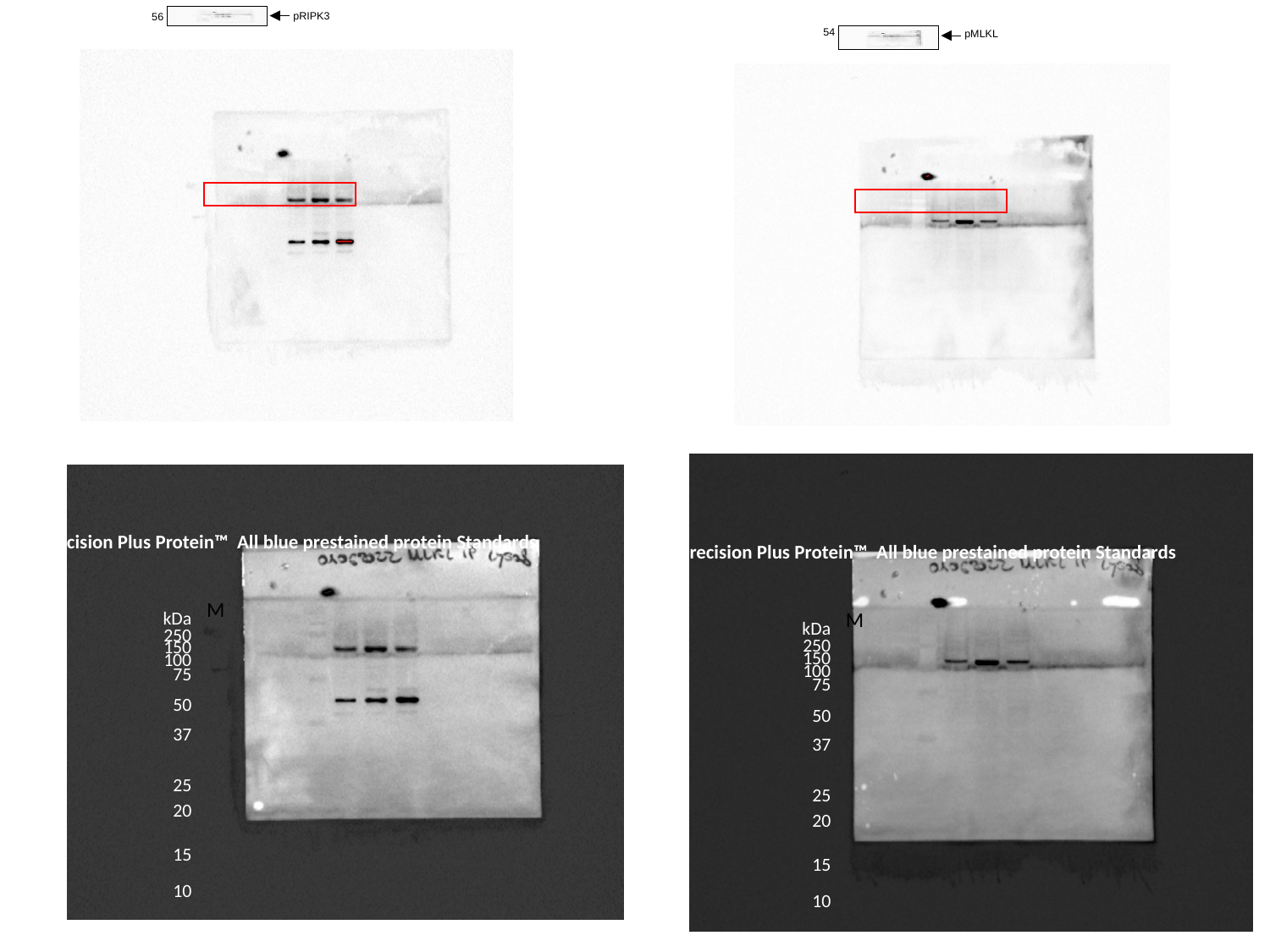

pRIPK3
56
54
pMLKL
Precision Plus Protein™ All blue prestained protein Standards
Precision Plus Protein™ All blue prestained protein Standards
M
kDa
M
kDa
250
250
150
150
100
100
75
75
50
50
37
37
25
25
20
20
15
15
10
10

## Slide 8
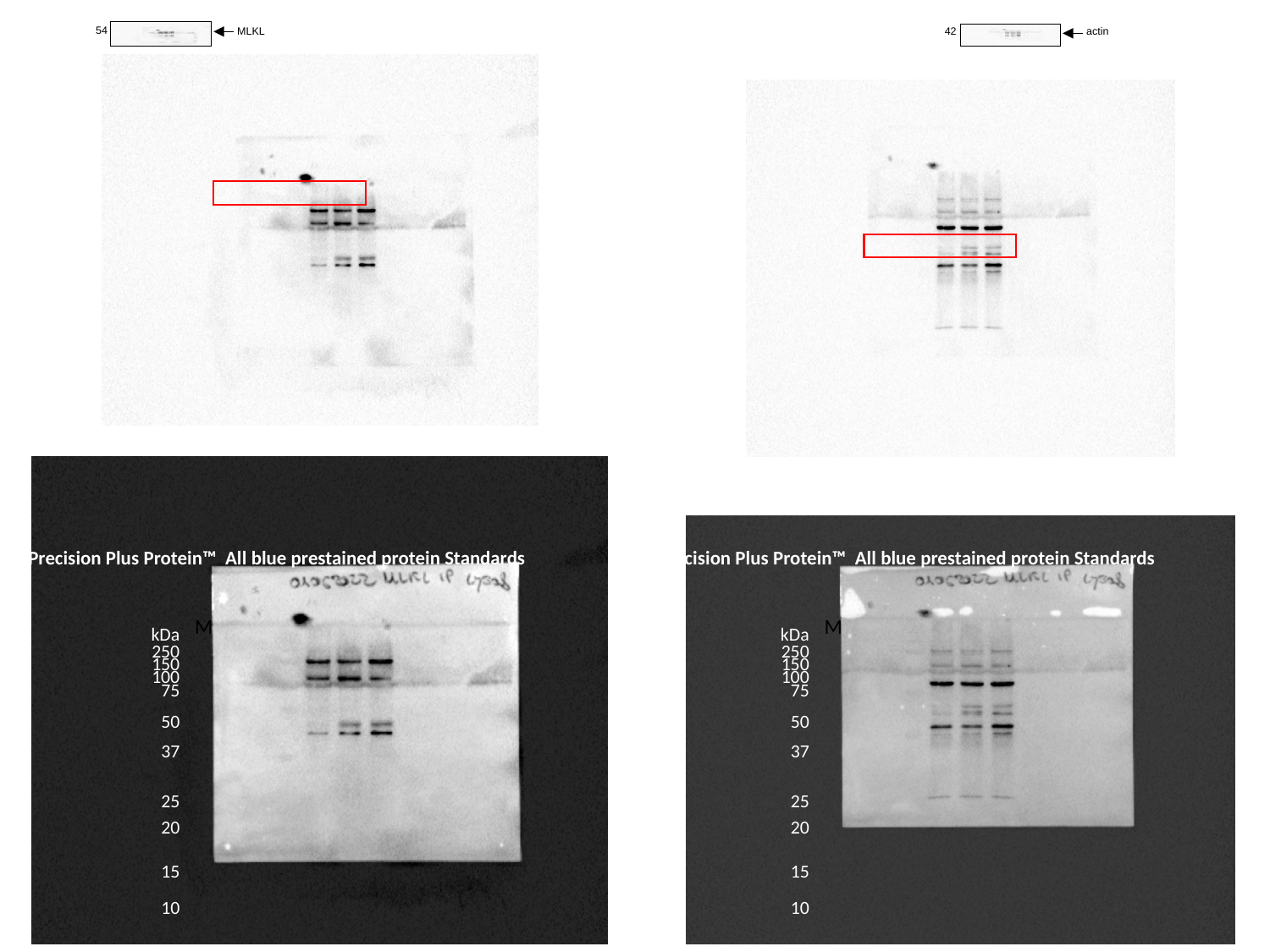

54
MLKL
42
actin
Precision Plus Protein™ All blue prestained protein Standards
Precision Plus Protein™ All blue prestained protein Standards
M
M
kDa
kDa
250
250
150
150
100
100
75
75
50
50
37
37
25
25
20
20
15
15
10
10

## Slide 9
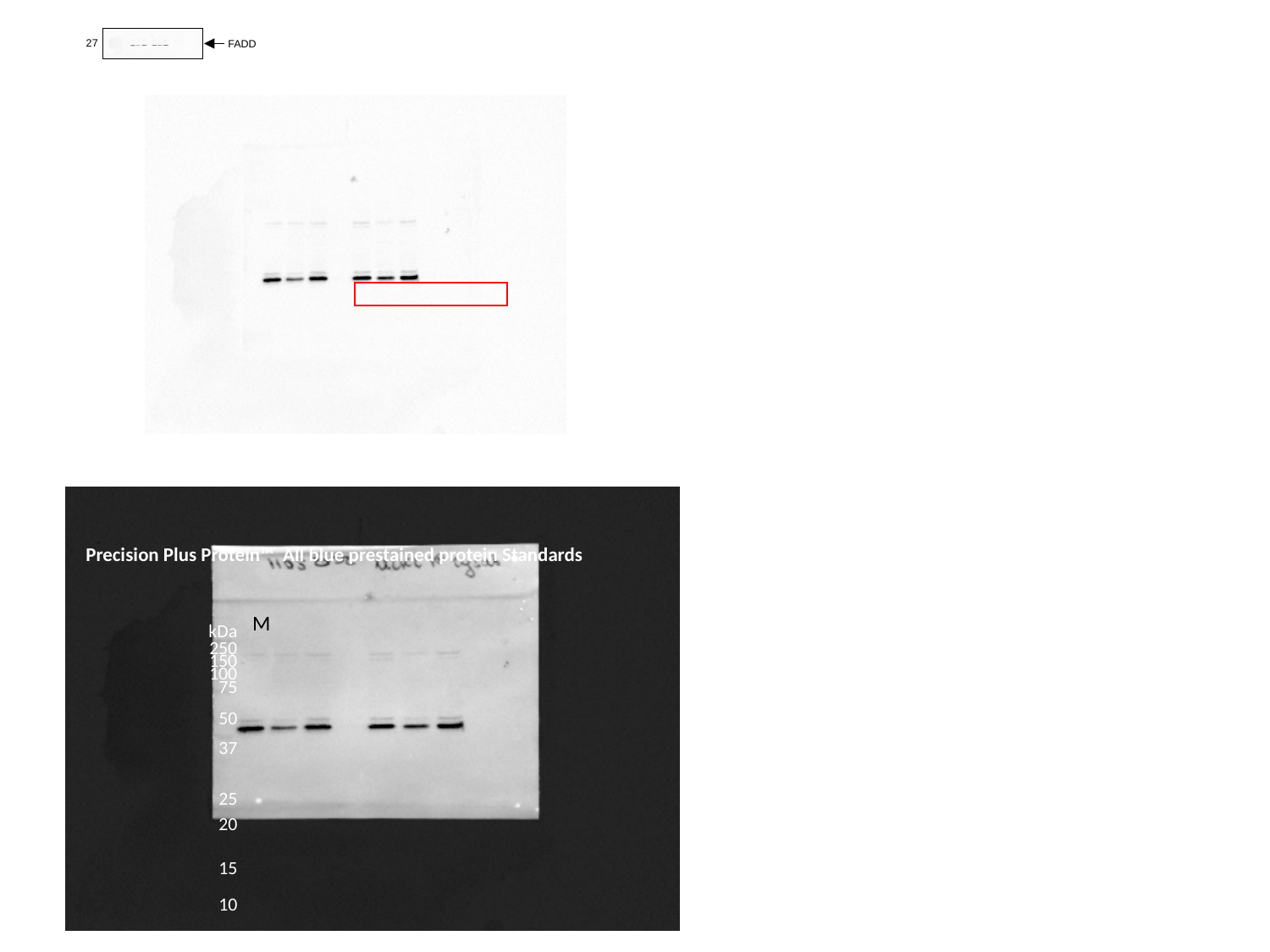

27
FADD
Precision Plus Protein™ All blue prestained protein Standards
M
kDa
250
150
100
75
50
37
25
20
15
10

## Slide 10
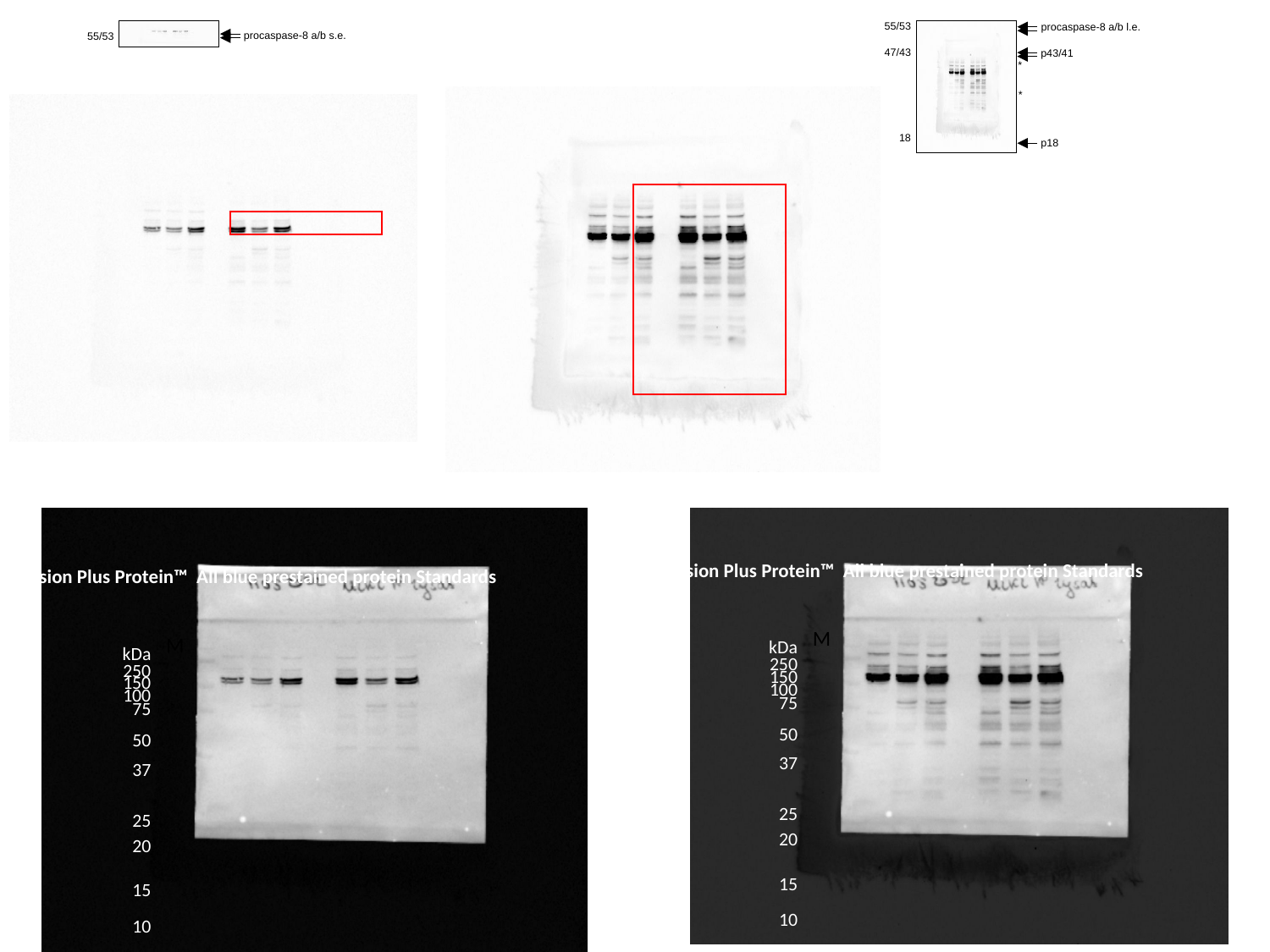

55/53
procaspase-8 a/b l.e.
procaspase-8 a/b s.e.
55/53
47/43
p43/41
*
*
18
p18
Precision Plus Protein™ All blue prestained protein Standards
Precision Plus Protein™ All blue prestained protein Standards
M
M
kDa
kDa
250
250
150
150
100
100
75
75
50
50
37
37
25
25
20
20
15
15
10
10

## Slide 11
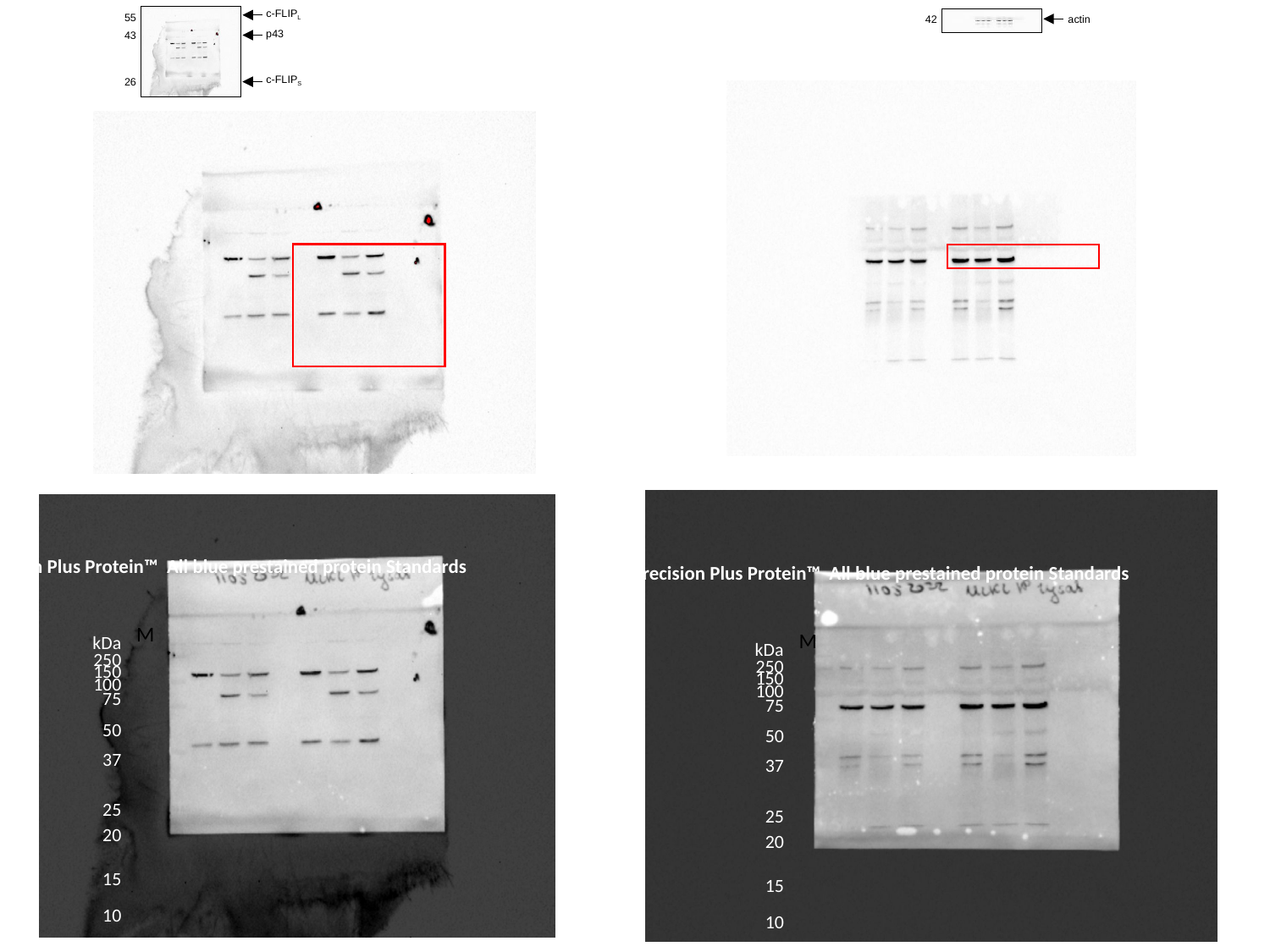

c-FLIPL
55
actin
42
p43
43
c-FLIPS
26
Precision Plus Protein™ All blue prestained protein Standards
Precision Plus Protein™ All blue prestained protein Standards
M
M
kDa
kDa
250
250
150
150
100
100
75
75
50
50
37
37
25
25
20
20
15
15
10
10
