# Supplementary material for: Oligomerised RIPK1 is the main core component of the CD95 necrosome
Source: EMBO J. 2025 Apr 16;44(11):3231–65. doi: 10.1038/s44318-025-00433-0 (PMC12130296; doi:10.1038/s44318-025-00433-0)
Supplement: Supplementary file 14 — Appendix Source Data [file 44318_2025_433_MOESM14_ESM.zip › S2A.pptx]

## Slide 1
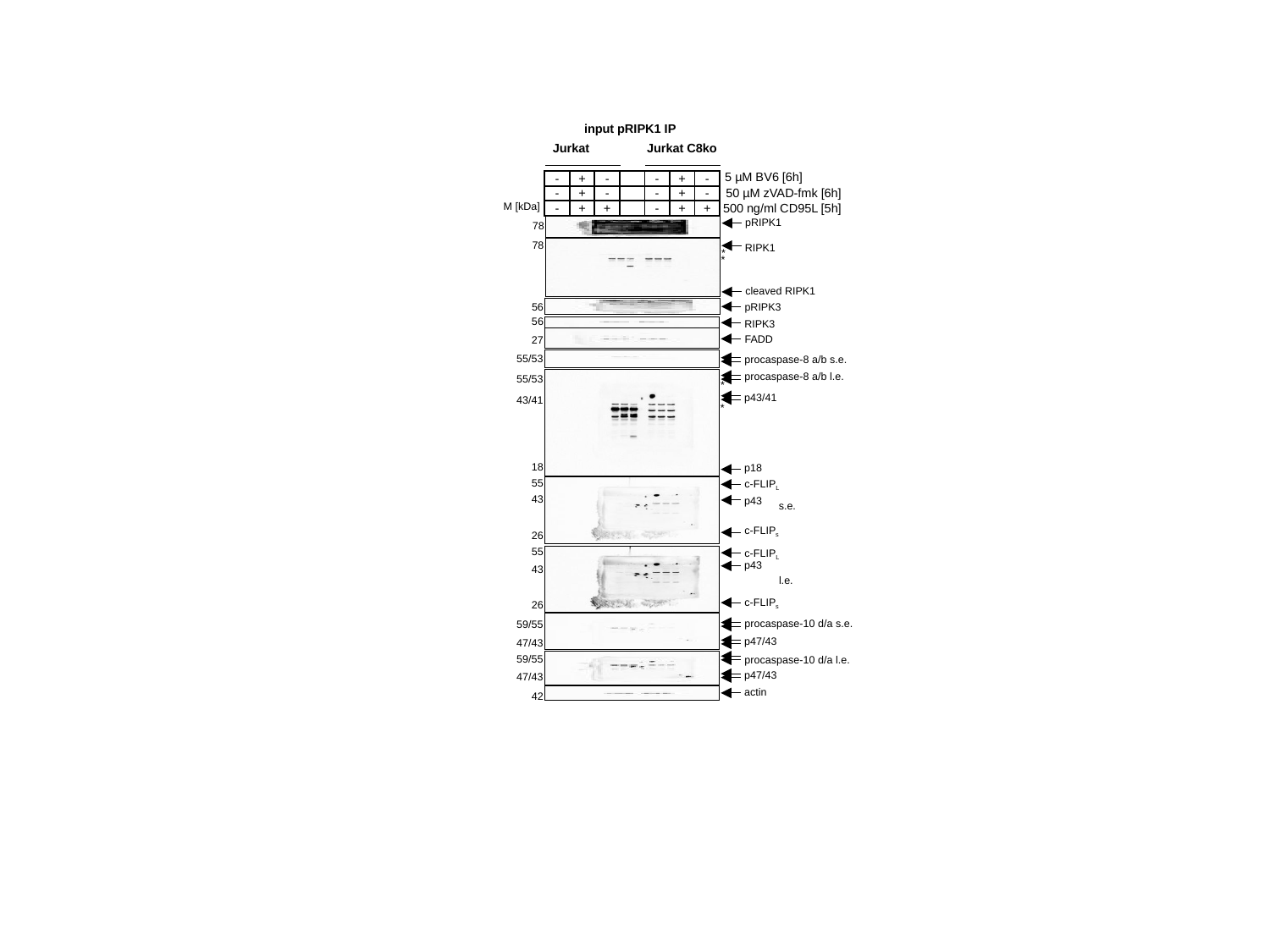

input pRIPK1 IP
Jurkat C8ko
Jurkat
5 µM BV6 [6h]
| - | + | - | | - | + | - |
| --- | --- | --- | --- | --- | --- | --- |
| - | + | - | | - | + | - |
| - | + | + | | - | + | + |
50 µM zVAD-fmk [6h]
M [kDa]
500 ng/ml CD95L [5h]
pRIPK1
78
78
RIPK1
*
*
cleaved RIPK1
pRIPK3
56
56
RIPK3
FADD
27
55/53
procaspase-8 a/b s.e.
procaspase-8 a/b l.e.
55/53
*
p43/41
43/41
*
18
p18
55
c-FLIPL
43
p43
s.e.
c-FLIPs
26
55
c-FLIPL
p43
43
l.e.
c-FLIPs
26
procaspase-10 d/a s.e.
59/55
p47/43
47/43
59/55
procaspase-10 d/a l.e.
p47/43
47/43
actin
42

## Slide 2
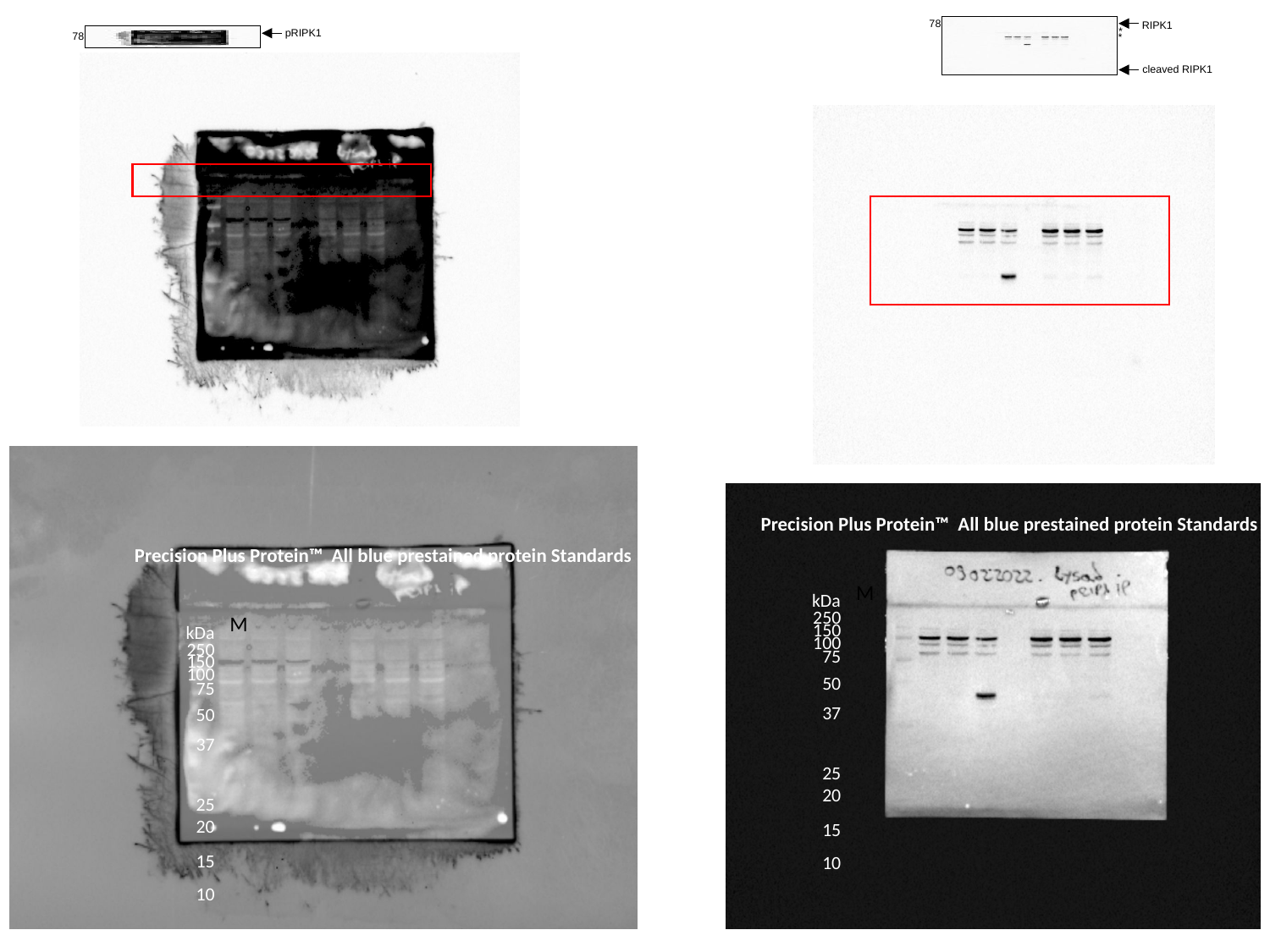

78
RIPK1
*
pRIPK1
78
*
cleaved RIPK1
Precision Plus Protein™ All blue prestained protein Standards
Precision Plus Protein™ All blue prestained protein Standards
M
kDa
250
M
150
kDa
100
250
75
150
100
50
75
37
50
37
25
20
25
20
15
15
10
10

## Slide 3
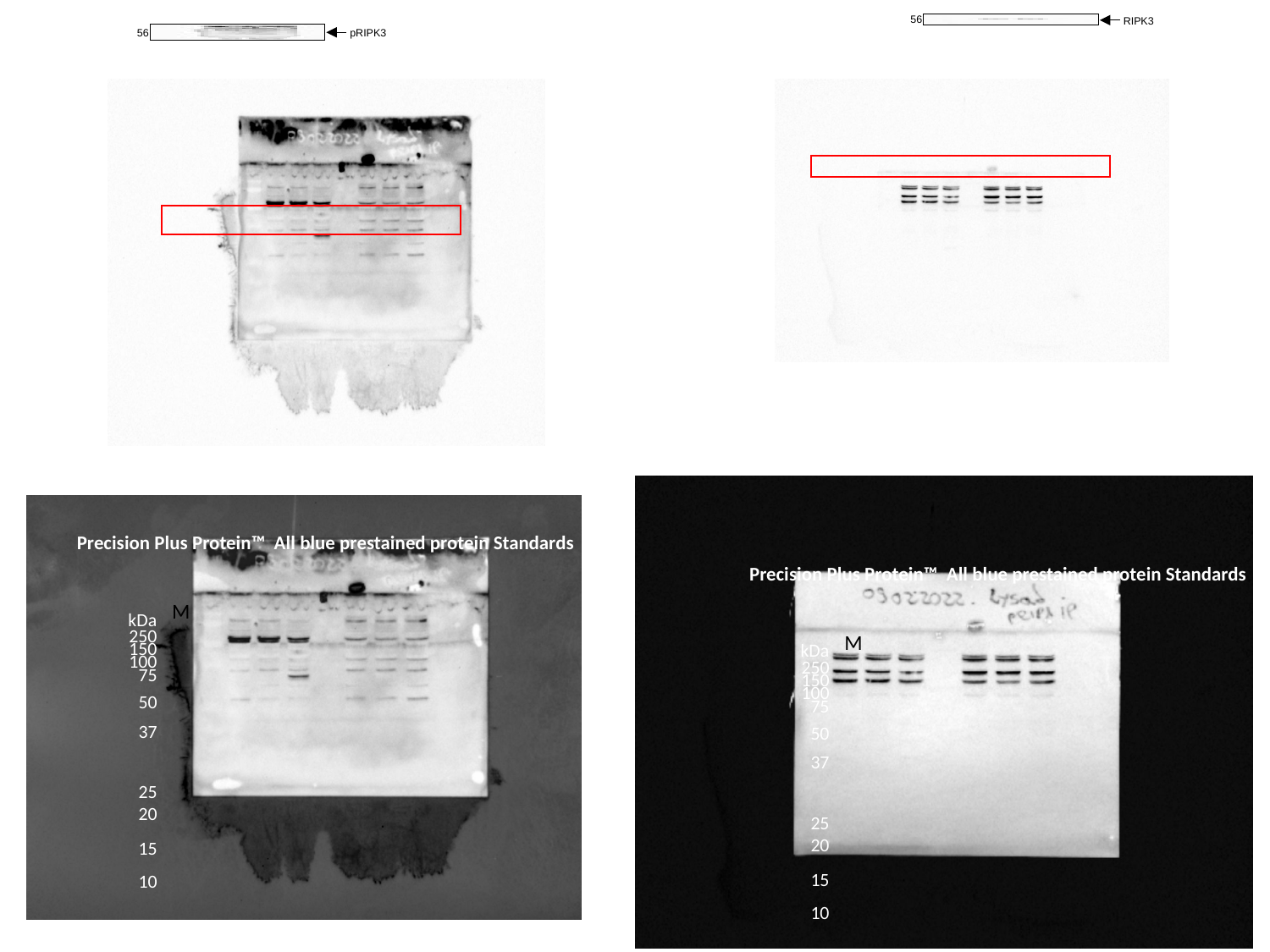

56
RIPK3
pRIPK3
56
Precision Plus Protein™ All blue prestained protein Standards
Precision Plus Protein™ All blue prestained protein Standards
M
kDa
250
M
150
kDa
100
250
75
150
100
50
75
37
50
37
25
20
25
20
15
15
10
10

## Slide 4
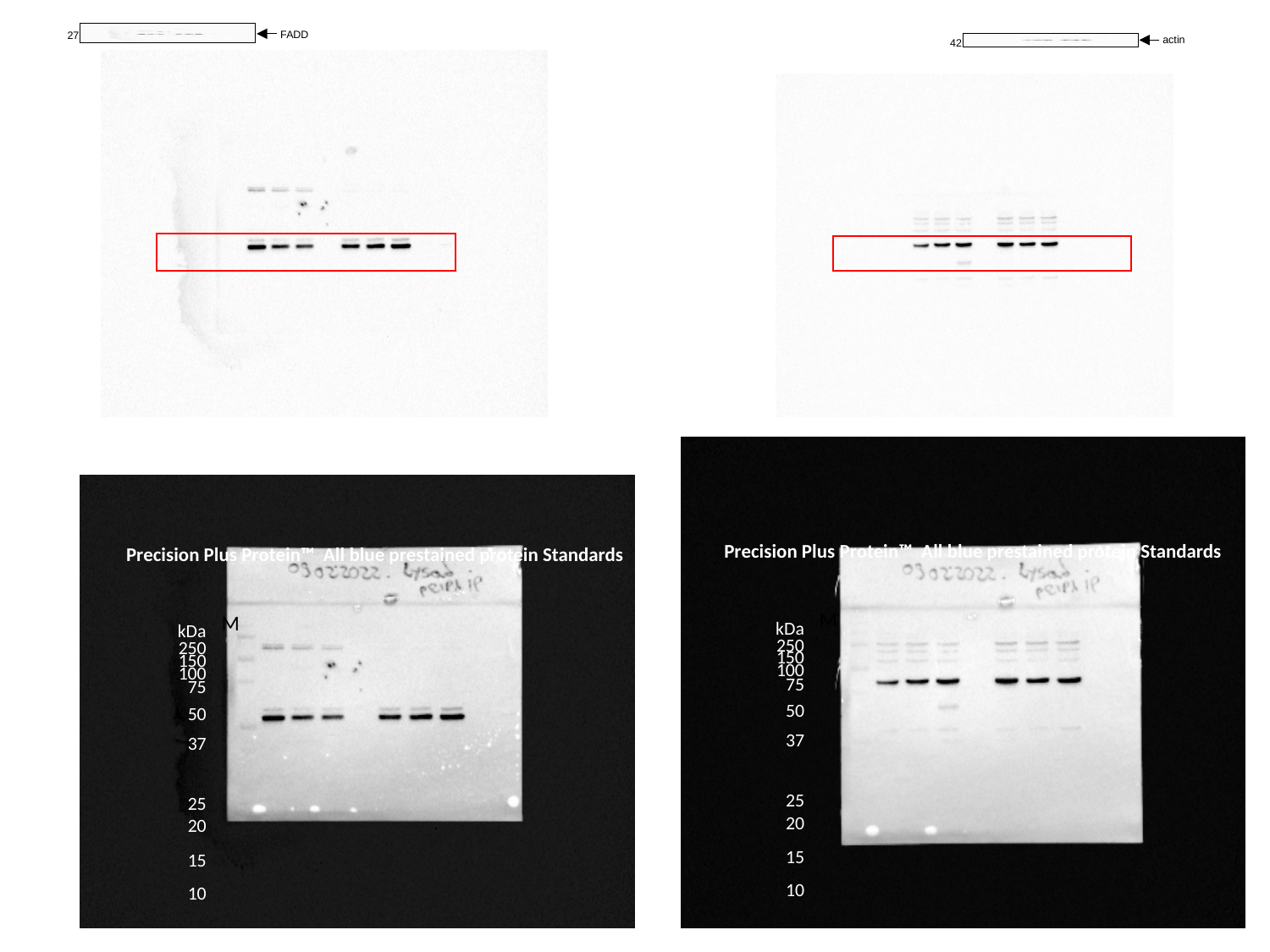

FADD
27
actin
42
Precision Plus Protein™ All blue prestained protein Standards
Precision Plus Protein™ All blue prestained protein Standards
M
M
kDa
kDa
250
250
150
150
100
100
75
75
50
50
37
37
25
25
20
20
15
15
10
10

## Slide 5
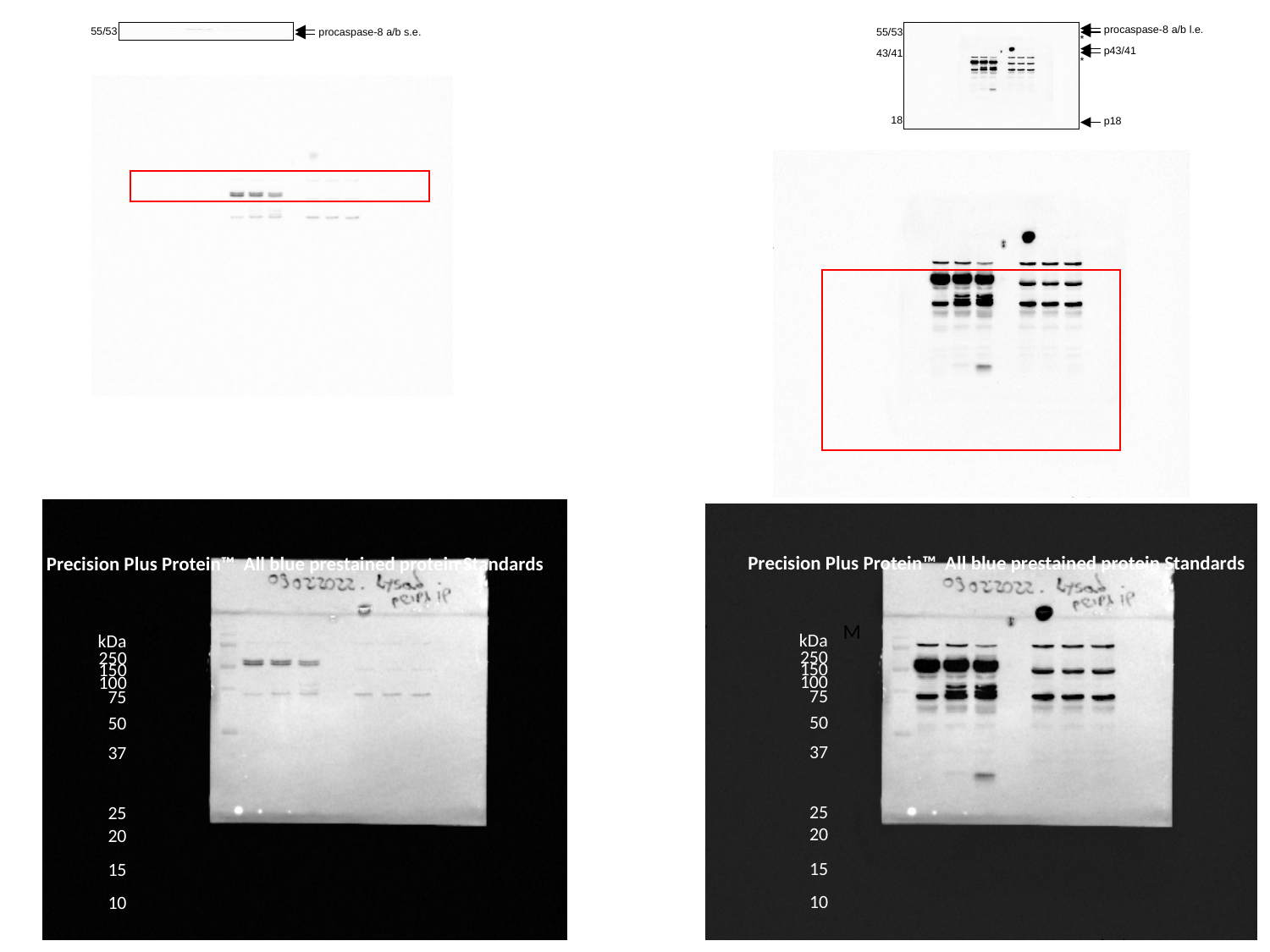

procaspase-8 a/b l.e.
55/53
procaspase-8 a/b s.e.
55/53
*
p43/41
43/41
*
18
p18
Precision Plus Protein™ All blue prestained protein Standards
Precision Plus Protein™ All blue prestained protein Standards
M
M
kDa
kDa
250
250
150
150
100
100
75
75
50
50
37
37
25
25
20
20
15
15
10
10

## Slide 6
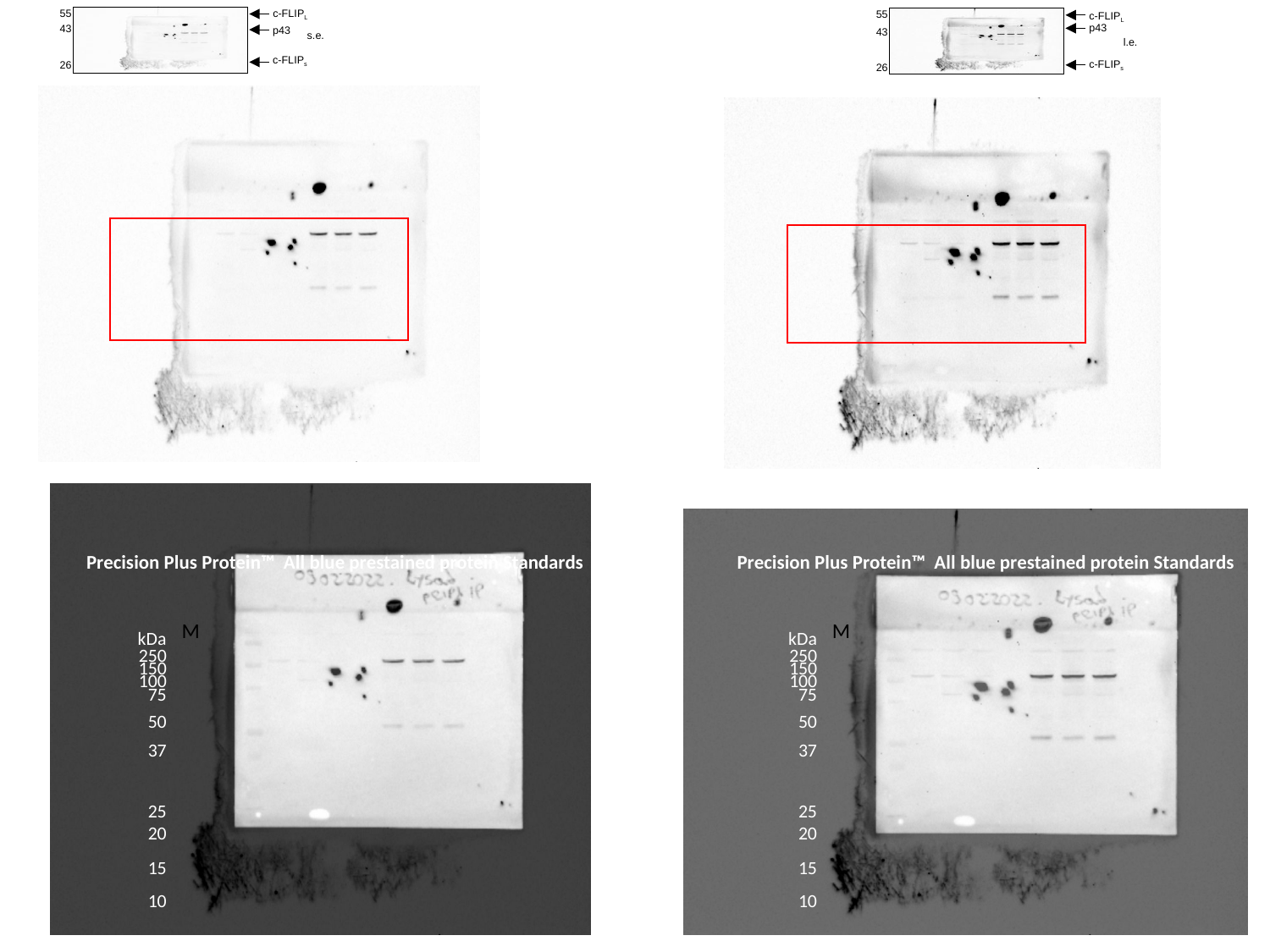

55
c-FLIPL
55
c-FLIPL
p43
43
p43
43
s.e.
l.e.
c-FLIPs
c-FLIPs
26
26
Precision Plus Protein™ All blue prestained protein Standards
Precision Plus Protein™ All blue prestained protein Standards
M
M
kDa
kDa
250
250
150
150
100
100
75
75
50
50
37
37
25
25
20
20
15
15
10
10

## Slide 7
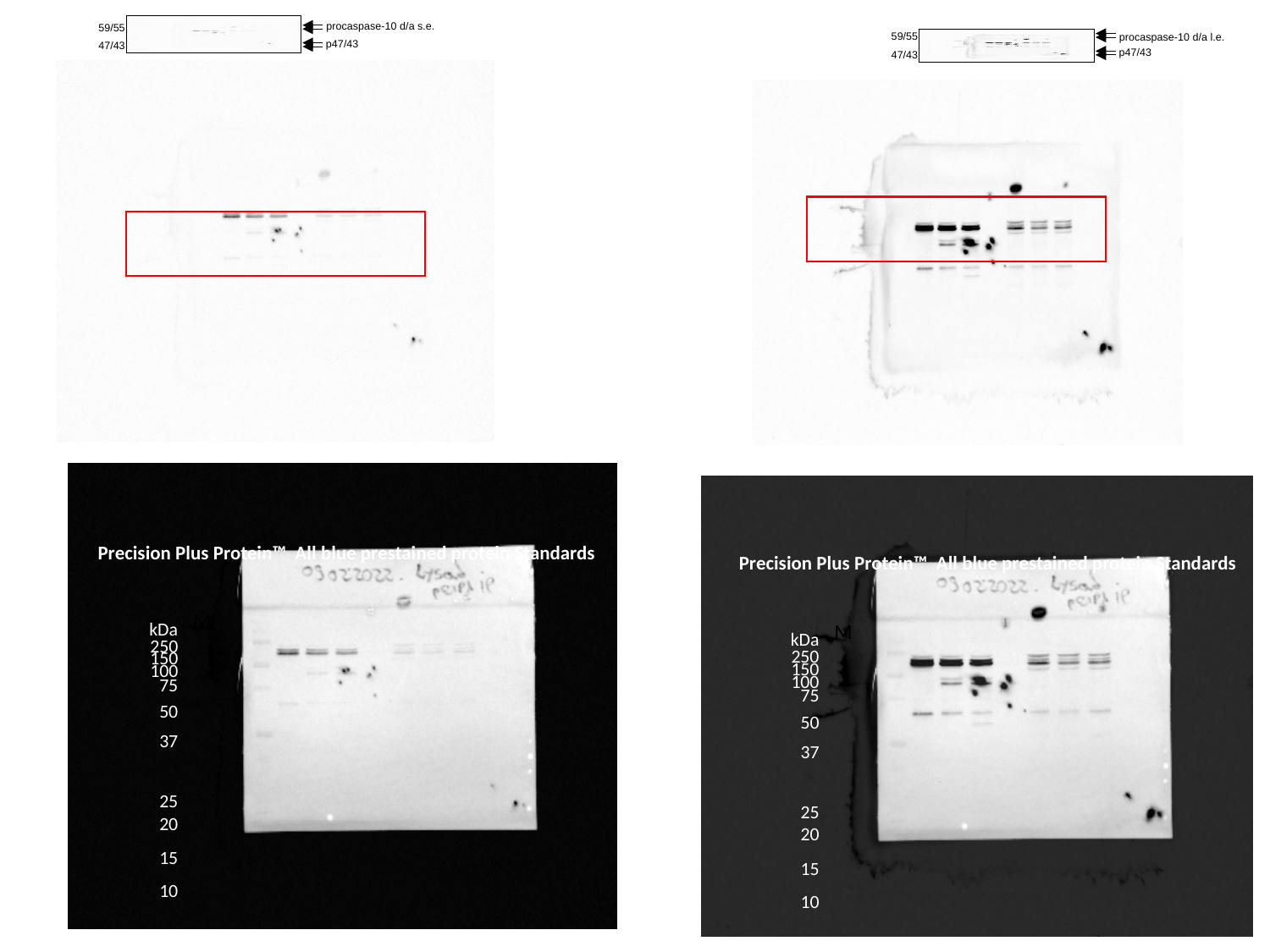

procaspase-10 d/a s.e.
59/55
59/55
procaspase-10 d/a l.e.
p47/43
47/43
p47/43
47/43
Precision Plus Protein™ All blue prestained protein Standards
Precision Plus Protein™ All blue prestained protein Standards
M
kDa
M
kDa
250
250
150
150
100
100
75
75
50
50
37
37
25
25
20
20
15
15
10
10
