# Supplementary material for: Oligomerised RIPK1 is the main core component of the CD95 necrosome
Source: EMBO J. 2025 Apr 16;44(11):3231–65. doi: 10.1038/s44318-025-00433-0 (PMC12130296; doi:10.1038/s44318-025-00433-0)
Supplement: Supplementary file 14 — Appendix Source Data [file 44318_2025_433_MOESM14_ESM.zip › S2C.pptx]

## Slide 1
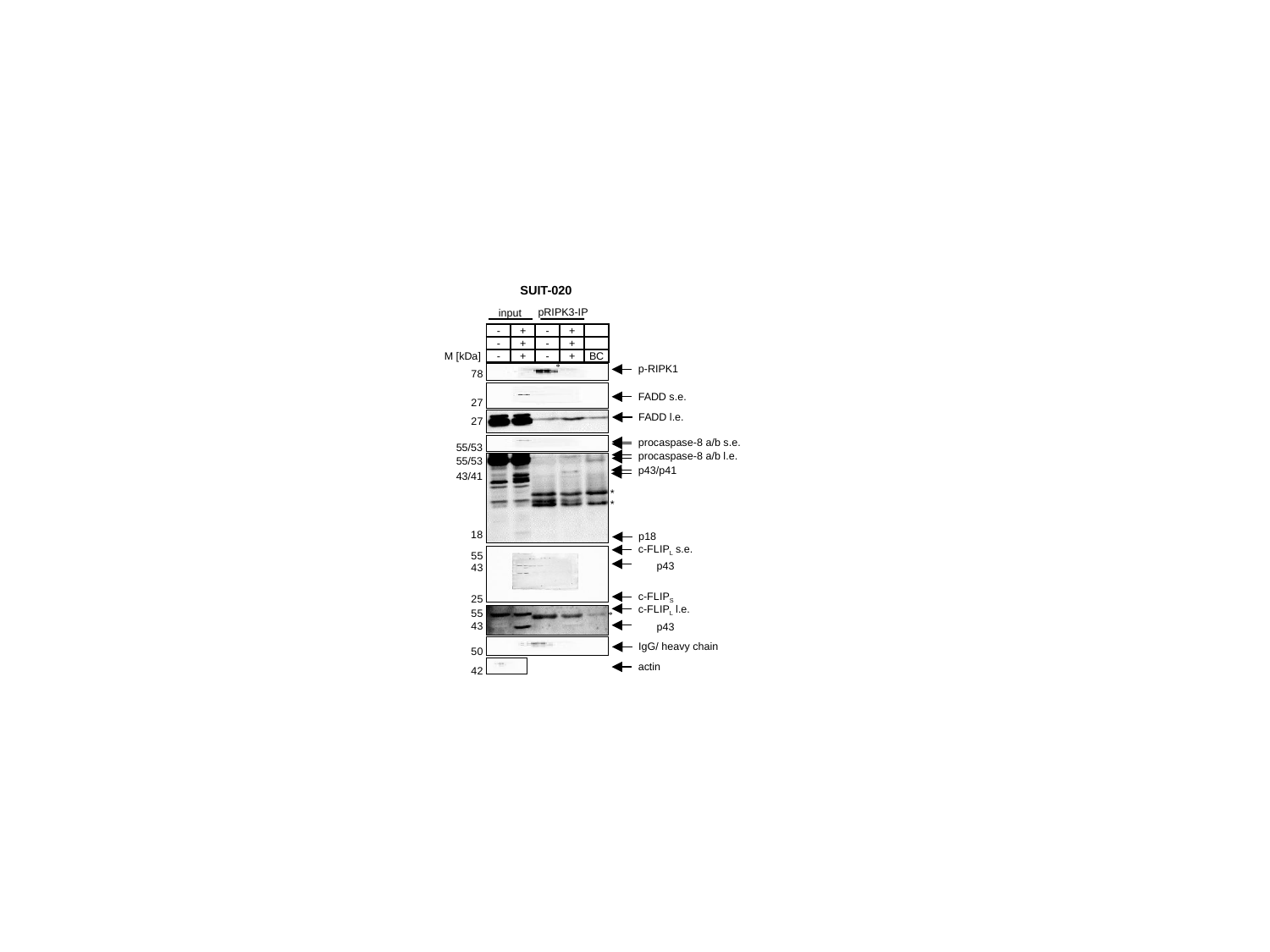

SUIT-020
pRIPK3-IP
input
| - | + | - | + | |
| --- | --- | --- | --- | --- |
| - | + | - | + | |
| - | + | - | + | BC |
M [kDa]
*
*
p-RIPK1
78
FADD s.e.
27
FADD l.e.
27
procaspase-8 a/b s.e.
55/53
procaspase-8 a/b l.e.
55/53
p43/p41
43/41
*
*
18
p18
c-FLIPL s.e.
55
p43
43
c-FLIPS
25
c-FLIPL l.e.
55
43
p43
IgG/ heavy chain
50
actin
42

## Slide 2
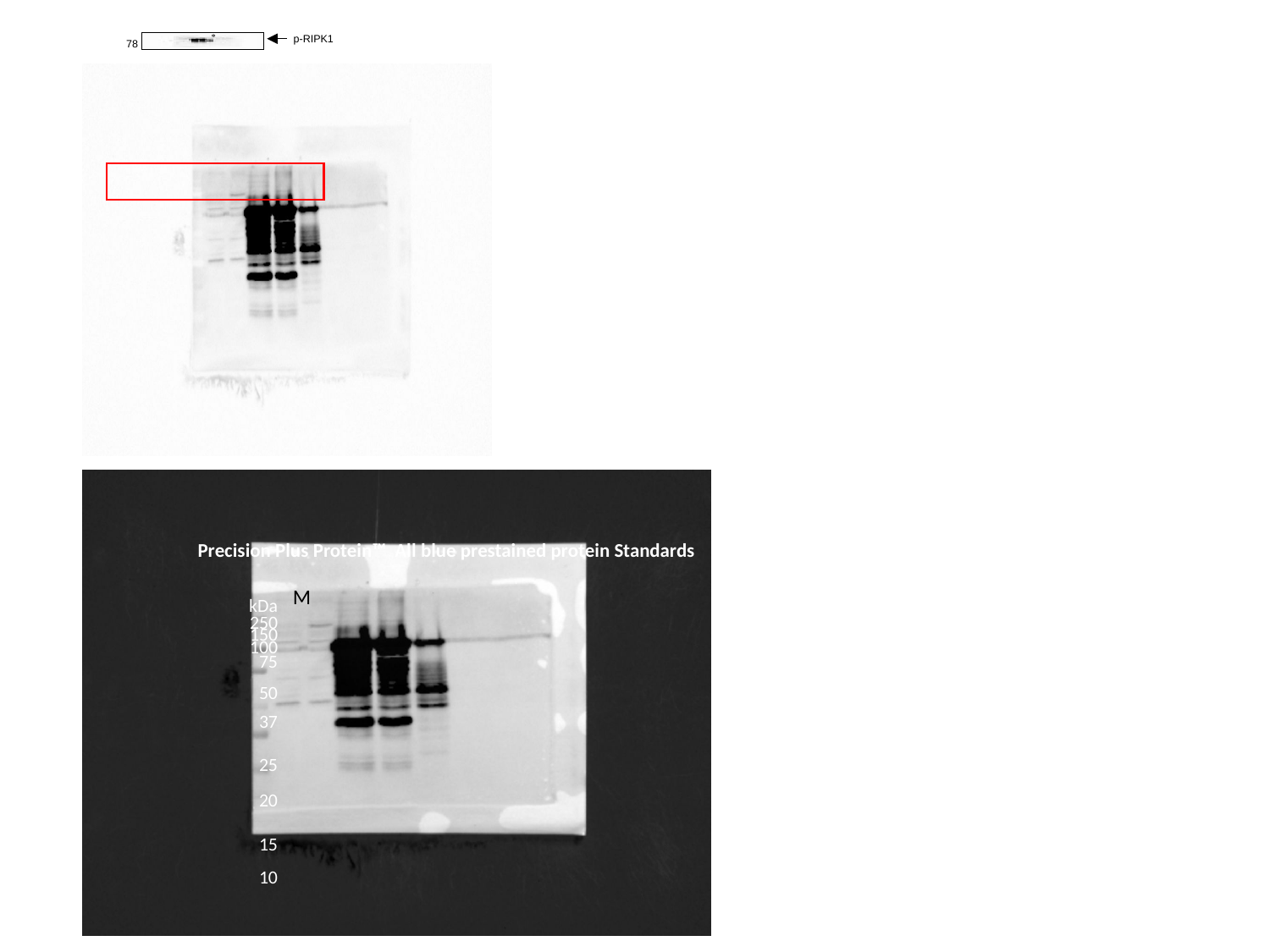

*
p-RIPK1
78
Precision Plus Protein™ All blue prestained protein Standards
M
kDa
250
150
100
75
50
37
25
20
15
10

## Slide 3
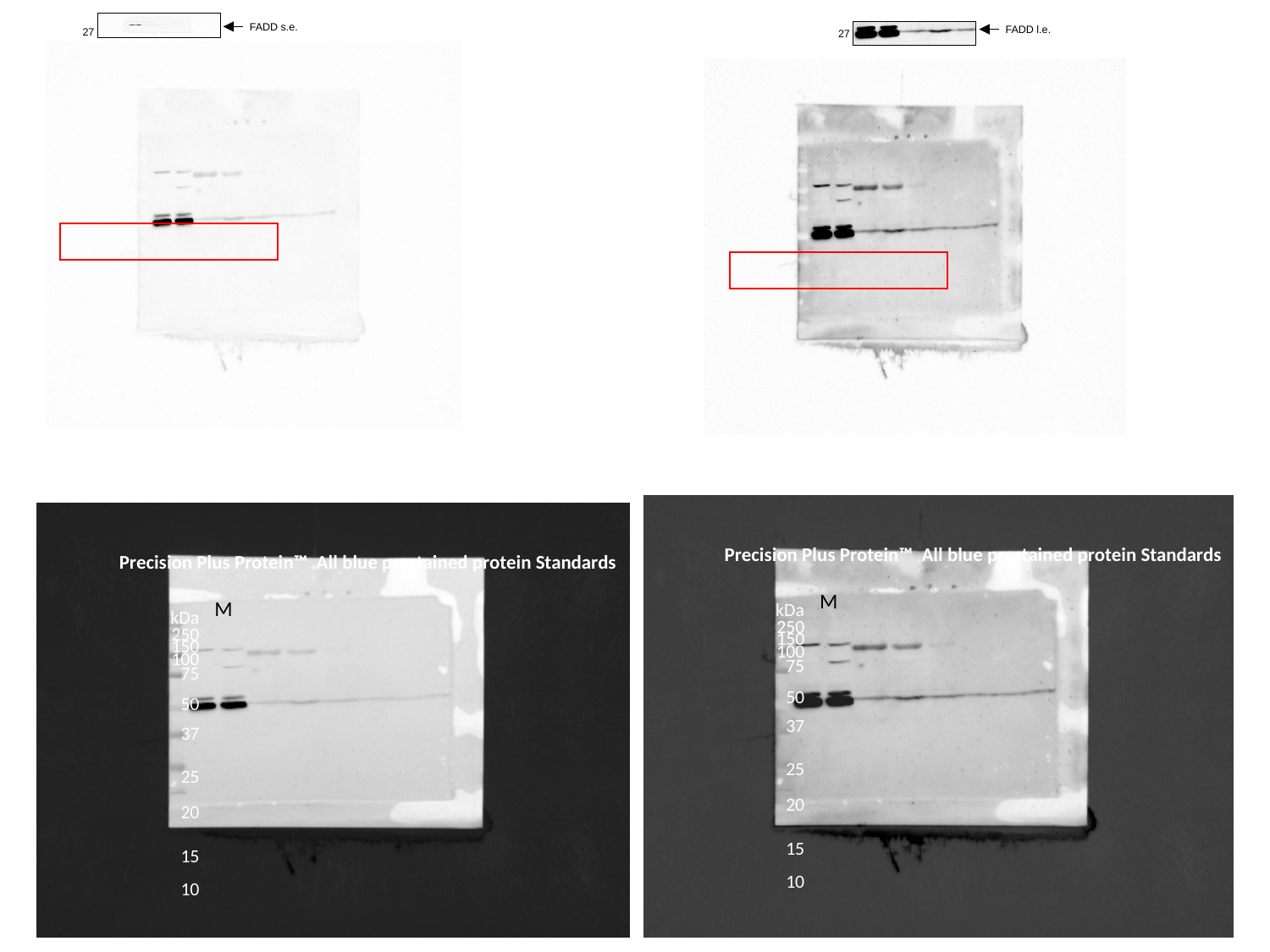

FADD s.e.
FADD l.e.
27
27
Precision Plus Protein™ All blue prestained protein Standards
Precision Plus Protein™ All blue prestained protein Standards
M
M
kDa
kDa
250
250
150
150
100
100
75
75
50
50
37
37
25
25
20
20
15
15
10
10

## Slide 4
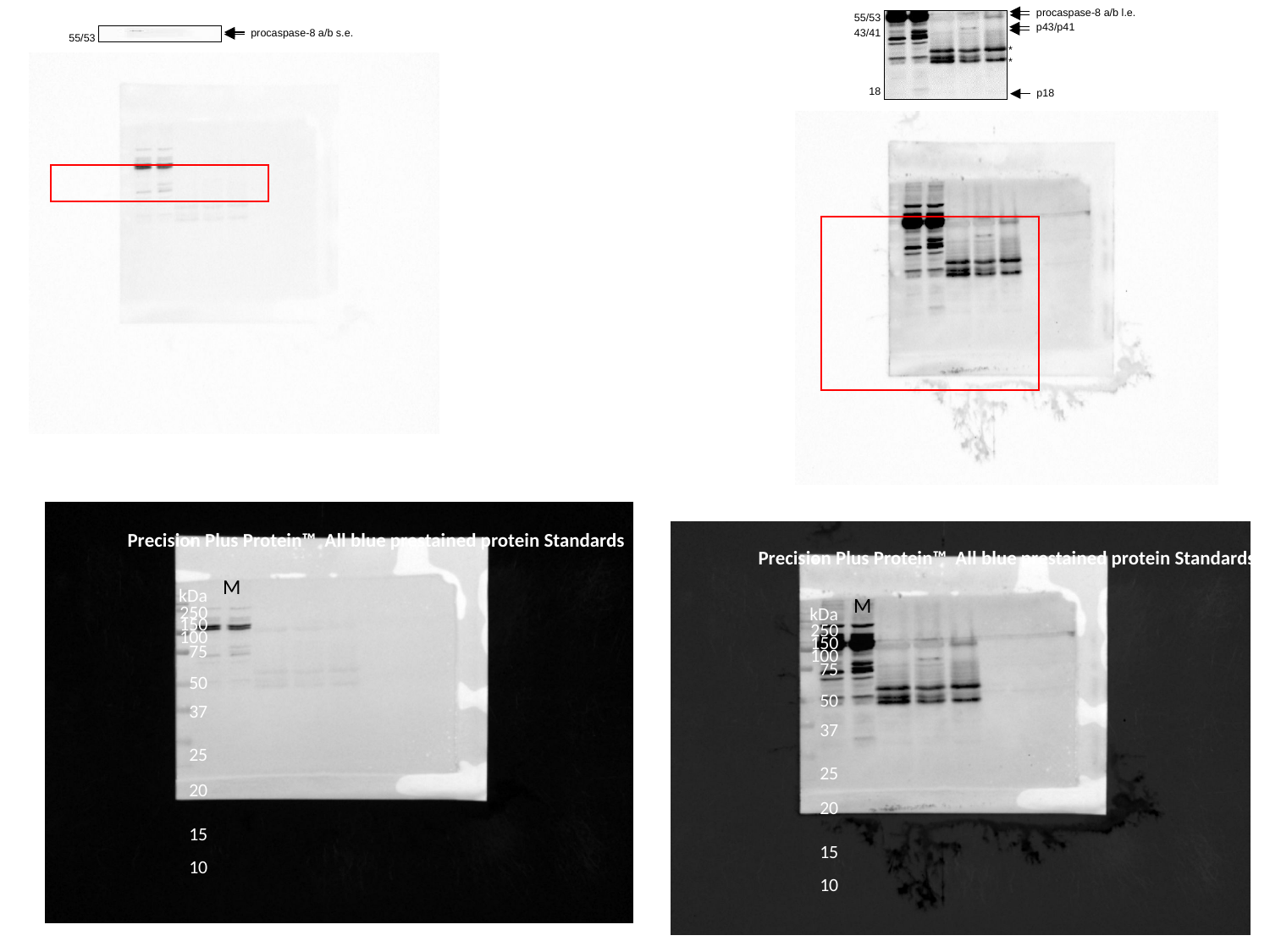

procaspase-8 a/b l.e.
55/53
p43/p41
procaspase-8 a/b s.e.
43/41
55/53
*
*
18
p18
Precision Plus Protein™ All blue prestained protein Standards
Precision Plus Protein™ All blue prestained protein Standards
M
kDa
M
250
kDa
150
250
100
150
75
100
75
50
50
37
37
25
25
20
20
15
15
10
10

## Slide 5
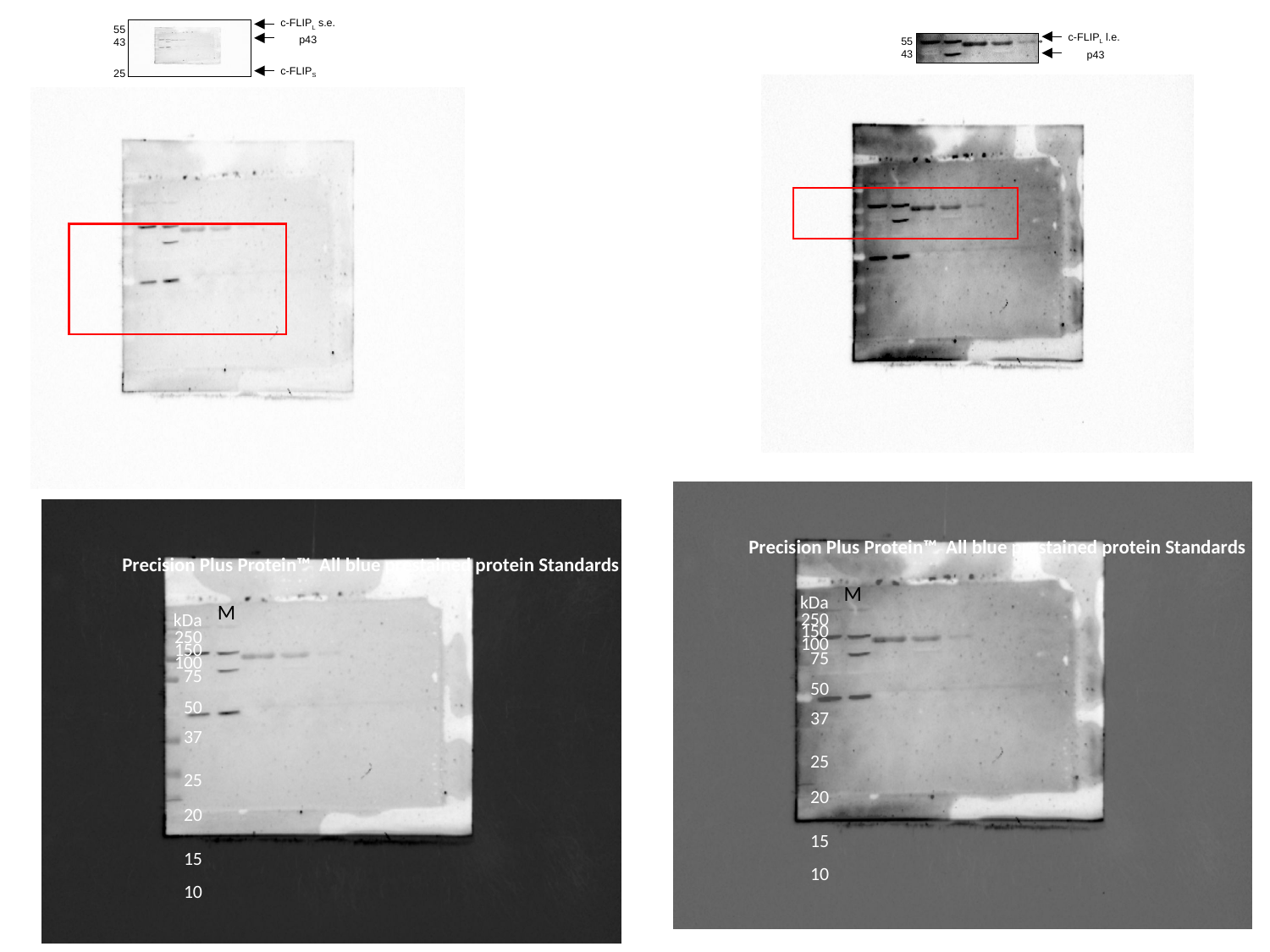

c-FLIPL s.e.
55
c-FLIPL l.e.
p43
55
43
*
43
p43
c-FLIPS
25
Precision Plus Protein™ All blue prestained protein Standards
Precision Plus Protein™ All blue prestained protein Standards
M
kDa
M
250
kDa
150
250
100
150
75
100
75
50
50
37
37
25
25
20
20
15
15
10
10

## Slide 6
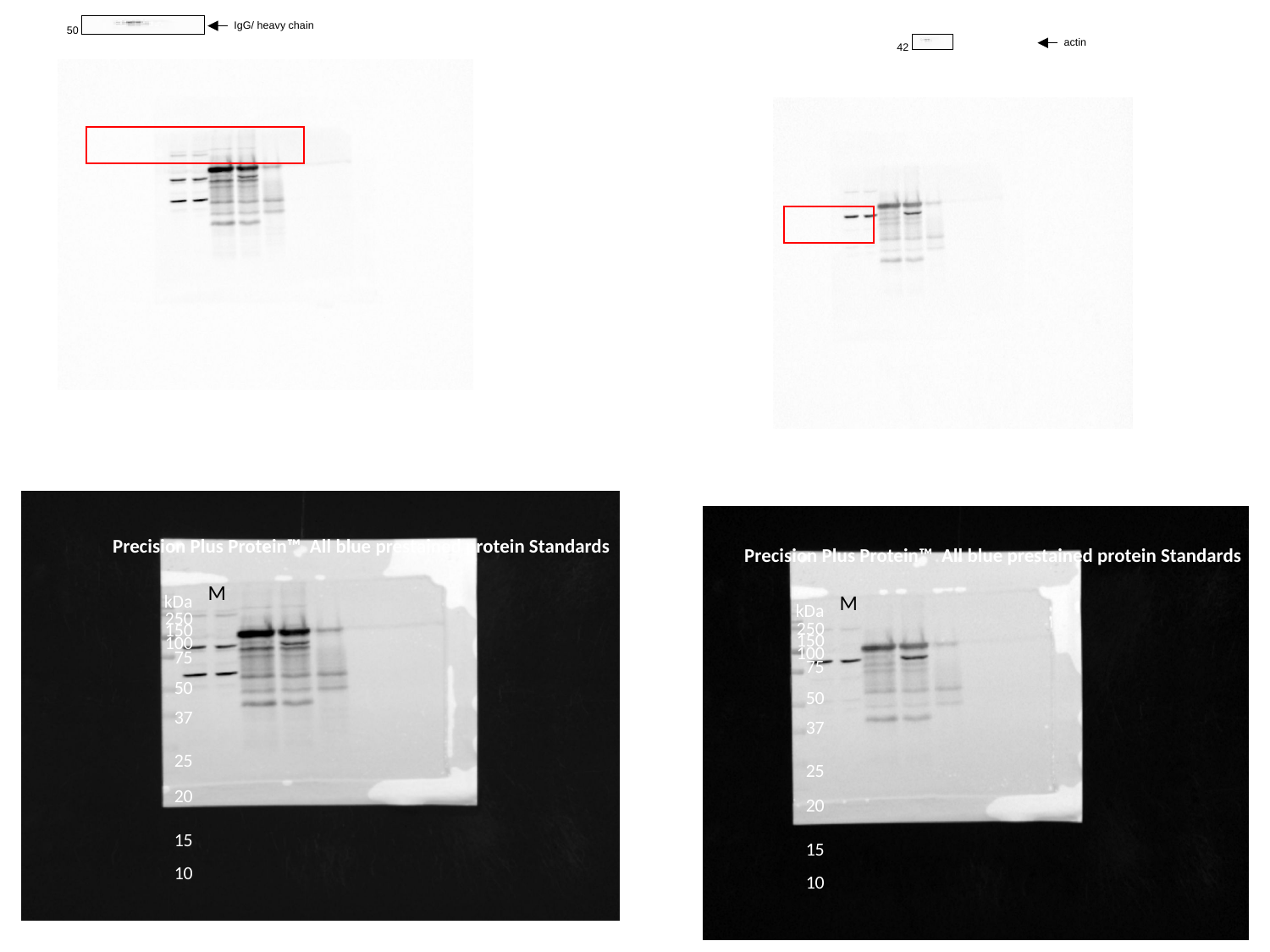

IgG/ heavy chain
50
actin
42
Precision Plus Protein™ All blue prestained protein Standards
Precision Plus Protein™ All blue prestained protein Standards
M
M
kDa
kDa
250
250
150
150
100
100
75
75
50
50
37
37
25
25
20
20
15
15
10
10
