# Supplementary material for: Oligomerised RIPK1 is the main core component of the CD95 necrosome
Source: EMBO J. 2025 Apr 16;44(11):3231–65. doi: 10.1038/s44318-025-00433-0 (PMC12130296; doi:10.1038/s44318-025-00433-0)
Supplement: Supplementary file 14 — Appendix Source Data [file 44318_2025_433_MOESM14_ESM.zip › S2D.pptx]

## Slide 1
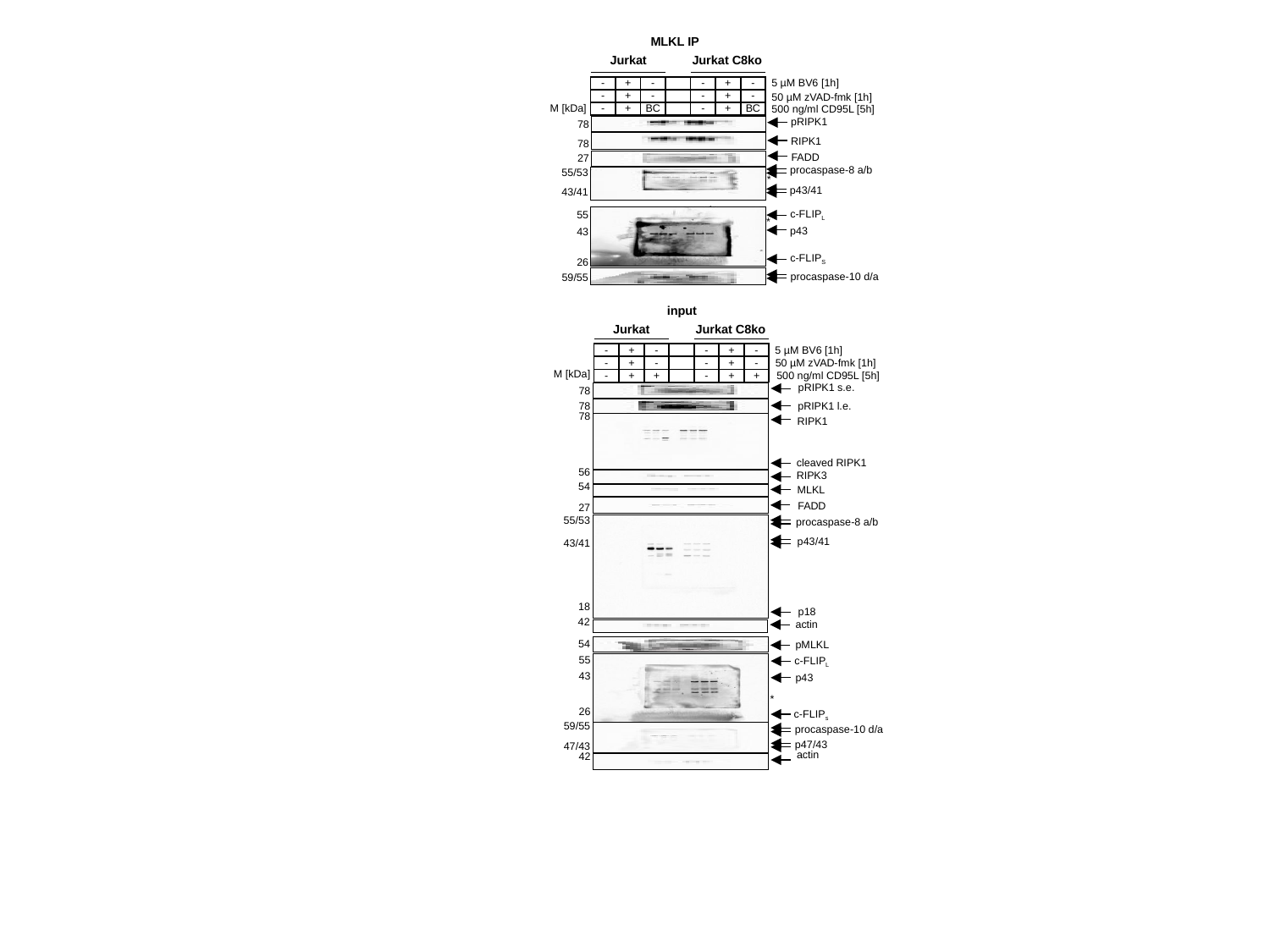

MLKL IP
Jurkat C8ko
Jurkat
5 µM BV6 [1h]
| - | + | - | | - | + | - |
| --- | --- | --- | --- | --- | --- | --- |
| - | + | - | | - | + | - |
| - | + | BC | | - | + | BC |
50 µM zVAD-fmk [1h]
M [kDa]
500 ng/ml CD95L [5h]
pRIPK1
78
RIPK1
78
FADD
27
procaspase-8 a/b
55/53
*
p43/41
43/41
c-FLIPL
55
*
p43
43
c-FLIPS
26
procaspase-10 d/a
59/55
input
Jurkat C8ko
Jurkat
5 µM BV6 [1h]
| - | + | - | | - | + | - |
| --- | --- | --- | --- | --- | --- | --- |
| - | + | - | | - | + | - |
| - | + | + | | - | + | + |
50 µM zVAD-fmk [1h]
M [kDa]
500 ng/ml CD95L [5h]
pRIPK1 s.e.
78
pRIPK1 l.e.
78
78
RIPK1
cleaved RIPK1
56
RIPK3
54
MLKL
FADD
27
55/53
procaspase-8 a/b
p43/41
43/41
18
p18
42
actin
54
pMLKL
55
c-FLIPL
43
p43
*
26
c-FLIPs
59/55
procaspase-10 d/a
p47/43
47/43
actin
42

## Slide 2
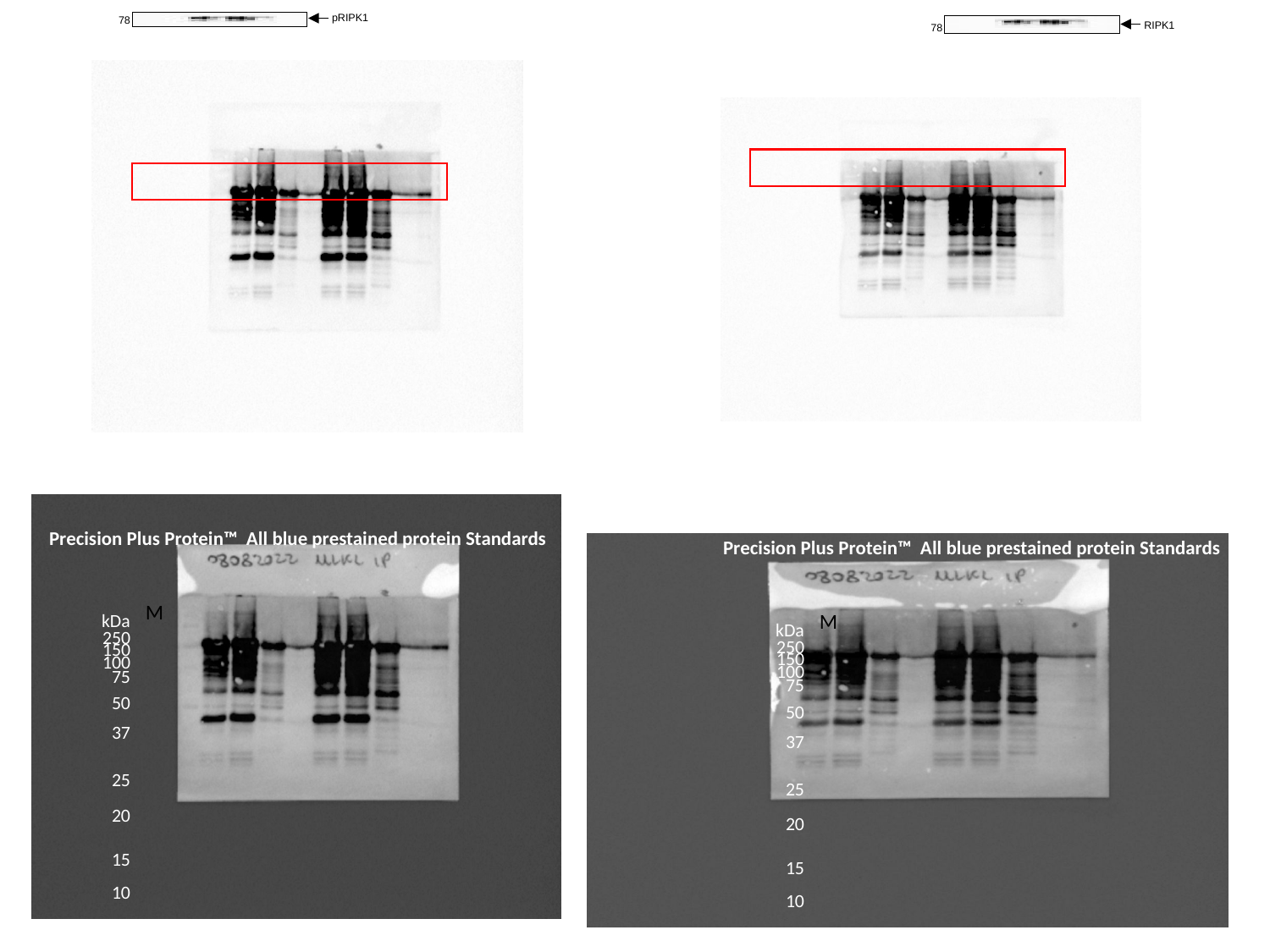

pRIPK1
78
RIPK1
78
Precision Plus Protein™ All blue prestained protein Standards
Precision Plus Protein™ All blue prestained protein Standards
M
M
kDa
kDa
250
250
150
150
100
100
75
75
50
50
37
37
25
25
20
20
15
15
10
10

## Slide 3
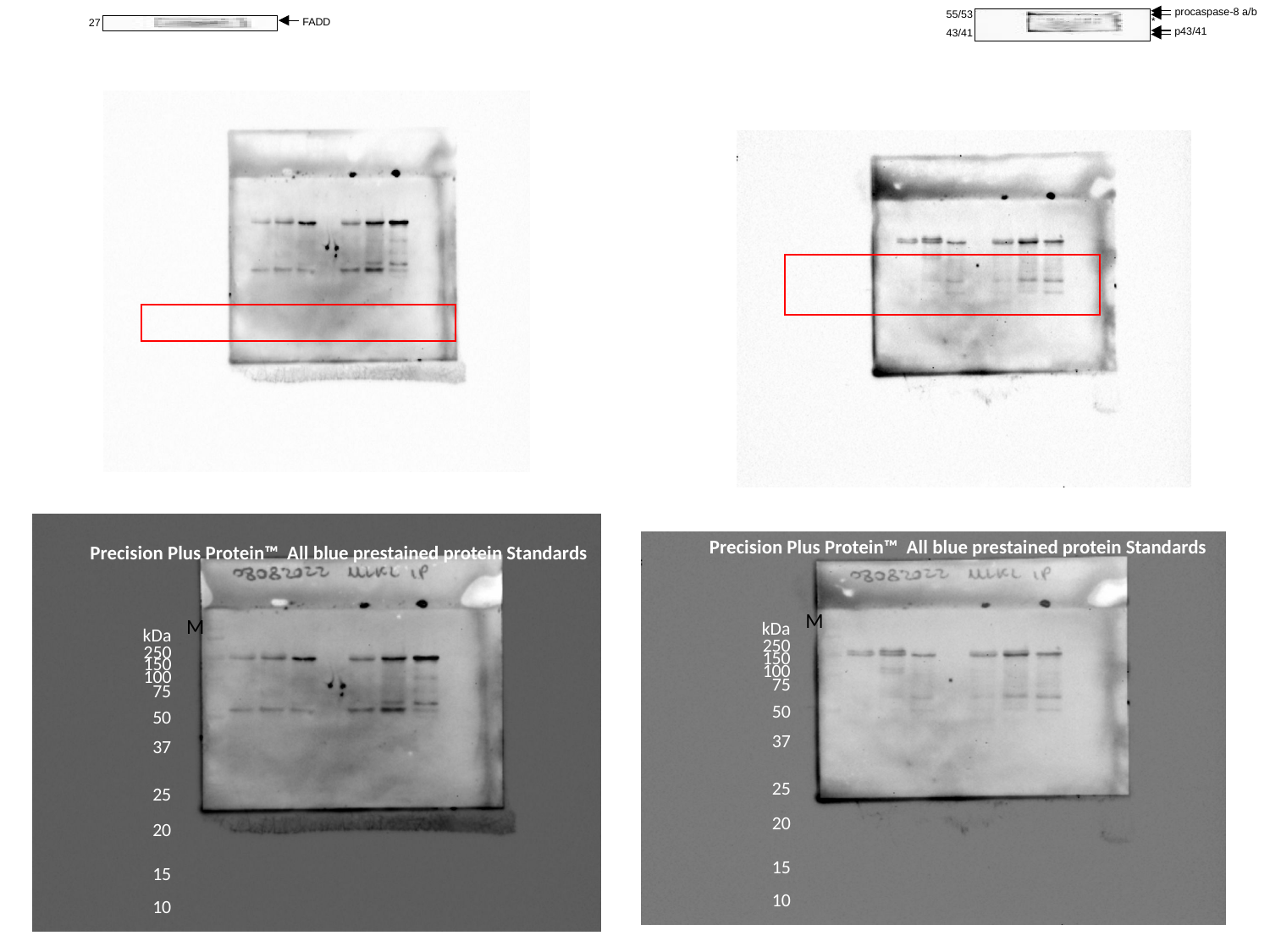

procaspase-8 a/b
55/53
*
FADD
27
p43/41
43/41
Precision Plus Protein™ All blue prestained protein Standards
Precision Plus Protein™ All blue prestained protein Standards
M
M
kDa
kDa
250
250
150
150
100
100
75
75
50
50
37
37
25
25
20
20
15
15
10
10

## Slide 4
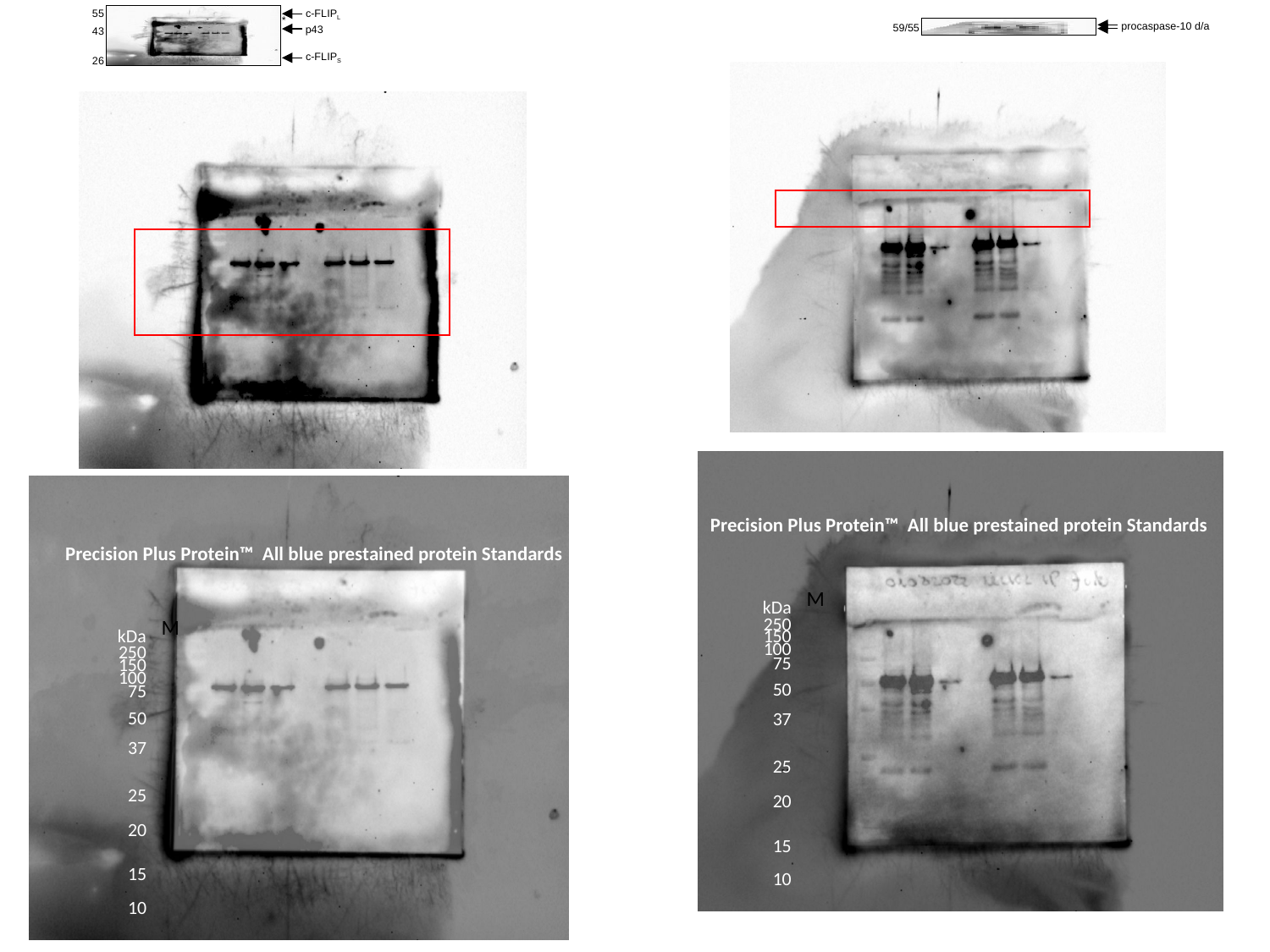

c-FLIPL
55
*
procaspase-10 d/a
59/55
p43
43
c-FLIPS
26
Precision Plus Protein™ All blue prestained protein Standards
Precision Plus Protein™ All blue prestained protein Standards
M
kDa
250
M
kDa
150
100
250
75
150
100
50
75
50
37
37
25
25
20
20
15
15
10
10

## Slide 5
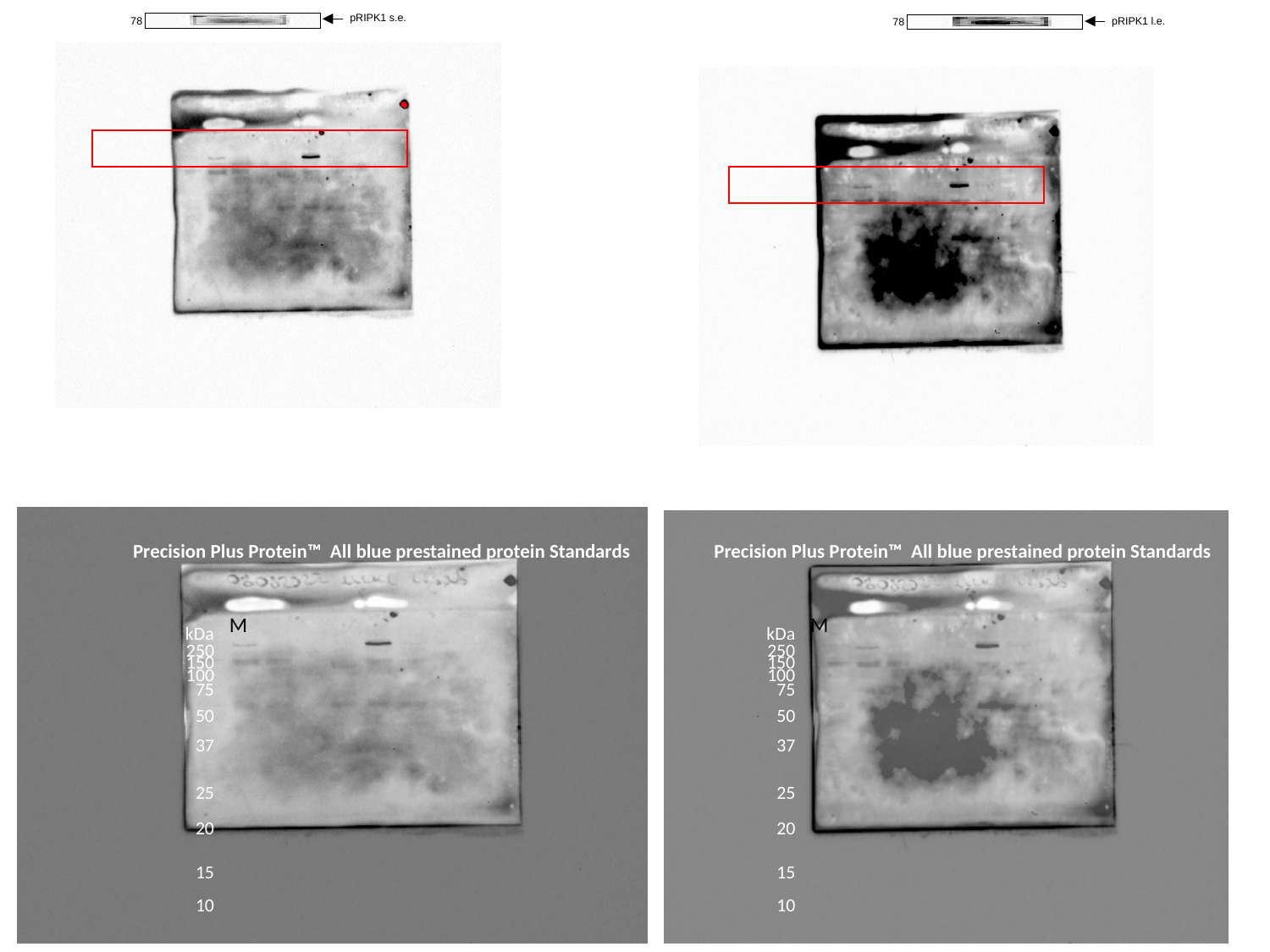

pRIPK1 s.e.
78
pRIPK1 l.e.
78
Precision Plus Protein™ All blue prestained protein Standards
Precision Plus Protein™ All blue prestained protein Standards
M
M
kDa
kDa
250
250
150
150
100
100
75
75
50
50
37
37
25
25
20
20
15
15
10
10

## Slide 6
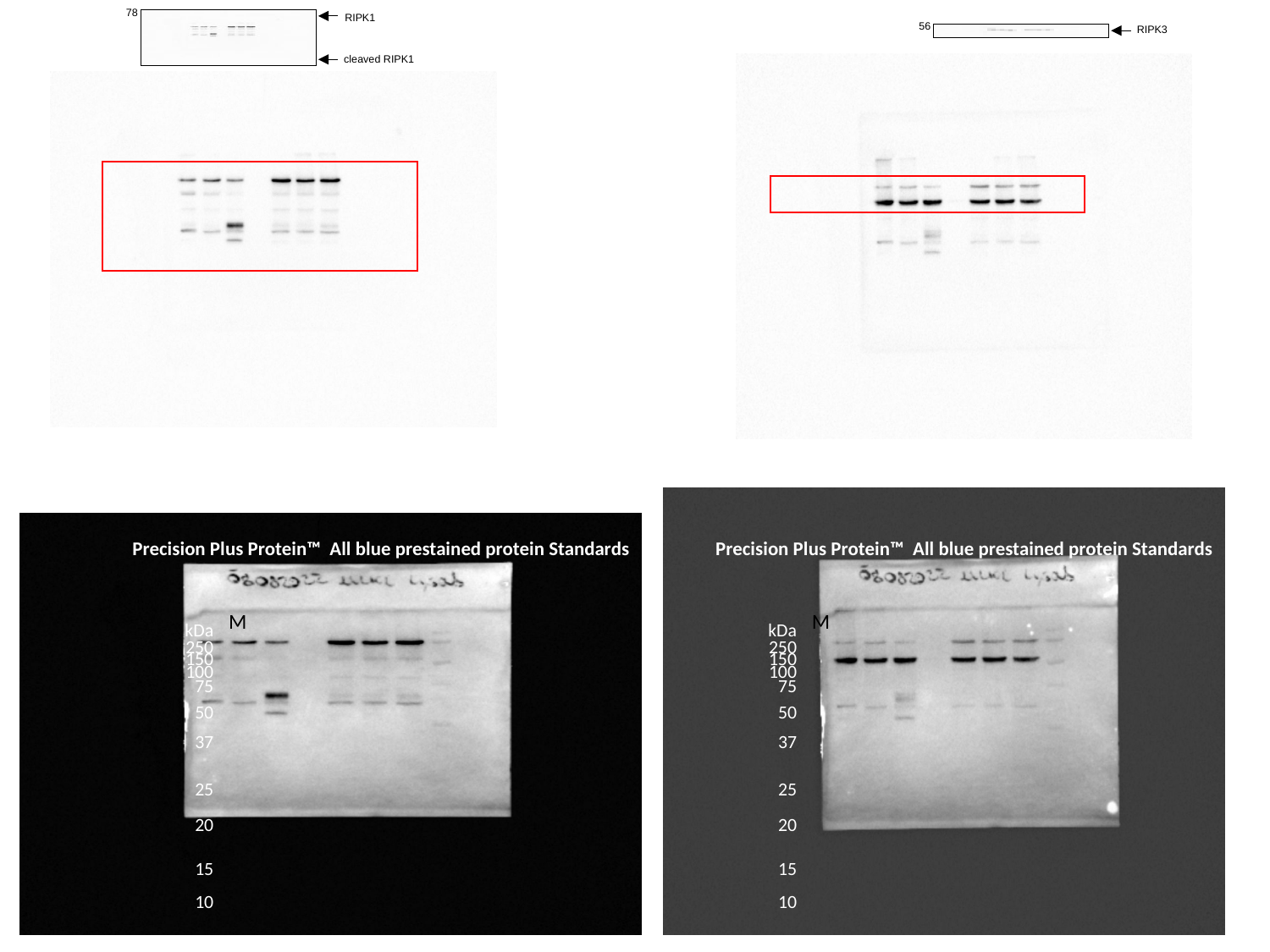

78
RIPK1
56
RIPK3
cleaved RIPK1
Precision Plus Protein™ All blue prestained protein Standards
Precision Plus Protein™ All blue prestained protein Standards
M
M
kDa
kDa
250
250
150
150
100
100
75
75
50
50
37
37
25
25
20
20
15
15
10
10

## Slide 7
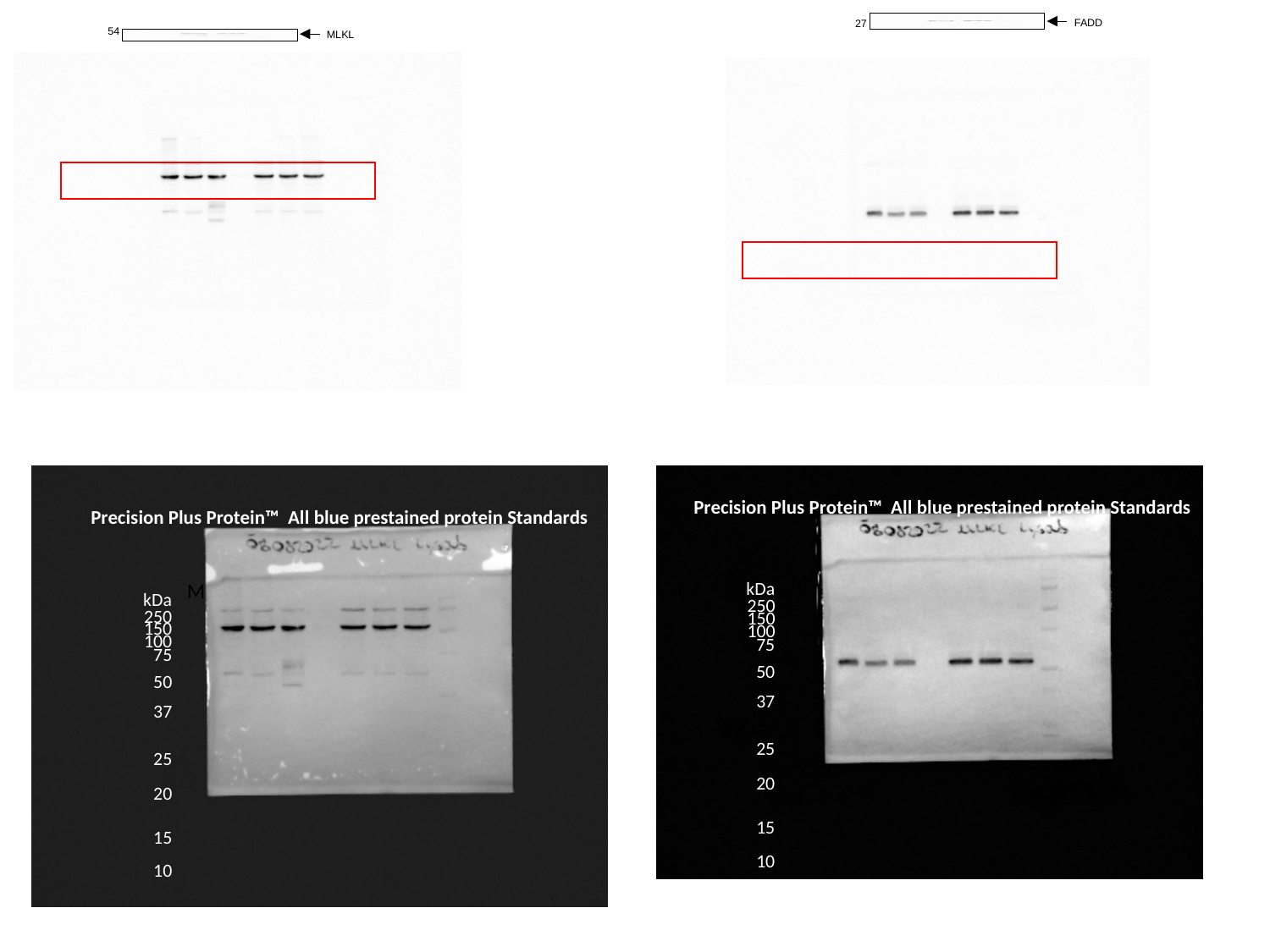

FADD
27
54
MLKL
Precision Plus Protein™ All blue prestained protein Standards
Precision Plus Protein™ All blue prestained protein Standards
M
kDa
M
kDa
250
250
150
150
100
100
75
75
50
50
37
37
25
25
20
20
15
15
10
10

## Slide 8
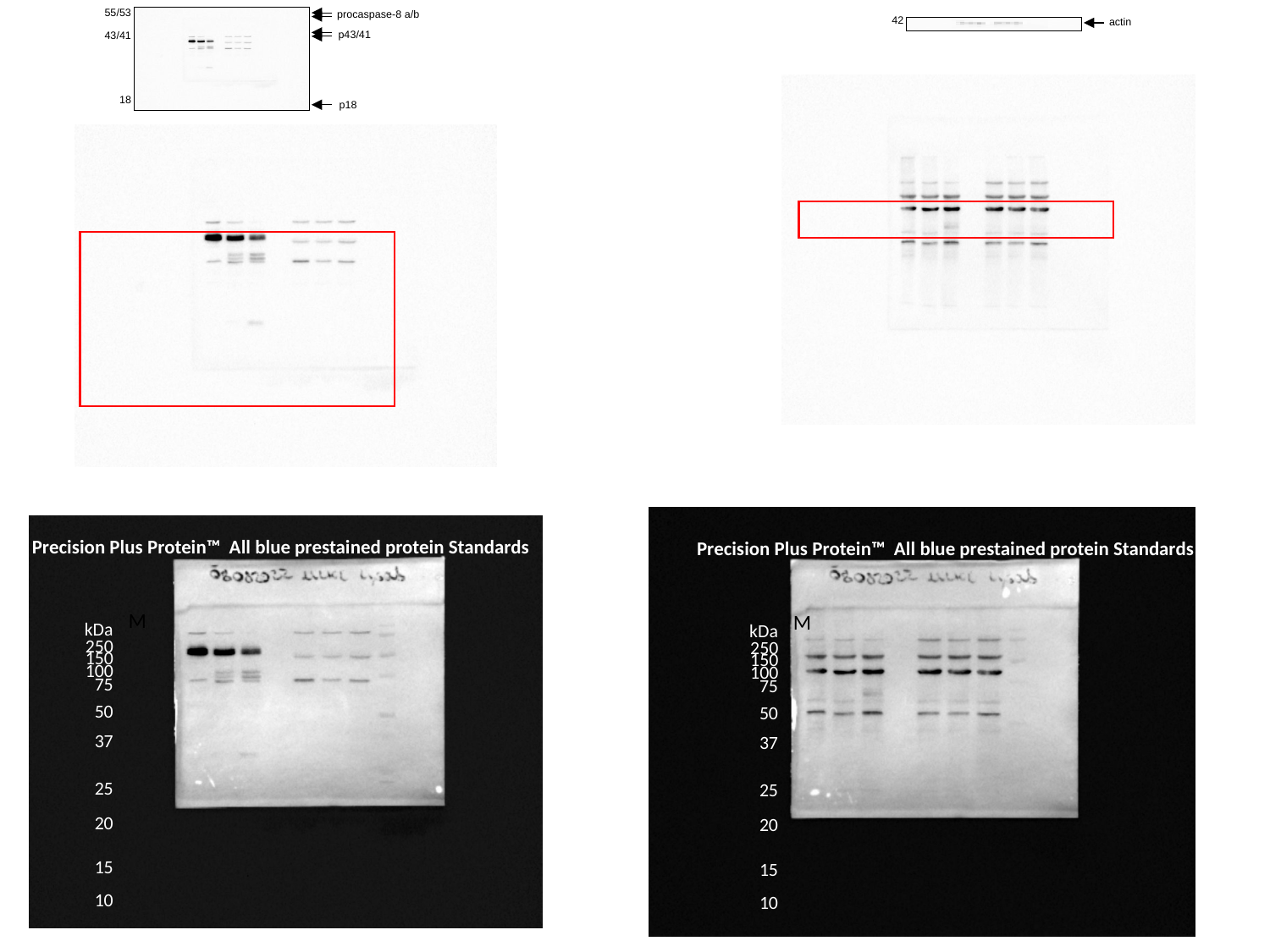

55/53
procaspase-8 a/b
42
actin
p43/41
43/41
18
p18
Precision Plus Protein™ All blue prestained protein Standards
Precision Plus Protein™ All blue prestained protein Standards
M
M
kDa
kDa
250
250
150
150
100
100
75
75
50
50
37
37
25
25
20
20
15
15
10
10

## Slide 9
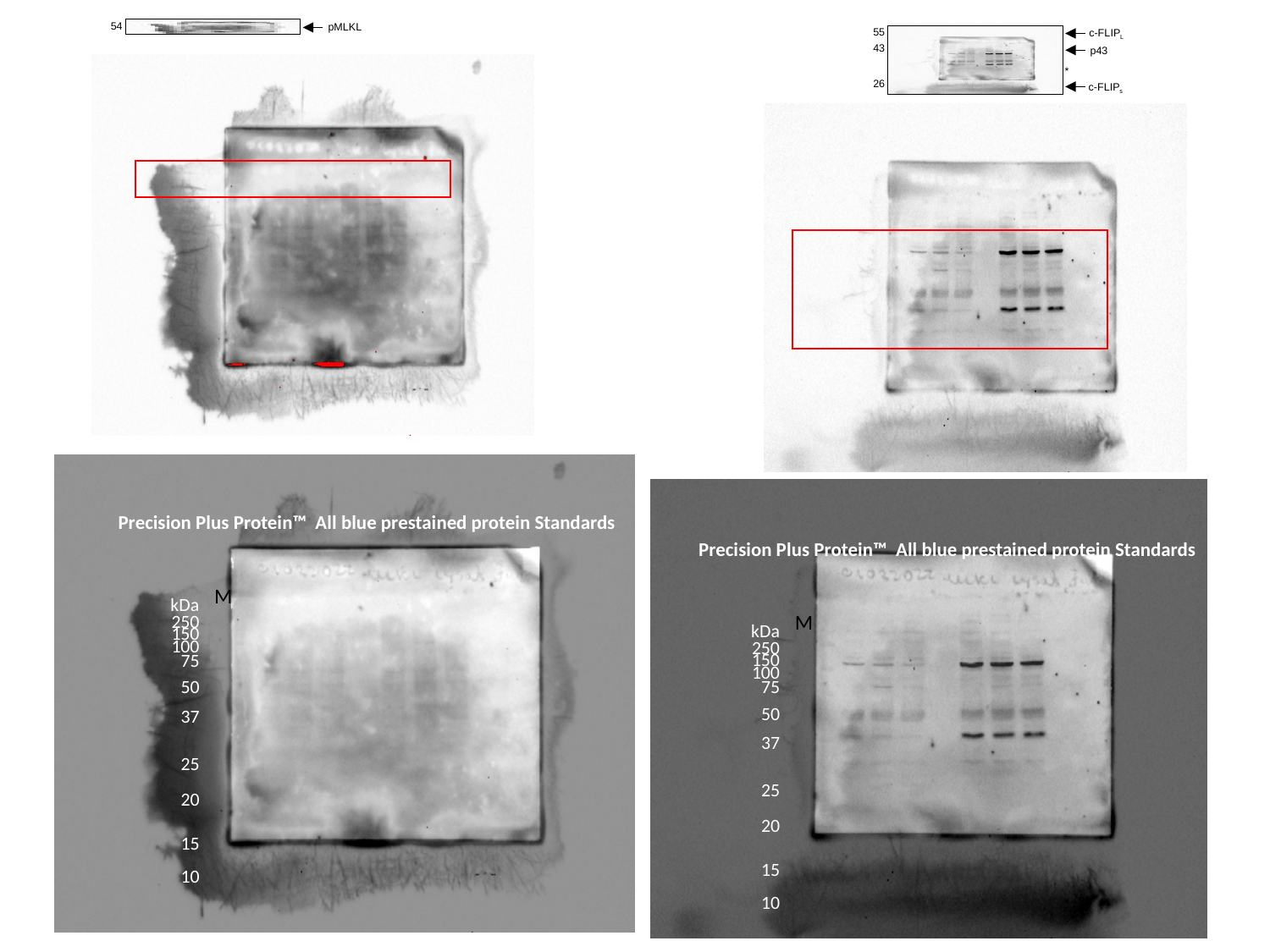

54
pMLKL
55
c-FLIPL
43
p43
*
26
c-FLIPs
Precision Plus Protein™ All blue prestained protein Standards
Precision Plus Protein™ All blue prestained protein Standards
M
kDa
M
250
kDa
150
100
250
150
75
100
75
50
50
37
37
25
25
20
20
15
15
10
10

## Slide 10
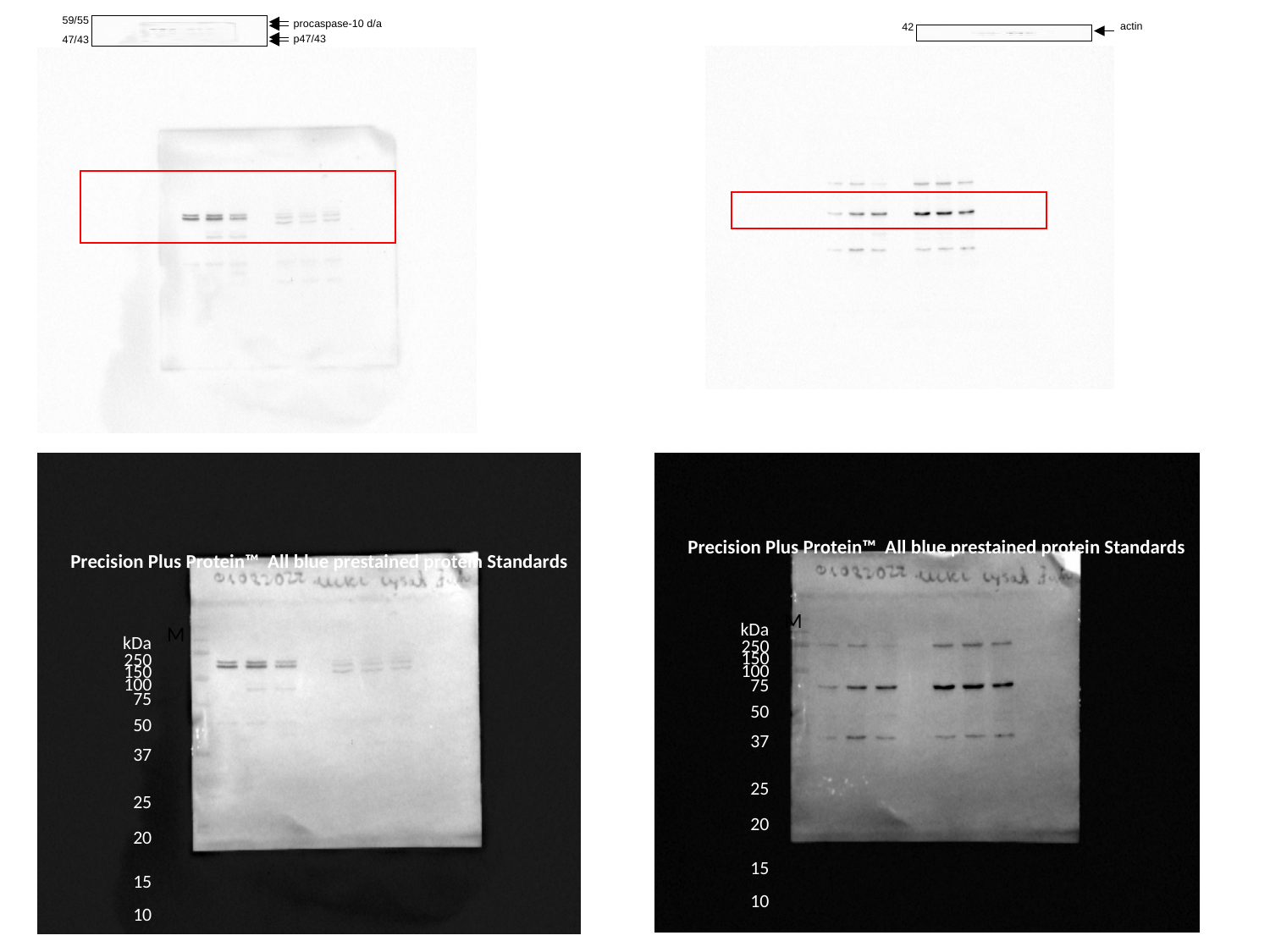

59/55
procaspase-10 d/a
actin
42
p47/43
47/43
Precision Plus Protein™ All blue prestained protein Standards
Precision Plus Protein™ All blue prestained protein Standards
M
kDa
M
kDa
250
150
250
100
150
100
75
75
50
50
37
37
25
25
20
20
15
15
10
10
