# Supplementary material for: Oligomerised RIPK1 is the main core component of the CD95 necrosome
Source: EMBO J. 2025 Apr 16;44(11):3231–65. doi: 10.1038/s44318-025-00433-0 (PMC12130296; doi:10.1038/s44318-025-00433-0)
Supplement: Supplementary file 15 — Expanded View Figures [file 44318_2025_433_MOESM15_ESM.pdf]

Expanded View Figures

Figure EV1. Combination of CD95L/BV6/zVAD-fmk leads to the appearance of necroptotic markers.

(**A**, **B**) HT29 (**A**) or SUIT-020 (**B**) cells were pretreated for 1 h with 5 µM BV6, 50 µM zVAD-fmk and 10 µM Nec-1s and subsequently treated with 500 ng/ml CD95L for the indicated timepoints. Total cellular lysates were analyzed using western blot with the indicated antibodies. Actin served as loading control. One representative experiment out of three is shown. (**C–H**) HT29 (**C**, **D**) and Jurkat 282 as well as Jurkat C8 ko (**E–H**) cells were treated for 1 h with 5 µM BV6, 50 µM zVAD-fmk, 10 µM Nec-1s or with indicated concentrations of GSK872 and subsequently treated with 500 ng/ml (**C**, **D**, **F**, **H**) or 100 ng/ml (**E**, **G**) CD95L, or medium as a control for 24 h. Cell viability was measured using the Cell Titer-Glo®-Luminescent Cell Viability Assay by Promega. The cell viability of untreated cells was taken as 100%. Mean and standard deviation from three independent experiments are shown. Statistics were calculated with unpaired one-way ANOVA with Tukey post hoc test to compare two conditions. Significance values: ****$P < 0.0001$; ***$P < 0.001$; **$P < 0.01$; *$P < 0.05$; ns not significant. P values from left to right for (**C**) $P < 0.0001$, $P > 0.9999$, $P < 0.0001$, $P < 0.0001$, $P < 0.0001$. (**D**) $P < 0.0001$, $P = 0.0499$ (**E**) $P = 0.0031$, $P = 0.0108$, $P < 0.0001$, $P < 0.0001$ (**F**) $P = 0.0018$, $P < 0.0001$, $P = 0.0004$, $P = 0.0002$ (**G**) $P = 0.0031$, $P = 0.0108$, $P < 0.0001$, $P < 0.0001$ (**H**) $P = 0.0018$, $P < 0.0001$, $P = 0.0004$, $P = 0.0002$. Source data are available online for this figure.

 © The Author(s)

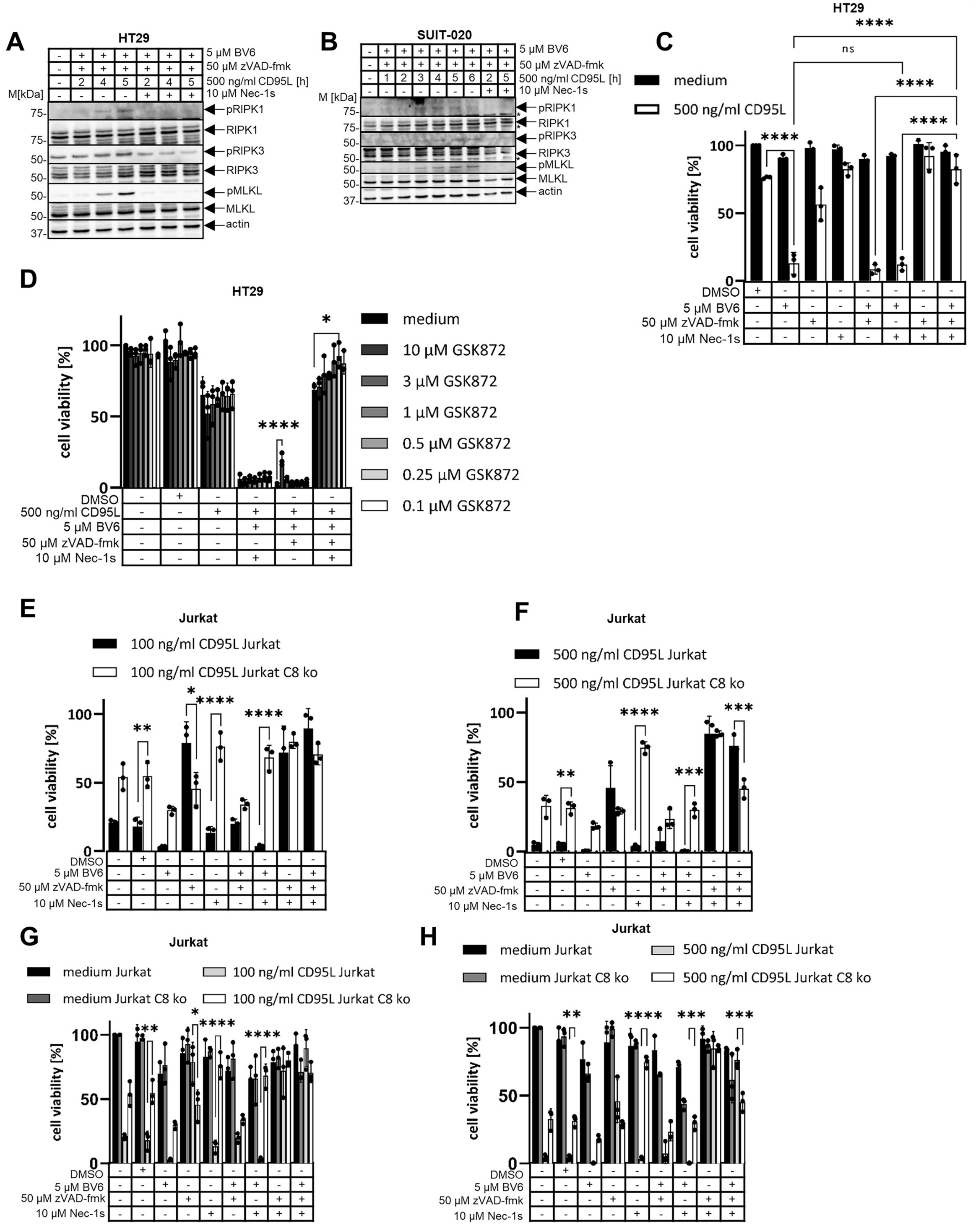
A
HT29
5 μM BV6
50 μM zVAD-fmk
500 ng/ml CD95L [h]
10 μM Nec-1s
M[kDa]
pRIPK1
RIPK1
pRIPK3
RIPK3
pMLKL
MLKL
actin
B
SUIT-020
5 μM BV6
50 μM zVAD-fmk
500 ng/ml CD95L [h]
10 μM Nec-1s
M [kDa]
pRIPK1
RIPK1
pRIPK3
RIPK3
pMLKL
MLKL
actin
C
HT29
medium
500 ng/ml CD95L
cell viability [%]
ns
****
DMSO
5 μM BV6
50 μM zVAD-fmk
10 μM Nec-1s
D
HT29
medium
10 μM GSK872
3 μM GSK872
1 μM GSK872
0.5 μM GSK872
0.25 μM GSK872
0.1 μM GSK872
cell viability [%]
DMSO
500 ng/ml CD95L
5 μM BV6
50 μM zVAD-fmk
10 μM Nec-1s
E
Jurkat
100 ng/ml CD95L Jurkat
100 ng/ml CD95L Jurkat C8 ko
cell viability [%]
DMSO
5 μM BV6
50 μM zVAD-fmk
10 μM Nec-1s
F
Jurkat
500 ng/ml CD95L Jurkat
500 ng/ml CD95L Jurkat C8 ko
cell viability [%]
DMSO
5 μM BV6
50 μM zVAD-fmk
10 μM Nec-1s
G
Jurkat
medium Jurkat
100 ng/ml CD95L Jurkat
medium Jurkat C8 ko
100 ng/ml CD95L Jurkat C8 ko
cell viability [%]
DMSO
5 μM BV6
50 μM zVAD-fmk
10 μM Nec-1s
H
Jurkat
medium Jurkat
500 ng/ml CD95L Jurkat
medium Jurkat C8 ko
500 ng/ml CD95L Jurkat C8 ko
cell viability [%]
DMSO
5 μM BV6
50 μM zVAD-fmk
10 μM Nec-1s

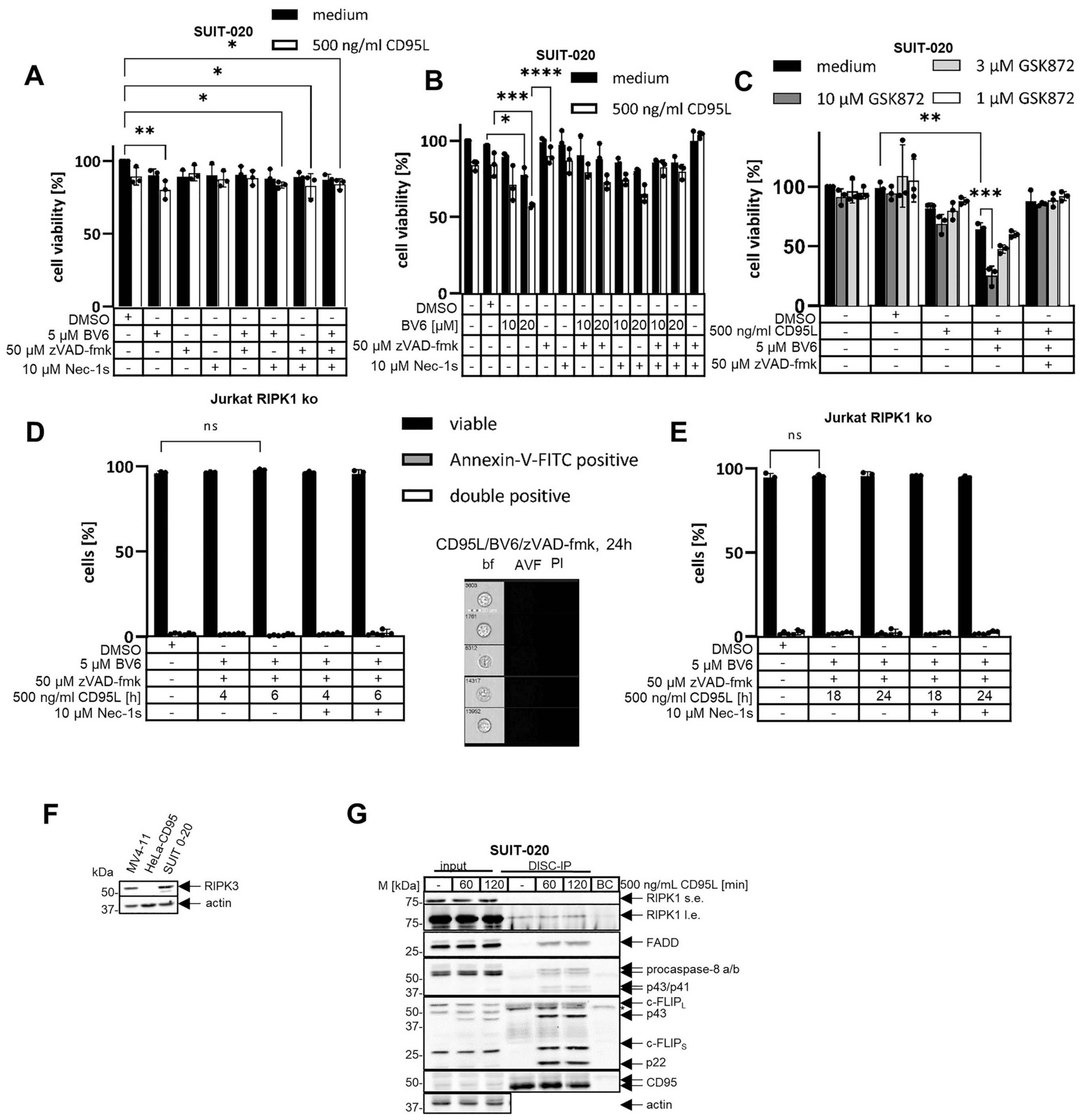
A
SUIT-020
medium
500 ng/ml CD95L
cell viability [%]
DMSO
5 µM BV6
50 µM zVAD-fmk
10 µM Nec-1s
B
SUIT-020
medium
500 ng/ml CD95L
cell viability [%]
DMSO
BV6 [µM]
50 µM zVAD-fmk
10 µM Nec-1s
C
SUIT-020
medium
10 µM GSK872
3 µM GSK872
1 µM GSK872
cell viability [%]
DMSO
500 ng/ml CD95L
5 µM BV6
50 µM zVAD-fmk
D
Jurkat RIPK1 ko
viable
Annexin-V-FITC positive
double positive
cells [%]
DMSO
5 µM BV6
50 µM zVAD-fmk
500 ng/ml CD95L [h]
10 µM Nec-1s
CD95L/BV6/zVAD-fmk, 24h
bf
AVF
PI
E
Jurkat RIPK1 ko
cells [%]
DMSO
5 µM BV6
50 µM zVAD-fmk
500 ng/ml CD95L [h]
10 µM Nec-1s
F
MV4-11
HeLa-CD95
SUIT 0-20
kDa
RIPK3
actin
G
SUIT-020
input
DISC-IP
M [kDa]
500 ng/mL CD95L [min]
BC
RIPK1 s.e.
RIPK1 l.e.
FADD
procaspase-8 a/b
p43/p41
c-FLIPL
p43
c-FLIPS
p22
CD95
actin

 © The Author(s)

Figure EV2. CD95L/BV6/zVAD-fmk co-treatment induces necroptosis in sensitive cells.

(**A–C**) SUIT-020 were pretreated with BV6, zVAD-fmk, Nec-1s and GSK872 with the indicated concentrations for 1 h. Afterwards, the cells were treated with 500 ng/ml CD95L for 24 h. Cell viability was measured using the Cell Titer-Glo®-Luminescent Cell Viability Assay by Promega. The cell viability of untreated cells was taken as 100%. (**D**, **E**) Jurkat RIPK1 ko cells were pretreated for 1 h with 5 μM BV6, 50 μM zVAD-fmk and 10 μM Nec-1s. Afterwards, the cells were treated with 500 ng/ml CD95L for the indicated time intervals. Cells were analyzed via imaging flow cytometry. Populations were gated for viable (negative), Annexin-V-FITC positive and double-positive (Annexin-V-FITC and PI positive) cells. Representative pictures from imaging flow cytometry with viable (negative) Annexin-V-FITC positive and double-positive (Annexin-V-FITC and PI positive) cells are shown in the middle. Mean and standard deviation from three independent experiments are shown. Statistics were calculated with unpaired one-way ANOVA with Tukey post hoc test to compare two conditions. Significance values: ****$P < 0.0001$; ***$P < 0.001$; **$P < 0.01$; *$P < 0.05$; ns not significant. *P* values from left to right for (**A**) $P = 0.0030$, $P = 0.0281$, $P = 0.0198$, $P = 0.0403$ (**B**) $P = 0.0173$, $P = 0.0002$, $P < 0.0001$ (**C**) $P = 0.0033$, $P = 0.0006$ (**D**) $P > 0.9999$ (**E**) $p > 0.9999$. (**F**) Total cellular lysates of MV4-11, HeLa-CD95 and SUIT-020 cells were tested for RIPK3 expression using western blot. HeLa-CD95 cells do not express RIPK3 and were used as a negative control. One representative western blot out of two is shown. Actin served as loading control. (**G**) SUIT-020 cells were with 500 ng/ml CD95L for indicated timepoints and DISC IP was analyzed by western blot. Actin served as loading control. BC beads-only control, IP immunoprecipitation, bf bright field, AVF Annexin-V-FITC, PI Propidium Iodide, * unspecific band, IgG_H the heavy chain of antibody. Source data are available online for this figure.

© The Author(s)

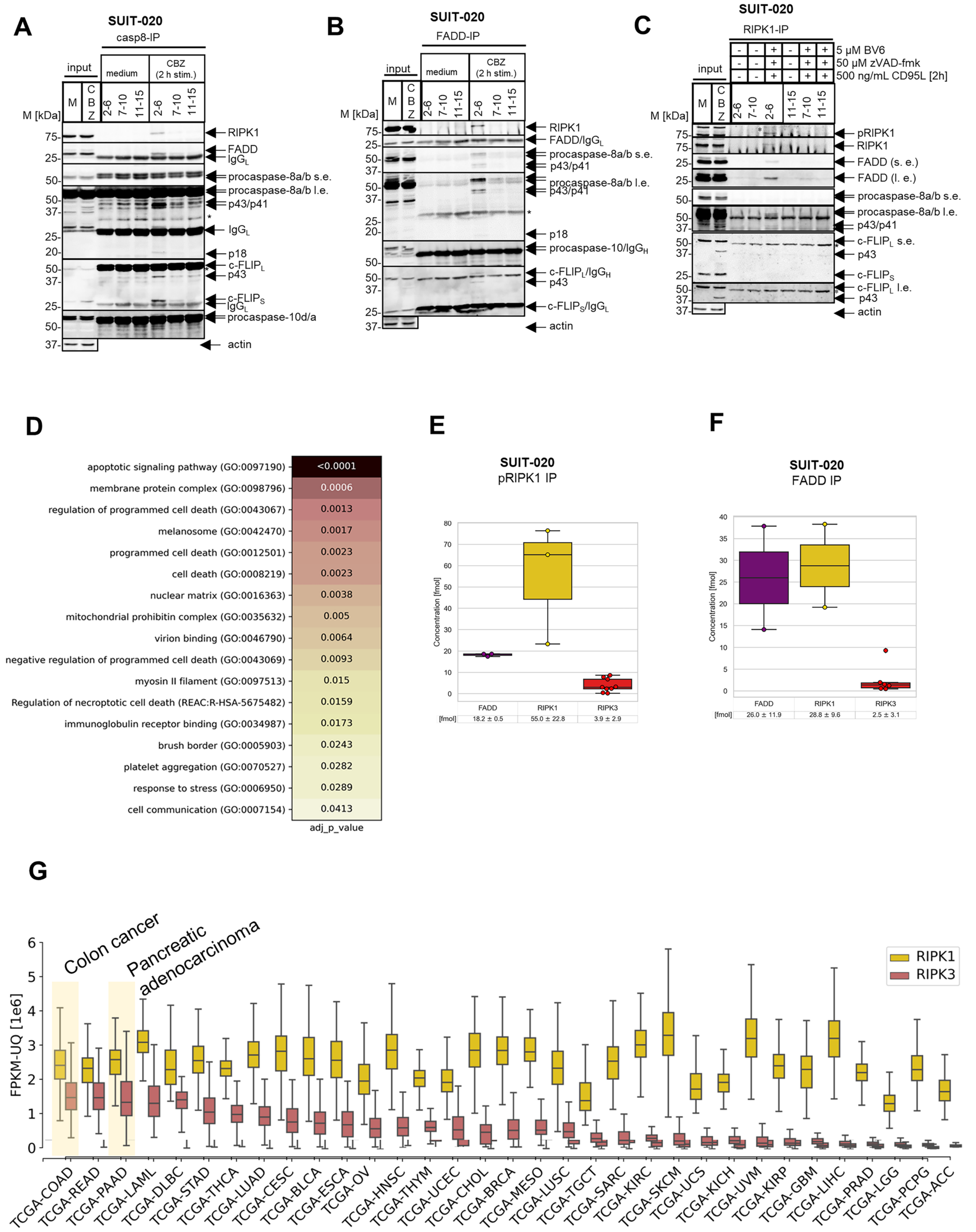
A
SUIT-020
casp8-IP
input
medium
CBZ (2 h stim.)
M [kDa]
RIPK1
FADD
IgGL
procaspase-8a/b s.e.
procaspase-8a/b l.e.
p43/p41
IgGL
p18
c-FLIPL
p43
c-FLIPS
IgGL
procaspase-10d/a
actin
B
SUIT-020
FADD-IP
RIPK1
FADD/IgGL
procaspase-8a/b s.e.
p43/p41
procaspase-8a/b l.e.
p43/p41
p18
procaspase-10/IgGH
c-FLIPL/IgGH
p43
c-FLIPS/IgGL
actin
C
SUIT-020
RIPK1-IP
5 µM BV6
50 µM zVAD-fmk
500 ng/mL CD95L [2h]
pRIPK1
RIPK1
FADD (s. e.)
FADD (l. e.)
procaspase-8a/b s.e.
procaspase-8a/b l.e.
p43/p41
c-FLIPL s.e.
p43
c-FLIPS
c-FLIPL l.e.
p43
actin
D
apoptotic signaling pathway (GO:0097190) <0.0001
membrane protein complex (GO:0098796) 0.0006
regulation of programmed cell death (GO:0043067) 0.0013
melanosome (GO:0042470) 0.0017
programmed cell death (GO:0012501) 0.0023
cell death (GO:0008219) 0.0023
nuclear matrix (GO:0016363) 0.0038
mitochondrial prohibitin complex (GO:0035632) 0.005
virion binding (GO:0046790) 0.0064
negative regulation of programmed cell death (GO:0043069) 0.0093
myosin II filament (GO:0097513) 0.015
Regulation of necroptotic cell death (REAC:R-HSA-5675482) 0.0159
immunoglobulin receptor binding (GO:0034987) 0.0173
brush border (GO:0005903) 0.0243
platelet aggregation (GO:0070527) 0.0282
response to stress (GO:0006950) 0.0289
cell communication (GO:0007154) 0.0413
adj_p_value
E
SUIT-020
pRIPK1 IP
Concentration [fmol]
FADD RIPK1 RIPK3
[fmol] 18.2 ± 0.5 55.0 ± 22.8 3.9 ± 2.9
F
SUIT-020
FADD IP
Concentration [fmol]
FADD RIPK1 RIPK3
[fmol] 26.0 ± 11.9 28.8 ± 9.6 2.5 ± 3.1
G
Colon cancer
Pancreatic adenocarcinoma
FPKM-UQ [1e6]
RIPK1
RIPK3
TCGA-COAD TCGA-READ TCGA-PAAD TCGA-LAML TCGA-DLBC TCGA-STAD TCGA-THCA TCGA-LUAD TCGA-CESC TCGA-BLCA TCGA-ESCA TCGA-OV TCGA-HNSC TCGA-THYM TCGA-UCEC TCGA-CHOL TCGA-BRCA TCGA-MESO TCGA-LUSC TCGA-TGCT TCGA-SARC TCGA-KIRC TCGA-SKCM TCGA-UCS TCGA-KICH TCGA-UVM TCGA-KIRP TCGA-GBM TCGA-LIHC TCGA-PRAD TCGA-LGG TCGA-PCPG TCGA-ACC

 © The Author(s)

Figure EV3. Necrosome detection in HMW fractions and AQUA peptide mass spectrometry analysis of the necrosome.

(**A–C**) SUIT-020 cells were prestimulated with 5 µM BV6 and 50 µM zVAD-fmk for 1 h and afterwards stimulated with 500 ng/mL CD95L for 2 h. Total cellular lysate was fractionated by gel filtration. The different fractions were pooled (2–6; 7–10, 11–15) followed by casp8-IP (**A**), FADD-IP (**B**) or RIPK1-IP (**C**). Total cellular lysates and IPs were analyzed by western blot and probed for the indicated proteins. Actin was used as a loading control for total cellular lysates (input). One representative experiment out of two is shown. (**D**) Groups of proteins identified by mass spectrometry analysis were analyzed by GO bioinformatics analysis. IPs from CBZ-treated cells (FADD-IP/CBZ, FLIP-IP/CBZ, pRIPK1-IP/CBZ) were analyzed against IPs from untreated cells (FADD-IP/control, FLIP-IP/control, pRIPK-IP/control and beads-only control). The major groups of proteins are shown. The full analysis is presented in Dataset EV2. The lowest significance is shown in black, the highest in light yellow. The proteins from the necroptotic cell death group have a similar significance to the proteins from the myosin group and immunoglobulin, which always have a high abundance in the IP experiments. (**E**, **F**) SUIT-020 cells were prestimulated with 5 µM BV6 and 50 µM zVAD-fmk for 1 h and afterwards stimulated with 500 ng/mL CD95L for 2 h. IP was done using anti-pRIPK1 (**E**) or anti-FADD (**F**) antibodies. The IPs were analyzed by AQUA peptide-based mass spectrometry analysis. The amounts of the AQUA peptides (fmols) corresponding to each protein, that were detected in the IP, are shown. Mean and standard deviations from three experiments are shown. Box plots show the distribution of the data using the median (center line), interquartile range (IQR; box limits represent the first and third quartiles, Q1 and Q3) and whiskers extending from the lower and upper quartiles to 1.5 × IQR. Points outside the whiskers are considered outliers and are shown as single points. (**G**) Gene expression levels in cancer tissues, as obtained from the TCGA data portal (https://www.cancer.gov/tcga). Gene expression levels were obtained using the RNA-Seq technique. IP immunoprecipitation, CBZ CD95L/BV6/zVAD-fmk, s.e. short exposure, l.e. long exposure, * unspecific band, IgG_L the light chain of antibody. Source data are available online for this figure.

© The Author(s)

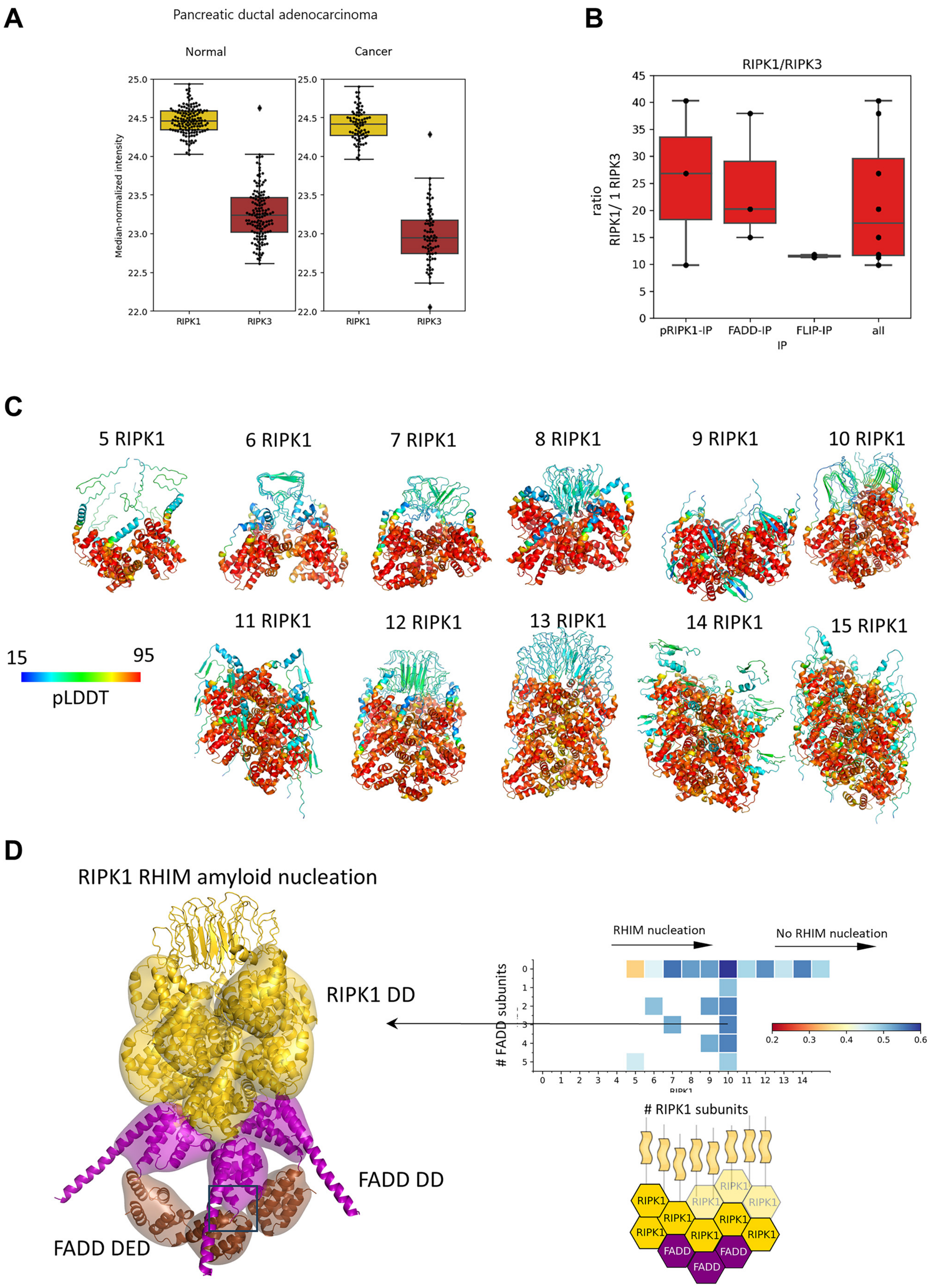
A
Pancreatic ductal adenocarcinoma
Normal
Cancer
Median-normalized intensity
RIPK1
RIPK3
B
RIPK1/RIPK3
ratio
RIPK1/ 1 RIPK3
pRIPK1-IP
FADD-IP
FLIP-IP
all
IP
C
5 RIPK1
6 RIPK1
7 RIPK1
8 RIPK1
9 RIPK1
10 RIPK1
11 RIPK1
12 RIPK1
13 RIPK1
14 RIPK1
15 RIPK1
15
95
pLDDT
D
RIPK1 RHIM amyloid nucleation
RIPK1 DD
FADD DD
FADD DED
RHIM nucleation
No RHIM nucleation
FADD subunits
RIPK1 subunits
RIPK1
FADD

 © The Author(s)

Figure EV4. Necrosome stoichiometry analysis by mass spectrometry and AlphaFold3 modeling of necrosome.

(**A**) Comparison of RIPK1 and RIPK3 expression levels from normal and cancer pancreatic ductal adenocarcinoma. (**B**) HT29 cells were pretreated with 5 µM BV6 and 50 µM zVAD-fmk for 1 h. Afterwards cells were stimulated with 500 ng/mL CD95L for 5 h, which was followed by pRIPK1-IP, FADD-IP or c-FLIP-IP. The IPs were analyzed by mass spectrometry analysis. The results are presented in the Fig. 5. Box plots show the distribution of the data using the median (center line), interquartile range (IQR; box limits represent the first and third quartiles, Q1 and Q3) and whiskers extending from the lower and upper quartiles to 1.5 × IQR. Points outside the whiskers are considered outliers and are shown as single points. The average ratios of proteins were calculated using interquartile range and presented in this panel. (**C**) Molecular models of RIPK1 (DD-RHIM) predicted by AlphaFold3 for various oligomerization states, with models colored according to the confidence metric pLDDT. (**D**) Molecular models of RIPK1 (DD-RHIM) and FADD predicted by AlphaFold3. The heatmap on the right shows AlphaFold3 ipTM scores for different stoichiometries of FADD and RIPK1 proteins, with colors ranging from red (low confidence) to blue (high confidence). The molecular model of a 10 RIPK1/3 FADD complex, as predicted by AlphaFold3, is shown on the left.

© The Author(s)

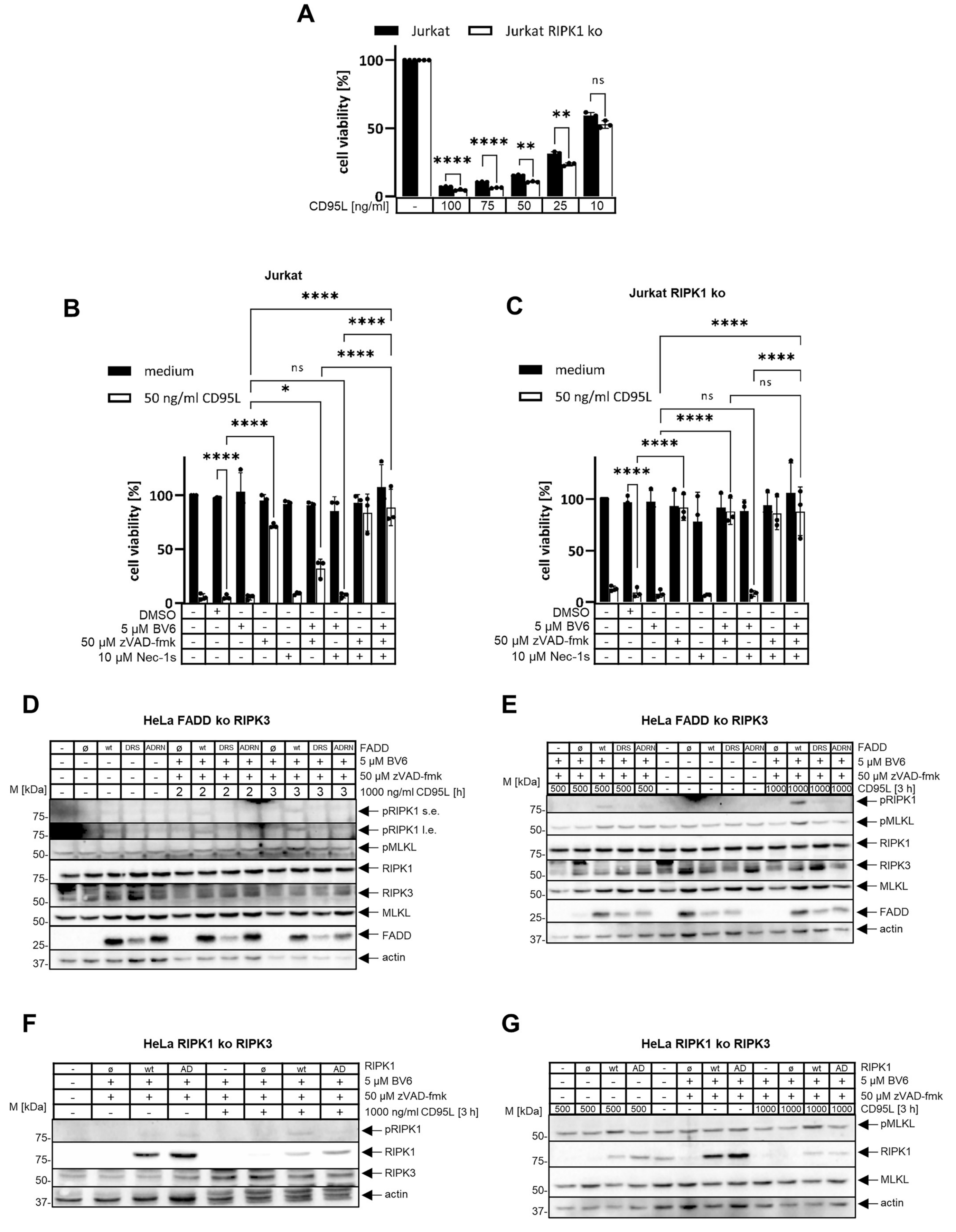
A
Jurkat
Jurkat RIPK1 ko
cell viability [%]
100
50
0
CD95L [ng/ml]
-
100
75
50
25
10
ns
B
Jurkat
medium
50 ng/ml CD95L
C
Jurkat RIPK1 ko
DMSO
5 µM BV6
50 µM zVAD-fmk
10 µM Nec-1s
D
HeLa FADD ko RIPK3
E
HeLa FADD ko RIPK3
FADD
1000 ng/ml CD95L [h]
CD95L [3 h]
M [kDa]
pRIPK1 s.e.
pRIPK1 l.e.
pMLKL
RIPK1
RIPK3
MLKL
actin
F
HeLa RIPK1 ko RIPK3
G
HeLa RIPK1 ko RIPK3
1000 ng/ml CD95L [3 h]
pRIPK1
wt
DRS
ADRN
AD

 © The Author(s)

Figure EV5. The analysis of the role of the mutations in FADD and RIPK1 on necroptosis induction.

(**A**) Jurkat A3 and Jurkat A3 RIPK1 ko cells were treated with indicated concentrations of CD95L for 24 h. (**B**, **C**) Jurkat A3 (**B**) or Jurkat A3 RIPK1 ko (**C**) cells were prestimulated with 5 µM BV6, 50 µM zVAD-fmk and 10 µM Nec-1s for 1 h and afterwards stimulated with 50 ng/mL CD95L for 24 h. Cell viability was measured using the Cell Titer-Glo®-Luminescent Cell Viability Assay by Promega. The cell viability measurements of untreated cells were taken as 100%. Mean and standard deviation from three independent experiments are shown. Statistics were calculated with unpaired one-way ANOVA with Tukey post hoc test to compare two conditions. Significance values: ****$P < 0.0001$; ***$P < 0.001$; **$P < 0.01$; *$P < 0.05$; ns not significant. (**D**, **E**) HeLa FADD ko RIPK3 cells were transfected with empty vector, WT-FADD, FADD-DRS (L172D, D175R, L176S) or FADD-ADRN (M170A, L172D, D175R, L176N). Transfected cells were pretreated with 5 µM BV6, 50 µM zVAD-fmk or 10 µM Nec-1s for 1 h and afterwards stimulated with 500 or 1000 ng/mL CD95L for 3 h or as indicated. (**F**, **G**) HeLa RIPK1 ko RIPK3 cells were transfected with empty vector, WT-RIPK1 or RIPK1-AD (M637A, I641D). Transfected cells were pretreated with 5 µM BV6 and 50 µM zVAD-fmk for 1 h and afterwards stimulated with 500 or 1000 ng/mL CD95L for 3 h. Total cellular lysates were analyzed using western blot with the indicated antibodies. Actin served as loading control. One representative western blot out of three is shown. WT wild type, - untransfected, ø empty vector, s.e. short exposure, l.e. long exposure. Source data are available online for this figure.

© The Author(s)
